# Population genomics of the critically endangered kākāpō

**DOI:** 10.1016/j.xgen.2021.100002

**Published:** 2021-09-08

**Authors:** Nicolas Dussex, Tom van der Valk, Hernán E. Morales, Christopher W. Wheat, David Díez-del-Molino, Johanna von Seth, Yasmin Foster, Verena E. Kutschera, Katerina Guschanski, Arang Rhie, Adam M. Phillippy, Jonas Korlach, Kerstin Howe, William Chow, Sarah Pelan, Joanna D. Mendes Damas, Harris A. Lewin, Alex R. Hastie, Giulio Formenti, Olivier Fedrigo, Joseph Guhlin, Thomas W.R. Harrop, Marissa F. Le Lec, Peter K. Dearden, Leanne Haggerty, Fergal J. Martin, Vamsi Kodali, Françoise Thibaud-Nissen, David Iorns, Michael Knapp, Neil J. Gemmell, Fiona Robertson, Ron Moorhouse, Andrew Digby, Daryl Eason, Deidre Vercoe, Jason Howard, Erich D. Jarvis, Bruce C. Robertson, Love Dalén

**Affiliations:** 1 Centre for Palaeogenetics, Svante Arrhenius väg 20C, 10691 Stockholm, Sweden; 2 Department of Bioinformatics and Genetics, Swedish Museum of Natural History, Box 50007, 10405 Stockholm, Sweden; 3 Department of Zoology, Stockholm University, 10691 Stockholm, Sweden; 4 Department of Anatomy, University of Otago, PO Box 913, Dunedin 9016, New Zealand; 5 Section for Evolutionary Genomics, GLOBE Institute, University of Copenhagen, Copenhagen, Denmark; 6 Department of Zoology, University of Otago, PO Box 56, Dunedin 9054, New Zealand; 7 Department of Biochemistry and Biophysics, National Bioinformatics Infrastructure Sweden, Science for Life Laboratory, Stockholm University, Box 1031, 17121 Solna, Sweden; 8 Institute of Evolutionary Biology, School of Biological Sciences, University of Edinburgh, Edinburgh, UK; 9 Department of Ecology and Genetics, Animal Ecology, Uppsala University, 75236 Uppsala, Sweden; 10 Genome Informatics Section, Computational and Statistical Genomics Branch, National Human Genome Research Institute, National Institutes of Health, Bethesda, MD 20892, USA; 11 Pacific Biosciences, 1305 O’Brien Drive, Menlo Park, CA 94025, USA; 12 Wellcome Sanger Institute, Wellcome Trust Genome Campus, Hinxton CB10 1SA, UK; 13 Department of Evolution and Ecology and the UC Davis Genome Center, 4321 Genome and Biomedical Sciences Facility, University of California Davis, Davis, CA 95616, USA; 14 Bionano Genomics, 9540 Towne Centre Drive, San Diego, CA 92121, USA; 15 Vertebrate Genome Laboratory, The Rockefeller University, New York, NY 10065, USA; 16 Laboratory of Neurogenetics of Language, Box 54, The Rockefeller University, New York, NY 10065, USA; 17 Howard Hughes Medical Institute, Chevy Chase, MD 20815, USA; 18 Genomics Aotearoa and Laboratory for Evolution and Development, Department of Biochemistry, University of Otago, PO Box 56, Dunedin 9016, New Zealand; 19 European Molecular Biology Laboratory, European Bioinformatics Institute, Wellcome Genome Campus, Hinxton, Cambridge CB10 1SD, UK; 20 National Center for Biotechnology Information, National Library of Medicine, National Institutes of Health, Bethesda, MD 20894, USA; 21 The Genetic Rescue Foundation, Wellington, New Zealand; 22 Kākāpō Recovery, Department of Conservation, PO Box 743, Invercargill 9840, New Zealand; 23 BioSkryb Genomics, 701 W Main Street, Suite 200, Durham, NC 27701, USA

**Keywords:** kākāpō, mutational load, inbreeding, purging, bottleneck, conservation

## Abstract

The kākāpō is a flightless parrot endemic to New Zealand. Once common in the archipelago, only 201 individuals remain today, most of them descending from an isolated island population. We report the first genome-wide analyses of the species, including a high-quality genome assembly for kākāpō, one of the first chromosome-level reference genomes sequenced by the Vertebrate Genomes Project (VGP). We also sequenced and analyzed 35 modern genomes from the sole surviving island population and 14 genomes from the extinct mainland population. While theory suggests that such a small population is likely to have accumulated deleterious mutations through genetic drift, our analyses on the impact of the long-term small population size in kākāpō indicate that present-day island kākāpō have a reduced number of harmful mutations compared to mainland individuals. We hypothesize that this reduced mutational load is due to the island population having been subjected to a combination of genetic drift and purging of deleterious mutations, through increased inbreeding and purifying selection, since its isolation from the mainland ∼10,000 years ago. Our results provide evidence that small populations can survive even when isolated for hundreds of generations. This work provides key insights into kākāpō breeding and recovery and more generally into the application of genetic tools in conservation efforts for endangered species.

## Highlights

First genome sequencing and population genomic analyses of the critically endangered kākāpō
Generation of a high-quality annotated reference genome assembly for kākāpō
Sequencing of 49 kākāpō genomes from surviving island and extinct mainland populations
Genomic analyses of long-term isolated populations inform conservation strategies

## Graphical abstract

**Figure:**
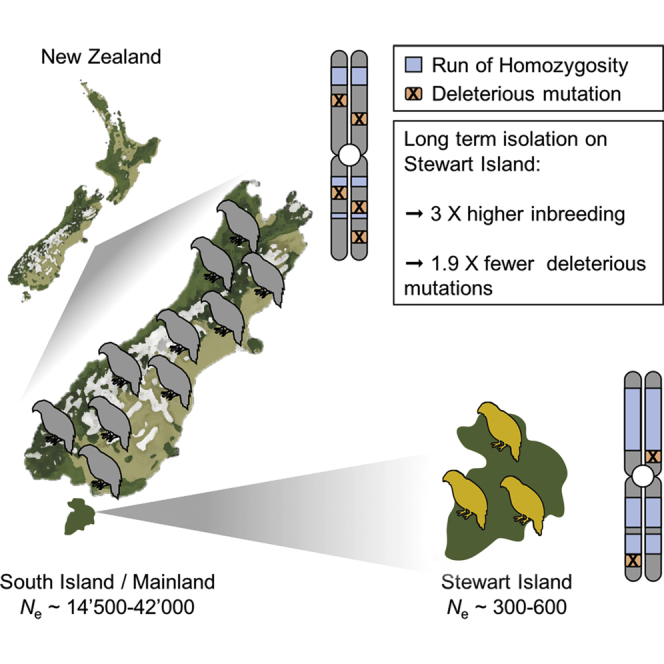

## Teaser

Dussex et al. sequence modern and historical genomes from the critically endangered kākāpō to investigate the genetic consequences of long-term small population size. They find that the remaining kākāpō from a small island population are inbred but have fewer deleterious mutations compared to the now-extinct mainland population, providing insights into conservation strategies.

## Introduction

New Zealand was one of the last landmasses colonized by humans.^1^ Following Polynesian colonization circa 1360 CE and European colonization in the 1800s, and the resulting overhunting and introduction of mammalian predators, New Zealand experienced major extinction events of endemic species.^2^ The kākāpō (*Strigops habroptilus*), a flightless parrot species, was widespread before human arrival and likely numbered in the hundreds of thousands.^3^ By 1995, the species was reduced to 51 birds, 50 kākāpō from the isolated Stewart Island and one single male, named Richard Henry, from the extinct mainland population.^4^ Richard Henry and 39 Stewart Island birds were the only kākāpō to reproduce and are thus the ancestors of all birds born since 1995. As of 2021, a total of 201 kākāpō survive and are managed on island sanctuaries. Previous studies indicate that kākāpō have lost ∼70%–80% of their genetic diversity since the 1800s and have elevated levels of inbreeding.^3^^,^^5^ Poor sperm quality and low hatching success^6^ suggest that kākāpō carry deleterious mutations as a consequence of genetic drift and reduced efficacy of purifying selection, a clear evidence of reduced fitness (i.e., genetic load^7^^,^^8^). However, the genome-wide impact of the severe population bottleneck of the kākāpō remains unknown.

Population genetic theory suggests that, in small populations, genetic load may accumulate and increase the risk of extinction via “mutational meltdown.”^8^ However, because the effects of observed mutations on fitness are often unknown, estimating genetic load is challenging. Recent studies based on genomic data from temporally spaced samples of extinct and endangered species^9^, ^10^, ^11^, ^12^, ^13^, ^14^ have instead focused on the accumulation of deleterious mutations by examining increases in mutational load. However, theory and empirical data suggest that mutational load can also be purged in long-term isolated and inbred populations when selection against recessive or partially recessive detrimental alleles is increased as they are expressed in the homozygous state.^15^ To date, although simulations and comparisons among species or subspecies have shown this to be possible in rare examples (see, for example, Robinson et al.^16^ and Grossen et al.^17^), studies on the purging of mutational load in the wild and between recently diverged populations remain scarce.

To test these hypotheses, we compared the genomes of surviving and extinct kākāpō populations, representing the first population genomics analysis of kākāpō in the context of the Kākāpo 125+ project. Here, we present the reference genome for kākāpō, one of the first chromosome-level reference genomes sequenced by the Vertebrate Genomes Project (VGP),^18^ and a population genomics analysis of 49 kākāpō from Stewart Island and the extinct mainland population. Our population genomic analyses indicate that present-day island kākāpō have a reduced number of predicted deleterious mutations compared to mainland individuals. We suggest that this reduced mutational load may have resulted from a combination of genetic drift and purging of deleterious mutations through increased inbreeding and purifying selection in the island population since their isolation from the mainland ∼10,000 years ago. Our findings will aid in the development of genetic approaches to support the recovery of kākāpō and contribute to informing future breeding programs and translocation efforts. More generally, such population genomic analyses of other endangered species will be useful to inform those conservation efforts.

## Results

### Island and mainland populations are distinct and separated after the last glaciation

**Figure 1:**
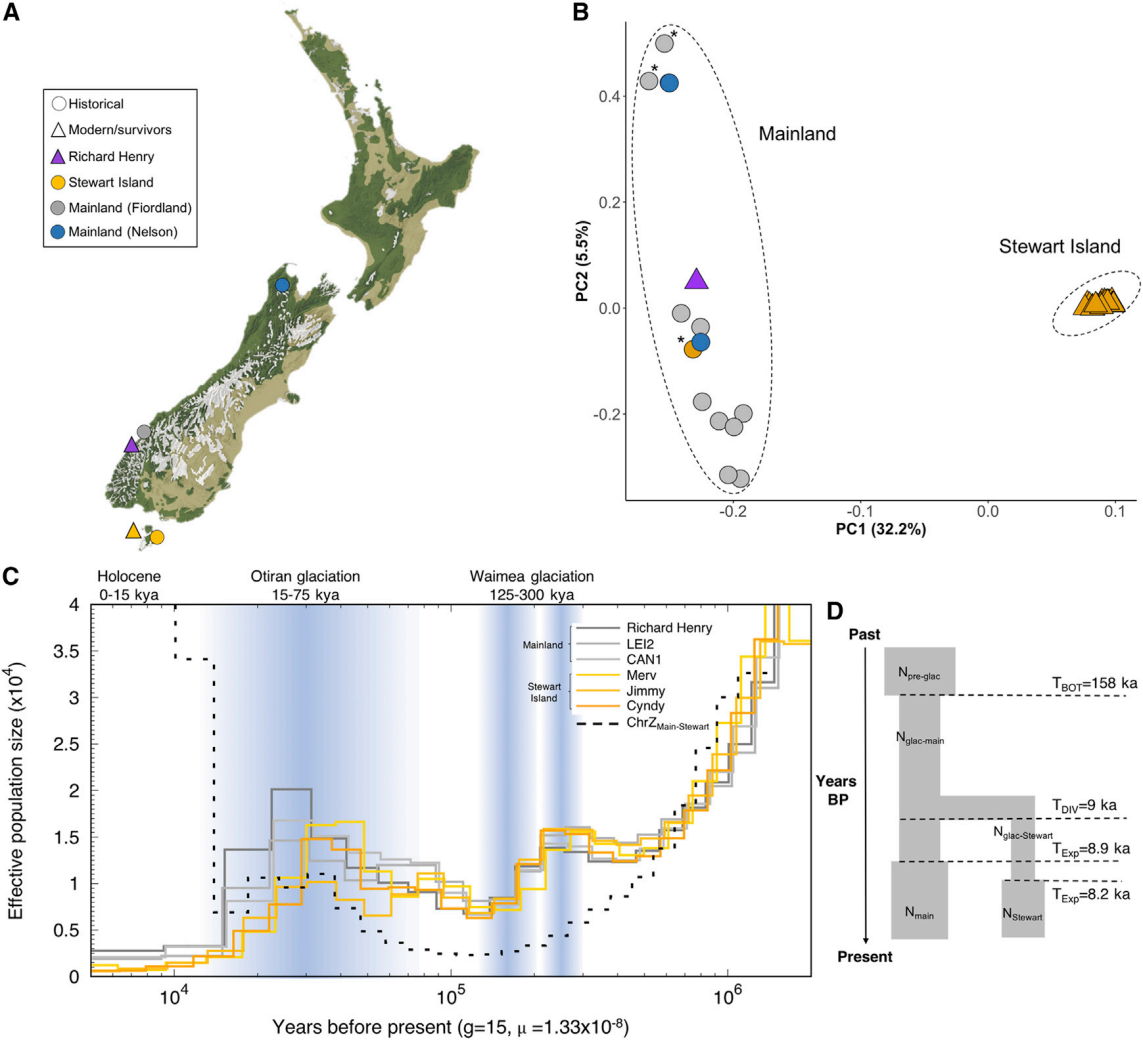
Sampling locations, population structure, and past demography of kākāpō (A) Sampling locations for historical and modern specimens on a map showing the vegetation cover circa 1840.^19^ (B) Principal-component analysis (PCA) for 14 mainland and 35 Stewart Island kākāpō. Asterisks indicate museum samples likely to have been mislabeled (STAR Methods). (C) Demographic history and divergence time between the mainland and Stewart Island population inferred using the PSMC method. Each colored curve represents an individual bird. The black dashed curve represents the sex chromosome comparison (i.e., Z chromosome), with population size reaching infinity at the time of divergence between the two populations. (D) Parameter estimates for a scenario of post-glacial population divergence and expansion using ABC. See also Figures S5–S10 and Tables S2 and S3.

We generated a high-quality chromosome-level genome assembly for a female kākāpō (Figures S1–S3) and sequenced the genomes from 36 individuals that survived the bottleneck at its most severe phase in the 1990s (Richard Henry and 35 Stewart Island birds) as well as 13 genomes from ∼130-year-old museum specimens that originated from the extinct mainland population (Figure 1A; Table S1). No offspring from the surviving individuals that subsequently founded the present-day population were included in the analyses. Principal-component analysis (PCA) of the 49 re-sequenced genomes identified genetic distinctions between the mainland and Stewart Island populations (Figures 1B and S5). We found evidence for historical samples’ mislabeling and subsequently analyzed samples according to their genetically assigned population when estimating differences in inbreeding and mutational load (STAR Methods).

We performed pairwise sequentially Markovian coalescent (PSMC) analysis to track changes in effective population sizes (*N*_e_) over time (STAR Methods). Analyses of the genomes from the mainland and Stewart Island populations showed nearly identical demographic histories marked by a severe decline in *N*_e_, starting some 30,000 years (30 ka) before present (BP; Figure 1C), a period coinciding with the onset of the Last Glacial Maximum (LGM).^20^ By the end of the last glaciation ∼10 ka BP, kākāpō *N*_e_ had declined from ∼15,000–20,000 to a minimum of ∼1,000–3,000. Since the PSMC has reduced power to estimate recent changes in *N*_e_ (i.e., <10 ka BP^21^), we also performed approximate Bayesian computation (ABC) simulations and demographic reconstructions using MSMC2 (Figures S6–S9), which are better suited to infer recent demography. These analyses also supported a demographic decline during the LGM (Figure 1D, S8 and S9; Tables S2 and S3). Moreover, Late Holocene *N*_e_ estimates from the MSMC2 and ABC were similar, with *N*_e_ ∼300–600 for Stewart Island and *N*_e_ ∼14,500–42,000 for the mainland population (Figures S8 and S9; Table S3).

Historical accounts and a lack of fossil remains originally suggested that kākāpō were introduced to Stewart Island in the past ∼500 years, by either Maōri or European settlers.^4^^,^^22^ However, based on our ABC simulations and analyses of coalescence rates between Z chromosomes, we find that the divergence time between the mainland and the Stewart Island populations dates back to the end of the last glaciation (Figures 1C, 1D, S8, and S10). This timing coincides with the isolation of Stewart Island from the mainland as sea levels rose at the end of the Pleistocene some 12 ka BP.^23^ Thus, our analyses suggest that instead of having been established by humans in the past ∼500 years, the Stewart Island population constitutes a distinct lineage that has been isolated from the mainland for up to 1,000 generations.

### The island population is highly inbred

**Figure 2:**
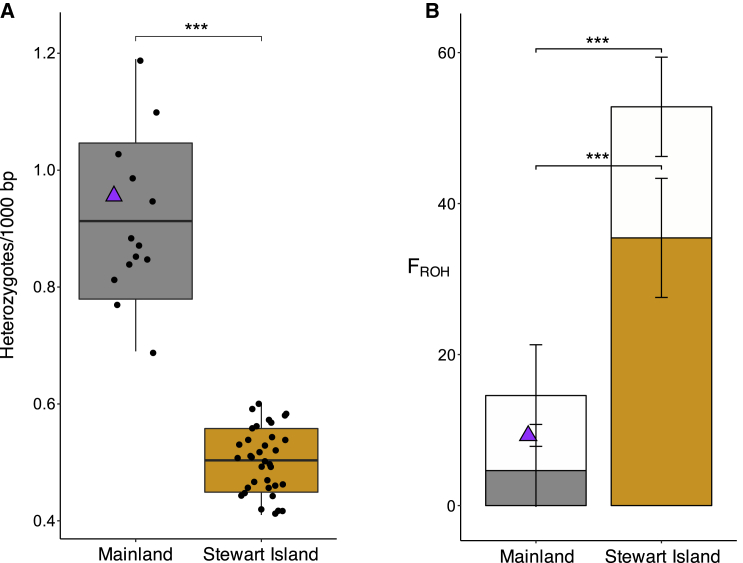
Heterozygosity and inbreeding estimates for kākāpō (A) Individual genome-wide heterozygosity. Horizontal lines within boxplots and bounds of boxes represent means and standard deviations, respectively. Vertical lines represent minima and maxima. (B) Individual inbreeding coefficients estimated using ROH (F_ROH_). Open bars show the total proportion of the genome in ROH ≥ 100 kb and solid bars show proportions in ROH ≥ 2 Mb. Bars extending from the mean values represent the standard deviation (Welch’s two-sample t test; ∗∗∗p < 0.001). Richard Henry is shown with a purple triangle. See also Figures S11–S15.

The long-term isolation and reduced *N*_e_ since the end of the last glaciation and the severe decline in the past 150 years on Stewart Island due to introduced predators^4^ may have led to the additional loss of genetic diversity via genetic drift. Supporting this hypothesis, we find much lower average individual heterozygosity (Figure 2A) and lower population-level nucleotide diversity (*π*; Figure S11) in the genomes of the Stewart Island birds. Furthermore, based on a high-quality dataset of variants covered in all individuals, we find that extended runs of homozygosity (F_ROH_), an expected outcome of inbreeding, also differed markedly between the populations. Stewart Island kākāpō had, on average, 53% of their genome sequence in ROH ≥ 100 kb, while mainland kākāpō had 15% (Figure 2B). Genome proportions comprising very long ROH (≥2 Mb) were, on average, 34% and 4% for Stewart Island and mainland kākāpō, respectively (Figures 2B and S12–S15). Such comparatively long ROH indicate recent mating between closely related individuals, most likely during the past ten generations.^24^ Overall, these results are in agreement with the long-term insular isolation at low *N*_e_ as well as a recent decline in the past few hundred years.

### Genomes from Stewart Island kākāpō have comparatively fewer deleterious mutations

**Figure 3:**
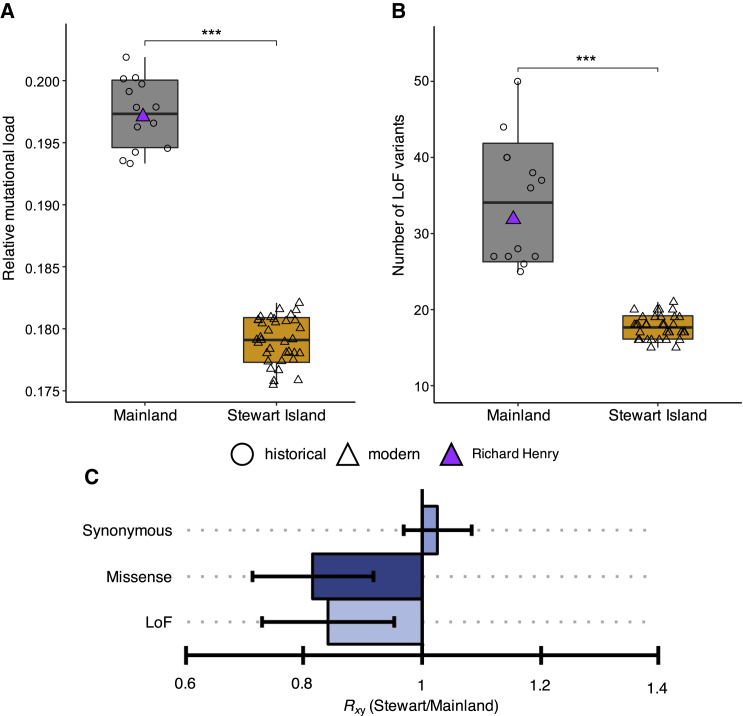
Mutational load estimates for kākāpō (A) Individual relative mutational load measured as the sum of all homozygous and heterozygous derived alleles multiplied by their conservation score (GERP score > 2) over the total number of derived alleles. (B) Number of loss of function (LoF) variants per individual. Horizontal lines within boxplots and bounds of boxes represent means and standard deviations, respectively (Welch’s two-sample t test; ∗∗∗p < 0.001). Vertical lines represent minima and maxima. (C) *R*_xy_ ratio of derived alleles for synonymous, missense, and LoF variants. *R*_xy_ < 1 indicates a relative frequency deficit of the corresponding category in Stewart Island compared to mainland kākāpō. Whiskers represent 95% confidence interval (CI). See also Figures S16–S22.

To test the hypotheses of small populations being affected by either increased mutational load due to strong drift causing a reduced efficacy of purifying selection in removing deleterious mutations^8^ or purging of recessive deleterious mutations as a consequence of increased inbreeding,^15^ we estimated the mutational load in mainland and Stewart Island kākāpō. First, we measured individual mutational load as the number of derived alleles at sites that are under strict evolutionary constraints, predicted as likely to be deleterious using genomic evolutionary rate profiling (GERP) scores (Figures S16 and S17; STAR Methods). These results indicate ∼1.1 times lower mutational load in the genomes of Stewart Island compared to mainland kākāpō (Figure 3A). Moreover, the difference in the number of deleterious alleles was most pronounced at sites under the strongest evolutionary constraint (Figures S18 and S19), consistent with the purging of deleterious mutations in the Stewart Island population.

Second, we estimated mutational load in each individual by identifying variants in our annotation of 15,699 coding genes (STAR Methods). Similar to the GERP analysis, we find ∼1.9 times fewer mutations classified as highly deleterious (i.e., predicted loss of function [LoF]) in Stewart Island relative to the mainland kākāpō population (Figures 3B, S20, and S21), with, on average, 17.6 and 34.1 predicted LoF variants per bird genome for the Stewart Island and mainland population, respectively (Figure 3B). The ratio of derived alleles (*R*_xy_) between the two populations also showed reduced LoF and missense variants in the Stewart Island population compared to the mainland population (Figure 3C). Furthermore, the mainland population had a higher number of LoF alleles in the heterozygous state compared to the Stewart Island population (Figures S20 and S21). This suggests that many of these mutations are primarily deleterious in the homozygous state, consistent with theoretical predictions.^15^ We also found a significantly lower number of LoF alleles inside ROH compared to heterozygous parts of the genomes, and this difference was 3-fold smaller in the Stewart Island population (Figure S22), suggesting that repeated inbreeding may have facilitated the removal of heterozygous LoF alleles.^25^

**Figure 4:**
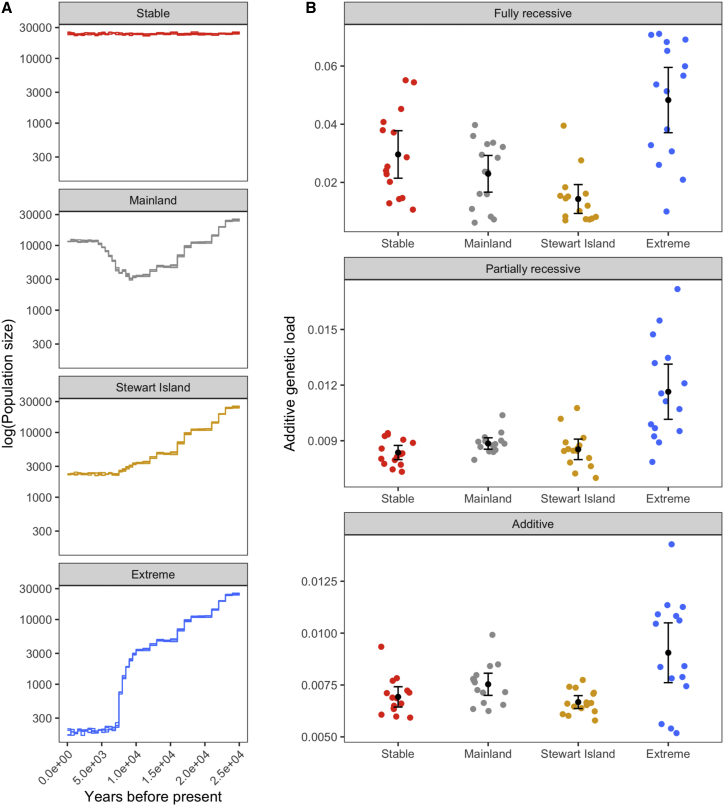
Forward simulations of demographic scenarios and impact on deleterious mutations (A) Simulated demographic scenarios representing a Stable scenario as control (*N*_e_ = 10,000), a Mainland scenario (LGM bottleneck and long-term *N*_e_ = 6,000), a Stewart Island scenario (LGM decline and long-term *N*_e_ = 1,000), and an Extreme decline scenario (LGM decline and long-term *N*_e_ = 100). (B) Additive genetic load calculated as the sum of selection coefficients for homozygous mutations plus the sum of selection coefficients multiplied by the dominance coefficients for heterozygous mutations. Black dots and whiskers show the means and 95% CIs for each demographic scenario. See also Figures S23–S27.

To further test whether our results are consistent with the purging of deleterious mutations, we performed forward simulations recapitulating the demographic history of the mainland and Stewart Island populations (Figure 4A). We also simulated scenarios for hypothetical stable and severely bottlenecked populations to model a weak and strong effect of drift, respectively (STAR Methods). When assuming a scenario consistent with the history of the Stewart Island population, our simulations indicated that the additive genetic load (Figure 4B) and the number of deleterious alleles were significantly reduced compared to simulations recapitulating the mainland population’s history (t test, p < 0.01; Figures S23–S26), particularly for mildly and strongly deleterious mutations. This result is slightly different from a previous study on Alpine Ibex (*Capra ibex*), which found evidence for the purging of highly deleterious mutations but an accumulation of mildly deleterious mutations.^17^ When assuming an extreme population decline, our simulations indicated an increase in additive genetic load (Figure 4B), consistent with strong drift increasing the number and expression of deleterious mutations in homozygous state (Figures S23–S26). These simulations thus suggest that purging requires a large enough population for selection to be effective, whereas in a population that is too small, drift will overwhelm purifying selection. Overall, these results are consistent with the hypothesis that purifying selection has led to purging in the Stewart Island population since its isolation from the mainland some 15–20 ka BP.

### Functional consequences of deleterious mutations in modern kākāpō

Our findings highlight that the identification of variants with deleterious effects in the surviving kākāpō population is of critical conservation relevance as these variants will help assess the beneficial and detrimental effects of mixing the extinct mainland and extant Stewart Island genetic lineages.^26^ While our results are consistent with a historically high rate of purging of mutational load in Stewart Island kākāpō, the present-day kākāpō population likely still suffers from inbreeding depression, as indicated by the generally low hatching success and poor sperm quality in a large number of males.^6^ Moreover, because surviving birds originate from two distinct populations with different levels of mutational load, an assessment of the relative mutational load contributed by the Stewart Island survivors, Richard Henry, and his descendants may be valuable to guide future conservation actions.^27^ We therefore examined the predicted functional consequences of the identified LoF variants (STAR Methods). Analyzing the genomes of all modern Stewart Island birds, we identified predicted LoF variants in 61 genes (Tables S6 and S7). We observed predicted LoF variants in genes that have been associated with reproduction (BUB1), development (e.g., BMPR1A, FLT1), and immunity (e.g., IGDCC4; Tables S6 and S7), consistent with observations of low reproductive and hatching success in kākāpō.^6^ Interestingly, we found predicted LoF variants in 27 other genes associated with immunity and development (e.g., FLT1) only in the genomes of Stewart Island birds and 21 unique predicted LoF variants in the genome of Richard Henry in genes associated with, for instance, reproduction (e.g., BUB1) and development (e.g., LYN; Table S6).

## Discussion

Our population genomic analyses indicate that Stewart Island kākāpō are more inbred but have a lower mutational load than mainland kākāpō. One explanation for this reduced mutational load is that random genetic drift led to a loss of alleles that were at low frequency before the decline in population size on Stewart Island. However, while most deleterious alleles at low frequency will be lost due to random drift, a small proportion will be fixed, meaning that the average frequency of deleterious alleles will not change. Because we observed a reduced relative frequency of missense and LoF variants in the Stewart Island population (Figure 3C), we therefore consider a scenario in which mutational load was reduced through drift alone less likely.^8^

An alternative, and in our view more probable, explanation is that a combination of inbreeding and purifying selection contributed to the reduction in mutational load in the Stewart Island population.^15^ This interpretation is also supported by the finding of a less pronounced difference in LoF alleles within and outside ROH in the genomes from Stewart Island kākāpō, possibly indicating a reduction in LoF through repeated inbreeding events. Moreover, our forward simulations demonstrated that additive genetic load and the number of deleterious alleles can be reduced in a scenario that recapitulates the demographic history of the Stewart Island population.

Our results suggest that a long-term isolation and slow increase in inbreeding may have offered circumstances for the purging of mutational load in the Stewart Island population.^15^ However, it is important to point out that purging in the Stewart Island population is more likely a reflection of its long-term small *N*_e_ and that the more recent severe decline may now be exposing the population to the same level of drift load as in our simulated extreme decline scenario.

Previous empirical studies on populations that have experienced population declines have in some cases identified increases in mutational load.^9^^,^^10^ However, there are also multiple studies that have identified a reduced mutational load following population decline, similar to what we observed in the Stewart Island kākāpō.^10^^,^^17^^,^^25^^,^^28^ These contrasting results have important implications since they suggest a complex interaction between population declines and the trajectory of deleterious genetic variation, thus making generalizations across species challenging.

The importance of genetic tools in kākāpō recovery has been increasingly recognized over the past 20 years.^27^ Since the translocation of all surviving kākāpō to predator-free islands in the 1980s, efforts have been made to maintain genetic diversity, reduce inbreeding, and avoid the spread of harmful traits.^4^^,^^27^ Management actions have focused not only on reducing inbreeding by preventing pairings between related individuals^27^^,^^29^ but also on the maintenance of evolutionary potential by favoring matings with birds of mainland genetic heritage (i.e., Richard Henry and his offspring^27^). While mixing distinct genetic lineages can result in genetic rescue in highly inbred populations,^26^ our results show that Richard Henry has a higher mutational load than birds from the Stewart Island population. Even though the fitness and ecological effects of these deleterious mutations are unknown, mixing between the mainland and Stewart Island lineages could lead to the introduction of additional mutational load and thus be detrimental to the viability of the remaining population.^26^ Furthermore, the current extremely low population size could be conducive to reduced efficacy of selection and lead to the fixation of deleterious mutations in future generations (i.e., increased drift load). Isle Royal wolves are a natural example of genetic rescue with such unintended negative consequences.^30^ Here, the migration of a single male wolf into this small and isolated population resulted in a population decline associated with the introduction of detrimental variation.^16^^,^^30^ It is thus crucial to balance the positive (i.e., genetic rescue and maintenance of adaptive potential) and negative (i.e., increase in the proportion and expression of mutational load) effects that could result from mixing the two kākāpō genetic lineages and, if necessary, limit breeding between them.^31^ Our findings reinforce the need to further examine the genetic basis of inbreeding depression in the extant kākāpō population, in particular with relation to traits associated with fertility and hatching success.

Breeding programs and translocation efforts for other endangered taxa that have experienced severe anthropogenic population declines would benefit greatly from the type of genomic data analyzed here. For instance, evidence of inbreeding depression has been observed also in the New Zealand stitchbird (*Notiomystis cincta*).^32^ Similarly, Tasmanian devil (*Sarcophilus harrisii*) populations display very low genetic diversity and high incidence of a tumor disease (Tasmanian devil facial tumor disease [DFTD]).^33^ Because captive breeding and/or translocations are part of the management strategy of these species, assessing the mutational load of potential candidates for breeding and translocations will be essential for the success of their conservation.

## STAR★Methods

### Key resources table

**Table:** 

REAGENT or RESOURCE	SOURCE	IDENTIFIER
**Chemicals, peptides, and recombinant proteins**		

Tango Buffer (10X)	ThermoFisher Scientific	Cat#BY5
ATP (100mM)	ThermoFisher Scientific	Cat#R0441
T4 Polynucleotide Kinase (10U/ul)	ThermoFisher Scientific	Cat#EK0032
T4 DNA Polymerase 5U/ul	ThermoFisher Scientific	Cat#EP0062
USER Enzyme	NEB	Cat#M5505L
T4 DNA Ligase (5U/ul)	ThermoFisher Scientific	Cat#EL0011
Bst Polymerase, Large Fragments	NEB	Cat#M0275S
AccuPrime Pfx	ThermoFisher Scientific	Cat#12344024
T4 DNA ligase (400U/ul)	NEB	Cat#M0202S
T4 DNA polymerase (3U/ul)	NEB	Cat#M0203S
PEG-4000	Sigma-Aldrich	Cat#95904-250G-F

**Critical commercial assays**		

High Sensitivity DNA kit	Agilent	Cat#5067-4626
DNeasy Blood & Tissue Kit	QIAGEN, Hilden, Germany	Cat#69504

**Deposited data**		

Raw fastq reads	This study	Historical resequencing data: ENA:PRJEB35522; modern resequencing data: https://repo.data.nesi.org.nz/TAONGA-KAKAPO
*de-novo* assembly for *Strigops habroptilus*	This study	GenBank: GCF_004027225.2 and GCA_004011185.1; https://ftp.ncbi.nlm.nih.gov/genomes/all/GCF/004/027/225/GCF_004027225.2_bStrHab1.2.pri/GCF_004027225.2_bStrHab1.2.pri_genomic.fna.gz

**Oligonucleotides**		

IS1 adaptor P5: 5′- A∗C∗A∗C∗TCTTTCCCTACACG ACGCTCTTCCG∗A∗T∗C∗T-3′	Meyer and Kircher^34^; Sigma-Aldrich	N/A
IS2 adaptor P7: 5′- G∗T∗G∗A∗CTGGAGTTCAGAC GTGTGCTCTTCCG∗A∗T∗C∗T-3′	Meyer and Kircher^37^; Sigma-Aldrich	N/A
IS3 adaptor P5+P7: 5′- A∗G∗A∗T∗CGGAA∗G∗A∗G∗C-3′	Meyer and Kircher^37^; Sigma-Aldrich	N/A
Illumina AmplifyingPrimer IS4: 5′- AATGATACGGCG ACCACCGAGATCTACACTCTTTCCCTACACGACG CTCTT-3′	Meyer and Kircher^37^; Sigma-Aldrich	N/A
Illumina Indexing Primer: 5′- CAAGCAGAAGACGGC ATACGAGATNNNNNNNGTGACTGGAGTTCAGAC GTGT-3′	Meyer and Kircher^37^; Sigma-Aldrich	N/A
Ns represent indexes		N/A

**Software and algorithms**		

VGP pipeline	Rhie et al.^18^	https://vertebrategenomesproject.org/
BLAST+ 2.5.0	Camacho et al.^35^	NCBI
Qualimap v2.2.1	Okonechnikov et al.^36^	http://qualimap.bioinfo.cipf.es/
CpG site masking script	von Seth et al.^14^; Lord et al.^37^	https://github.com/tvdvalk/find_CpG
RepeatMasker v4.0.7	Smit et al.^38^	http://repeatmasker.org
MESPA pipeline	Neethiraj et al.^39^	https://sourceforge.net/projects/mespa/
BRAKER v2.1.1	Hoff et al.^40^; Stanke et al.^41^^,^^42^	https://github.com/Gaius-Augustus/BRAKER
SPALN2	Iwata and Gotoh^43^	https://github.com/ogotoh/spaln
cufflinks v 2.2.1	Trapnell et al.^44^^;^ Roberts et al.^45^	http://cole-trapnell-lab.github.io/cufflinks/
eggNOG-mapper v4.5.1	Huerta-Cepas et al.^46^	http://eggnog-mapper.embl.de/
bcl2Fastq v1.17.1	Illumina	https://support.illumina.com/sequencing/sequencing_software/bcl2fastq-conversion-software.html
SeqPrep	John^47^	https://github.com/jstjohn/SeqPrep
BWA v0.7.13	Li and Durbin^48^	http://bio-bwa.sourceforge.net/
SAMtools v1.3	Li et al.^49^	https://sourceforge.net/projects/samtools/files/samtools/1.3/
Picard v1.141	Broad Institute	http://broadinstitute.github.io/picard
Mapdamage v2.0	Jónsson et al.^50^	https://ginolhac.github.io/mapDamage/
Trimmomatic v0.32	Bolger et al.^51^	http://www.usadellab.org/cms/?page=trimmomatic
GATK v3.4.0	McKenna et al.^52^	https://gatk.broadinstitute.org/hc/en-us
bcftools v1.3	Li^53^	http://www.htslib.org/
BEDtools v2.29.2	Quinlan and Hall^54^	https://bedtools.readthedocs.io/en/latest/
PLINK v1.9	Purcell et al.^55^	https://www.cog-genomics.org/plink2/
SNPRelate	Zheng et al.^56^	https://www.bioconductor.org/packages/release/bioc/html/SNPRelate.html
ADMIXTURE v1.3.0	Alexander et al.^57^	http://dalexander.github.io/admixture/publications.html
RapidNJ v2.3.2	Simonsen et al.^58^	https://anaconda.org/bioconda/rapidnj
Timetree	Kumar et al.^68^	http://timetree.org/
PSMC v0.6.5	Li and Durbin^21^	https://github.com/lh3/psmc
Fastsimcoal v2.6	Excoffier and Foll ^59^; Excoffier et al.^60^	http://cmpg.unibe.ch/software/fastsimcoal2/
PGDspider	Lischer and Excoffier^61^	http://www.cmpg.unibe.ch/software/PGDSpider/
Arlequin v3.5	Excoffier and Lischer^62^	http://cmpg.unibe.ch/software/arlequin35/
MSMC2	Schiffels and Wang ^63^	https://github.com/stschiff/msmc2
Beagle v5.1	Browning and Browning^64^	https://faculty.washington.edu/browning/beagle/beagle.html
vcftools	Danecek^65^	http://vcftools.sourceforge.net/
mlRho v2.7	Haubold et al.^66^	http://guanine.evolbio.mpg.de/mlRho/
R	R Development Core Team^84^	https://www.r-project.org/
GERP++	Davydov et al.^67^	http://mendel.stanford.edu/sidowlab/downloads/gerp/index.html
htsbox v1.0	N/A	https://github.com/lh3/htsbox
SNPeff v4.3	Cingolani et al.^68^	http://snpeff.sourceforge.net/index.html
Pilon v1.22	Walker et al.^69^	https://github.com/broadinstitute/pilon/releases/tag/v1.22
Panther v16.0	Mi et al.^70^	http://www.pantherdb.org/
SLiM 3	Haller and Messer^71^; Kim et al.^72^	https://messerlab.org/slim/

**Other**		

Proteinase K	VWR	Cat#1.24568.0100
dNTPs	VWR	Cat#733-1854
Min Elute PCR purification Kit	QIAGEN	Cat#28006
Agencourt AmPure XP 5mL Kit	Beckman Coulter	Cat#63880

### Resource availability

#### Lead contact

Further information and requests for resources and reagents should be directed to and will be fulfilled by the lead contact, Nicolas Dussex (nicolas.dussex@gmail.com).

#### Materials availability

This study did not generate new unique reagents.

### Experimental model and subject details

The sample for the reference kākāpō genome was from a female named Jane (deceased in 2018 of natural causes), collected as part of the G10K-VGP Project, avian B10K Project, and the Kākāpō Recovery Program. We obtained modern genomic data sequenced from blood DNA extracts by the Kākāpō 125+ Project for one mainland male (i.e., Richard Henry) and 35 birds from Stewart Island discovered on Stewart Island in the 1980s (Table S1). Out of these 36 modern birds, 28 birds, including Richard Henry, were founders of the current population and are thus the direct ancestors of all 201 surviving kākāpō. Seven additional founders were not included in our dataset. We also obtained samples from 13 historical birds collected between 1847 and 1924 (Table S1) from the South Island of New Zealand hereafter referred to as the mainland population. Because kākāpō have a long generation time (∼15 years; see Methods S1), we can assume that the historical specimens correspond to the same temporal period.

### Method details

#### DNA extraction

For the *de-novo* assembly and modern samples, DNA was extracted using a phenol/chloroform extraction protocol^73^ (see Methods S1).

For historical samples, we extracted DNA from samples with high endogenous DNA content (i.e., 75.9%–91.4%)^3^ using a DNeasy Blood & Tissue Kit (QIAGEN, Hilden, Germany). Appropriate precautions were taken to minimize the risk of contamination in historical samples.^74^

#### Library preparation

For the *de-novo* assembly, 15kb and 30kb PacBio libraries were generated, and long-range scaffolding performed with Bionano optical mapping (see Methods S1).

For modern samples, double-stranded libraries were prepared according to New Zealand Genomics Limited (NZGL, Palmerston North, New Zealand) protocols for modern DNA and sequenced on an Illumina HiSeq2500 with a 2 × 150bp setup.

For historical samples, we prepared double-stranded Illumina libraries according to Meyer & Kircher.^34^ We used 20 μL of DNA extract in a 40 μL blunt-end repair reaction with the following final concentration: 1 × buffer Tango, 100 μM of each dNTP, 1 mM ATP, 25 U T4 polynucleotide kinase (Thermo Scientific) and 3U USER enzyme (New England Biolabs). To reduce biases caused by erroneous variant calls in historical genomes, we performed USER enzyme treatment to excise uracil residues resulting from post-mortem damage.^75^^,^^76^ Samples were incubated for 3 h at 37°C, followed by the addition of 1 μL T4 DNA polymerase (Thermo Scientific) and incubation at 25°C for 15 min and 12°C for 5 min. We then cleaned the samples using MinElute spin columns following the manufacturer’s protocol and eluted them in 20ul EB Buffer. Next, we performed an adaptor ligation step to ligate DNA fragments within each library to a combination of incomplete, partially double-stranded P5- and P7-adapters (10 μM each). This reaction was done in a 40 μL reaction volume using 20 μL of blunted DNA from the clean-up step and 1 μL P5-P7 adaptor mix per sample with a final concentration of 1 × T4 DNA ligase buffer, 5% PEG-4000, 5U T4 DNA ligase (Thermo Scientific). Samples were incubated for 30 minutes at room temperature and cleaned using MinElute spin columns as described above. Next, we performed an adaptor fill-in reaction in 40 μL final volume using 20 μL adaptor ligated DNA with a final concentration of 1 × Thermopol Reaction Buffer, 250 μM of each dNTP, 8U *Bst* Polymerase, Long Fragments. The libraries were incubated at 37°C for 20 minutes, heat-inactivated at 80°C for 20 minutes. These libraries were then used as stock for indexing PCR amplification.

In order to increase library complexity, we performed six indexing PCR amplifications for each library using different P7 indexing primers^34^ in 25 μL volumes with 3 μL of adaptor-ligated library as template, with the following final concentrations: 1x AccuPrime reaction mix, 0.3 μM IS4 amplification primer, 0.3 μM P7 indexing primer, 7 U AccuPrime Pfx (Thermo Scientific) and the following cycling protocol: 95°C for 2 min, 12 cycles at 95°C for 30 s, 55°C for 30 s and 72°C for 1 min and a final extension at 72°C for 5 minutes.

We used Agencourt AMPure XP beads (Beckman Coulter, Brea, CA, USA) for purification and size selection of libraries, first using 0.5X bead:DNA ratio and second 1.8X to remove long and short (i.e., adaptor dimers) fragments, respectively. We then measured library concentration with a high-sensitivity DNAchip on a Bioanalyzer 2100 (Agilent, Santa Clara, CA, USA). Finally, multiplexed libraries (i.e., six indexed libraries) were pooled in equimolar concentrations and sequenced on an Illumina HiSeqX with a 2 × 150bp setup in the High Output mode at the SciLifeLab sequencing facility in Stockholm.

### Quantification and statistical analysis

#### De-novo assembly and annotation

The kākāpō assembly was generated with the Vertebrate Genomes Project (VGP) v1.6 assembly pipeline^18^ using a combination of PacBio and Hi-C libraries (see Methods S1). The final assembly size was of 1.17 Gb, with a scaffold N50 of 83.2Mb and assigned to 26 chromosomes (24 autosomes and two sex chromosomes; see Methods S1). We identified Z and W chromosomes from the assembled genome by blasting all scaffolds against the Z-chromosome of zebra finch (v3.2.4, *Taeniopygia guttata*; GenBank: GCA_000151805.2) and W-chromosome of chicken (v5.0, *Gallus gallus*, GenBank: GCA_000002315.5) using BLAST+ 2.5.0.^35^ The BLAST+ parameters were set as: -evalue = 1e-10; -word_size = 15; -max_target_seqs = 1000. We excluded the identified Z chromosome (CM013763.1; 101.23Mb) and W chromosome (CM013773.1; 35.7Mb), from all downstream analyses in order to avoid bias associated with analyses relying on heterozygosity estimates. We also visually examined genome coverage estimated with Qualimap v2.2.1^36^ (see below) for males and females alignments to confirm the identity of the Z and W chromosomes. Males had on average ∼15X and ∼0X for the Z and W chromosome, respectively, and females had on average ∼7X and ∼7X for the Z and W chromosome, respectively. We identified CpG sites using a custom script masking CG sites^14^^,^^37^ and masked repetitive elements in the genome assembly using RepeatMasker v4.0.7^38^ applying the repeat element library of the *aves* database.

We annotated the assembly using the MESPA pipeline^39^ (see Methods S1). Briefly, we collapsed reference protein sets for zebra finch (*Taeniopygia guttata*; GenBank: GCA_000151805.2) to 90% coverage following Uniprot90 guidelines using a custom script to only retain sequences with at least 90% sequence identity to, and 80% overlap with, the longest sequence. We then generated an annotation in gff format and extracted 85% (13,175 out of 15,342) high quality kākāpō protein models using zebra finch as a reference protein set. We refined this annotation using the BRAKER2 v2.1.1 pipeline^40^, ^41^, ^42^ and used the resulting zebra finch proteome to predict kākāpō proteins with the exon-aware, protein-to-genome aligner SPALN2.^43^ We then extracted CDSs and protein sequences from this annotation with cufflinks v2.2.1^44^^,^^45^ gffread command using the -V option to exclude genes with in-frame STOP codons. We identified 16,171 kākāpō gene models with a mean length of 1,514bp (Median = 672; min = 50; max = 26,940) to be used in downstream analyses. Finally, we performed a functional annotation of these gene models using the eggNOG-mapper v4.5.1^46^ and obtained 15,699 annotated gene models (see Methods S1).

Two other annotations not used in downstream analyses were also generated using the Ensembl gene annotation system^77^ and NCBI Eukaryotic Genome Annotation Pipeline^78^ (see Methods S1).

#### Historical and modern data processing

All data processing and analyses were performed on resources provided by the Swedish National Infrastructure for Computing (SNIC) at UPPMAX, Uppsala University. Raw historical sequence data were demultiplexed using bcl2Fastq v2.17.1 with default settings (Illumina Inc.). We merged forward and reverse sequencing reads before mapping as recommended for damaged and short reads.^79^ We used SeqPrep v1.1^47^ to trim adapters and merge paired-end reads using default settings. We made a minor modification to the source code, which enabled to choose the best quality score of the overlapping bases in the merged region instead of aggregating the scores, following Palkopoulou et al.^12^ We mapped the merged reads against the reference genome using the BWA v0.7.13 aln algorithm^48^ with deactivated seeding (-l 16,500), allowing more substitutions (-n 0.01) and allowing up to two gaps (-o 2). We used the BWA samse command to generate alignments in SAM format and Samtools v1.3^49^ to convert these alignments to BAM format, sort and index them. Finally, we removed PCR duplicate reads using a custom python script that takes into account both start and end position of the reads.^12^ Even though all historical genomes were USER-treated^75^^,^^76^ during library preparation to remove post-mortem DNA damage, we used mapDamage v2.0^50^ on the 13 historical samples to estimate damage patterns (Figure S4).

For modern samples, we trimmed forward and reverse reads to remove Illumina adapters using Trimmomatic v0.32 with default settings^51^ and then mapped them to the reference genome using BWA mem v0.7.13.^48^ Samtools was used for sorting, indexing, and removing duplicates from the alignments.

Next, we processed historical and modern bam files using the same approach. We used Picard v1.141 to assign read group information including library, lane and sample identity to each bam file. We then re-aligned reads around indels using GATK IndelRealigner v3.4.0,^52^ and only kept reads with mapping quality mapQ ≥ 30 for subsequent analysis. For each genome, we estimated the depth of coverage using Qualimap v2.2.1.^36^ After this filtering, average genome coverage ranged from 11.8 and 18.2 (average = 15.3) and from 10.3 to 27.7 (average = 14.2) for modern and historical genomes, respectively (Table S1).

We called variants in historical and modern genomes separately for each individual using bcftools mpileup v1.3and bcftools call v1.3^49^^,^^53^ using a minimum depth of coverage (DP4) of 1/3X of the average coverage (i.e., 5X) and removed SNPs with base quality QV < 30 and those within 5bp of indels. We also filtered out SNPs in heterozygous state with an allelic balance (i.e., number of reads displaying the reference allele/depth) of < 0.2 and > 0.8 in order to avoid biases caused by contamination, mapping or sequencing error.

We removed the Z and W chromosomes, hard masked all identified CpG sites and repeat regions using BEDtools v2.27.1.^54^ After merging all 49 individual vcf. files we obtained 2,785,380 high quality SNP calls. We then used PLINK v1.9^55^ to filter variants not covered in all of the 49 individuals resulting in a total of 880,370 high quality SNPs that were used in all downstream analyses (i.e., population structure, demography, genome-wide diversity and inbreeding, mutational load estimation).

#### Population structure

We first used the R package SNPRelate to perform a principal component analysis (PCA) based on the genetic covariance matrix calculated from the genotypes^56^ using our filtered SNP dataset.

Second, we used the program ADMIXTURE v1.3.0^57^ to identify genetic clusters (K = 1-4) within our dataset. This program estimates ancestry in a model-based manner where individuals are considered unrelated and uses a cross-validation procedure to determine the best number of possible genetic groups present in the dataset.

Third, we constructed a phylogenetic tree using RapidNJ v2.3.2^58^ based on the neighbor-joining method.^80^ This method calculates the distance matrix of *D*_ij_ between each pair of individuals (i and j) with the following formula:

Dij=∑m=1MdijL

Where, *M* is the number of segregating sites in i and j, *L* is the length of regions, d_ij_ is the distance between individuals i and j at given site. d_ij_ = 0, when individuals i and j are both homozygous for the same allele (AA/AA); d_ij_ = 0.5, when at least one of the genotypes of an individual i or j is heterozygous (Aa/AA, AA/Aa or Aa/Aa); and d_ij_ = 1, when individuals i and j are both homozygous but for different alleles (AA/aa or aa/AA).

Since all three methods agreed in the main population structure within the specimens in our dataset and showed a clear distinction between the Stewart Island and the mainland population (Figure S5), we used the identified clusters for all downstream analyses. All mislabelled specimens (i.e., VM5, AUC2, LEI2, AUS1) were analyzed as part of the population they were genetically assigned to.

#### Demographic reconstruction and population divergence

We used the Pairwise Sequentially Markovian Coalescent (PSMC v0.6.5)^21^ model to estimate temporal changes in effective population sizes (*N*_e_) of kākāpō. We generated consensus sequences for all autosomes of a subset of historical and modern genomes using the Samtools mpileup v1.3^49^ command and the ‘vcf2fq’ command from vcfutils.pl. We filtered for base and mapping quality below 30, and depth below 1/3X of the average coverage for each specimen. We set N (the number of iterations) = 30, t (Tmax) = 15 and p (atomic time interval) = 64 (4+25∗2+4+6, for each of which parameters are estimated with 28 free interval parameters). To estimate the substitutions rate per site/year, we used TimeTree,^81^ which estimates the substitution rate based on automated literature searches. We aligned 135 birds genomes and assumed a divergence time of 25 my BP between the kea and kākāpō lineages.^82^ We obtained an estimate of 0.89 × 10^−9^ substitutions/site/year.

In order to scale population parameters, we assumed a generation time of 15 years making for a rate (μ) of 1.33 × 10^−8^ substitutions/site/generation which is biologically realistic in a large and natural kākāpō population (data not shown; see Methods S1).

Second, we reconstructed the population history of kākāpō and estimated divergence times between the Stewart Island (N = 35) and the mainland (N = 11; excluding TEP11, AUC2 and VM5 which formed their own cluster; Figures 1and S5) populations and their effective population sizes (*N*_e_) using a composite-likelihood method based on the site frequency spectrum^83^ implemented in fastsimcoal2 v2.6.^59^^,^^60^ We obtained a folded site frequency spectrum by converting the vcf file filtered for missing data (880,370 SNPs) into Arlequin format in PGDspider^61^ and then by converting the resulting Arlequin file into a joint Site Frequency Spectrum (joint 2D-SFS) in Arlequin v3.5.^62^ We also collapsed all SFS entries less than 5 in a single category (command line option –C5). We designed four competing scenarios including a post-glacial population size change (bottleneck or expansion) and a divergence event of the Stewart Island population from the Mainland: (a) *Post-glacial divergence*, (b) *Post-glacial divergence followed by Stewart Island population expansion*, (c) *Post-glacial divergence followed by Mainland population expansion* and (d) *Post-glacial divergence followed by Stewart Island and Mainland populations expansion* (Figure S6). The latter population size change was not constrained in the model in order to allow for either a bottleneck or population expansion to occur but is referred to as an expansion since it was supported by the simulations (Figure S8; Table S3). For each scenario, we carried out 50 replicate runs with the following settings: -n 100000 -m -q -M 0.001 -l 10 -L 40. Initial prior distributions followed a log-uniform distribution for population sizes (*N*_pre-glac_: 10^3^ –10^5^*; N*_Main_: 10^3^ –10^5^; and *N*_Stewart_: 10^2^ –10^4^; *N*_glac-main_: 10^3^ –10^5^; and *N*_glac-Stewart_: 10^2^ –10^4^), timing of glacial bottleneck (T_BOT_: 10^2^ – 10^5^), timing of divergence (T_DIV_: 5×10^2^ – 1.5×10^3^), timing of terminal expansion for both populations (T_EXP_: 5×10^2^ – 1.5×10^3^). The data was modeled as FREQ (1 bp simulated for each locus), with the number of independent chromosomes equal to the total number of loci (including monomorphic loci) characterized. We used the same substitution rate and generation time as mentioned above for the PSMC. We then used the range in parameter estimates across the initial 50 runs as the prior distribution for another 50 replicates within each scenario, until no further increase in likelihood was detected. The parameter values from the final run with the highest likelihood for each scenario were then used for 50 additional runs with –n = 1000000 to obtain a final estimate of the maximum observed likelihood. We assessed the best fitting scenario by Akaike’s information criterion (AIC) score^84^ and with the AIC’s weight (w), as described in Excoffier et al.^60^ (Table S2). We then used the parameter values from the best-fitting scenario to simulate 100 parametric bootstraps datasets. In order to obtain confidence intervals for parameter estimates, we used the ∗.tpl and initial prior distribution ∗.est files that led to the best replicate and ran 50 replicates per simulated dataset, making for a total of 5000 parameter estimates (Table S3). We changed the data type to DNA (1 bp), with the number of chromosomal segments equalling the total number of loci in the SFS (including monomorphic sites).

Third, we used the multiple sequential Markovian coalescent (MSMC2) model^63^ based on phased haplotypes from the two populations to infer changes in kākāpō *N*_e_. We used Beagle v5.1^64^ on default settings to phase the SNP-calls. Genome mappability masks and multi-sample input files were obtained using msmc-tools following the pipelines described in Schiffels and Wang.^63^ MSMC2 was then run using the five genomes with highest coverage for each population and using default settings. We used the same substitution rate (μ) and generation time as those described for the PSMC for scaling.

Finally, we estimated the split time (T), assuming no coalescent events since divergence between the mainland and Stewart Island using the PSMC approach applied to a pseudo-diploid Z chromosome genome as described in Palkopoulou et al.^12^ We extracted the Z-chromosomes from one mainland historical (CAN1) and one modern Stewart Island (Ruth) female. We generated a Z chromosome haploid consensus sequence for each these two females and merged them into a pseudo-diploid sequence using the seqtk mergefa command. We then applied the PSMC method on the pseudo-diploid Z chromosome to estimate changes in *N*_e_ over time. Finally, we rescaled the pseudo-diploid Z chromosome curve to 0.25 consistent with the effective population size of chromosome Z relative to that of autosomes (sex-chromosome/autosome ratio: 0.75). We ran the analysis using the same quality filters, parameters (i.e., 64 discrete time intervals) and the same substitution rate as above for the PSMC on autosomes. As a comparison, we also ran the analysis using fewer discrete intervals (i.e., 49 = 6+4+3+13∗2+4+6 or 37 = 2+2+1+15∗2+2) as recommended by Prado-Martinez et al.^85^ in order to avoid underestimation of the split time.

#### Genomic diversity and inbreeding

We first estimated genome-wide population-level nucleotide diversity (π)^86^ in mainland and Stewart Island birds with vcftools^65^ using a sliding window of 10kbp.

Second, we used mlRho v2.7^66^ to estimate the mutation rate (θ), which approximates the per site heterozygosity under the infinite sites model and uses bam files as input. We first filtered out bases and reads with quality below 30, and positions with root-mean-squared mapping quality below 30 from the historical and modern bam files. Because high or low coverage in some regions resulting from structural variation can create erroneous mapping to the reference genome and false heterozygous sites, for each specimen, we also filtered out sites with depth lower than five times and higher than two times the average coverage across all our specimens. We then estimated the individual θ as the number of heterozygote sites per 1,000bp. The maximum likelihood approach implemented in mlRho has been shown to provide unbiased estimates of average within-individual heterozygosity at high coverage.^66^^,^^87^

Third, we estimated individual inbreeding coefficients, by estimating the number and length of Runs of homozygosity (ROH). ROH are long tracts of the genome with very little or no heterozygote sites that can inform about recent and past population events and can be used to estimate individual inbreeding levels.^88^ We used PLINK v1.9^55^ to identify ROH and per sample inbreeding coefficients (F_ROH_). We first converted the filtered multi-individual vcf. file comprising 35 Stewart Island and 14 mainland individuals into a ped file and identified ROH in autosomal chromosomes. We used a sliding window size of 100 SNPs (*homozyg-window-snp 100*). We assumed a window to be homozygous if there were not more than 1 heterozygous site per window (*homozyg-window-het 1*). Moreover, if at least 5% of all windows that included a given SNP were defined as homozygous, the SNP was defined as being in a homozygous segment of a chromosome (*homozyg-window-threshold 0.05*). This threshold was chosen to ensure that the edges of a ROH are properly delimited. Furthermore, a homozygous segment was defined as a ROH if all of the following conditions were met: the segment included ≥ 25 SNPs (*homozyg-snp 25*) and covered ≥ 100kb (*homozyg-kb* 100); the minimum SNP density was one SNP per 50kb (*homozyg-density 50*); the maximum distance between two neighboring SNPs was ≤ 1,000kb (*homozyg-gap 1,000*). For the number of heterozygous sites within ROH, we set the value at 750 (*homozyg-het 750*) in order to prevent sequencing errors to cut ROH. Based on these results, we estimated the inbreeding coefficient F_ROH_ estimated as the overall proportion of the genome contained in ROH.

While we were mainly interested in estimating the relative difference between mainland and Stewart Island birds, we also assessed the robustness of our results to the various parameters used and to potential sequencing errors, by running the same analysis using more stringent parameters. Specifically, we varied the number of heterozygous sites per ROH segment (*homozyg-het 1*), at least one SNP in a ROH per 100kb (*homozyg-density* 100) and the maximum distance between two neighboring SNPs (*homozyg-gap* 500).

We statistically compared heterozygosity, F_ROH_ between mainland and Stewart Island kākāpō using a Welch’s two-sample t tests in R.^89^

#### Mutational load estimation

We estimated mutational load in mainland and Stewart Island kākāpō genomes using two approaches. First, we measured the relative mutational load in each individual as the number of derived alleles at sites that are under strict evolutionary constraints (i.e., highly conserved) and thus likely to be deleterious using genomic evolutionary rate profiling scores (GERP) with the GERP++ software^67^ and following van der Valk et al.^90^ We included both heterozygous (counted as one allele) and homozygous positions (counted as two alleles) even though the mutational effect of heterozygous positions depends on additional assumptions about the dominance coefficient. GERP identifies constrained elements in multiple alignments by quantifying the amount of substitution deficits (e.g., substitutions that would have occurred if the element were neutral DNA, but did not occur because the element has been under functional constraint) by accounting for phylogenetic divergence. High GERP scores (> 1) represent highly conserved regions whereas low scores (< 1) are putatively neutral.

To identify genomic regions under strong evolutionary constraint in the kākāpō we obtained 135 published bird genomes from NCBI (Figure S16). We used TimeTree^81^ to estimate the divergence times among these genomes as described above. Each of these genomes were then converted into fastq-format (50 bp reads) and realigned against the kākāpō assembly using BWA mem v0.7.13,^48^ slightly lowering mismatch and gaps penalty scores (-B 3, -O 4,4). Additionally, we filtered out all reads from the processed bam files aligning to more than one genomic location using Samtools.^49^ Next, we converted each alignment file to fasta-format using htsbox v1.0 -R -q 30 -Q 30 -l 35 -s 1. GERP++ was then used to calculate conservation scores for each site in the genome for which at least three bird species could be accurately aligned to the kākāpō reference (Figure S17). The kea genome (*N. notabilis*) alignment was used for the ancestral allele inference.^91^^,^^92^

To estimate the mutational load of each individual we obtained the total number of derived alleles stratified by GERP-score within highly conserved regions of the kākāpō genome (excluding sites with missing genotypes). The individual relative mutational load was then calculated as the sum of the number of all derived alleles above GERP-score of two (as these are considered to be deleterious) multiplied by their GERP-score, divided by the total number of derived alleles by individual (including those below a GERP-score of one). Higher values indicate that a relatively larger proportion of derived alleles is found at conserved genomic sites, thus indicating higher mutational load. We statistically compared GERP-scores between mainland and Stewart Island kākāpō using a Welch’s two-sample t tests in R.^89^

Second, we estimated mutational load in coding regions for mainland and Stewart Island kākāpō genomes using SNPeff v4.3.^68^ We used our dataset filtered for missing genotypes (880,370 SNPs) to avoid any bias due to sequencing stochasticity when estimating the difference in mutational load between populations and the annotation of 15,699 genes from the MESPA pipeline (see Methods S1) for this analysis. In order to avoid a reference bias when identifying synonymous and non-synonymous variants, we replaced the reference allele with the ancestral allele by using kea (*N. notabilis*) as reference and using a custom script as described above. After replacing the reference allele, we obtained a total of 406,510 SNPs.

We generated a database for kākāpō using the protein sequences extracted from our annotation. We further removed any gene model with in-frame STOP codons using the -V option of gffread from the cufflinks v2.2.1^44^^,^^45^ package. We first identified putative deleterious variants in four different impact categories as defined in the SNPeff manual: a) *Low*: mostly harmless or unlikely to change protein behavior (i.e., synonymous variants); b) *Moderate*: non-disruptive variants that might change protein effectiveness (i.e., missense variants; Table S4); c) *High*: variant assumed to have high (disruptive) impact in the protein, probably causing protein truncation, loss of function (LoF) or triggering nonsense mediated decay (i.e., stop codons, splice donor variant and splice acceptor, start codon lost; Table S5); d) *Modifier*: usually non-coding variants or variants affecting non-coding genes, where predictions are difficult or there is no evidence of impact (i.e., downstream or upstream variants).^68^ Next, we identified the number of variants in these four categories separated by homozygous and heterozygous state. Because we only used sites covered in all individuals, we counted the number of variants in these four categories separated by homozygous and heterozygous state and did not need to use bootstrapping of allele counts. We then compared the number of each of these variants in mainland and Stewart Island kākāpō using a Welch’s two-sample t tests in R.^89^

We then estimated the difference in frequency of variants of all impact categories listed above between mainland and Stewart Island kākāpō using a similar approach to the one described by Xue et al.^25^ and van der Valk et al.^10^ For each category of variants, we calculated at each site *i* the observed allele frequency in Population x as f^x^_*i*_ = d^x^_*i*_ / n^x^_*i*,_ where n^x^_i,_ is the total number of alleles called in population x and d^x^_i_ is the total number of called derived alleles. Similarly, we define f^y^_*i*_ for population y. For each C category of variants we estimated:

$${\text{Freq}}_{\text{pop}-\text{x}}\left(C\right)=\sum_{i\;\in \;C}{\text{f}}_{i}^{x}\left(1-{\text{f}}_{i}^{y}\right)$$

We then calculated the *R*_xy_ = Freq_pop-x_ / Freq_pop-y_ ratio, where a value of 1 corresponds to no change in frequency, > 1 a decrease in frequency in population y relative to population x and < 1 to an increase in frequency in population y. relative to population x We estimated the variance in the *R*_xy_ ratio by running a Jackknife approach in blocks of 1000 from the set of sites in each category of mutation. The R_xy_ ratio only included sites where at least one out of all alleles is derived in both populations.

To check for annotation bias, we performed the same analysis using a consensus mainland historical genome. We modified our modern high quality genome by changing SNPs and indels to the historical state using the genome polishing software Pilon v.1.22^69^ with quality filtering (–minmq 20 –minqual 20) and by mapping merged reads from individual LEI2, which had the highest coverage of the historical genomes (Table S1) using BWA mem v0.7.13.^48^ A second annotation for the historical genome was generated with the MESPA pipeline (see Methods S1), by using the historical genome as the reference with all other steps identical. The raw data was then mapped to this consensus and the variant calling performed as described above. After filtering for missing genotypes, we obtained 834,420 SNPs. Finally, we also replaced the reference allele with the ancestral allele by using kea (*N. notabilis*) in order to avoid reference bias as described above obtained a total of 371,886 SNPs. Results were consistent with those based on data mapped to the modern assembly (Figure S21).

Purging of recessive deleterious variants (i.e., LoF alleles) is expected to lead to different signatures in homozygous (i.e., runs of homozygosity; ROH) and non-homozygous tracts within individuals. Since the individuals in this study were adults when sampled, recessive LoF variants with a deleterious effect on viability or survival in early infancy should thus be less common in homozygous tracts, where they have been exposed to purifying selection, than elsewhere in the genome. To test this hypothesis, we measured the number of LoF variants sites in homozygous and heterozygous portions of the genome and controlled for differing amounts of homozygosity within individuals by normalizing the rates of LoF variant sites by the rates of synonymous homozygous variant sites in the same regions obtained from the SNPeff output. We then assessed significance of the difference between relative rates of LoF variants in the homozygous and non-homozygous portions of the genomes using a paired t test in R.^89^

#### Gene Ontology

We performed a functional analysis for genes with LoF variants identified in SNPeff and based on Jane’s annotation (Table S6). We obtained the gene IDs associated with each LoF allele identified in the SNPeff analysis from our functional annotation. We then assessed the functional classification of these LoF variants with a Gene Ontology analysis in Panther v16.0^70^ using chicken as reference set. Because identifying mutational load in birds that survived the peak of the 1990s bottleneck is highly valuable to guide future conservation actions for kākāpō, we performed this analysis only on survivor birds (i.e., 35 Stewart Island birds and Richard Henry; Tables S6 and S7).

#### Forward simulations

Since the effect of drift and purifying selection are dependent on *N*_e_^8^, we estimated changes in mutational load under contrasting demographic scenarios to assess their respective roles in declining populations. To test whether our results were consistent with purging of deleterious mutations in the Stewart Island population, we performed forward simulations recapitulating the demographic history of mainland and Stewart Island kākāpō. We also simulated scenarios for hypothetical stable and severely bottlenecked populations to model a weak and strong effect of drift, respectively.

We performed individual-based simulations with SLiM 3^71^ using the non-Wright-Fisher (non-WF) implementation. As opposed to Wright-Fisher models, which operates under a more restrictive set of assumptions, non-WF models are fully customizable in terms of mate choice, reproduction, survival and population regulation, which allowed us to approximate the kākāpō life-history traits in a more realistic way based on Powlesland et al.^4^ We controlled the sex ratio to simulate the observed skewed sex ratio of kākāpō in the wild (∼2:1 in favor of males). We controlled time to sexual maturity by only allowing individuals to reproduce after females reached sexual maturity between 7 and 11 years old and males slightly sooner, between 5 and 7 years old. We simulated the known variance in reproductive success by allowing more experienced males (i.e., older males) to form pairs more readily as a function of their age. Pairs produced clutches in accordance with clutch-sizes observed in the wild, using random draws from a normal distribution (mean = 3, sd = 1.5) each pair produced between two and four individuals, and rarely more than four and less than two (including zero to represent inviable eggs). This mating scheme revealed that in our simulations, approximately a third of the individuals produced all the offspring in a given generation. Therefore, we simulated 2.8 times more individuals than our target effective population size. In non-WF simulations generations are overlapping (as in nature) and the average generation time is an emerging property of the simulation in function of the life-history parameters used. We recorded the full genealogy of 500 simulations steps and estimated that in average the distance between parents and offspring nodes was of ∼16 (sd = 2) simulation steps. This is remarkably consistent with the estimated generation time for kākāpō, estimated around 15 years. Thus, each simulation step can be thought as one year (the total simulation time was 25,000 years) and the generation time in our simulation to be in average 16.5 years.

We simulated 3,291 genes across the 23 fully assembled chromosome in relative proportions and positions as observed in the genome assembly, representing 20% of the total kākāpō exome. Each in-silico gene had a length of 1.5kb adding to a total amount of 4,936,500 base pairs simulated for each individual. We used a per-base, per-generation mutation rate of 1.33x10^−08^. A recombination rate of 1x10^−9^ was used between genes, but no recombination was allowed within genes. Neutral and deleterious mutations occurred at a relative proportion of 1:2.31.^72^ Selection coefficients of deleterious mutations were drawn from a gamma distribution (mean = −0.024, sd = 0.14), and simulations were performed independently for fully recessive (h = 0), partially recessive (h = 0.25) or additive (h = 0.5) dominance coefficients.

We simulated four distinct scenarios that spanned 25,000 years and that modeled distinct population trajectories since the LGM with *N*_e_ estimates from the PSMC used as priors: (i) a *Stewart Island* scenario modeled a decline to a long-term *N*_e_ of ∼1,000; (ii) a *Mainland* scenario modeled a decline to a long-term *N*_e_ of ∼6,000; (iii) an *Extreme* scenario modeled a sustained LGM decline to a long-term *N*_e_ of ∼100 to specifically simulate a strong effect of drift; (iv) a *Stable* scenario modeled a constant *N*_e_ of ∼10,000 and was used as a control, where the effect of drift should be weak.

We first performed a burn-in simulation step to obtain a fully coalesced population. Since our initial population size of N ∼28,000 with overlapping generations could take a very long time to reach coalescence, we sped-up this stage of the simulation by scaling-down population size and scaling-up recombination/mutation rates and selection coefficients by a factor of 10 as recommended in the SLiM 3 manual. We ran the burn-in simulation for 100,000 steps and collected the entire genealogy by the means of tree-sequence recording^93^ to confirm the tree had a single root with pyslim (i.e., has reached full-coalescence^94^). We then loaded the tree-sequence to start a new simulation where the scaling factors were removed. We first ran 10,000 generations and kept track of the trend of nucleotide diversity to confirm the scaling change had not disrupted the mutation-selection equilibrium (Figure S27). After 10,000 steps we varied the carrying capacity of the simulation to follow the different trajectories of our demographic scenarios for 25,000 steps (Figure 4) . We randomly sub-sampled 200 individuals from the last simulation step to compare the same sampling effort across all scenarios and models. We counted derived mutations for mutation classes of weakly deleterious (−0.001 ≤ s < 0), mildly deleterious (−0.01 ≤ s < −0.001) and strongly deleterious (s < −0.01) selection coefficients. We calculated additive genetic load as in Pedersen et al.^95^ by adding the sum of selection coefficients for homozygous mutations and the sum of selection coefficients times the dominance coefficients for heterozygous mutations.

## Data Availability

The genome assembly can be accessed at the NCBI database under BioProject: PRJNA510145. Assembly accesion numbers are Genbank: GCF_004027225.2 and GCA_004011185.1. Historical resequencing data can be accessed at the European Nucleotide Archive under project ENA: PRJEB35522. Modern resequencing data https://repo.data.nesi.org.nz from the ongoing Kākāpo 125+ project is maintained at the Genomics Aotearoa data repository (direct link to the Kākāpo 125+ genome sequencing dataset at https://repo.data.nesi.org.nz/TAONGA-KAKAPO). In this study, we used 36 genomes from a dataset currently consisting of 145 whole-genome sequences of kākāpo (raw and untrimmed fastq files). This dataset is made available with controlled access, managed via a data access committee of the New Zealand Department of Conservation (DOC) and Te Rūnanga o Ngāi Tahu. The kākāpo samples were obtained under an agreement that the genomic data is shared in accordance with principles of indigenous data sovereignty and that Te Rūnanga o Ngāi Tahu maintain Kaitiakitanga (i.e., governance and responsibility) over the data. The terms of the controlled access and management follow this agreement. To request access, users need to submit an application from the Genomics Aotearoa repository. This application needs to be made on the form provided at the Genomics Aotearoa repository or directly at the Kākāpo 125+ webpage (https://www.doc.govt.nz/our-work/kakapo-recovery/what-we-do/research-for-the-future/kakapo125-gene-sequencing/request-kakapo125-data/; a copy of this application form is provided in Figure S28). The application will require applicants to provide details of their proposed research project, including names of researchers and collaborators, if phenotypic data about Kākāpo traits is required from DOC, what engagement with Māori has been undertaken (e.g., discussions with Te Rūnanga o Ngāi Tahu about the proposed project, involvement of Māori researchers, benefit sharing with Māori), what considerations have been made for Mātauranga Māori (Māori knowledge; e.g., is Mātauranga Māori part of the project, will results of the project be fed back to Māori, are there intellectual property concerns that could affect Mātauranga Māori?). The application will also require a project summary that details the planned research studies, and which includes a section that details how this research will benefit kākāpo conservation. Direct benefit to kākāpō conservation is preferred, but is not a requirement for acceptance. Applications will be regularly assessed by DOC and Te Rūnanga o Ngāi Tahu. The default approach is to approve applications, as long as the applications are complete. Applications for basic research and to replicate previously published analyses are highly likely to be accepted. It is possible that an application will be rejected if there are significant concerns raised by DOC or Te Rūnanga o Ngāi Tahu. Concerns raised might include commercial use of the data (e.g., data shared and/or used by a for-profit organization such as drug or other companies), the applicant having a track-record of unethical behavior, and loss of the ability to exercise Kaitiakitanga (i.e., governance and responsibility) over the data, among others.

## Supplemental information

Document S1. Methods S1, Figures S1–S28, and Tables S2 and S3

Table S1. Sample list with reads and coverage statistics for 14 mainland and 35 Stewart Island kākāpō genomes used in this study, related to STAR Methods Asterisks indicate museum samples likely to have been mislabeled.

Table S4. Missense variants showing allele frequency difference between the mainland and Stewart Island using the dataset filtered for missing data, related to STAR Methods

Table S5. LoF variants showing allele frequency difference between the mainland and Stewart Island kākāpō using the dataset filtered for missing data, related to STAR Methods

Table S6. LoF variants showing allele frequency difference between Richard Henry and Stewart Island kākāpō, related to STAR Methods

Table S7. Functional analysis for genes with LoF variants showing allele frequency difference between the Richard Henry and Stewart Island kākāpō, related to STAR Methods

Document S2. Article plus supplemental information
