# Supplementary material for: Population genomics of the critically endangered kākāpō
Source: Cell Genom. 2021 Sep 8;1(1):100002. doi: 10.1016/j.xgen.2021.100002 (PMC9903828; doi:10.1016/j.xgen.2021.100002)
Supplement: Document S1. Methods S1, Figures S1–S28, and Tables S2 and S3 [file mmc1.pdf]

Cell Genomics, Volume *1*

Supplemental information

Population genomics

# of the critically endangered kākāpō

Nicolas Dussex, Tom van der Valk, Hernán E. Morales, Christopher W. Wheat, David Díez-del-Molino, Johanna von Seth, Yasmin Foster, Verena E. Kutschera, Katerina Guschanski, Arang Rhie, Adam M. Phillippy, Jonas Korlach, Kerstin Howe, William Chow, Sarah Pelan, Joanna D. Mendes Damas, Harris A. Lewin, Alex R. Hastie, Giulio Formenti, Olivier Fedrigo, Joseph Guhlin, Thomas W.R. Harrop, Marissa F. Le Lec, Peter K. Dearden, Leanne Haggerty, Fergal J. Martin, Vamsi Kodali, Françoise Thibaud-Nissen, David Iorns, Michael Knapp, Neil J. Gemmell, Fiona Robertson, Ron Moorhouse, Andrew Digby, Daryl Eason, Deidre Vercoe, Jason Howard, Erich D. Jarvis, Bruce C. Robertson, and Love Dalén

Supplemental Information

Population genomics reveals the impact of long-term small population size in the critically endangered kākāpō

*Contact lead: nicolas.dussex@gmail.com; ejarvis@mail.rockefeller.edu; bruce.robertson@otago.ac.nz; love.dalen@nrm.se

Contents

Methods S1

1.1 DNA extraction

1.2 Library preparation for *De-novo* assembly

1.3 *De-novo* assembly generation and curation

1.4 Genome annotations

1.5 Generation time for demographic reconstructions

Figures S1-S27

Methods S1

1.1 DNA extraction

For all modern samples, DNA extractions were performed using phenol-chloroform[1], including for the PacBio and 10X genomics libraries for the *de-novo* assembly. DNA isolated for generating Bionano libraries was mixed and centrifuged at 2,200 rcf for 5 minutes at 4°C. The resulting pellet was re-suspended in 100uL of buffer containing 10 mM Tris (pH 7.2), 50 mM EDTA, and 2 mM NaCl. The cell suspension was embedded into four 0.8% agarose plugs, targeting a 1:1, 1:2, 1:4, and 1:8 titration. Agarose plugs were then treated with Puregene proteinase K and RNAse A, washed, treated with agarase, and drop dialyzed, as described by the Bionano Prep Blood DNA Isolation Protocol (Document number 30033).

For historical samples, we extracted DNA from the toepads of the 13 historical birds (Table S1) using a DNeasy Blood & Tissue Kit (Qiagen, Hilden, Germany). These samples were selected based on high endogenous DNA content (i.e., 75.9-91.4%; Table S1) which was estimated with a

screening sequencing step where sequencing reads were mapped to the kākāpō mitogenome as described in[2]. Appropriate precautions were taken to minimize the risk of contamination in historical samples[3].

1.2 Library preparation for *De-novo* assembly

For the PacBio sequencing, Jane's genomic DNA was sheared using the Megaruptor (Diagenode, Denville, NJ), followed by SMRTbell library preparation according to the manufacturer protocols (Pacific Biosciences, Menlo Park, CA). Two libraries were generated with size selection (Sage Science, Beverly, MA) cut offs of 15kb and 30kb, resulting in libraries with average insert sizes of 40kb and 43kb, respectively. Sequencing was performed in 2016 on the PacBio RSII using P6-C4 sequencing chemistry and 10-hour acquisition times per run. A total of 127 SMRT Cells were run, resulting in ~90 Gb of total sequence data, with an insert size N50 of 13kb.

For Bionano optical mapping using the genomic DNA was fluorescently labeled with the enzymes DLE-1. The labeled DNA was run on a Saphyr instrument according to the Bionano Prep Direct Label and Stain (DLS) (document number 30206) and Bionano Prep Labeling NLRS (document number 30024) protocols.

1.3 *De-novo* assembly generation and curation

The kākāpō assembly was generated with the Vertebrate Genomes Project (VGP) v1.6 assembly pipeline[4]. Contigs were generated from PacBio subreads using FALCON-Unzip with 1 round of Arrow polishing using the DNAnexus FALCON 5.1.1 and FALCON-Unzip 1.0.2 pipelines[5]. This resulted in an initial set of primary contigs, which represent a pseudohaplotype, and a secondary set of haplotigs containing the alternative form of heterozygous alleles. The primary contig set was then iteratively scaffolded with 10X Genomics linked reads using scaff10x 2.0 (git 4.28.2018; https://github.com/wtsi-hpag/Scaff10X). Bionano optical maps were generated into in-silico cmaps using Bionano Solve v3.2.1[6] using non-haplotype aware arguments. One-enzyme scaffolding was performed with default parameters Hi-C libraries were made using the Arima-HiC Kit (P/N: A510008). Existing contigs and scaffolds were arranged into chromosome-level scaffolds using the Illumina paired end reads with Salsa 2.0[7] mapped with Arima mapping pipeline (https://github.com/ArimaGenomics/mapping_pipeline; Fig. S1).

Additional rounds of consensus polishing were conducted by mapping all the PacBio subreads and 10X reads to both the primary and alternative sets. One more round of Arrow polishing was conducted with PacBio reads using smrtanalysis v5.1.0.26412. For final polishing, the 10X linked reads were aligned with Longranger v2.2.2[8], and erroneous bases (variants) were called with Freebayes v1.2.0[9] before correction with bcftools v1.8[10]. During curation, assignment of alternative alleles in the primary assembly was further evaluated by Purge Haplotigs bitbucket 7.10.2018 to reduce artefactual duplication[11]. All scripts used in the assembly pipeline are publicly available from https://github.com/VGP/vgp-assembly.

The resulting kākāpō assembly was subjected to contamination screening and manual curation to detect and correct remaining assembly issues. The contamination screen detected and removed 34 scaffolds that consisted of *E. coli* sequence only (2,243kb total). For manual assembly curation, all available sequencing and mapping and previous assembly data were compared to the assembly using gEVAL[12]. Alignment discordances were manually assessed and resolved by breaking and re-joining scaffolds. A HiC 2D map generated with Juicer and visualized in Juicebox[13] allowed for further scaffold correction and super-scaffolding in order to bring the assembly to chromosome scale (Fig. S1). HiC-led changes were verified against Pacbio, Bionano, 10X link reads and other data in gEVAL.

The manual curation resulted in 19 scaffold rearrangements and the removal of 99 sequence regions that were identified as haplotypic duplications. This reduced the initial assembly length by 14.5Mb (1.2%) and the scaffold number from 229 to 99, whilst decreasing the scaffold NG50 from 88.2Mb to 83.2Mb to correct over-scaffolding. NG50 contig was of 9.1Mb. 99.2% of the final total assembly sequence of 1.17 Gb was assigned to the identified 26 chromosomes (24 autosomes and two sex chromosomes, see below), which were named according to their size (Figs. S2-3). Only 67 scaffolds remain unplaced.

We then identified Z and W chromosomes from the assembled genome by blasting all scaffolds against the Z-chromosome of zebra finch (v3.2.4, *Taeniopygia guttata*; GenBank: GCA_000151805.2) and W-chromosome of chicken (v5.0, *Gallus gallus*, GenBank: GCA_000002315.5) using BLAST+ 2.5.0[14]. The BLAST+ parameters were set as: -evalue = 1e-10; -word_size = 15; -max_target_seqs = 1000. We then excluded the identified Z chromosome (CM013763.1; 101.23Mb) and W chromosome (CM013773.1; 35.7Mb), from all downstream analyses in order to avoid bias associated with analyses relying on heterozygosity estimates. We

also visually examined the coverage across the genome in the bam files generated for males and females to check that the identified chromosomes were in fact the Z and W using Qualimap v2.2.1[15]. Males had on average ~15X and ~0X for the Z and W chromosome, respectively; and females had on average ~7X and ~7X for the Z and W chromosome, respectively. We then identified CpG sites using a custom script masking CG sites and masked repetitive elements in the genome assembly using RepeatMasker v4.0.7[16] (http://repeatmasker.org) applying the repeat element library of the *aves* database.

We also examined synteny between the *de-novo* kākāpō assembly and both zebra finch (v 3.2.4, *Taeniopygia guttata*; GenBank: GCA_000151805.2) and chicken (v5.0, *Gallus gallus*, GenBank: GCA_000002315.5) assemblies. The alignments were done with Mashmap2[17] at 150Kb resolution and 75% identity (Figs. S2-3).

The mitogenome was assembled using a dedicated mitoVGP v1.0 pipeline complementing both PacBio long-reads and 10x linked-reads using the same WGS data employed for the nuclear genome. Briefly, mitochondrial reads were identified in WGS long reads by similarity with a previously reported mitogenome assembly of the kākāpō (NC_005931.1) using BlasR v5.3.2[18]. Reads were assembled with Canu v1.8, the assembly polished with long reads using variantCaller v2.2.2 (Arrow), and further polished with short reads using Bowtie2 v2.1.0[19] and Freebayes v1.2.0[9]. Overlapping ends were trimmed using a custom script (https://github.com/GiulioF1/mitoVGP/tree/master/pipeline_v1.0), a second round of short-read polishing was performed, and the sequence was further trimmed with the same script. The final consensus sequence was manually oriented to start with trnF using annotation from MITOS2 (PMID: 22982435).

The final resulting assembled genome was submitted to the GenBank archive in two parts: the primary pseudohaplotype containing the best representation of the haploid genome (GCA_004027225.1/GCF_004027225.1), and the alternate pseudohaplotype containing all the alternative alleles (GCA_004011185.1).

1.4 Genome annotations

Three types of annotations were built for the kākāpō *de-novo* genome assembly. These annotations generated between 15,699 and 16,060 high-quality protein-coding gene models.

First, because RNA data was not available at the time of the initial analyses, we annotated the kākāpō *de-novo* genome assembly using publicly available protein sequences datasets and used this annotation for the analysis of mutational load in coding and non-coding regions. We first assessed the quality of the annotation using different reference protein sets with the MESPA pipeline[20]. We collapsed reference protein sets for the kea (*Nestor notabilis*; GenBank: SRP029311), which is closely related to kākāpō and zebra finch (*Taeniopygia guttata*; GenBank: GCA_000151805.2) to 90% coverage following Uniprot90 guidelines using a custom script. This results in each protein cluster being composed of sequences with at least 90% sequence identity to, and 80% overlap with, the longest sequence. In that way, we discarded isoforms of the reference datasets. We then used MESPA to extract the gene models in kākāpō with at least 90% length coverage to each set of reference proteins and to generate an annotation in gff format. We extracted 85% (13,175 out of 15,342) high quality kākāpō protein models (i.e., aligning to 90% of their expected length) using zebra finch as a reference protein set, and 86% (10,159 out of 11,806) using kea as a reference protein set.

Next, we refined the annotation for the kākāpō assembly using the BRAKER2 v2.1.1 pipeline[21–23]. BRAKER is an automated method for accurate gene structure annotation, which allows fully automated training of the gene prediction tools GeneMark-EX[24,25] and AUGUSTUS[26–28] for gene model prediction and can incorporate protein homology information from closely related species for training purposes.

In order to generate a high quality protein dataset for training in BRAKER2, we predicted proteins for kākāpō using the zebra finch proteome[29] (ftp://ftp.ensembl.org/pub/release-89/fasta/taeniopygia_guttata/pep/Taeniopygia_guttata.taeGut3.2.4.pep.all.fa.gz), then filtered these for high quality models by comparing them to the flycatcher proteome[30] (ftp://ftp.ensembl.org/pub/release-89/fasta/ficedula_albicollis/pep/Ficedula_albicollis.FicAlb_1.4.pep.all.fa.gz).

The resulting proteins after filtering, were considered high quality kākāpō protein sequences, which were then used as training for gene model predictions by BRAKER2. Both the zebra finch and flycatcher proteomes were first clustered to the longest representative of each set of proteins having at least 90% amino acid identity, using cd-hit v4.6.1 (-c 0.90 -T 16 -aS 0.8 -M 0), reducing the protein dataset from 18,204 to 15,363 and 15,983 to 15,372 proteins, for zebra finch and flycatcher, respectively, in order to remove recent duplicates and isoforms. Using the

zebra finch clustered proteome as input, along with the kākāpō genome (ftp://ftp.ncbi.nlm.nih.gov/genomes/all/GCA/004/027/225/GCA_004027225.1_bStrHab1_v1.p/GCA_004027225.1_bStrHab1_v1.p_genomic.fna.gz), proteins were predicted with the exon aware, protein to genome aligner SPALN2[31].

The resulting kākāpō proteins where then filtered to only retain those with a start codon and no internal stop codons (n=8,784). These were then further filtered to only retain those that when aligned to the flycatcher filtered proteome, covered at least 95% of the flycatcher protein length (n=6,657), and then this filtered kākāpō protein set was then used as a high quality protein set for BRAKER2 training.

Soft masking of the repeat content in the genome was performed using Red[32], which masked 32.33% of the genome (375,531,262 bp). Alignment of the filtered kākāpō protein dataset against the soft masked genome was performed by GENOME THREADER v1.7.0, with the option "-species "chicken" set in order to improve splice-site recognition[33]. The output of the GENOME THREADER alignment was then used by BRAKER2 for gene prediction training (settings: --prg=gth --gth2traingenes --trainFromGth –softmasking).

We then extracted CDSs and protein sequences from this annotation using cufflinks v2.2.1[34,35] gffread command using the -V option to exclude genes with in-frame STOP codons. We identified 16,171 kākāpō gene models with a mean length of 1,514bp (Median=672; min=50; max=26,940) to be used in downstream analyses.

Finally, we performed a functional annotation of these gene models using the eggNOG-mapper v4.5.1[36], which uses fast orthology assignments upon precomputed protein clusters within a phylogenetic context[36]. We used '*Aves*' as taxonomic scope and the 'Restrict to one-to-one' and the 'Use experimental terms only' to prioritize precision, quality of matches and also to remove multiple matches which could represent pseudogenes. Overall, we obtained 15,699 annotated gene models.

Secondly, the Ensembl gene annotation system[37] was used to annotate the kākāpō *de-novo* genome assembly. Multiple lines of evidence were used: MinION kidney and brain long reads (SRX6605068, SRX6605069), Pacbio Iso-Seq liver long reads (SRX5842929), Illumina kidney and brain short reads (SRX6605066, SRX6605067), splice aware protein-to-genome alignments of vertebrate proteins classed as either protein existence level 1 (experimental evidence at the protein level) or level 2 (experimental evidence at the transcript level) from UniProt[38] and

coordinate mapping of human reference annotations to kākāpō via a pairwise whole genome alignment.

Long read data were mapped to the genome using Minimap2[39] (PMID: 29750242) with the recommended settings for Iso-Seq and Nanopore data. Short read data were initially mapped via BWA[40] and then locally re-aligned in a splice-aware manner via Exonerate[41]. Protein-to-genome alignments were carried out via GenBlastG[42]. Annotation mapping from human was carried out via a pairwise alignment using LastZ[43] and subsequent exon coordinate mapping and transcript reconstruction via both in-house software and CESAR v2.0[44].

Due to the high error rate of the Nanopore data, post mapping error correction was employed to maximize the number of usable mappings. Intron/exon boundaries that were non-canonical or deemed low frequency (five or fewer observations across all mappings at a locus) were replaced with high frequency boundary coordinates (greater than five observations) within a 50bp edit distance. High frequency boundary observations were determined both from canonical boundary observations from the Nanopore mapping themselves and also from the alignments of the short-read data. A similar strategy was employed to remove likely artificial gaps of 200bp or less from exons described by the Nanopore data. In these cases, low frequency potential gaps between two adjoining exons were filled in based on high frequency observations of single exons with the same terminal boundary coordinates.

In order to determine the protein coding genes and transcripts, all evidence lines were analysed at each locus. ORF likelihood was determined by aligning the ORF translation against known vertebrate proteins. Preference was given to transcript isoforms generated from the transcriptomic data that had high coverage matches to known vertebrate proteins. For loci where the transcriptomic data was not available or highly fragmented, gap filling was done using the splice-aware protein-to-genome alignments and annotation mappings from human via a pairwise whole genome alignment, with preference given to resulting ORFs that showed a high percent coverage and identity when re-aligned to the original evidence. A total of 16,037 protein-coding genes were identified with 24,520 transcript isoforms.

Pseudogenes called from the protein-coding loci that did not have transcriptional support, were analysed for evidence of structural abnormalities, such as absence of a start coding, non-canonical splicing, unusually small intron structures (< 75bp) or excessive repeat coverage. Loci with two or more such abnormalities were reclassified as pseudogenes. Single exon loci that

showed matched a multi-exon ORF elsewhere in the genome with greater than 80 percent coverage were reclassified as retrotransposed pseudogenes. At total of 103 pseudogenes and 9 retrotransposed pseudogenes were identified.

Long non-coding loci were called using both the short and long read transcriptomic data. Potential lncRNAs were initially called from transcript models where no BLAST[28] hit to a known vertebrate protein was found. The resulting set was then filtered to remove and transcripts with genomic overlap with a protein-coding locus. An addition filter was then applied to remove single exon loci (due to the abundance of transcriptional noise generally found in long read data). We identified 3,578 long non-coding loci with 4,030 transcript isoforms.

Small non-coding loci were predicted using data from miRbase[45] and Rfam[46] and scanning against the genome (described in more detail in the Ensembl annotation system[37]). Initial hits were then filtered based on predicted ability to form stem-loop secondary structures. This resulted in 465 small non-coding gene predictions. The kākāpō gene annotation is due to be released in Ensembl release 99 (expected December 2019). For more detail on the annotation system please refer to the Ensembl annotation system[37].

Finally, the NCBI Eukaryotic Genome Annotation Pipeline[47] was used to annotate genes, transcripts and proteins on bStrHab1_v1.p (GCF_004027225.1), the primary pseudohaplotype of the assembly. Next, the assembly was first masked with WindowMasker[48]. Nearly 350 million RNA-Seq reads from kākāpō brain and kidney tissue, 700 million RNA-Seq from other species in the Psittacidae family (*Amazona ventralis*, *Aratinga solstitialis* and *Platycercus eximius*), 351,938 IsoSeq consensus reads from liver and 8.1 million Oxford Nanopore reads from brain and liver tissue were retrieved from SRA and aligned to the masked genome, along with 9,075 known RefSeq transcripts, 42,131 GenBank transcripts, and 756,464 ESTs from birds. The alignments were performed using BLAST[49] followed by the global aligner Splign[50] for all transcripts except the IsoSeq and Oxford Nanopore data, which were aligned with Minimap2[39]. In the absence of kākāpō proteins in GenBank, the proteins that were chosen as candidates for alignment to the genome by BLAST and ProSplign spanned a wide range of other birds, including, in particular, RefSeq proteins from *Columba livia*, *Parus major*, *Gallus gallus.* RefSeq proteins for *Xenopus laevis* and human were also included.

The resulting transcript and protein alignments were used as evidence to predict the structures and boundaries of gene and transcript models. *Ab initio* extension or joining/filling of

partial open reading frames in compatible frames of these preliminary models was performed by Gnomon (https://www.ncbi.nlm.nih.gov/genome/annotation_euk/gnomon/), using a hidden Markov model trained on kākāpō, in the minority of cases where the overlapping alignments did not define a complete model but the coding propensity of the CDS on the alignments was sufficiently high. RNAs were predicted with tRNAscan-SE:1.2326[51] and small non-coding RNAs were predicted by searching the RFAM 12.0 HMMs for eukaryotes using cmsearch from the Infernal package[52]. The annotation of bStrHab1_v1.p (NCBI *Strigops habroptila* Annotation Release 100) resulted in 16,060 protein-coding genes, 3,131 non-coding genes and 165 pseudogenes (see details in https://www.ncbi.nlm.nih.gov/genome/annotation_euk/Strigops_habroptila/100/).

For the Ensembl and NCBI assembly pipelines, RNA data for kākāpō was extracted from brain and kidney tissue from a female chick that died at three-days old through grinding on liquid nitrogen, tissue disruption in Trizol reagent (Thermofisher), followed by extraction of total RNA using Qiagen RNAeasy technologies. mRNA was purified from 23 µg and 11 µg of kidney and brain total RNA respectively using the Dynabeads mRNA Purification Kit (Invitrogen, U.S.A.). 124 ng and 229 ng of purified mRNA from kidney and brain, respectively, was used for library preparation with the Direct cDNA Sequencing DCS109 kit (Oxford Nanopore Technologies, U.K.). Libraries were sequenced for 48 hours on R9.4.1 MinION flow cells using two Mk1B MinION sequencers (Oxford Nanopore Technologies). Raw data were base-called using guppy_basecaller 3.1.5 in high accuracy mode (dna_r9.4.1_450bps_hac) on a NVIDIA GeForce RTX 2070 GPU.

1.5 Generation time for demographic reconstructions

It was previously thought that generation time in kākāpō was of ~25 years[53]. However, this number may be an overestimate because it is based on the average age to first reproduction in the extant population, with females recorded to have bred between 5 and 18 years while males have bred between 11 to 23 years. However, in this small population, only a few older dominant males have reproduced (Daryl Eason, DOC, pers. comm.) and many young males have thus not had the opportunity to breed yet. We thus used a shorter generation time, assuming that a time of 15 years may be more biologically realistic in a large and natural kākāpō population.

Supplemental figures

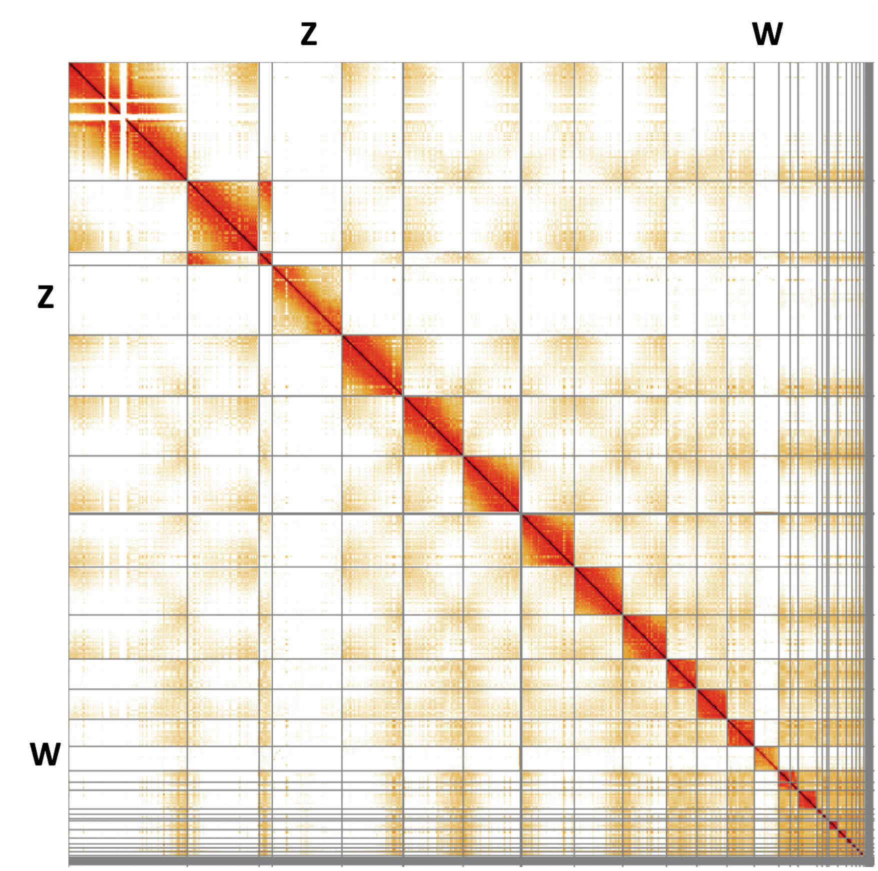

Figure S1. HiC 2D map of the curated bStrHab1 assembly, Related to STAR Methods. The assembly plot was generated using HiGlass[54]. Black lines depict scaffold boundaries. The Z and W sex chromosomes are shown in bold letters. The kākāpō chromosome assignments were based on HiC data and over 99% of the sequence was assignable to arm-to-arm chromosomes via digital karyotyping, where compartimentalization of the HiC signal is interpreted as outline for a self-contained sequence unit, (i.e., chromosome).

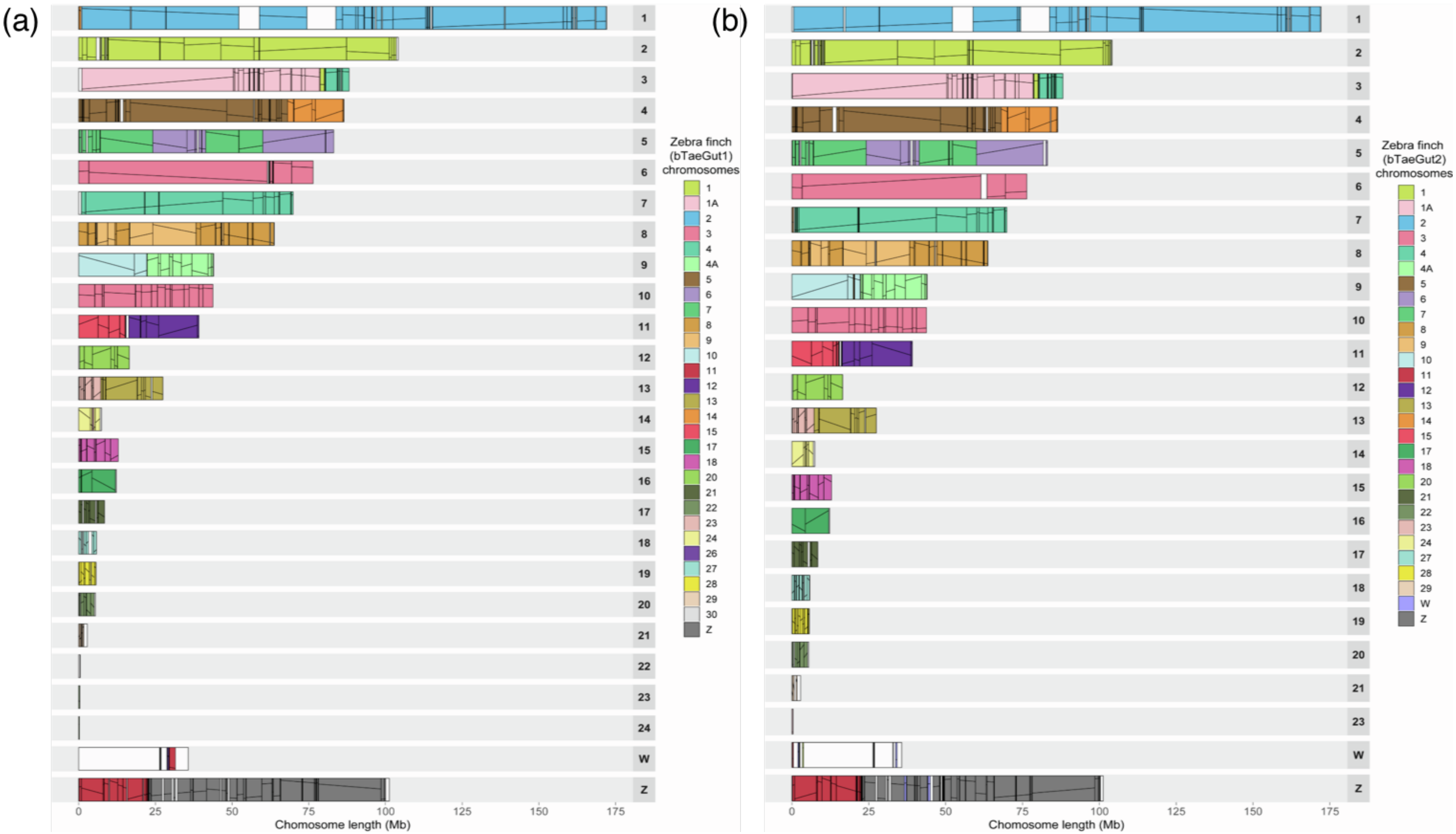

Figure S2. Ideogram of kākāpō chromosomes relative to (a) male and (b) female zebra finch, Related to STAR Methods. Numbered rectangles represent kākāpō chromosomes, and colored blocks inside represent regions of homology with zebra finch chromosomes. Lines within the colored blocks represent block orientation.

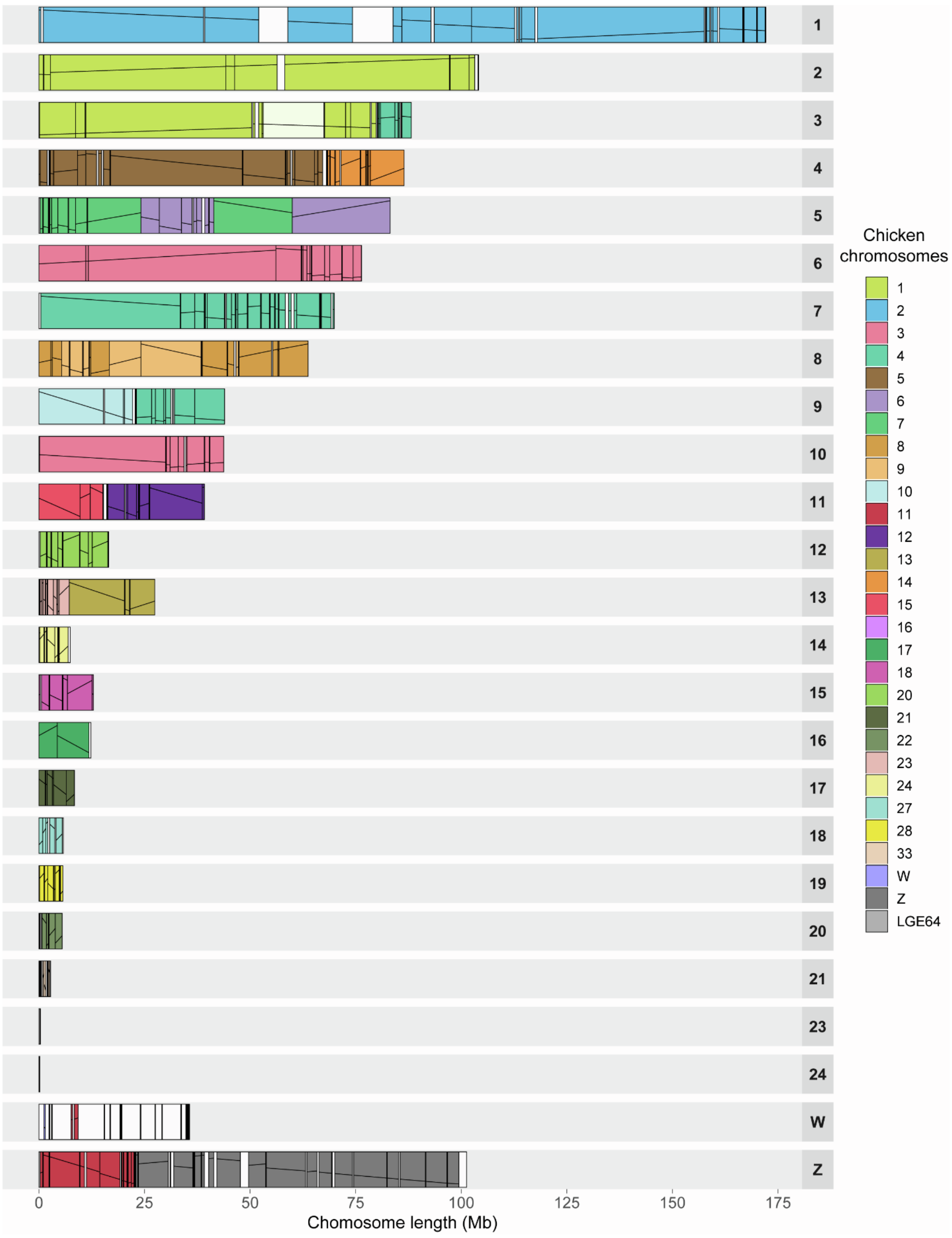

Figure S3. Ideogram of kākāpō chromosomes relative to chicken, Related to STAR Methods. Numbered rectangles represent chromosomes, and colored blocks inside represent regions of homology with chicken chromosomes. Lines within the colored blocks represent block orientation. The Z chromosome

homolog is fused with the chromosome 11 equivalent of chicken based on read and mapping data. Also, two autosomes (Chr 16 and 18) had 0 gaps each and no evidence of collapsed repeats, and all others had only 1 to 24 gaps, which may be explained by the fact that in birds most microchromosomes are acrocentric (i.e., centromere at the extremity of chromosomes).

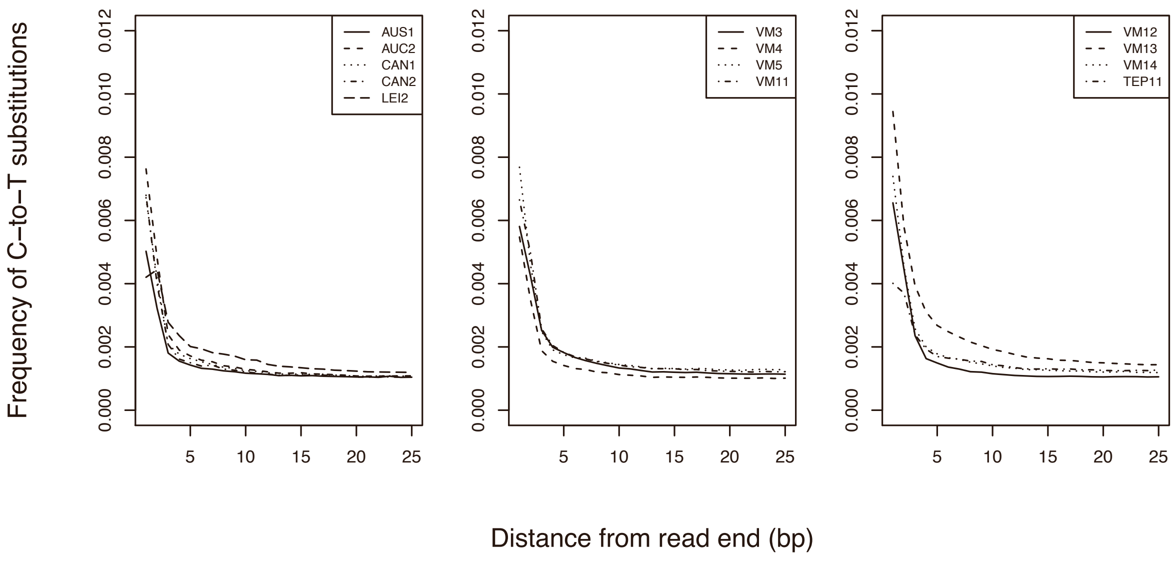

Figure S4. **DNA-damage in 13 historical genomes, Related to STAR Methods.** Fraction of C-to-T substitutions by distance from the read-end averaged over the historical genomes. The USER treatment removed the majority of typical post-mortem damage patterns, with the historical genomes showing slightly elevated levels of these substitutions.

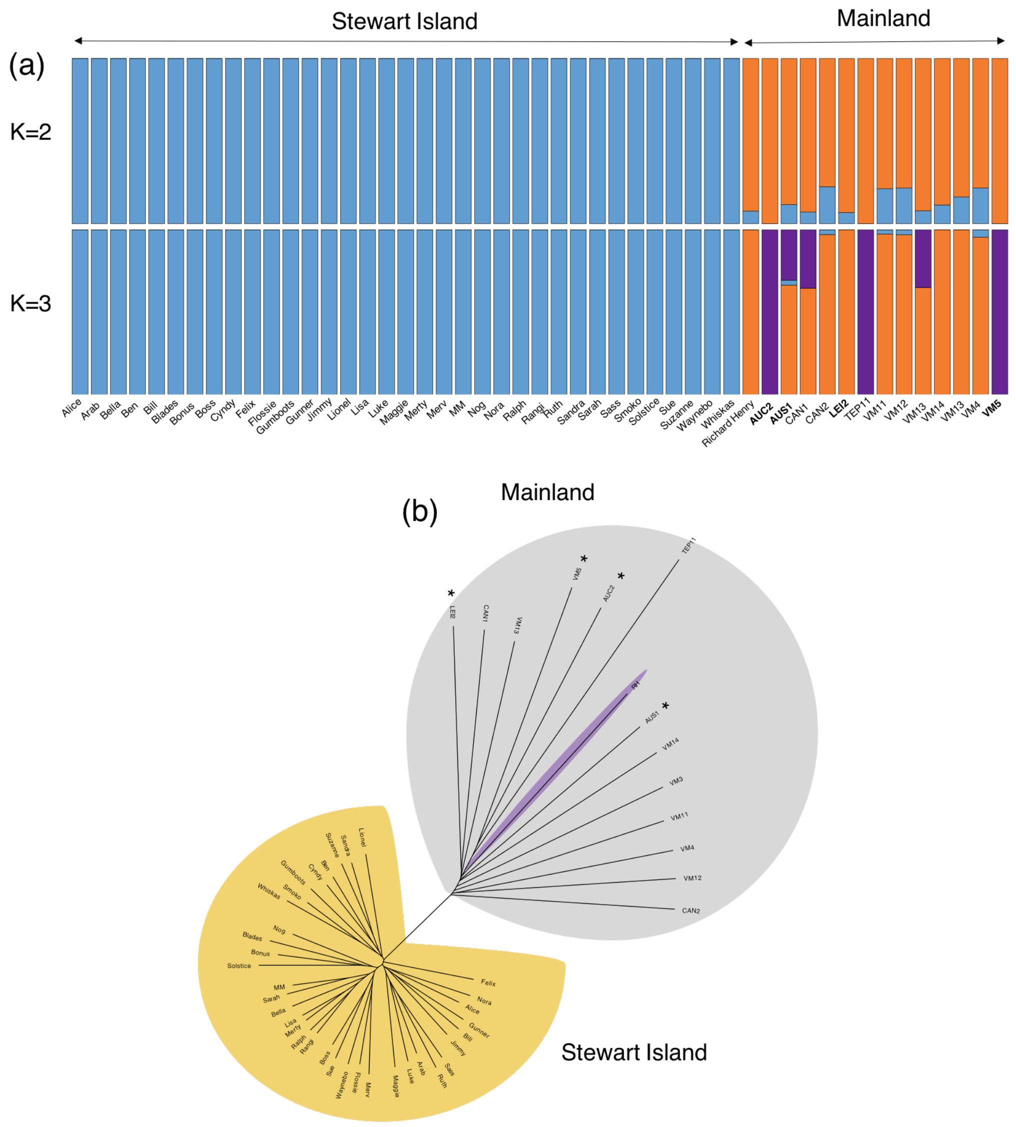

Figure S5. Population structure in 35 Stewart Island and 14 mainland kākāpō, Related to Figure 1 and STAR Methods. (a) Individual clustering assignment in ADMIXTURE. The lowest cross-validation error was obtained for K=2. Individuals in bold are museum specimen showing inconsistencies between genetic clustering and specimen labelling, potentially as a result of museum specimen mislabelling[2]. **(b)** Neighbour-joining tree with asterisks representing museum specimens showing inconsistencies between genetic clustering and specimen labelling. All mislabelled samples were analysed as part of their genetically assigned population in all downstream analyses.

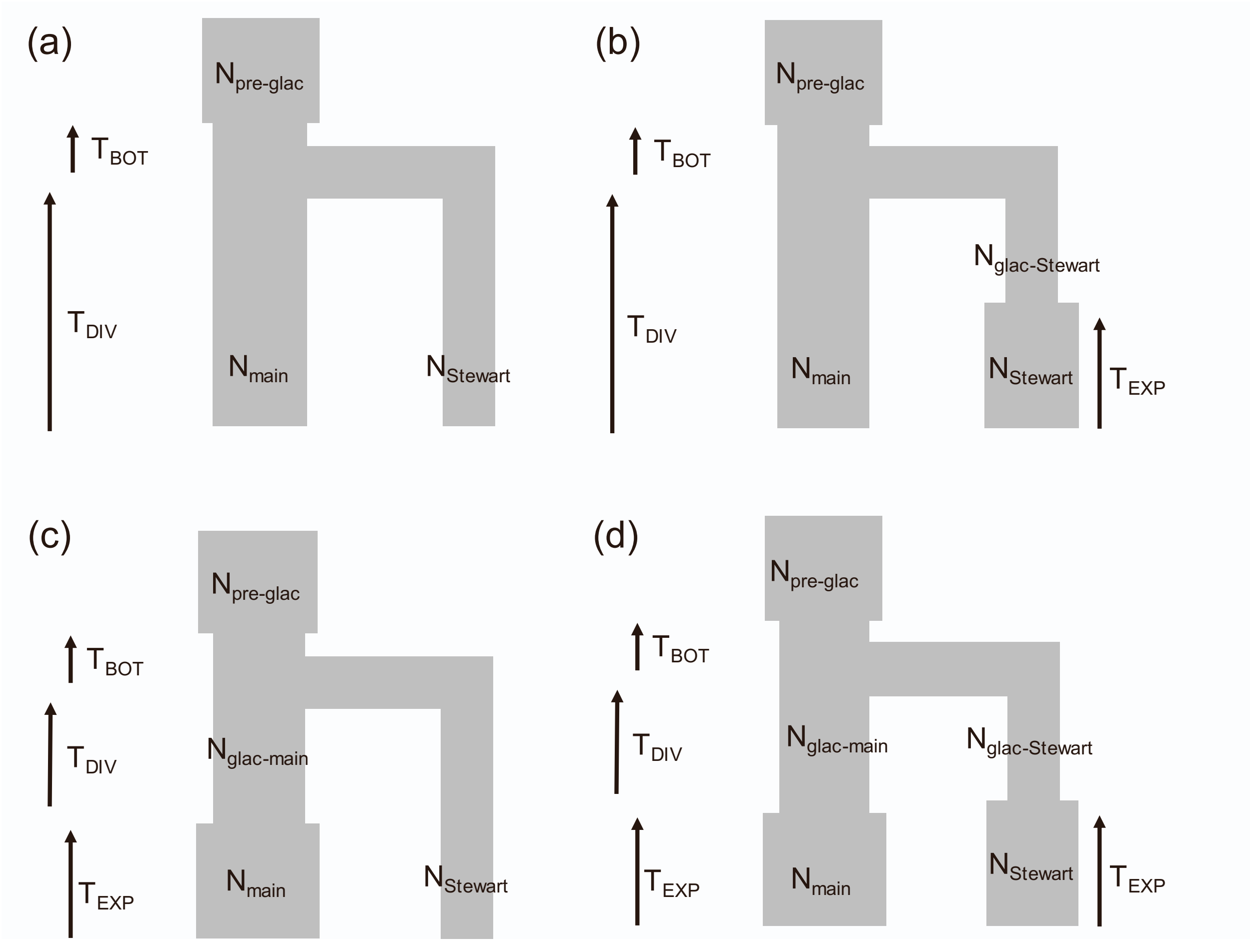

Figure S6. Demographic scenarios for mainland and Stewart Island kākāpō, Related to Figure 1 and STAR Methods. **(a)** *Post-glacial divergence*, **(b)** *Post-glacial divergence followed by Stewart Island population expansion*, **(c)** *Post-glacial divergence followed by Mainland population expansion* and, **(d)** *Post-glacial divergence followed by Stewart Island and Mainland population expansion.* Note that the recent population expansion for the Stewart and Mainland populations (T_{EXP}; STAR Methods) were not constrained in order to allow for either a bottleneck or population expansion to occur. These events are however referred to as expansions as they were supported by the best model. Scenario (d) also depicted in Fig. 1d obtained the highest support as estimated by AIC's weight (w) (Table S2).

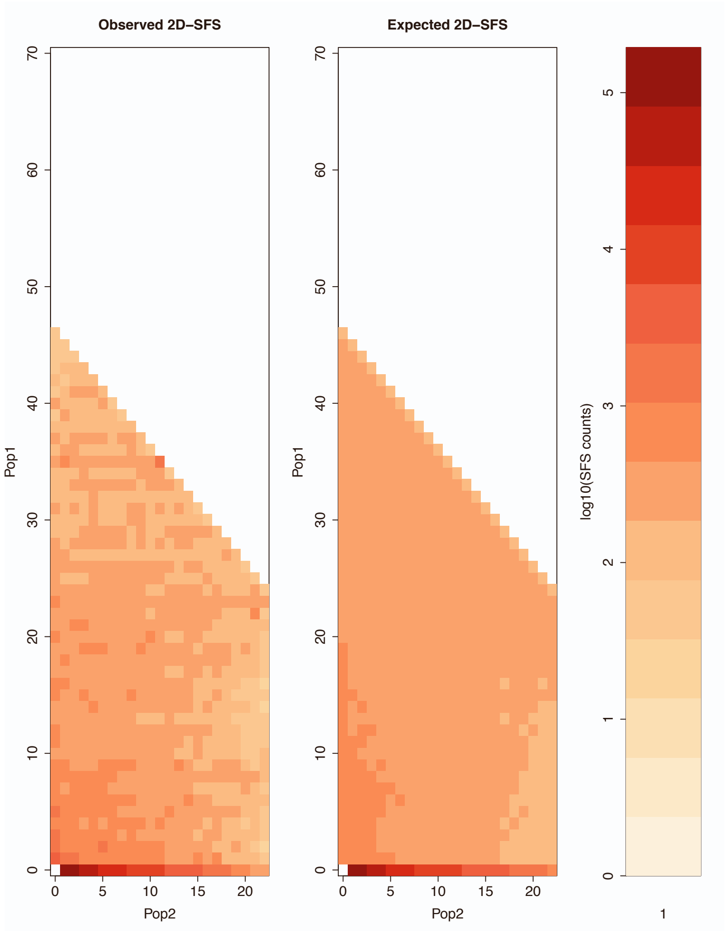

Figure S7. Comparison of observed (left panel) and expected (right panel) 2D-SFS for the preferred scenario of *Post-glacial divergence followed by Stewart Island and mainland population expansion*, Related to Figure 1 and STAR Methods. In the main two panels, each cell represents the number of alleles which occur at frequency *x* in Population 1 (Stewart Island) and frequency *y* in Population 2 (Mainland). The colored bar to the right depicts the count of allele on a logarithmic scale that occur at a x-y frequency combination in the main two panels.

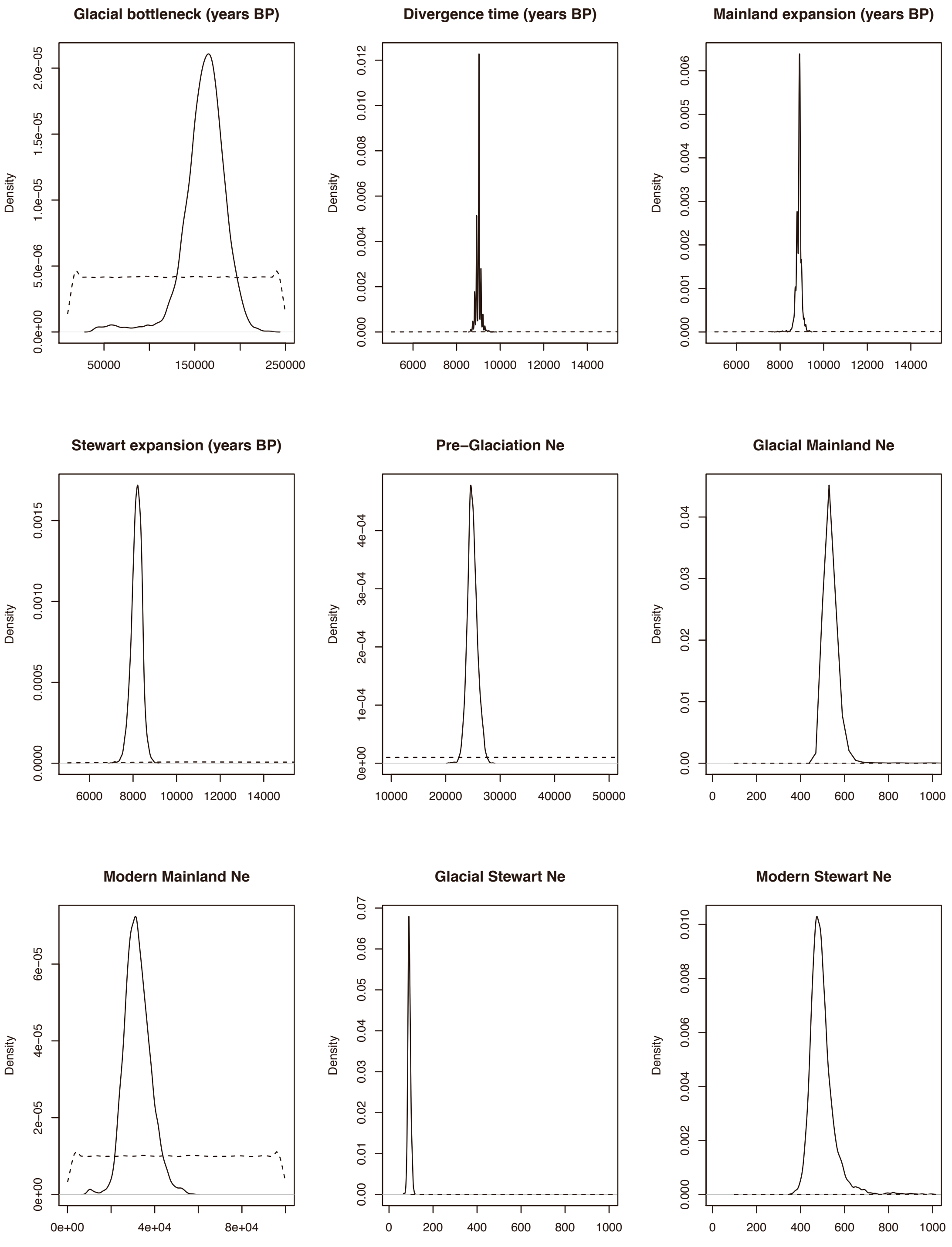

Figure S8. Posterior distributions of effective population sizes, glaciation bottleneck and divergence times and change in effective population size for the preferred scenario of *Post-glacial divergence followed by Stewart Island and mainland population expansion*, Related to Figure 1 and STAR Methods. Dashed and full lines represent prior and posterior distributions, respectively.

Table S2. Relative likelihood of the three different demographic models tested and depicted in Figure S6, Related to Figure 1 and STAR Methods. A scenario of '*Post-glacial divergence followed by Stewart Island and Mainland population expansion*' obtained most support based on AIC's weight.

**Model**	**Log MaxEstLhood**	**N of estimated parameters**	**AIC**	**Δ**	**AIC's weight (w)**
a) Post-glacial divergence	-1522664.81	7	3045343.61	126184.332	0.00E+00
b) Post-glacial divergence and Stewart Island population expansion	-1516988.68	9	3033995.35	114836.0731	0.00E+00
c) Post-glacial divergence and Mainland expansion	-1503772.70	9	3007563.41	88404.128	0.00E+00
d) Post-glacial divergence and Stewart Island and Mainland expansion	-1459568.64	11	2919159.28	0	1.00E+00

[a]Based on the best of 50 likelihood estimates of each scenario

[b]AIC_i=2d 2-ln($Lhood_i$)

[c]Δ_i =AIC_i -min(AIC)

[d] $w_i = \frac{\exp(-0.5\Delta_i)}{\sum_r^R \exp(-0.5\Delta_r)}$

Table S3. Posterior distributions of parameters for the preferred scenario d) *Post-glacial divergence followed by Stewart Island and mainland population expansion*, Related to Figure 1 and STAR Methods. Times are in years BP. Note that the recent population expansion for the Stewart and Mainland populations ($T_{EXP;}$ STAR Methods) were not constrained in order to allow for either a bottleneck or population expansion to occur. These events are however referred to as expansions as they were supported by the best model.

Parameter	mode	95% CI	
		Lower CI	Higher CI
T_{BOT}	157,800	130,800	198,165
T_{DIV}	9,030	8,805	9,120
$T_{EXP\text{-}main}$	8,910	8,670	9,015
$T_{EXP\text{-}Stewart}$	8,205	7,740	8,505
$N_{pre\text{-}glac}$	24,714	23,466	26,529
$N_{glac\text{-}main}$	510	505	570
N_{main}	28,135	23,026	41,850
$N_{glac\text{-}Stewart}$	91	81	102
$N_{Stewart}$	485	415	568

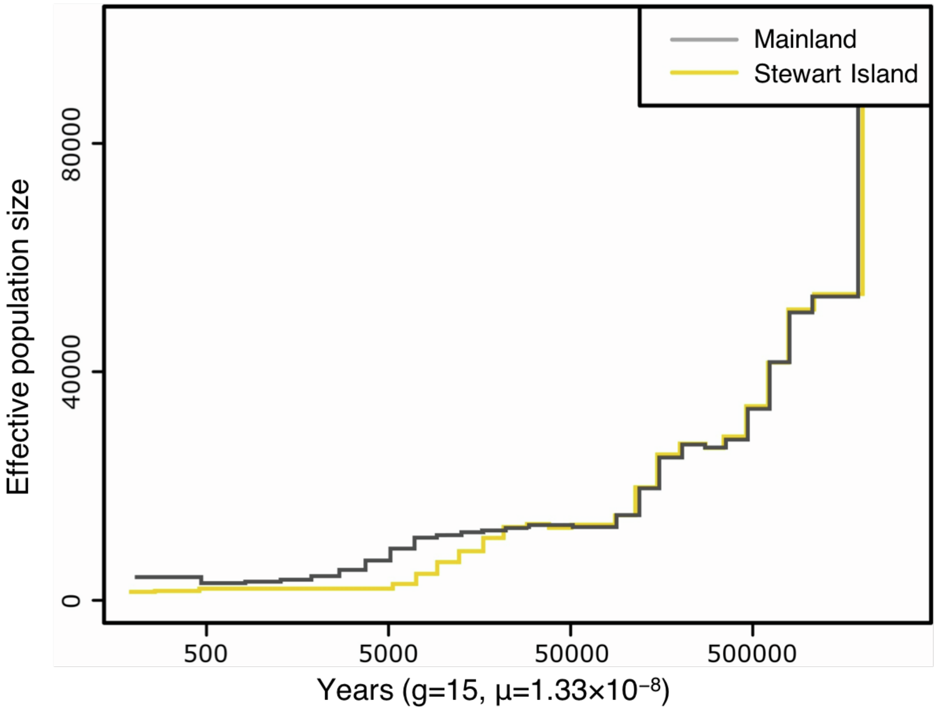

Figure S9. Past demography of kākāpō using the MSMC2 approach, Related to Figure 1 and STAR Methods. The x-axis corresponds to time before present in years on a log scale, assuming a mutation rate of 1.33×10^{-8} substitutions/site/generation and a generation time of 15 years[53]. At the end point of the curve some 300 y BP, N_e estimates for the Stewart Island and the mainland populations were ~300 and ~14,500, respectively. However, it is worth noting that N_e estimates at the end point of the curve should be taken with caution due to the low number of coalescent events[55].

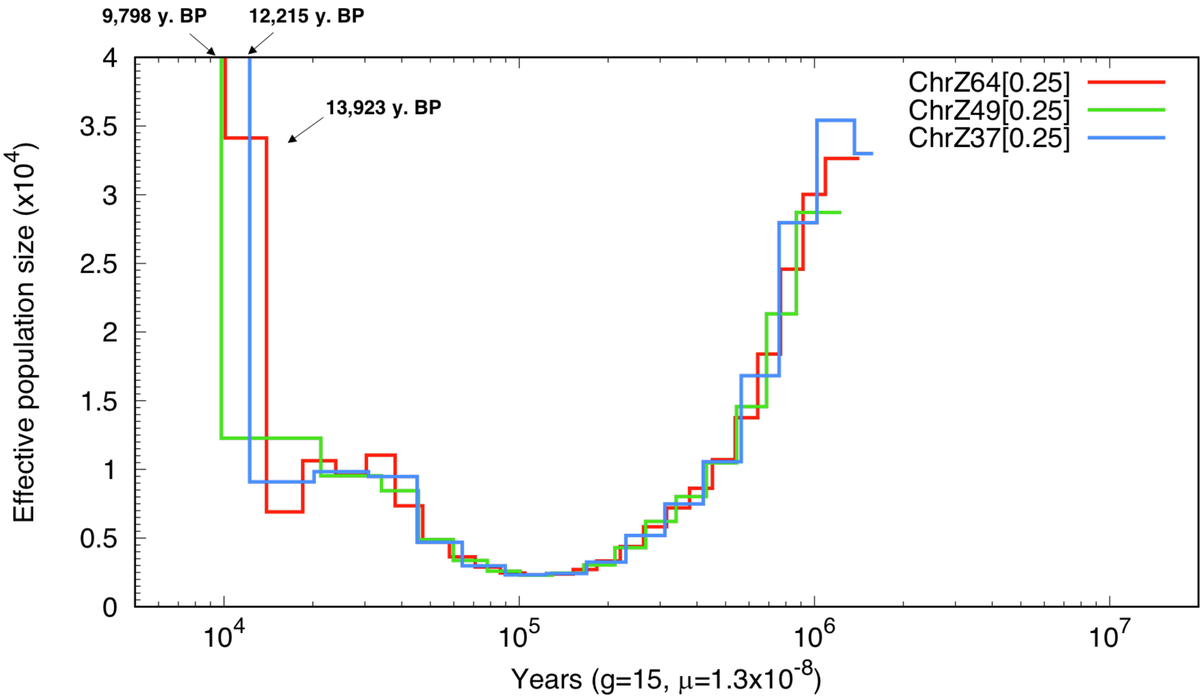

Figure S10. Comparison of divergence estimates between the ancestral population of mainland and Stewart Island using the PSMC approach, Related to Figure 1 and STAR Methods. The x axis corresponds to time before present in years on a log scale, assuming a mutation rate of 1.33×10^{-8} substitutions/site/generation and a generation time of 15 years[53]. The red, green and purple curves represent pseudo-diploid chromosome Z rescaled by factor of 0.25 (sex-chromosome/autosome ratio: 0.75) and using 64, 49 and 37 discrete intervals, respectively.

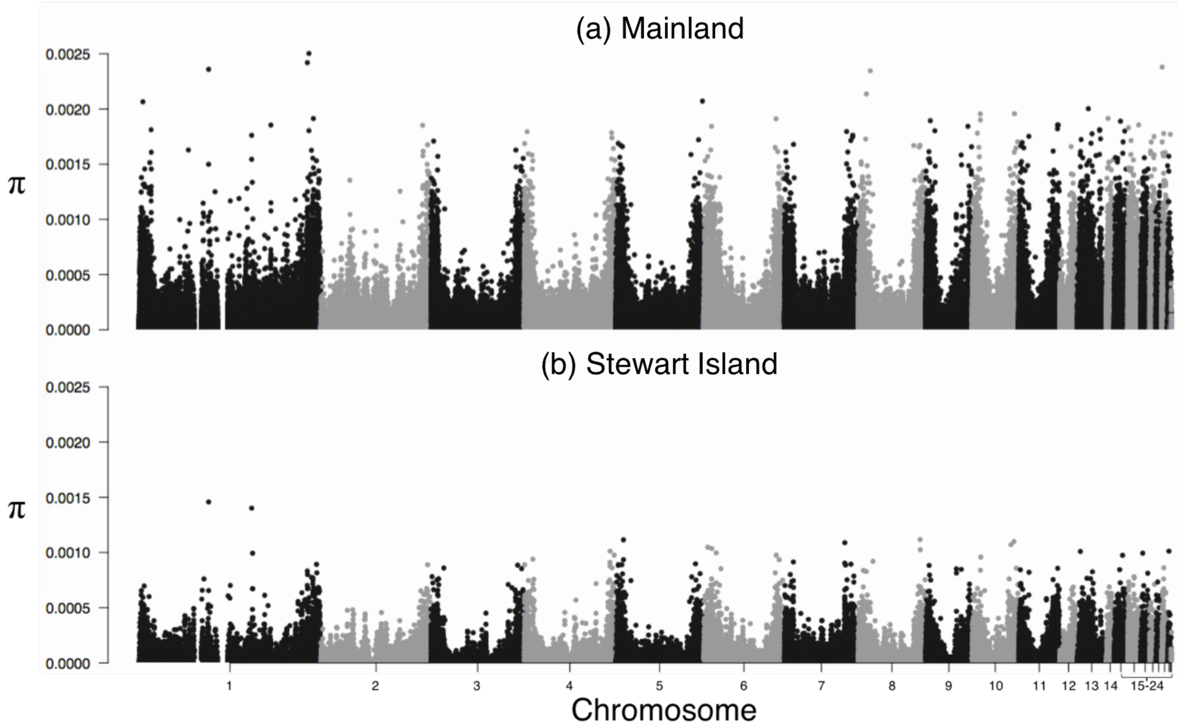

Figure S11. Genome-wide nucleotide diversity (π) estimates, Related to Figure 2 and STAR Methods. Autosomal genome-wide population-level nucleotide diversity for **(a)** Mainland (including Richard Henry) and **(b)** Stewart Island kākāpō. The higher π at the edges of chromosomes compared to the centre of chromosomes reflects the recombination landscape of the bird genome[56,57], with lower recombination rates in the centres of macrochromosomes, relative both to their edges and the micro-chromosomes[56,58].

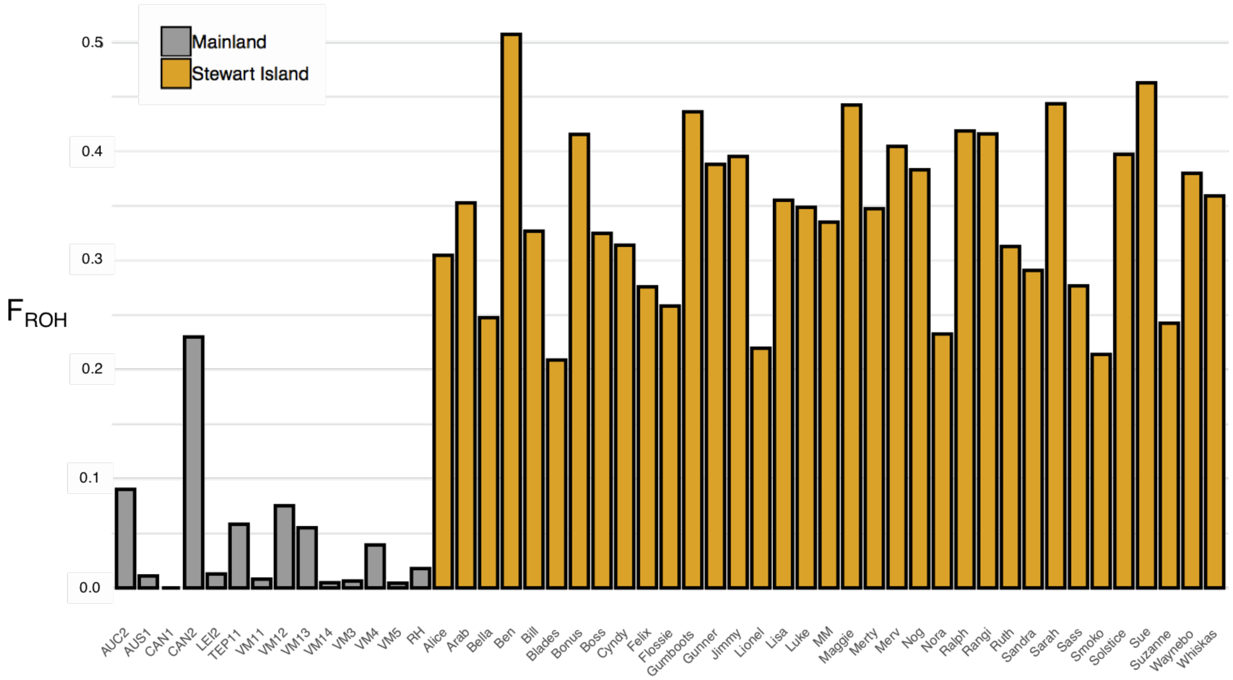

Figure S12. Proportion of the genome in Runs of Homozygosity (F_{ROH}), Related to Figure 2. F_{ROH} was calculated by including only ROH ≥2Mb in 14 mainland and 35 Stewart Island kākāpō using the sliding window approach implemented in PLINK. The average F_{ROH} for ROH ≥100kb was 15.1% ±6.5 SD, and 52.9% ±6.6 SD (P<0.01), for the mainland and Stewart Island, respectively. The average F_{ROH} for ROH ≥2Mb was 4.4% ±6.1 SD and 34.3% ±7.7 SD (P<0.01) for the mainland and Stewart Island, respectively.

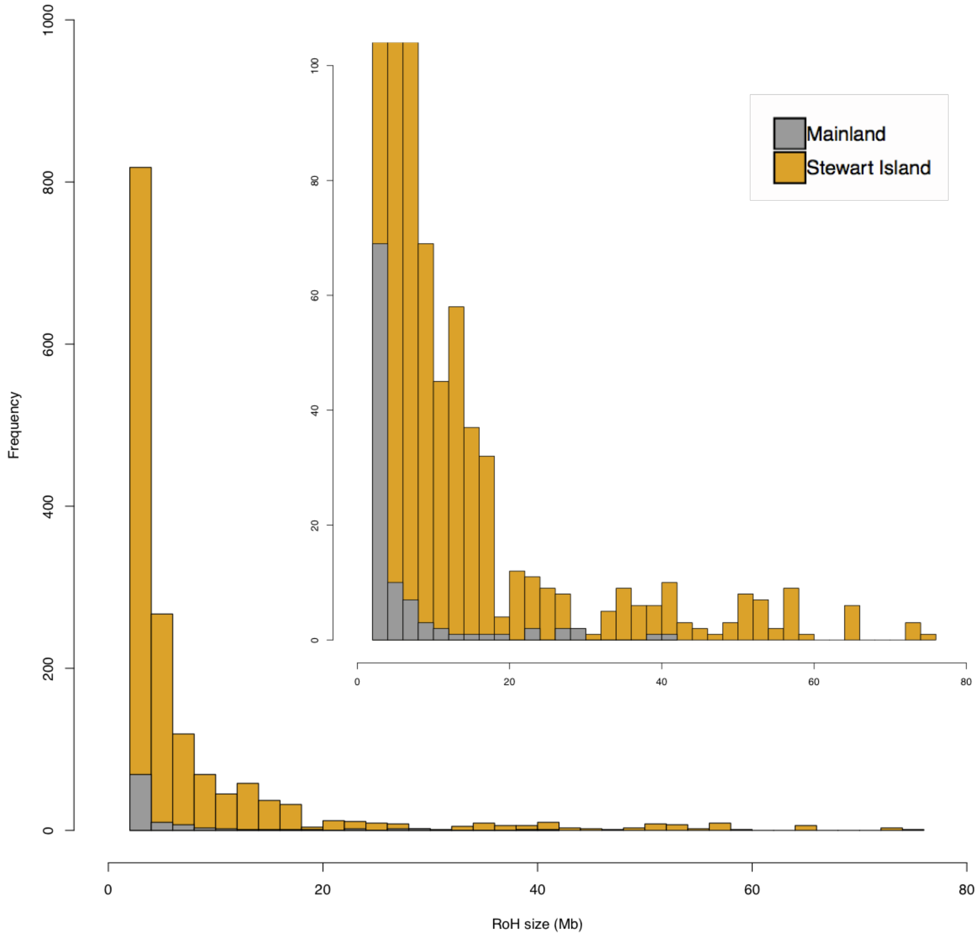

Figure S13. Frequency distribution of runs of homozygosity (ROH) size, Related to Figure 2. Only ROH ≥2Mb are shown. Inset shows the magnification for clarity. We identified a total of 22,458 ROH and the longest ROH sizes for the mainland and Stewart Island were 41.34Mb and of 75.61Mb, respectively.

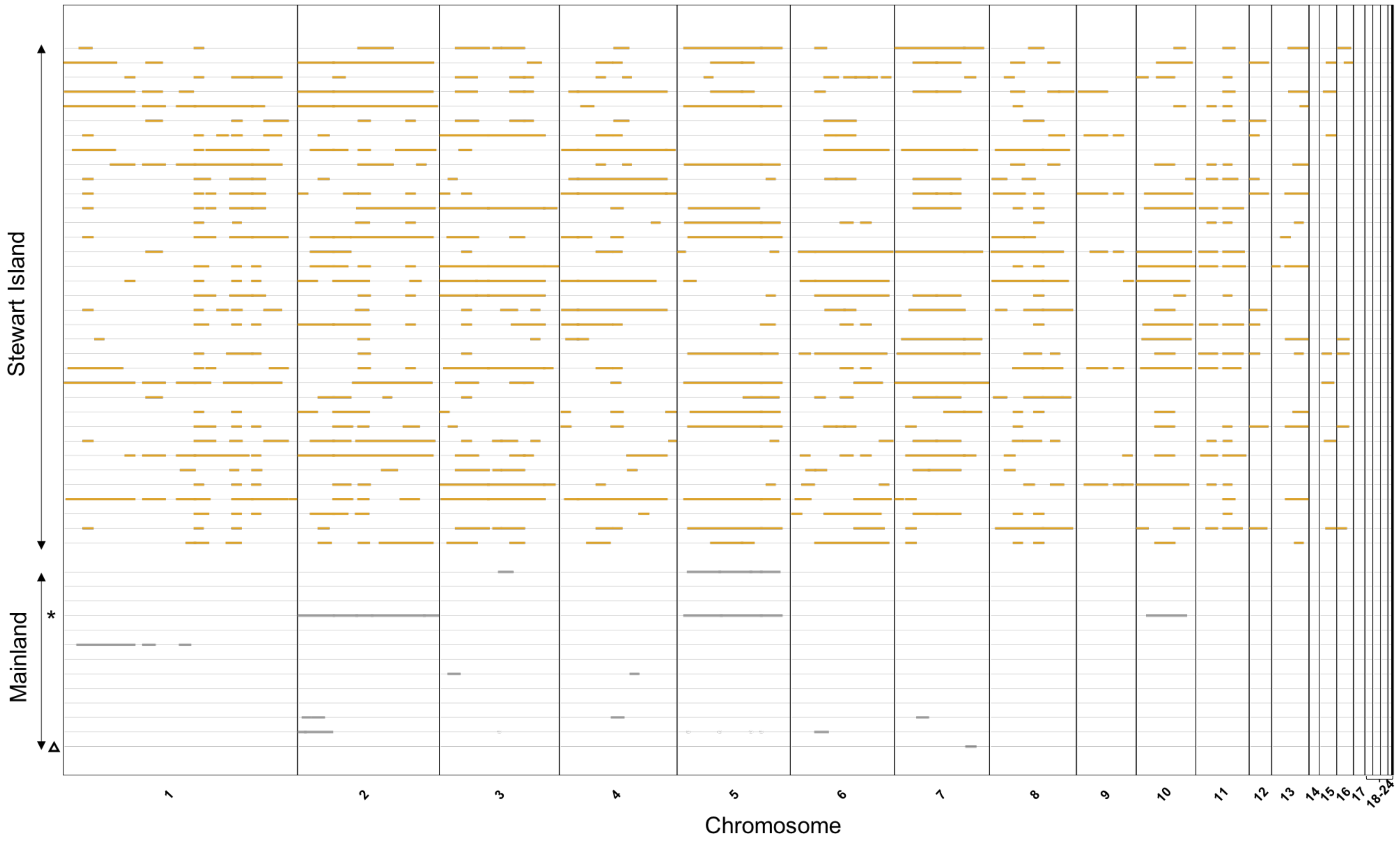

Figure S14. Distribution of ROH ≥5Mb along the 24 autosomes for 35 Stewart Island (top panel, yellow) and 14 mainland (bottom panel, grey) kākāpō, Related to Figure 2. We used the sliding window approach implemented in PLINK. The asterisk and triangle depict the mainland historical sample with the highest F_{ROH} (CAN2) and Richard Henry, respectively. Chromosomes are numbered and separated by vertical lines. In some cases, ROH span nearly entire chromosomes.

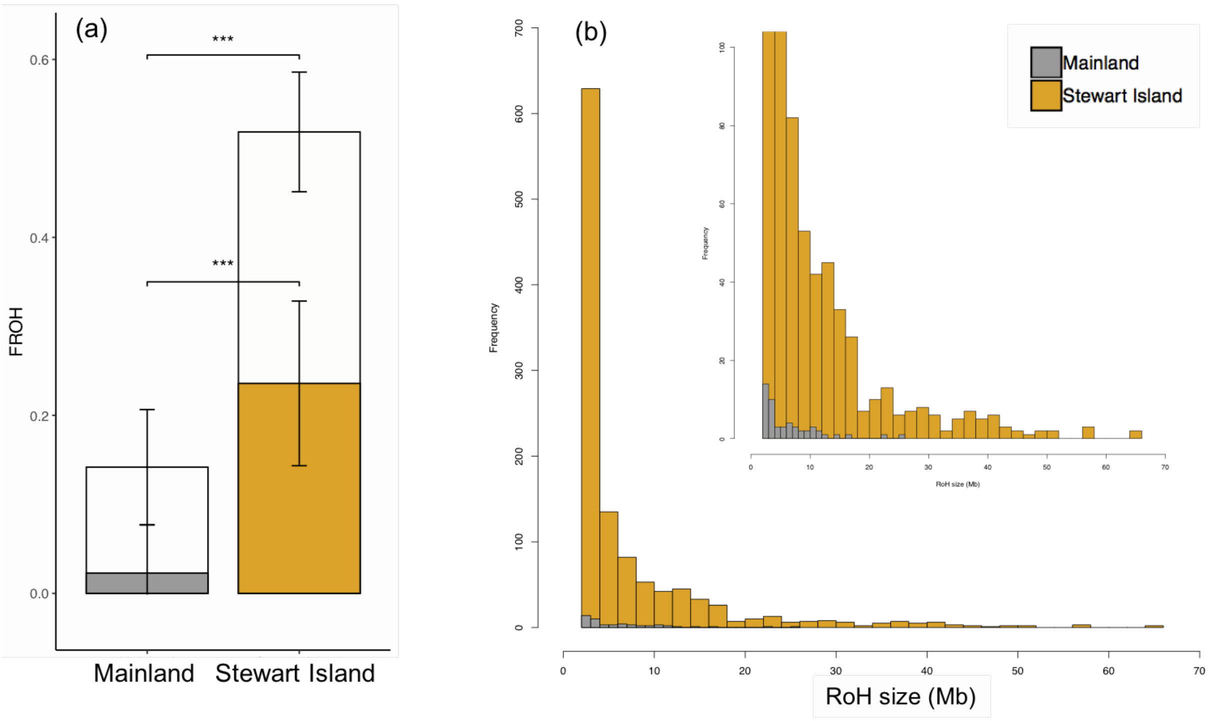

Figure S15. Average proportion of the genome in Runs of Homozygosity (F_{ROH}) and distribution of runs of homozygosity (ROH) in mainland and Stewart Island kākāpō, Related to STAR Methods. Results were obtained using more stringent parameters to assess the robustness of ROH estimates. **(a)** Open bars show total proportion of the genome in ROH ≥100kb and solid bars show proportions in ROH of length ≥2Mb. Bars extending from the mean values represent the standard deviation (Welch's two-sample t-test; ***P<0.001). The average F_{ROH} for ROH ≥100kb was 14.2% ±6.5 SD, and 51.8% ±6.7 SD (P<0.01), for the mainland and Stewart Island, respectively. The average F_{ROH} for ROH ≥2Mb was 2.3% ±5.4 SD and 23.6% ±9.3 SD (P<0.01) for the mainland and Stewart Island, respectively. **(b)** Frequency distribution of ROH size for ROH ≥1Mb. Results are shown here for comparison with main results and were obtained using more stringent parameters such as: *homozyg-het*=1; *homozyg-density* 100; *homozyg-gap* 500. Varying the number of heterozygous sites per ROH and allowing a maximum value of 1 (*homozyg-het 1*) had the most effect on ROH detection and resulted in large ROH being broken into ROH of smaller size, thereby increasing the overall number of ROH to 33,857. The longest ROH sizes for the mainland and Stewart Island were 25.19Mb and of 65.79Mb, respectively.

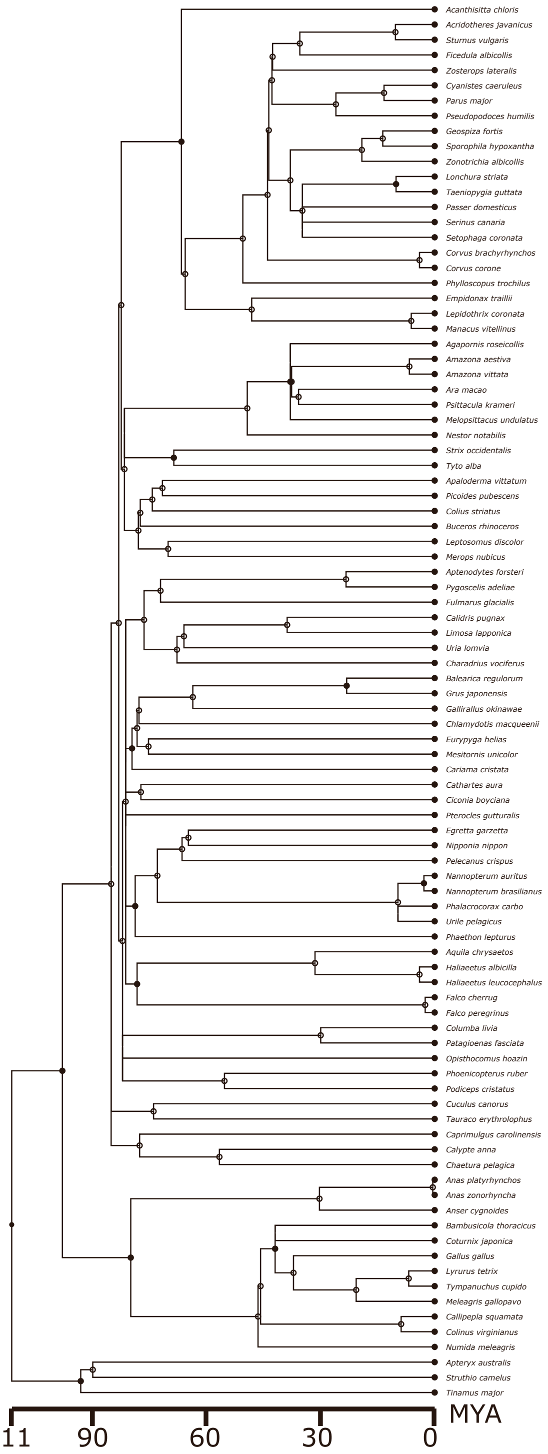

Figure S16. Bird genomes (n=135) used to calculate GERP-scores, Related to STAR Methods. Divergence time estimates were obtained from[59].

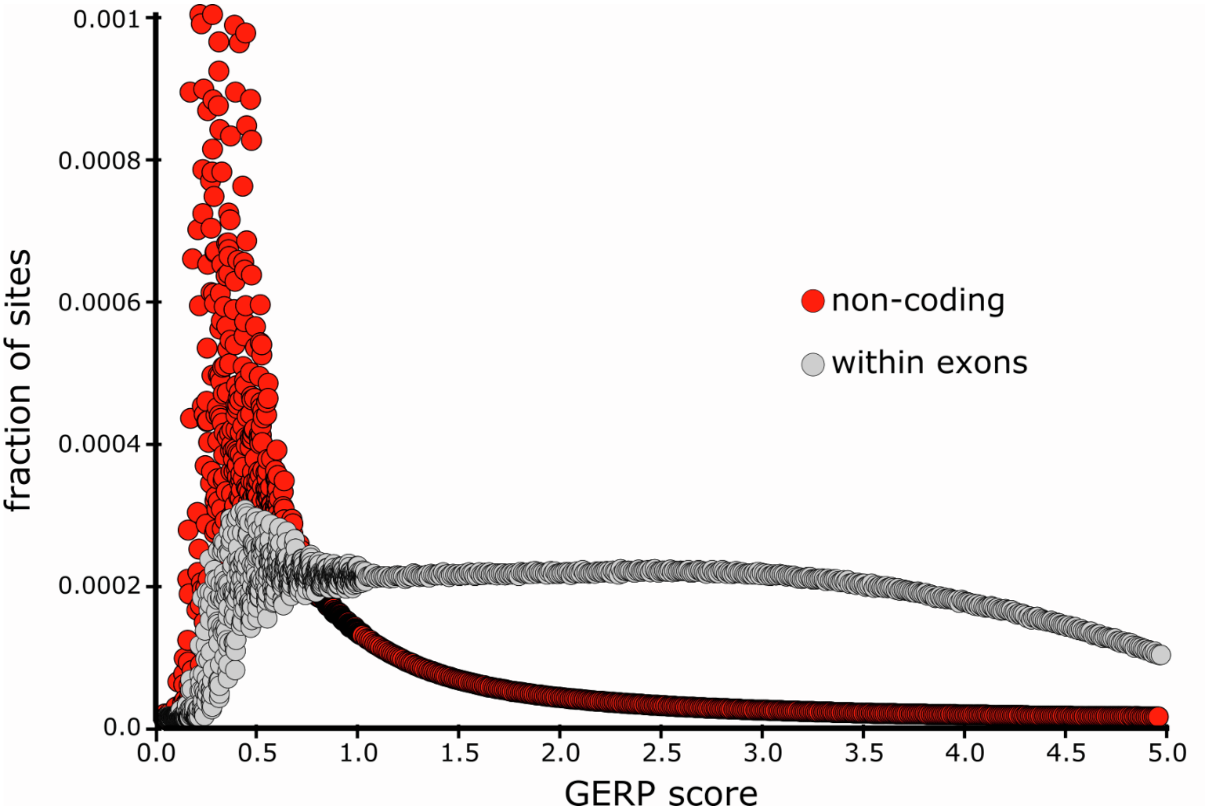

Figure S17. **Distribution of GERP-scores subdivided by non-coding regions (red) and within exons (grey), Related to STAR Methods.** The peak for low GERP scores (<1) is caused by stochastic effects as only few genomes align in these fast-evolving regions. The much higher GERP-scores within exons relative to outside of exons indicates that the method accurately estimates genome conservation[60]. The overlap indicates that not all exonic regions are highly conserved whereas some putatively non-coding regions are.

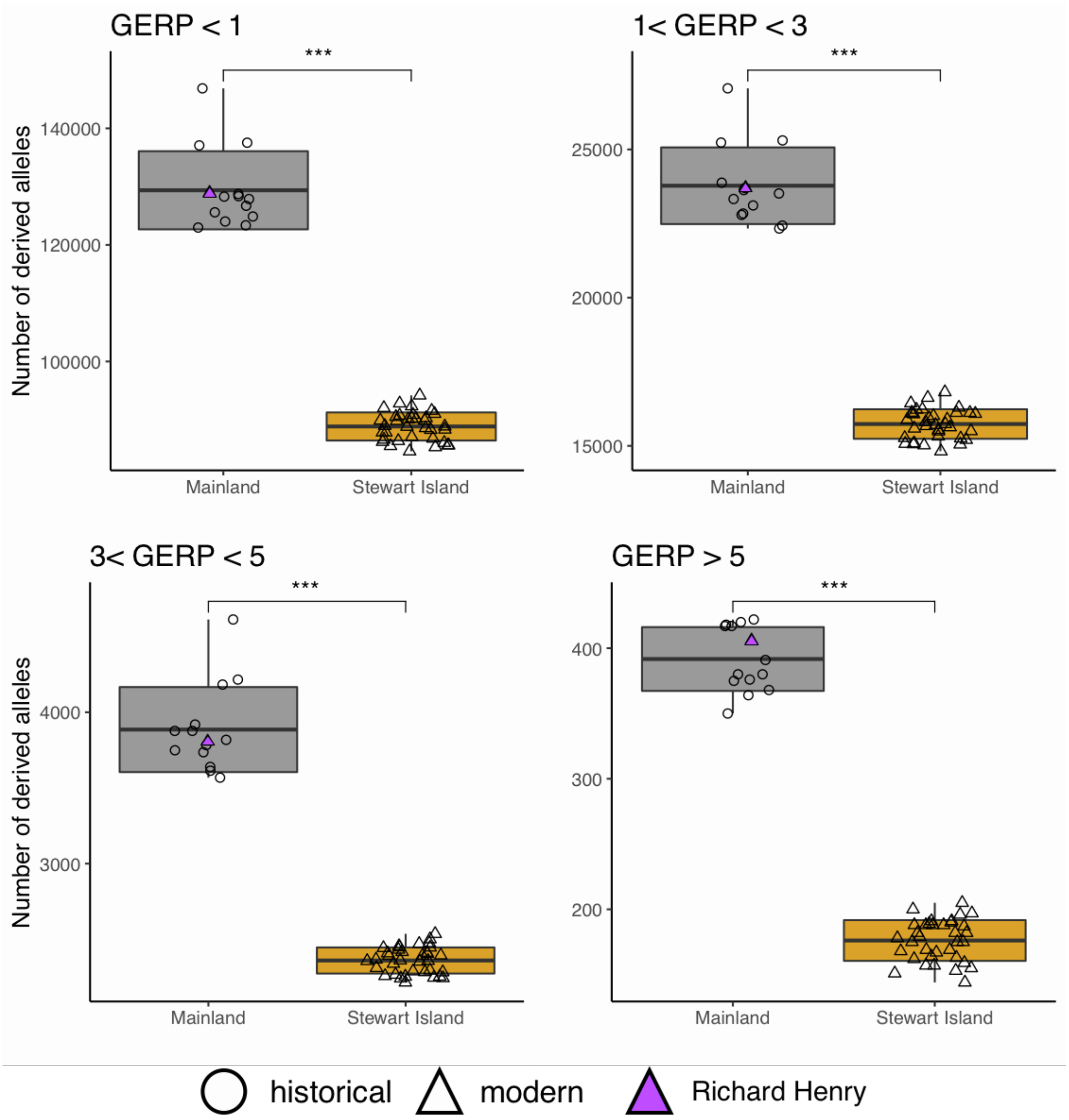

Figure S18. **Number of derived alleles per individual stratified by GERP-score, Related to Figure 3 and STAR Methods.** Middle line within boxplots bounds of boxes represent mean and standard deviation, respectively (Welch's two-sample t-test; ***P<0.001). Stewart Island kākāpō have consistently less derived alleles for all GERP-scores categories. At high GERP-scores, Stewart Island kākāpō have relatively much less derived alleles compared to the mainland population, consistent with a scenario where the most deleterious alleles are purged more efficiently than those at low GERP-scores.

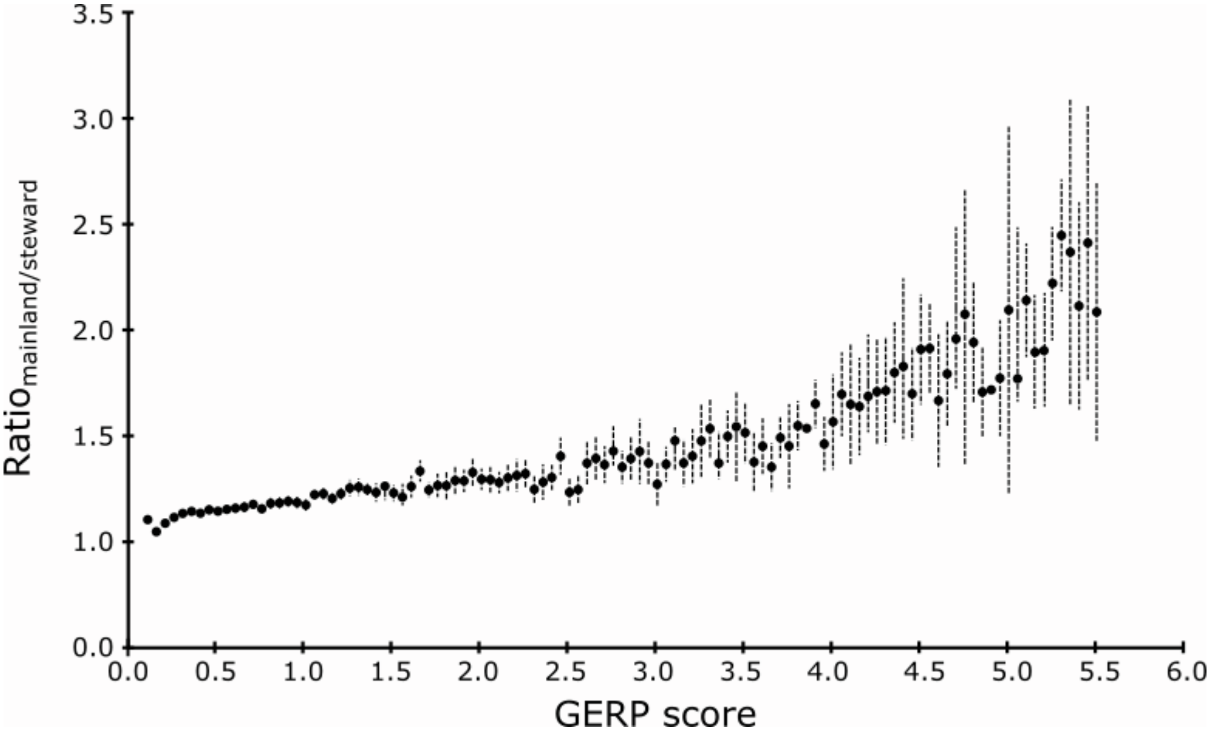

Figure S19. **The ratio of derived alleles between mainland and Stewart Island stratified by GERP-score, Related to Figure 3 and STAR Methods.** Each point represents the ratio of derived alleles between mainland and steward island by GERP (binned in intervals of 0.1) score category and striped lines show the 95% confidence interval. The difference in the number of deleterious alleles is most pronounced at sites under the strongest evolutionary constraint (i.e., GERP>2), with relatively more highly deleterious alleles in the mainland population compared to Stewart Island.

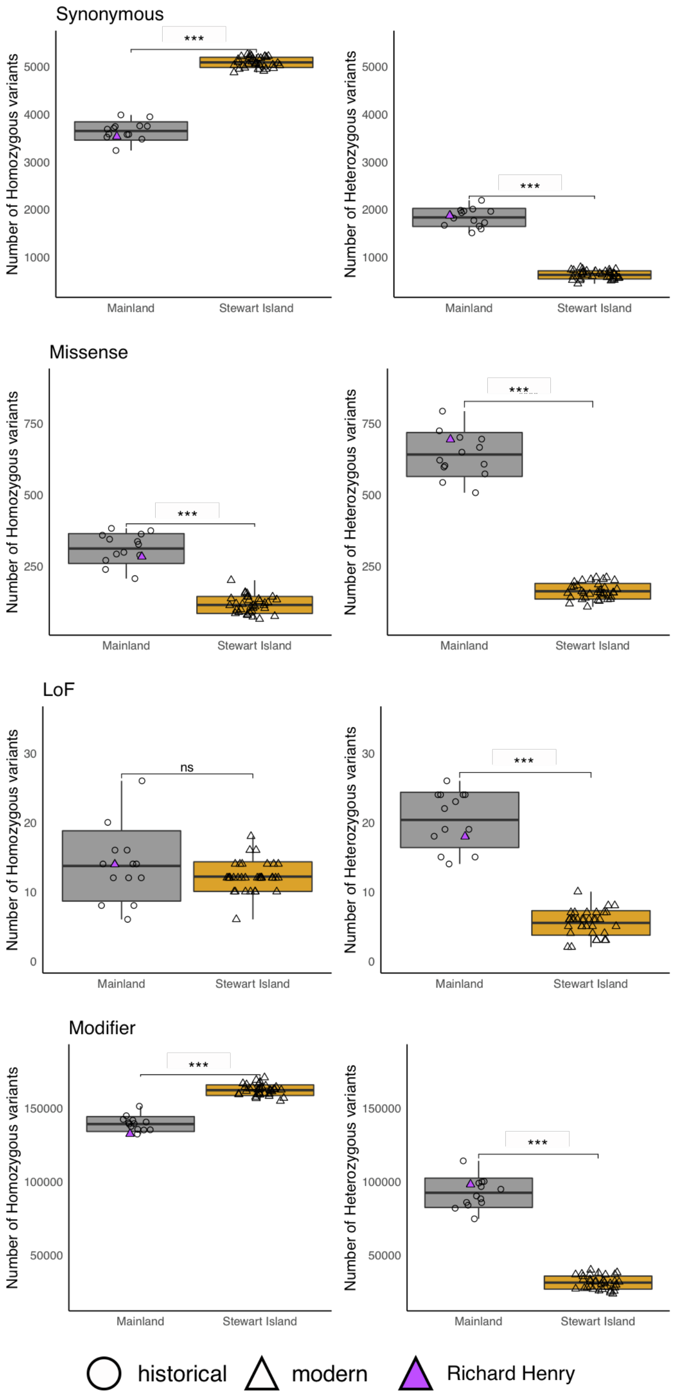

Figure S20. **Number of Synonymous, Missense, LoF and Modifier impact (i.e., downstream or upstream) variants in mainland and Stewart Island kākāpō based on the modern assembly (i.e., Jane), Related to Figure 3 and STAR Methods.** Variants are separated by homozygous or heterozygous state. Low = synonymous; Moderate = missense; High = Loss of function (LoF); Modifier = downstream or upstream. Middle thick lines within boxplots and bounds of boxes represent mean and standard deviation, respectively (Welch's two-sample t-test; ***P<0.001). The Stewart Island birds carried on average 17.6

LoF variants per bird while the mainland birds carried on average 34.1 LoF variants, with Richard Henry having 32 LoF variants. In contrast to the Stewart Island population, the mainland population had a higher number of LoF alleles in heterozygous state than the Stewart Island population, which is consistent with deleterious in heterozygous state being less to exposed to selection[61].

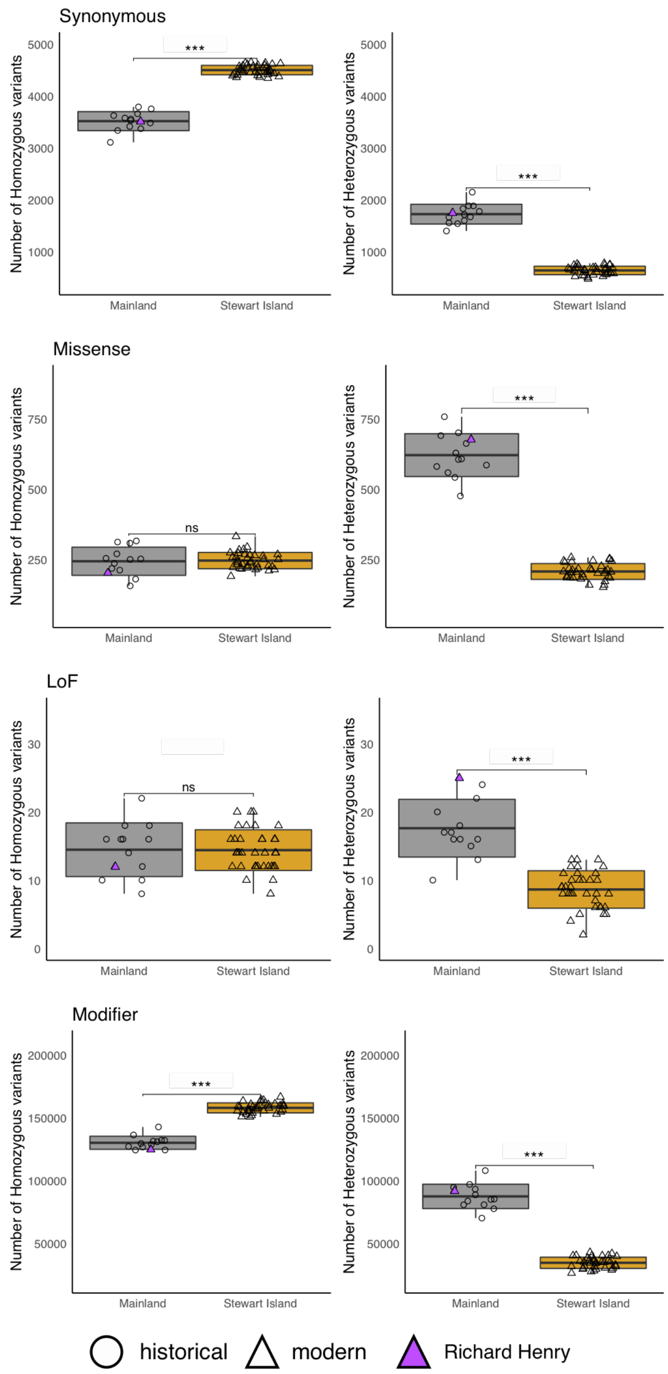

Figure S21. Number of Synonymous, Missense, LoF and Modifier impact (i.e., downstream or upstream) variants in mainland and Stewart Island kākāpō based on an historical consensus assembly (LEI2), Related to Figure 3 and STAR Methods. Variants are separated by homozygous or heterozygous state. Low = synonymous; Moderate = missense; High = Loss of function (LoF); Modifier = downstream or upstream. Middle thick lines within boxplots and bounds of boxes represent mean and standard deviation, respectively (Welch's two-sample t-test; ***P<0.001). The Stewart Island birds carried on average 23 LoF variants per bird while the mainland birds carried on average 31 LoF variants, with Richard Henry having 37 LoF variants.

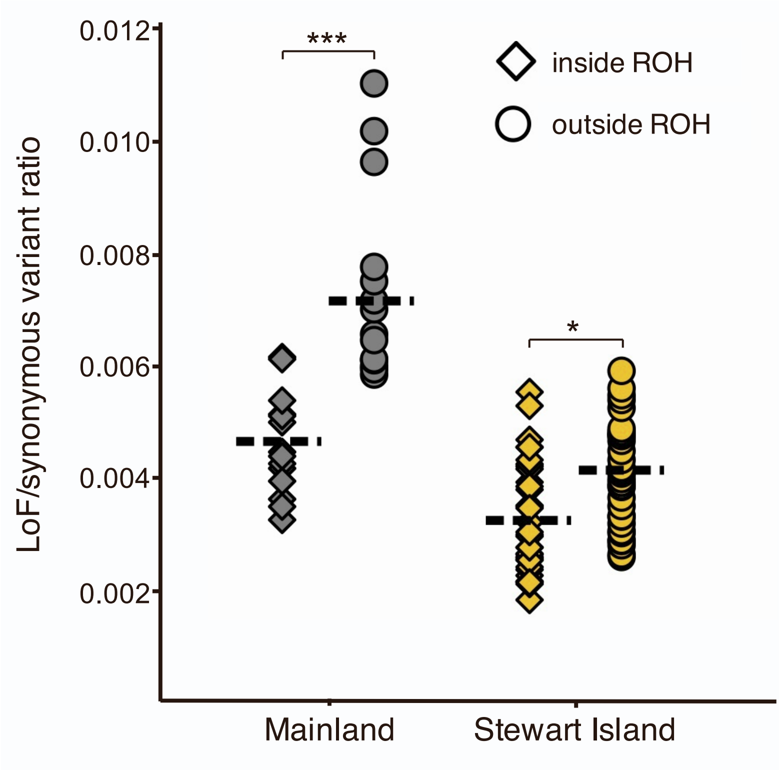

Figure S22. Rates of LoF variants in runs of homozygosity (ROH) for kākāpō, Related to Figure 3 and STAR Methods. Diamonds and circles show the rate of LoF variants relative to synonymous variants inside and outside ROH, respectively, for each individual genome. Middle dashed lines represent means (Welch's two-sample t-test; *P<0.05; ***P<0.001). There was a significantly lower number of LoF alleles inside ROH compared to heterozygous parts of individual genomes in both populations. However, this difference was almost 3-fold smaller in the Stewart Island population compared to the mainland population, suggesting that repeated inbreeding events may have facilitated the removal of a significant proportion of severely deleterious and recessive LoF alleles through exposure in homozygous state from the Stewart island population.

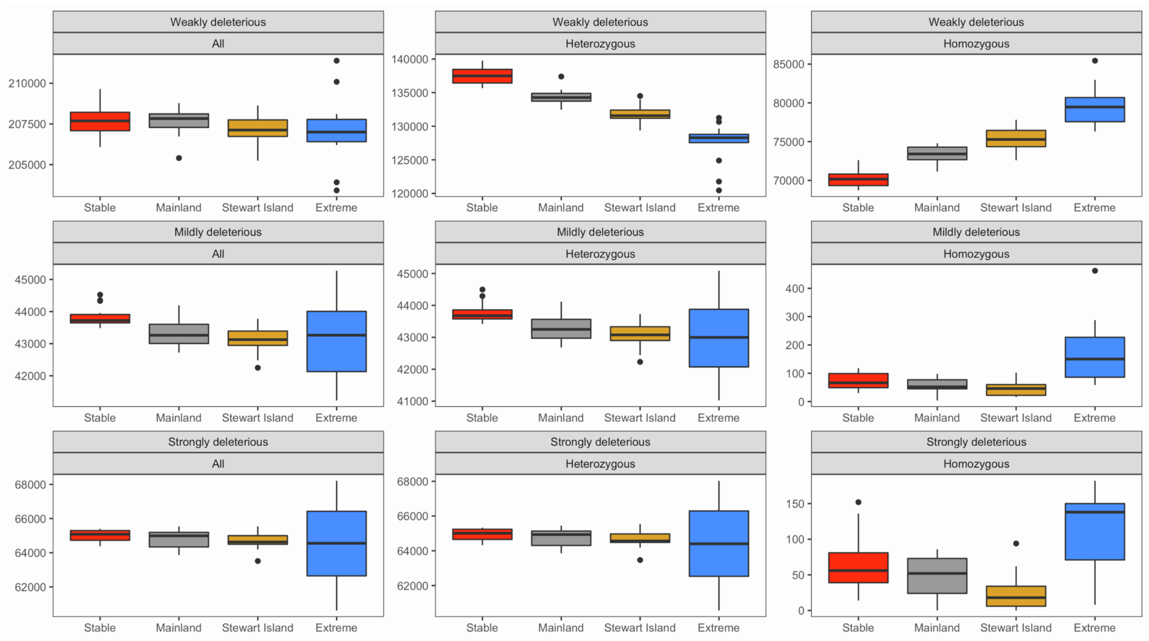

Figure S23. **Counts of derived alleles in the forward simulations with fully recessive dominance coefficients, Related to Figure 4 and STAR Methods.** Allelic counts of all, heterozygous and homozygous for weakly deleterious (-0.001≤ s < 0), mildly deleterious (-0.01 ≤ s < -0.001) and strongly deleterious (s < -0.01) mutations.

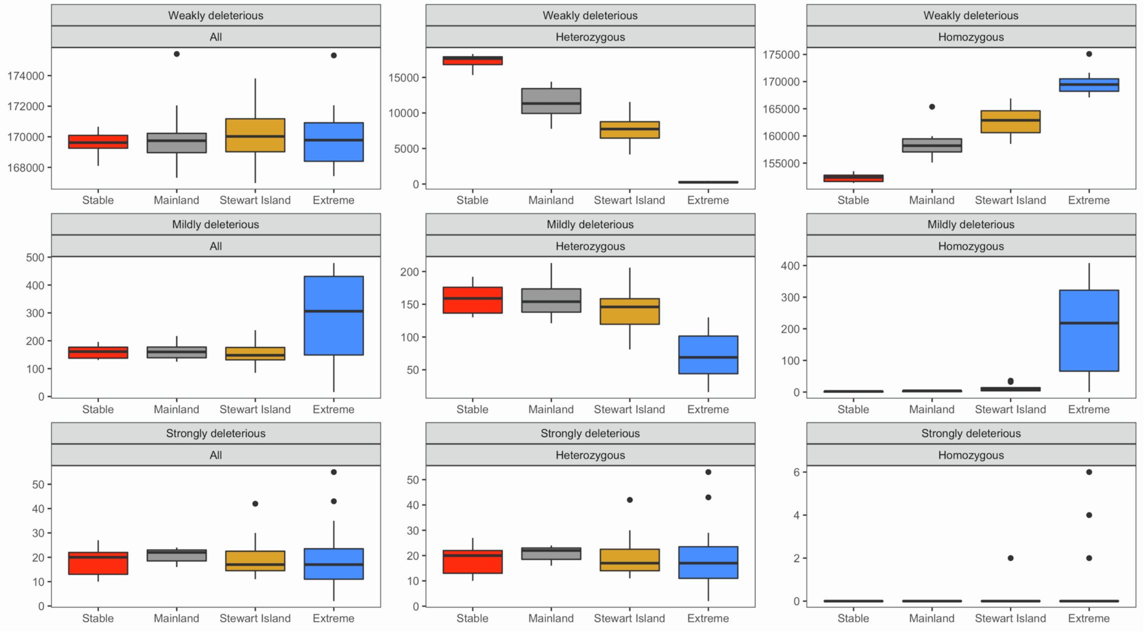

Figure S24. **Counts of derived alleles in the forward simulations with partially recessive dominance coefficients, Related to Figure 4 and STAR Methods.** Allelic counts of all, heterozygous and homozygous for weakly deleterious (-0.001≤ s < 0), mildly deleterious (-0.01 ≤ s < -0.001) and strongly deleterious (s < -0.01) mutations.

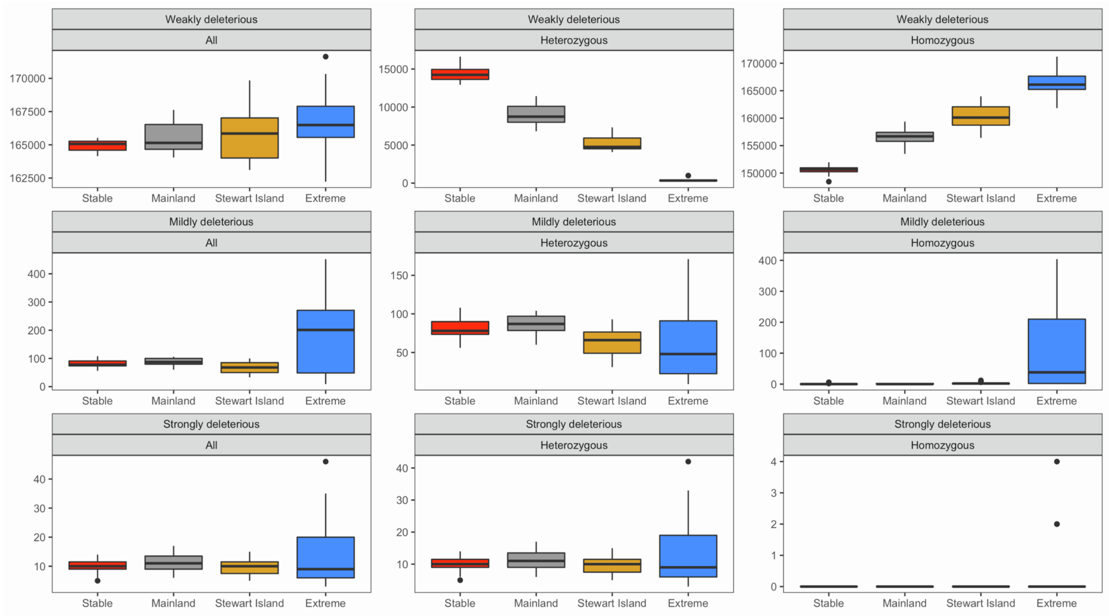

Figure S25. **Counts of derived alleles in the forward simulations with additive dominance coefficients, Related to Figure 4 and STAR Methods.** Allelic counts of all, heterozygous and homozygous for weakly deleterious (-0.001≤ s < 0), mildly deleterious (-0.01 ≤ s < -0.001) and strongly deleterious (s < -0.01) mutations.

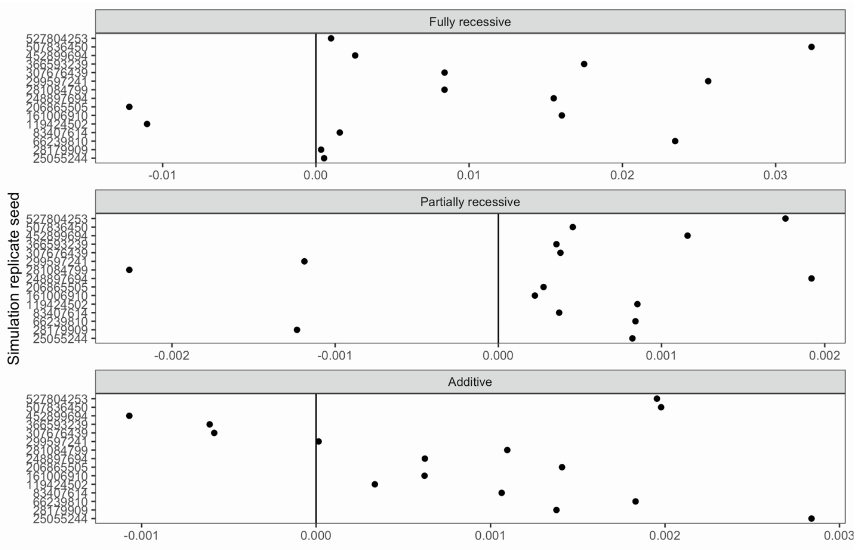

Figure S26. **Additive genetic load difference between simulations under the Stewart Island and the mainland scenarios per replicate, Related to Figure 4 and STAR Methods.** Values above zero indicate replicates where genetic load was lower in the Stewart Island population compared to the mainland population. Not all simulations replicates showed the same pattern, suggesting that this pattern is unlikely to be universal due to the randomness nature of evolutionary dynamics and historical contingencies. Moreover, variable dominance coefficients act simultaneously and partially recessive mutations are expected to disproportionately contribute to inbreeding depression and thus have an impact on purging effects[62]. A wider exploration of different demographic (e.g., size and duration of the founder and bottleneck events), genetic (e.g., range of selection and dominance effects) and life-history (e.g., levels of inbreeding and mating-types) comparisons with simulations and empirical data is needed to investigate the balance between purging and drift-load during evolutionary history (see[63,64]).

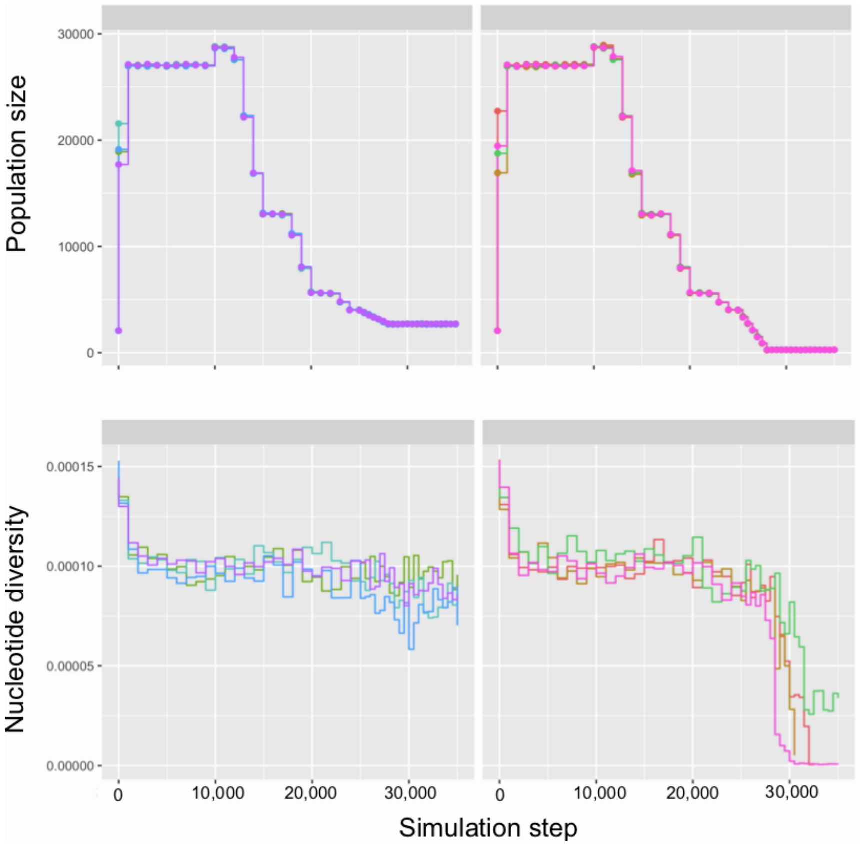

Figure S27. **Effect of scaling during forward simulations, Related to Figure 4 and STAR Methods.** Simulations were scaled to speed-up burn-in period as recommended in the SLiM manual. After burn-in the scaling factor was removed. Here, we show how nucleotide diversity is not impacted by removing the scaling factor (i.e., the mutation-selection equilibrium is maintained) during a period of 10,000 steps (steps 0-10,000) immediately before when we started to vary the population size according to demographic scenarios (steps 10,000-35,000 = 25,000 steps/years).

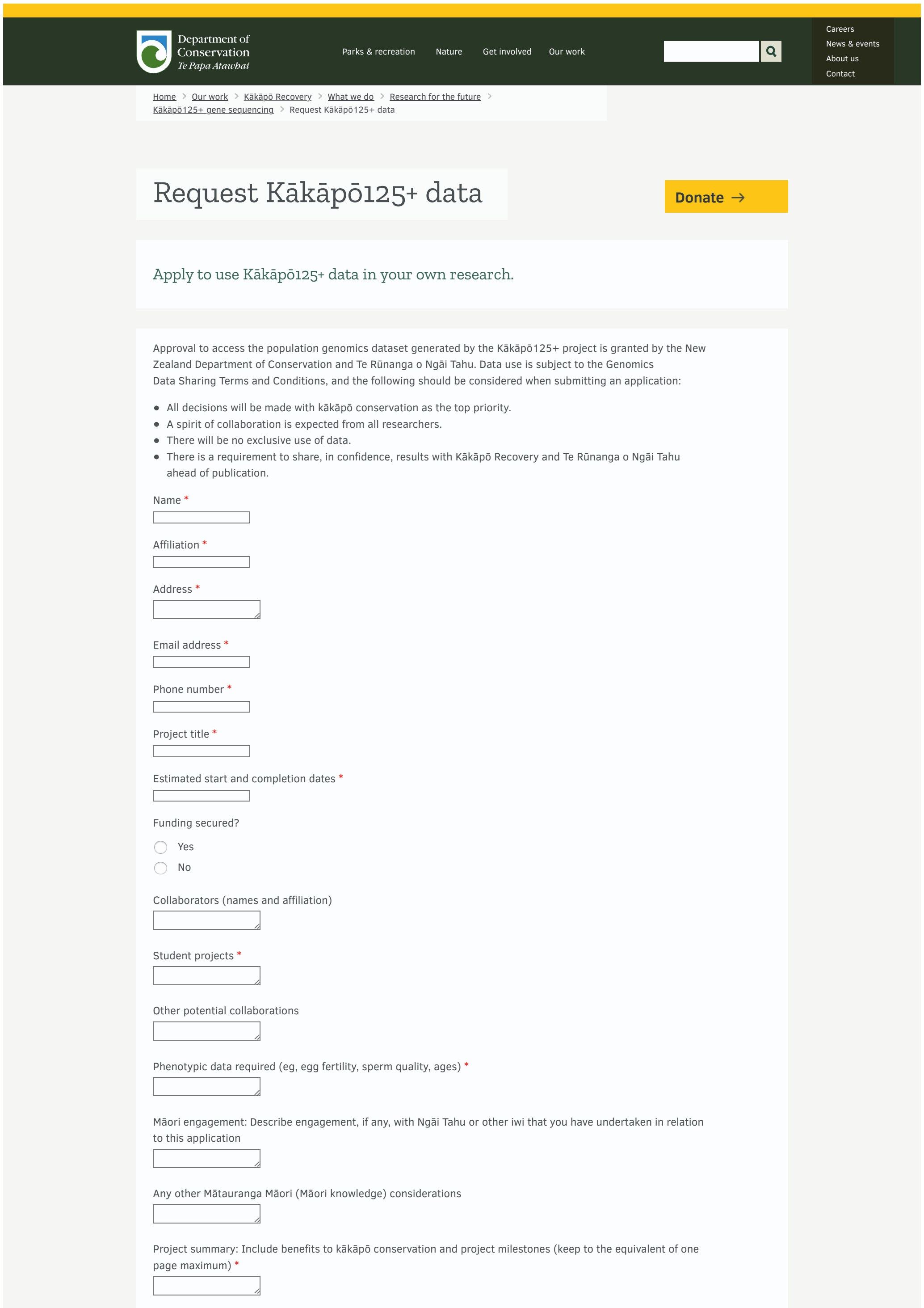

Department of Conservation
Te Papa Atawbai

Parks & recreation | Nature | Get involved | Our work

Careers
News & events
About us
Contact

Home > Our work > Kākāpō Recovery > What we do > Research for the future > Kākāpō125+ gene sequencing > Request Kākāpō125+ data

Request Kākāpō125+ data

Donate →

Apply to use Kākāpō125+ data in your own research.

Approval to access the population genomics dataset generated by the Kākāpō125+ project is granted by the New Zealand Department of Conservation and Te Rūnanga o Ngāi Tahu. Data use is subject to the Genomics Data Sharing Terms and Conditions, and the following should be considered when submitting an application:

- All decisions will be made with kākāpō conservation as the top priority.
- A spirit of collaboration is expected from all researchers.
- There will be no exclusive use of data.
- There is a requirement to share, in confidence, results with Kākāpō Recovery and Te Rūnanga o Ngāi Tahu ahead of publication.

Name *

Affiliation *

Address *

Email address *

Phone number *

Project title *

Estimated start and completion dates *

Funding secured?

- Yes
- No

Collaborators (names and affiliation)

Student projects *

Other potential collaborations

Phenotypic data required (eg, egg fertility, sperm quality, ages) *

Māori engagement: Describe engagement, if any, with Ngāi Tahu or other iwi that you have undertaken in relation to this application

Any other Mātauranga Māori (Māori knowledge) considerations

Project summary: Include benefits to kākāpō conservation and project milestones (keep to the equivalent of one page maximum) *

Figure S28. Request form for kākāpō125+ modern genomic data, Related to STAR Methods. The online form can be found at: https://www.doc.govt.nz/our-work/kakapo-recovery/what-we-do/research-for-the-future/kakapo125-gene-sequencing/request-kakapo125-data/.

Kākāpō125+ genomics data sharing terms and conditions

Parties

DIRECTOR-GENERAL of the New Zealand Department of Conservation Te Papa Atawhai ("the Director-General"). The Department of Conservation is the central government organisation charged with conserving the natural and historic heritage of New Zealand on behalf of and for the benefit of present and future New Zealanders.
Te Rūnanga o Ngāi Tahu, the governance entity of the Ngāi Tahu iwi and statutory representative of Ngāi Tahu Whānui (the collective of individuals who descend from the primary hapū of Waitaha, Ngāti Mamoe, and Ngāi Tahu, namely Kāti Kurī, Kāti Irakehu, Kāti Huirapa, Ngāi Tūāhuriri, and Kai Te Ruahikihiki)
The User

Background

Te Rūnanga and the Director-General will provide the User access to the Data on the condition that the User agrees to these Terms and Conditions.

Definitions

Data means the genomic sequences generated by the Kākāpō125+ genomics project, which include all sequences except for the first (reference) genome, from the kākāpō "Jane", which was sequenced separately in 2015.
DOC means the New Zealand Department of Conservation.
Te Rūnanga means Te Rūnanga o Ngāi Tahu.
Mātauranga Māori means engaging in traditional or present-day knowledge of Ngāi Tahu.
Director-General means the Director-General of Conservation or their delegate.
User means a researcher or research group granted access to the Data.
The singular includes the plural.
Kākāpō Recovery Team means the Operations Manager, Science Advisor and Technical Advisor of the kākāpō/takahē operations team, Southern South Island region, Department of Conservation.

The User agrees and acknowledges:

1. The User must not pass the Data on to a third party, except for those parts of the Data which are deposited in public databases pursuant to the requirements of any journals in which the results are published. (It is expected that the Kākāpō125+ project database will suffice for data access for journal publication requirements. In exceptional cases where this is not sufficient, genes used in publications may be deposited by the author(s) in public genetic databases, such as Genbank, in accord with the requirements of the journal in which the manuscript is published).
2. The User must only use the Data for the purposes outlined in the User's proposal to the Director General and Te Rūnanga.
3. The User must not use the Data or any results arising from them for unauthorised commercial gain.
4. The User agrees to engage with Te Rūnanga, if invited, to understand and consider any Mātauranga Māori aspects to the research and accepts that Ngāi Tahu retain the ownership of intellectual property rights over Mātauranga.
5. A publication embargo applies to use of the Data. Users must refer to the embargo terms advertised on the Kākāpō125+ web page.
6. The User agrees to share, in confidence, all results with the Kākāpō Recovery team ahead of publication.
7. The User agrees to share, in confidence, all results with Te Rūnanga ahead of publication, seeking advice from Te Rūnanga about the best way of doing so.
8. The User must provide Te Rūnanga and the Director-General with a 1-page written summary of the User's results upon completion of their study, and copies of all research reports and publications that use the Data.
9. Te Rūnanga and The Director-General will not disclose results from the User to a third party without the User's permission.
10. The User must, in any publications arising from the Data, acknowledge the contribution of the following parties in the generation of the Data:
    a. New Zealand Department of Conservation;
    b. Te Rūnanga o Ngāi Tahu;
    c. The Genetic Rescue Foundation;
    d. Genomics Aotearoa;
    e. The University of Otago, New Zealand;
    f. New Zealand Genomics Ltd;
    g. Duke University, USA;
    h. Science Exchange;
    i. Experiment.com
11. The Director-General and Te Rūnanga are to be advised and invited to any presentations of the research so they can assess the way it is represented and received by others.

I agree to the Kākāpō125+ genomics data sharing terms and conditions

☐ Yes

☐ I'm not a robot reCAPTCHA Privacy - Terms

Submit

Privacy disclosure: To process your request, we need to collect personal information about you. We'll only use your information for this purpose and we'll follow the principles of the Privacy Act 2020. See our privacy and security statement.

Use a modern browser: You might have problems on older browsers like Internet Explorer. Make sure you use a modern browser such as Chrome or Safari, so this form works properly.

Figure S28. (cont.) Request form for kākāpō125+ modern genomic data, Related to STAR Methods. The online form can be found at: https://www.doc.govt.nz/our-work/kakapo-recovery/what-we-do/research-for-the-future/kakapo125-gene-sequencing/request-kakapo125-data/.

References

1. Sambrook, J., Fritsch, E.F., and Maniatis, T. (1989). Molecular cloning: A laboratory manual, 2nd edn (Cold Spring Harbor Laboratory Press).
2. Dussex, N., von Seth, J., Robertson, B.C., and Dalén, L. (2018). Full mitogenomes in the critically endangered kākāpō reveal major post-glacial and anthropogenic effects on neutral genetic diversity. Genes. *9*, 1–14.
3. Knapp, M., Clarke, A.C., Horsburgh, K.A., and Matisoo-Smith, E.A. (2012). Setting the stage - Building and working in an ancient DNA laboratory. Ann. Anat. *194*, 3–6.
4. Rhie, A., McCarthy, S.A., Fedrigo, O., Damas, J., Formenti, G., Koren, S., Uliano-Silva, M., Chow, W., Fungtammasan, A., Gedman, G.L., et al. (2021). Towards complete and error-free genome assemblies of all vertebrate species. Nature *592*, 737–746.
5. Chin, C.S., Peluso, P., Sedlazeck, F.J., Nattestad, M., Concepcion, G.T., Clum, A., Dunn, C., O'Malley, R., Figueroa-Balderas, R., Morales-Cruz, A., et al. (2016). Phased diploid genome assembly with single-molecule real-time sequencing. Nat. Methods *13*, 1050–1054.
6. Lam, H.Y.K., Ball, M.P., Nielsen, C.B., Pan, C., Thakuria, J. V, Younesy, H., Clark, M.J., Zaranek, A.W., O'Geen, H., Lacroute, P., et al. (2012). Detecting and annotating genetic variations using the HugeSeq pipeline. Nat. Biotechnol. *30*, 226–229.
7. Ghurye, J., Rhie, A., Walenz, B.P., Schmitt, A., Selvaraj, S., Pop, M., Phillippy, A.M., and Koren, S. (2019). Integrating Hi-C links with assembly graphs for chromosome-scale assembly. PLoS Comput. Biol. *15*, 1–19.
8. Bishara, A., Liu, Y., Weng, Z., Kashef-Haghighi, D., Newburger, D.E., West, R., Sidow, A., and Batzoglou, S. (2015). Read clouds uncover variation in complex regions of the human genome. Genome Res. *25*, 1570–1580.
9. Garrison, E., and Marth, G. (2012). Haplotype-based variant detection from short-read sequencing. arXiv, 1207.3907 [q-bio].
10. Li, H. (2011). A statistical framework for SNP calling, mutation discovery, association mapping and population genetical parameter estimation from sequencing data. Bioinformatics *27*, 2987–2993.
11. Roach, M.J., Schmidt, S.A., and Borneman, A.R. (2018). Purge Haplotigs: Allelic contig reassignment for third-gen diploid genome assemblies. BMC Bioinformatics *19*, 1–10.

12. Chow, W., Brugger, K., Caccamo, M., Sealy, I., Torrance, J., and Howe, K. (2016). GEVAL - A web-based browser for evaluating genome assemblies. Bioinformatics *32*, 2508–2510.
13. Durand, N.C., Robinson, J.T., Shamim, M.S., Machol, I., Mesirov, J.P., Lander, E.S., and Aiden, E.L. (2016). Juicebox Provides a Visualization System for Hi-C Contact Maps with Unlimited Zoom. Cell Syst. *3*, 99–101.
14. Camacho, C., Coulouris, G., Avagyan, V., Ma, N., Papadopoulos, J., Bealer, K., and Madden, T.L. (2009). BLAST + : architecture and applications. BMC Bioinformatics *10*, 421.
15. Okonechnikov, K., Conesa, A., and García-Alcalde, F. (2015). Qualimap 2: Advanced multi-sample quality control for high-throughput sequencing data. Bioinformatics *32*, 292–294.
16. Smit, A.F.A., Hubley, R., and Green, P. (2010). RepeatMasker Open-3.0. 1996-2010. Inst. Syst. Biol.
17. Jain, C., Koren, S., Dilthey, A., Phillippy, A.M., and Aluru, S. (2018). A fast adaptive algorithm for computing whole-genome homology maps. Bioinformatics *34*, i748–i756.
18. Chaisson, M.J., and Tesler, G. (2012). Mapping single molecule sequencing reads using basic local alignment with successive refinement (BLASR): application and theory. BMC Bioinformatics *13*, 238.
19. Salzberg, S.L., and Langmead, B. (2012). Fast gapped-read alignment with Bowtie 2. Nat. Methods *9*, 357–359.
20. Neethiraj, R., Hornett, E.A., Hill, J.A., and Wheat, C.W. (2017). Investigating the genomic basis of discrete phenotypes using a Pool-Seq-only approach: New insights into the genetics underlying colour variation in diverse taxa. Mol. Ecol. *26*, 4990–5002.
21. Hoff, K.J., Lange, S., Lomsadze, A., Borodovsky, M., and Stanke, M. (2016). BRAKER1: Unsupervised RNA-Seq-based genome annotation with GeneMark-ET and AUGUSTUS. Bioinformatics *32*, 767–769.
22. Stanke, M., Diekhans, M., and Robert Baertsch, D.H. (2008). Using native and syntenically mapped cDNA alignments to improve de novo gene finding. Bioinformatics *24*, 637–644.
23. Stanke, M., Schöffmann, O., Morgenstern, B., and Waack, S. (2006). Gene prediction in

eukaryotes with a generalized hidden Markov model that uses hints from external sources. BMC Bioinformatics *7*.

24. Lomsadze, A., Ter-Hovhannisyan, V., Chernoff, Y.O., and Borodovsky, M. (2005). Gene identification in novel eukaryotic genomes by self-training algorithm. Nucleic Acids Res. *33*, 6494–6506.
25. Ter-Hovhannisyan, V., Lomsadze, A., Chernoff, Y.O., and Borodovsky, M. (2008). Gene prediction in novel fungal genomes using an ab initio algorithm with unsupervised training. Genome Res. *18*, 1979–1990.
26. Stanke, M., Keller, O., Gunduz, I., Hayes, A., Waack, S., and Morgenstern, B. (2006). AUGUSTUS: Ab initio prediction of alternative transcripts. Nucleic Acids Res. *34*, 435–439.
27. Camacho, C., Coulouris, G., Avagyan, V., Ma, N., Papadopoulos, J., Bealer, K., and Madden, T.L. (2009). BLAST+: Architecture and applications. BMC Bioinformatics *10*, 421.
28. Altschul, A.F., Gish, W., Miller, W., Myers, E.W. and Lipman, D.J. (1990). Basic Local Alignment Search Tool. Mol. Biol. *215*, 403–410.
29. Warren, W.C., Clayton, D.F., Ellegren, H., Arnold, A.P., Hillier, L.W., Künstner, A., Searle, S., White, S., Vilella, A.J., Fairley, S., et al. (2010). The genome of a songbird. Nature *464*, 757–762.
30. Ellegren, H., Smeds, L., Burri, R., Olason, P.I., Backström, N., Kawakami, T., Künstner, A., Mäkinen, H., Nadachowska-Brzyska, K., Qvarnström, A., et al. (2012). The genomic landscape of species divergence in Ficedula flycatchers. Nature *491*, 756–760.
31. Iwata, H., and Gotoh, O. (2012). Benchmarking spliced alignment programs including Spaln2, an extended version of Spaln that incorporates additional species-specific features. Nucleic Acids Res. *40*, 1–9.
32. Girgis, H.Z. (2015). Red: An intelligent, rapid, accurate tool for detecting repeats de-novo on the genomic scale. BMC Bioinformatics *16*.
33. Gremme, G., Brendel, V., Sparks, M.E., and Kurtz, S. (2005). Engineering a software tool for gene structure prediction in higher organisms. Inf. Softw. Technol. *47*, 965–978.
34. Trapnell, C., Roberts, A., Goff, L., Pertea, G., Kim, D., Kelley, D.R., Pimentel, H., Salzberg, S.L., Rinn, J.L., and Pachter, L. (2012). Differential gene and transcript

expression analysis of RNA-seq experiments with TopHat and Cufflinks. Nat. Protoc. *7*, 562–78.

35. Roberts, A., Trapnell, C., Donaghey, J., Rinn, J.L., and Pachter, L. (2011). Improving RNA-Seq expression estimates by correcting for fragment bias. Genome Biol. *12*.

36. Huerta-Cepas, J., Szklarczyk, D., Forslund, K., Cook, H., Heller, D., Walter, M.C., Rattei, T., Mende, D.R., Sunagawa, S., Kuhn, M., et al. (2016). EGGNOG 4.5: A hierarchical orthology framework with improved functional annotations for eukaryotic, prokaryotic and viral sequences. Nucleic Acids Res. *44*, D286–D293.

37. Aken, B.L., Ayling, S., Barrell, D., Clarke, L., Curwen, V., Fairley, S., Fernandez Banet, J., Billis, K., García Girón, C., Hourlier, T., et al. (2016). The Ensembl gene annotation system. Database (Oxford). *2016*.

38. The UniProt Consortium (2019). UniProt: A worldwide hub of protein knowledge. Nucleic Acids Res. *47*, D506–D515.

39. Li, H. (2018). Minimap2: Pairwise alignment for nucleotide sequences. Bioinformatics *34*, 3094–3100.

40. Li, H., and Durbin, R. (2010). Fast and accurate long-read alignment with Burrows-Wheeler transform. Bioinformatics *26*, 589–595.

41. Slater, G.S.C., and Birney, E. (2005). Automated generation of heuristics for biological sequence comparison. BMC Bioinformatics *6*.

42. She, R., Chu, J.S.C., Uyar, B., Wang, J., Wang, K., and Chen, N. (2011). genBlastG: Using BLAST searches to build homologous gene models. Bioinformatics *27*, 2141–2143.

43. Harris, R.S. (2007). Improved pairwise alignment of genomic DNA.

44. Sharma, V., Schwede, P., and Hiller, M. (2017). CESAR 2.0 substantially improves speed and accuracy of comparative gene annotation. Bioinformatics *33*, 3985–3987.

45. Kozomara, A., Birgaoanu, M., and Griffiths-Jones, S. (2019). MiRBase: From microRNA sequences to function. Nucleic Acids Res. *47*, D155–D162.

46. Kalvari, I., Argasinska, J., Quinones-Olvera, N., Nawrocki, E.P., Rivas, E., Eddy, S.R., Bateman, A., Finn, R.D., and Petrov, A.I. (2018). Rfam 13.0: Shifting to a genome-centric resource for non-coding RNA families. Nucleic Acids Res. *46*, D335–D342.

47. Pruitt, K.D., Brown, G.R., Hiatt, S.M., Thibaud-Nissen, F., Astashyn, A., Ermolaeva, O., Farrell, C.M., Hart, J., Landrum, M.J., McGarvey, K.M., et al. (2014). RefSeq: An update

on mammalian reference sequences. Nucleic Acids Res. *42*.
48. Morgulis, A., Gertz, E.M., Schäffer, A.A., and Agarwala, R. (2006). WindowMasker: Window-based masker for sequenced genomes. Bioinformatics *22*, 134–141.
49. Altschul, S.F., Gish, W.R., Miller, W., Myers, E.W., and Lipman, D.J. (1990). Basic local alignment search tool. J. Mol. Biol. *215*, 403–410.
50. Kapustin, Y., Souvorov, A., Tatusova, T., and Lipman, D. (2008). Splign: Algorithms for computing spliced alignments with identification of paralogs. Biol. Direct *3*.
51. Lowe, T.M., and Eddy, S.R. (1996). TRNAscan-SE: A program for improved detection of transfer RNA genes in genomic sequence. Nucleic Acids Res. *25*, 955–964.
52. Nawrocki, E.P., Burge, S.W., Bateman, A., Daub, J., Eberhardt, R.Y., Eddy, S.R., Floden, E.W., Gardner, P.P., Jones, T.A., Tate, J., et al. (2015). Rfam 12.0: Updates to the RNA families database. Nucleic Acids Res. *43*, D130–D137.
53. Elliott, G P (Department of Conservation, Nelson, N.Z. (2016). Personal communication.
54. Kerpedjiev, P., Abdennur, N., Lekschas, F., McCallum, C., Dinkla, K., Strobelt, H., Luber, J.M., Ouellette, S.B., Azhir, A., Kumar, N., et al. (2018). HiGlass: Web-based visual exploration and analysis of genome interaction maps. Genome Biol. *19*.
55. Schiffels, S., and Wang, K. (2020). MSMC and MSMC2: The multiple sequentially Markovian coalescent. Methods Mol. Biol. *2090*, 147–166.
56. Murray, G.G.R., Soares, A.E.R., Novak, B.J., Schaefer, N.K., Cahill, J.A., Baker, A.J., Demboski, J.R., Doll, A., Da Fonseca, R.R., Fulton, T.L., et al. (2017). Natural selection shaped the rise and fall of passenger pigeon genomic diversity. Science. *358*, 951–954.
57. Jarvis, E.D., Mirarab, S., Aberer, A.J., Li, B., Houde, P., Li, C., Ho, S.Y.W., Faircloth, B.C., Nabholz, B., Howard, J.T., et al. (2014). Whole-genome analyses resolve early branches in the tree of life of modern birds. Science. *346*, 1320–1331.
58. Weber, C.C., Boussau, B., Romiguier, J., Jarvis, E.D., and Ellegren, H. (2014). Evidence for GC-biased gene conversion as a driver of between-lineage differences in avian base composition. Genome Biol. *15*, 1–16.
59. Hedges, S.B., Marin, J., Suleski, M., Paymer, M., and Kumar, S. (2015). Tree of life reveals clock-like speciation and diversification. Mol. Biol. Evol. *32*, 835–845.
60. Valk, T. van der, Manuel, M. de, Marques-Bonet, T., and Guschanski, K. (2019). Estimates of genetic load in small populations suggest extensive purging of deleterious

alleles. bioRxiv, 696831.

61. Hedrick, P.W., and Garcia-Dorado, A. (2016). Understanding Inbreeding Depression, Purging, and Genetic Rescue. Trends Ecol. Evol. *31*, 940–952.

62. Wang, J., Hill, W.G., Charlesworth, D., and Charlesworth, B. (1999). Dynamics of inbreeding depression due to deleterious mutations in small populations: Mutation parameters and inbreeding rate. Genet. Res. *74*, 165–178.

63. Glémin, S. (2003). How are deleterious mutations purged? Drift versus nonrandom mating. Evolution (N. Y). *57*, 2678–87.

64. García-Dorado, A. (2012). Understanding and predicting the fitness decline of shrunk populations: Inbreeding, purging, mutation, and standard selection. Genetics *190*, 1461–1476.
