# Supplementary material for: Population genomics of the critically endangered kākāpō
Source: Cell Genom. 2021 Sep 8;1(1):100002. doi: 10.1016/j.xgen.2021.100002 (PMC9903828; doi:10.1016/j.xgen.2021.100002)
Supplement: Document S2. Article plus supplemental information [file mmc7.pdf]

Cell Genomics

Short article

Population genomics of the critically endangered kākāpō

Graphical abstract

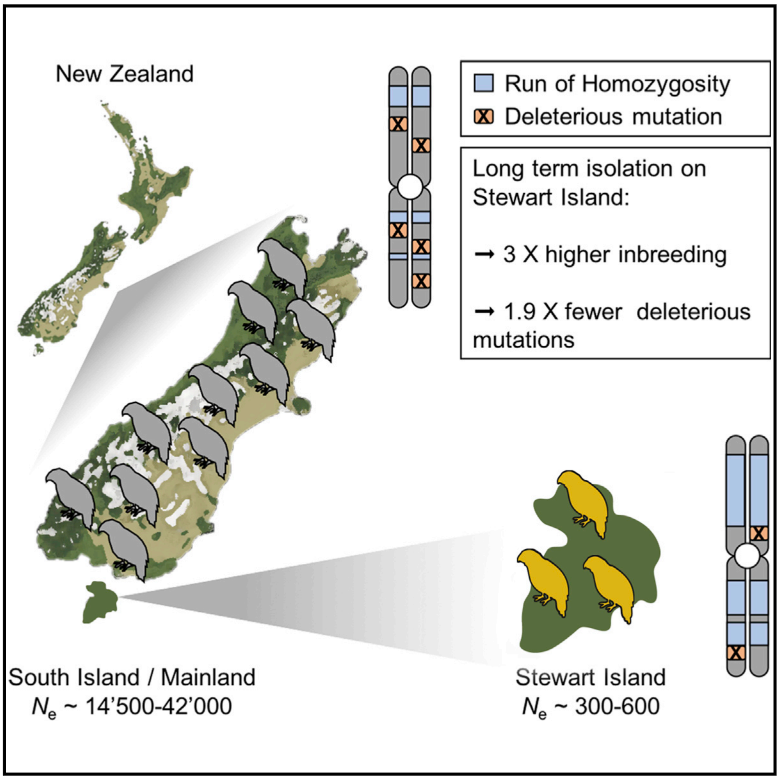

Highlights

- First genome sequencing and population genomic analyses of the critically endangered kākāpō
- Generation of a high-quality annotated reference genome assembly for kākāpō
- Sequencing of 49 kākāpō genomes from surviving island and extinct mainland populations
- Genomic analyses of long-term isolated populations inform conservation strategies

Authors

Nicolas Dussex, Tom van der Valk, Hernán E. Morales, ..., Erich D. Jarvis, Bruce C. Robertson, Love Dalén

Correspondence

nicolas.dussex@gmail.com (N.D.),
ejarvis@mail.rockefeller.edu (E.D.J.),
bruce.robertson@otago.ac.nz (B.C.R.),
love.dalen@nrm.se (L.D.)

In brief

Dussex et al. sequence modern and historical genomes from the critically endangered kākāpō to investigate the genetic consequences of long-term small population size. They find that the remaining kākāpō from a small island population are inbred but have fewer deleterious mutations compared to the now-extinct mainland population, providing insights into conservation strategies.

Dussex et al., 2021, Cell Genomics *1*, 100002
October 13, 2021 © 2021 The Author(s).
https://doi.org/10.1016/j.xgen.2021.100002

CellPress

Cell Genomics

CellPress
OPEN ACCESS

Short article

Population genomics of the critically endangered kākāpō

Nicolas Dussex,[1,2,3,4,24,*] Tom van der Valk,[1,2] Hernán E. Morales,[5] Christopher W. Wheat,[3] David Díez-del-Molino,[1,2] Johanna von Seth,[1,2,3] Yasmin Foster,[6] Verena E. Kutschera,[7] Katerina Guschanski,[8,9] Arang Rhie,[10] Adam M. Phillippy,[10] Jonas Korlach,[11] Kerstin Howe,[12] William Chow,[12] Sarah Pelan,[12] Joanna D. Mendes Damas,[13] Harris A. Lewin,[13] Alex R. Hastie,[14] Giulio Formenti,[15,16,17] Olivier Fedrigo,[15] Joseph Guhlin,[18] Thomas W.R. Harrop,[18] Marissa F. Le Lec,[18]

(Author list continued on next page)

[1]Centre for Palaeogenetics, Svante Arrhenius väg 20C, 10691 Stockholm, Sweden
[2]Department of Bioinformatics and Genetics, Swedish Museum of Natural History, Box 50007, 10405 Stockholm, Sweden
[3]Department of Zoology, Stockholm University, 10691 Stockholm, Sweden
[4]Department of Anatomy, University of Otago, PO Box 913, Dunedin 9016, New Zealand
[5]Section for Evolutionary Genomics, GLOBE Institute, University of Copenhagen, Copenhagen, Denmark
[6]Department of Zoology, University of Otago, PO Box 56, Dunedin 9054, New Zealand
[7]Department of Biochemistry and Biophysics, National Bioinformatics Infrastructure Sweden, Science for Life Laboratory, Stockholm University, Box 1031, 17121 Solna, Sweden
[8]Institute of Evolutionary Biology, School of Biological Sciences, University of Edinburgh, Edinburgh, UK
[9]Department of Ecology and Genetics, Animal Ecology, Uppsala University, 75236 Uppsala, Sweden
[10]Genome Informatics Section, Computational and Statistical Genomics Branch, National Human Genome Research Institute, National Institutes of Health, Bethesda, MD 20892, USA
[11]Pacific Biosciences, 1305 O'Brien Drive, Menlo Park, CA 94025, USA
[12]Wellcome Sanger Institute, Wellcome Trust Genome Campus, Hinxton CB10 1SA, UK
[13]Department of Evolution and Ecology and the UC Davis Genome Center, 4321 Genome and Biomedical Sciences Facility, University of California Davis, Davis, CA 95616, USA
[14]Bionano Genomics, 9540 Towne Centre Drive, San Diego, CA 92121, USA
[15]Vertebrate Genome Laboratory, The Rockefeller University, New York, NY 10065, USA
[16]Laboratory of Neurogenetics of Language, Box 54, The Rockefeller University, New York, NY 10065, USA

(Affiliations continued on next page)

SUMMARY

The kākāpō is a flightless parrot endemic to New Zealand. Once common in the archipelago, only 201 individuals remain today, most of them descending from an isolated island population. We report the first genome-wide analyses of the species, including a high-quality genome assembly for kākāpō, one of the first chromosome-level reference genomes sequenced by the Vertebrate Genomes Project (VGP). We also sequenced and analyzed 35 modern genomes from the sole surviving island population and 14 genomes from the extinct mainland population. While theory suggests that such a small population is likely to have accumulated deleterious mutations through genetic drift, our analyses on the impact of the long-term small population size in kākāpō indicate that present-day island kākāpō have a reduced number of harmful mutations compared to mainland individuals. We hypothesize that this reduced mutational load is due to the island population having been subjected to a combination of genetic drift and purging of deleterious mutations, through increased inbreeding and purifying selection, since its isolation from the mainland ~10,000 years ago. Our results provide evidence that small populations can survive even when isolated for hundreds of generations. This work provides key insights into kākāpō breeding and recovery and more generally into the application of genetic tools in conservation efforts for endangered species.

INTRODUCTION

New Zealand was one of the last landmasses colonized by humans.[1] Following Polynesian colonization circa 1360 CE and European colonization in the 1800s, and the resulting overhunting and introduction of mammalian predators, New Zealand experienced major extinction events of endemic species.[2] The kākāpō (*Strigops habroptilus*), a flightless parrot species, was

This is an open access article under the CC BY license (http://creativecommons.org/licenses/by/4.0/).

Cell Genomics
Short article

Peter K. Dearden,[18] Leanne Haggerty,[19] Fergal J. Martin,[19] Vamsi Kodali,[20] Françoise Thibaud-Nissen,[20] David Iorns,[21] Michael Knapp,[4] Neil J. Gemmell,[4] Fiona Robertson,[6] Ron Moorhouse,[22] Andrew Digby,[22] Daryl Eason,[22] Deidre Vercoe,[22] Jason Howard,[15,23] Erich D. Jarvis,[15,16,17,*] Bruce C. Robertson,[6,*] and Love Dalén[1,2,3,*]

[17]Howard Hughes Medical Institute, Chevy Chase, MD 20815, USA

[18]Genomics Aotearoa and Laboratory for Evolution and Development, Department of Biochemistry, University of Otago, PO Box 56, Dunedin 9016, New Zealand

[19]European Molecular Biology Laboratory, European Bioinformatics Institute, Wellcome Genome Campus, Hinxton, Cambridge CB10 1SD, UK

[20]National Center for Biotechnology Information, National Library of Medicine, National Institutes of Health, Bethesda, MD 20894, USA

[21]The Genetic Rescue Foundation, Wellington, New Zealand

[22]Kākāpō Recovery, Department of Conservation, PO Box 743, Invercargill 9840, New Zealand

[23]BioSkryb Genomics, 701 W Main Street, Suite 200, Durham, NC 27701, USA

[24]Lead contact

*Correspondence: nicolas.dussex@gmail.com (N.D.), ejarvis@mail.rockefeller.edu (E.D.J.), bruce.robertson@otago.ac.nz (B.C.R.), love.dalen@nrm.se (L.D.)

https://doi.org/10.1016/j.xgen.2021.100002

widespread before human arrival and likely numbered in the hundreds of thousands.[3] By 1995, the species was reduced to 51 birds, 50 kākāpō from the isolated Stewart Island and one single male, named Richard Henry, from the extinct mainland population.[4] Richard Henry and 39 Stewart Island birds were the only kākāpō to reproduce and are thus the ancestors of all birds born since 1995. As of 2021, a total of 201 kākāpō survive and are managed on island sanctuaries. Previous studies indicate that kākāpō have lost ~70%–80% of their genetic diversity since the 1800s and have elevated levels of inbreeding.[3,5] Poor sperm quality and low hatching success[6] suggest that kākāpō carry deleterious mutations as a consequence of genetic drift and reduced efficacy of purifying selection, a clear evidence of reduced fitness (i.e., genetic load[7,8]). However, the genome-wide impact of the severe population bottleneck of the kākāpō remains unknown.

Population genetic theory suggests that, in small populations, genetic load may accumulate and increase the risk of extinction via "mutational meltdown."[8] However, because the effects of observed mutations on fitness are often unknown, estimating genetic load is challenging. Recent studies based on genomic data from temporally spaced samples of extinct and endangered species[9–14] have instead focused on the accumulation of deleterious mutations by examining increases in mutational load. However, theory and empirical data suggest that mutational load can also be purged in long-term isolated and inbred populations when selection against recessive or partially recessive detrimental alleles is increased as they are expressed in the homozygous state.[15] To date, although simulations and comparisons among species or subspecies have shown this to be possible in rare examples (see, for example, Robinson et al.[16] and Grossen et al.[17]), studies on the purging of mutational load in the wild and between recently diverged populations remain scarce.

To test these hypotheses, we compared the genomes of surviving and extinct kākāpō populations, representing the first population genomics analysis of kākāpō in the context of the Kākāpo 125+ project. Here, we present the reference genome for kākāpō, one of the first chromosome-level reference genomes sequenced by the Vertebrate Genomes Project (VGP),[18] and a population genomics analysis of 49 kākāpō from Stewart Island and the extinct mainland population. Our population genomic analyses indicate that present-day island kākāpō have a reduced number of predicted deleterious mutations compared to mainland individuals. We suggest that this reduced mutational load may have resulted from a combination of genetic drift and purging of deleterious mutations through increased inbreeding and purifying selection in the island population since their isolation from the mainland ~10,000 years ago. Our findings will aid in the development of genetic approaches to support the recovery of kākāpō and contribute to informing future breeding programs and translocation efforts. More generally, such population genomic analyses of other endangered species will be useful to inform those conservation efforts.

RESULTS

Island and mainland populations are distinct and separated after the last glaciation

We generated a high-quality chromosome-level genome assembly for a female kākāpō (Figures S1–S3) and sequenced the genomes from 36 individuals that survived the bottleneck at its most severe phase in the 1990s (Richard Henry and 35 Stewart Island birds) as well as 13 genomes from ~130-year-old museum specimens that originated from the extinct mainland population (Figure 1A; Table S1). No offspring from the surviving individuals that subsequently founded the present-day population were included in the analyses. Principal-component analysis (PCA) of the 49 re-sequenced genomes identified genetic distinctions between the mainland and Stewart Island populations (Figures 1B and S5). We found evidence for historical samples' mislabeling and subsequently analyzed samples according to their genetically assigned population when estimating differences in inbreeding and mutational load (STAR Methods).

We performed pairwise sequentially Markovian coalescent (PSMC) analysis to track changes in effective population sizes (N_e) over time (STAR Methods). Analyses of the genomes from the mainland and Stewart Island populations showed nearly identical demographic histories marked by a severe decline in N_e, starting some 30,000 years (30 ka) before present (BP; Figure 1C), a period coinciding with the onset of the Last Glacial Maximum (LGM).[20] By the end of the last glaciation ~10 ka BP, kākāpō N_e had declined from ~15,000–20,000 to a minimum of ~1,000–3,000. Since the PSMC has reduced power to estimate recent changes in N_e (i.e., <10 ka BP[21]), we also performed approximate Bayesian computation (ABC) simulations and demographic reconstructions using MSMC2 (Figures S6–S9),

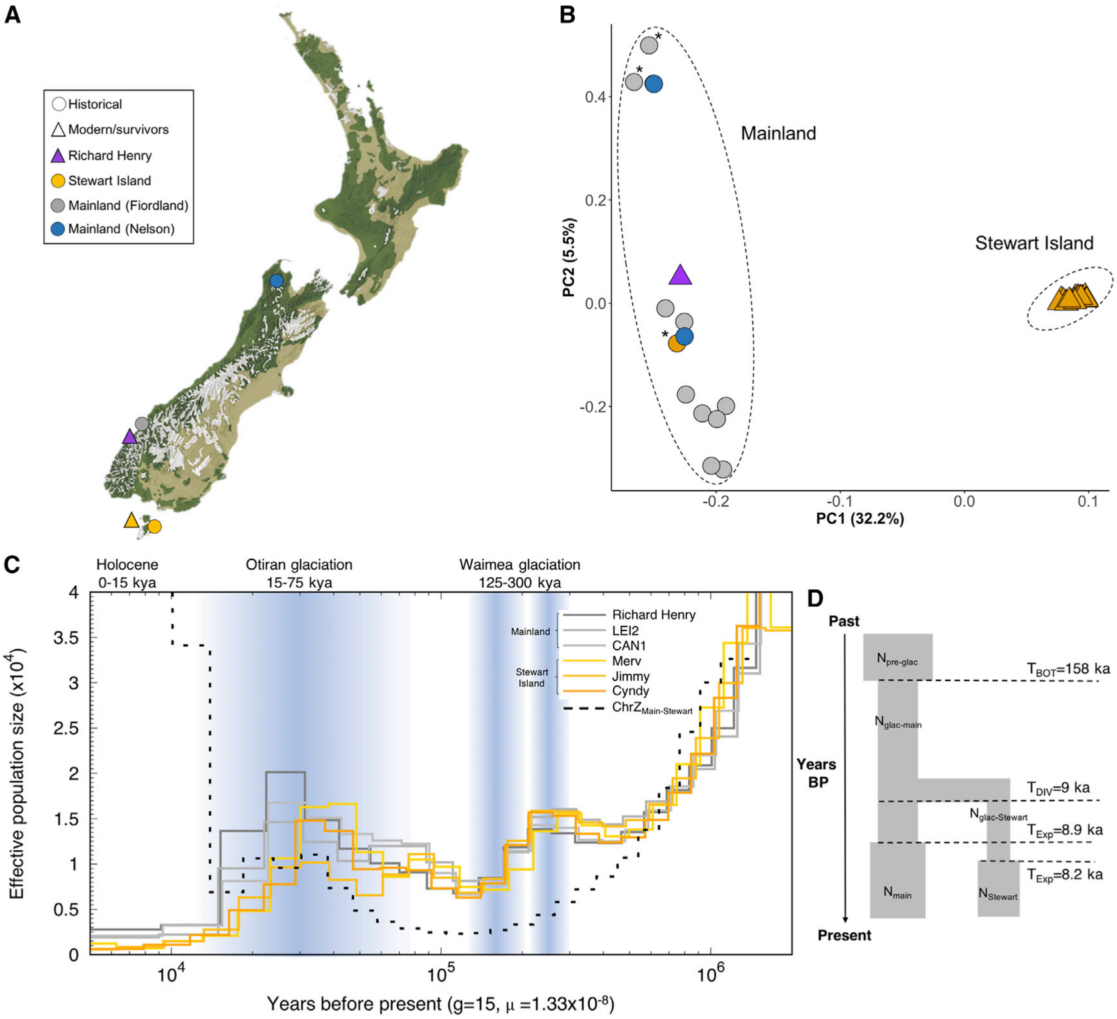

Figure 1. Sampling locations, population structure, and past demography of kākāpō

(A) Sampling locations for historical and modern specimens on a map showing the vegetation cover circa 1840.[19]

(B) Principal-component analysis (PCA) for 14 mainland and 35 Stewart Island kākāpō. Asterisks indicate museum samples likely to have been mislabeled (STAR Methods).

(C) Demographic history and divergence time between the mainland and Stewart Island population inferred using the PSMC method. Each colored curve represents an individual bird. The black dashed curve represents the sex chromosome comparison (i.e., Z chromosome), with population size reaching infinity at the time of divergence between the two populations.

(D) Parameter estimates for a scenario of post-glacial population divergence and expansion using ABC.

See also Figures S5–S10 and Tables S2 and S3.

which are better suited to infer recent demography. These analyses also supported a demographic decline during the LGM (Figure 1D, S8 and S9; Tables S2 and S3). Moreover, Late Holocene N_e estimates from the MSMC2 and ABC were similar, with N_e ~300–600 for Stewart Island and N_e ~14,500–42,000 for the mainland population (Figures S8 and S9; Table S3).

Historical accounts and a lack of fossil remains originally suggested that kākāpō were introduced to Stewart Island in the past ~500 years, by either Māori or European settlers.[4,22] However, based on our ABC simulations and analyses of coalescence rates between Z chromosomes, we find that the divergence time between the mainland and the Stewart Island populations dates back to the end of the last glaciation (Figures 1C, 1D, S8, and S10). This timing coincides with the isolation of Stewart Island from the mainland as sea levels rose at the end of the Pleistocene some 12 ka BP.[23] Thus, our analyses suggest that instead of having been established by humans in the past ~500 years, the Stewart Island population constitutes a distinct lineage that has been isolated from the mainland for up to 1,000 generations.

The island population is highly inbred

The long-term isolation and reduced N_e since the end of the last glaciation and the severe decline in the past 150 years on Stewart Island due to introduced predators[4] may have led to the additional loss of genetic diversity via genetic drift. Supporting this

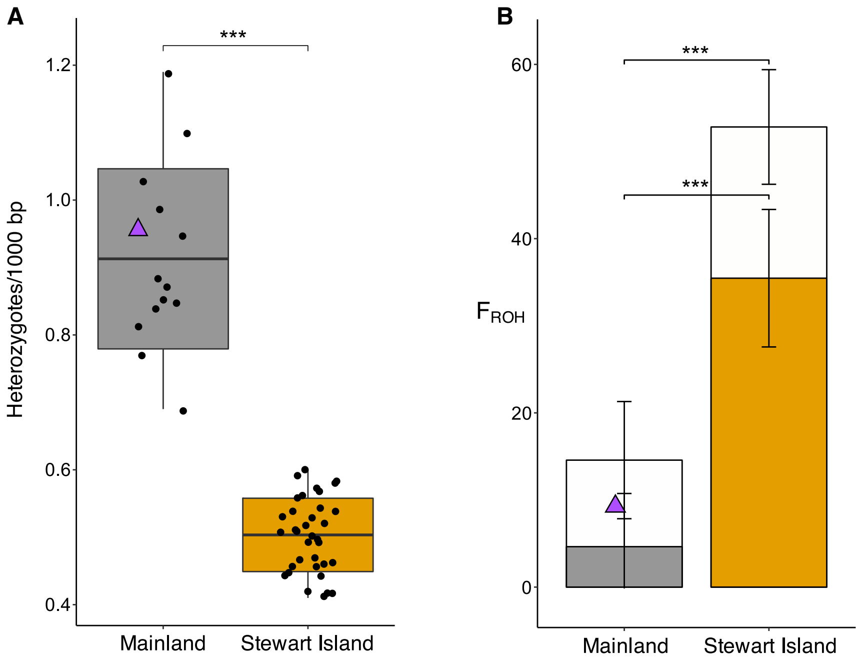

Figure 2. Heterozygosity and inbreeding estimates for kākāpō

(A) Individual genome-wide heterozygosity. Horizontal lines within boxplots and bounds of boxes represent means and standard deviations, respectively. Vertical lines represent minima and maxima.

(B) Individual inbreeding coefficients estimated using ROH (F_{ROH}). Open bars show the total proportion of the genome in ROH ≥ 100 kb and solid bars show proportions in ROH ≥ 2 Mb. Bars extending from the mean values represent the standard deviation (Welch's two-sample t test; ***p < 0.001). Richard Henry is shown with a purple triangle.

See also Figures S11–S15.

hypothesis, we find much lower average individual heterozygosity (Figure 2A) and lower population-level nucleotide diversity (π; Figure S11) in the genomes of the Stewart Island birds. Furthermore, based on a high-quality dataset of variants covered in all individuals, we find that extended runs of homozygosity (F_{ROH}), an expected outcome of inbreeding, also differed markedly between the populations. Stewart Island kākāpō had, on average, 53% of their genome sequence in ROH ≥ 100 kb, while mainland kākāpō had 15% (Figure 2B). Genome proportions comprising very long ROH (≥2 Mb) were, on average, 34% and 4% for Stewart Island and mainland kākāpō, respectively (Figures 2B and S12–S15). Such comparatively long ROH indicate recent mating between closely related individuals, most likely during the past ten generations.[24] Overall, these results are in agreement with the long-term insular isolation at low N_e as well as a recent decline in the past few hundred years.

Genomes from Stewart Island kākāpō have comparatively fewer deleterious mutations

To test the hypotheses of small populations being affected by either increased mutational load due to strong drift causing a reduced efficacy of purifying selection in removing deleterious mutations[8] or purging of recessive deleterious mutations as a consequence of increased inbreeding,[15] we estimated the mutational load in mainland and Stewart Island kākāpō. First, we measured individual mutational load as the number of derived alleles at sites that are under strict evolutionary constraints, predicted as likely to be deleterious using genomic evolutionary rate profiling (GERP) scores (Figures S16 and S17; STAR Methods). These results indicate ~1.1 times lower mutational load in the genomes of Stewart Island compared to mainland kākāpō (Figure 3A). Moreover, the difference in the number of deleterious alleles was most pronounced at sites under the strongest evolutionary constraint (Figures S18 and S19), consistent with the purging of deleterious mutations in the Stewart Island population.

Second, we estimated mutational load in each individual by identifying variants in our annotation of 15,699 coding genes (STAR Methods). Similar to the GERP analysis, we find ~1.9 times fewer mutations classified as highly deleterious (i.e., predicted loss of function [LoF]) in Stewart Island relative to the mainland kākāpō population (Figures 3B, S20, and S21), with, on average, 17.6 and 34.1 predicted LoF variants per bird genome for the Stewart Island and mainland population, respectively (Figure 3B). The ratio of derived alleles (R_{xy}) between the two populations also showed reduced LoF and missense variants in the Stewart Island population compared to the mainland population (Figure 3C). Furthermore, the mainland population had a higher number of LoF alleles in the heterozygous state compared to the Stewart Island population (Figures S20 and S21). This suggests that many of these mutations are primarily deleterious in the homozygous state, consistent with theoretical predictions.[15] We also found a significantly lower number of LoF alleles inside ROH compared to heterozygous parts of the genomes, and this difference was 3-fold smaller in the Stewart Island population (Figure S22), suggesting that repeated inbreeding may have facilitated the removal of heterozygous LoF alleles.[25]

To further test whether our results are consistent with the purging of deleterious mutations, we performed forward simulations recapitulating the demographic history of the mainland and Stewart Island populations (Figure 4A). We also simulated scenarios for hypothetical stable and severely bottlenecked populations to model a weak and strong effect of drift, respectively (STAR Methods). When assuming a scenario consistent with the history of the Stewart Island population, our simulations indicated that the additive genetic load (Figure 4B) and the number of deleterious alleles were significantly reduced compared to simulations recapitulating the mainland population's history (t test, $p < 0.01$; Figures S23–S26), particularly for mildly and strongly deleterious mutations. This result is slightly different from a previous study on Alpine Ibex (*Capra ibex*), which found

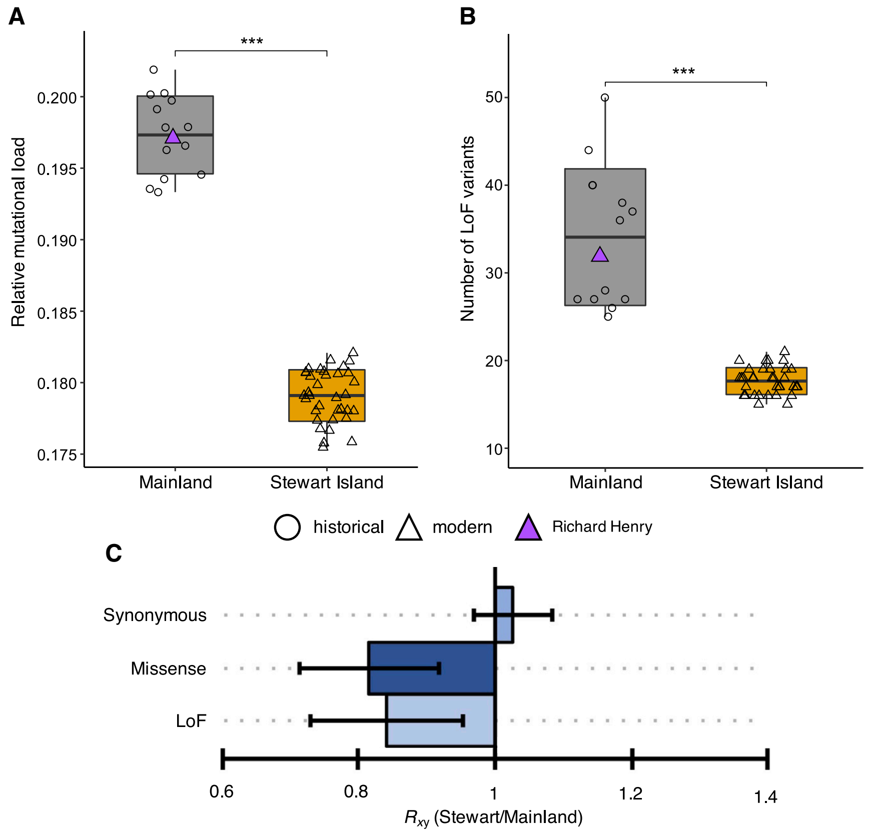

Figure 3. Mutational load estimates for kākāpō

(A) Individual relative mutational load measured as the sum of all homozygous and heterozygous derived alleles multiplied by their conservation score (GERP score > 2) over the total number of derived alleles.

(B) Number of loss of function (LoF) variants per individual. Horizontal lines within boxplots and bounds of boxes represent means and standard deviations, respectively (Welch's two-sample t test; ***p < 0.001). Vertical lines represent minima and maxima.

(C) R_{xy} ratio of derived alleles for synonymous, missense, and LoF variants. R_{xy} < 1 indicates a relative frequency deficit of the corresponding category in Stewart Island compared to mainland kākāpō. Whiskers represent 95% confidence interval (CI).

See also Figures S16–S22.

evidence for the purging of highly deleterious mutations but an accumulation of mildly deleterious mutations.[17] When assuming an extreme population decline, our simulations indicated an increase in additive genetic load (Figure 4B), consistent with strong drift increasing the number and expression of deleterious mutations in homozygous state (Figures S23–S26). These simulations thus suggest that purging requires a large enough population for selection to be effective, whereas in a population that is too small, drift will overwhelm purifying selection. Overall, these results are consistent with the hypothesis that purifying selection has led to purging in the Stewart Island population since its isolation from the mainland some 15–20 ka BP.

Functional consequences of deleterious mutations in modern kākāpō

Our findings highlight that the identification of variants with deleterious effects in the surviving kākāpō population is of critical conservation relevance as these variants will help assess the beneficial and detrimental effects of mixing the extinct mainland and extant Stewart Island genetic lineages.[26] While our results are consistent with a historically high rate of purging of mutational load in Stewart Island kākāpō, the present-day kākāpō population likely still suffers from inbreeding depression, as indicated by the generally low hatching success and poor sperm quality in a large number of males.[6] Moreover, because surviving birds originate from two distinct populations with different levels of mutational load, an assessment of the relative mutational load contributed by the Stewart Island survivors, Richard Henry, and his descendants may be valuable to guide future conservation actions.[27] We therefore examined the predicted functional consequences of the identified LoF variants (STAR Methods). Analyzing the genomes of all modern Stewart Island birds, we identified predicted LoF variants in 61 genes (Tables S6 and S7). We observed predicted LoF variants in genes that have been associated with reproduction (BUB1), development (e.g., BMPR1A, FLT1), and immunity (e.g., IGDCC4; Tables S6 and S7), consistent with observations of low reproductive and hatching success in kākāpō.[6] Interestingly, we found predicted LoF variants in 27 other genes associated with immunity and development (e.g., FLT1) only in the genomes of Stewart Island birds and 21 unique predicted LoF variants in the genome of Richard Henry in genes associated with, for instance, reproduction (e.g., BUB1) and development (e.g., LYN; Table S6).

DISCUSSION

Our population genomic analyses indicate that Stewart Island kākāpō are more inbred but have a lower mutational load than mainland kākāpō. One explanation for this reduced mutational load is that random genetic drift led to a loss of alleles that were at low frequency before the decline in population size on Stewart Island. However, while most deleterious alleles at low frequency will be lost due to random drift, a small proportion will be fixed, meaning that the average frequency of deleterious alleles will not change. Because we observed a reduced relative frequency of missense and LoF variants in the Stewart Island population (Figure 3C), we therefore consider a scenario in which mutational load was reduced through drift alone less likely.[8]

An alternative, and in our view more probable, explanation is that a combination of inbreeding and purifying selection contributed to the reduction in mutational load in the Stewart Island

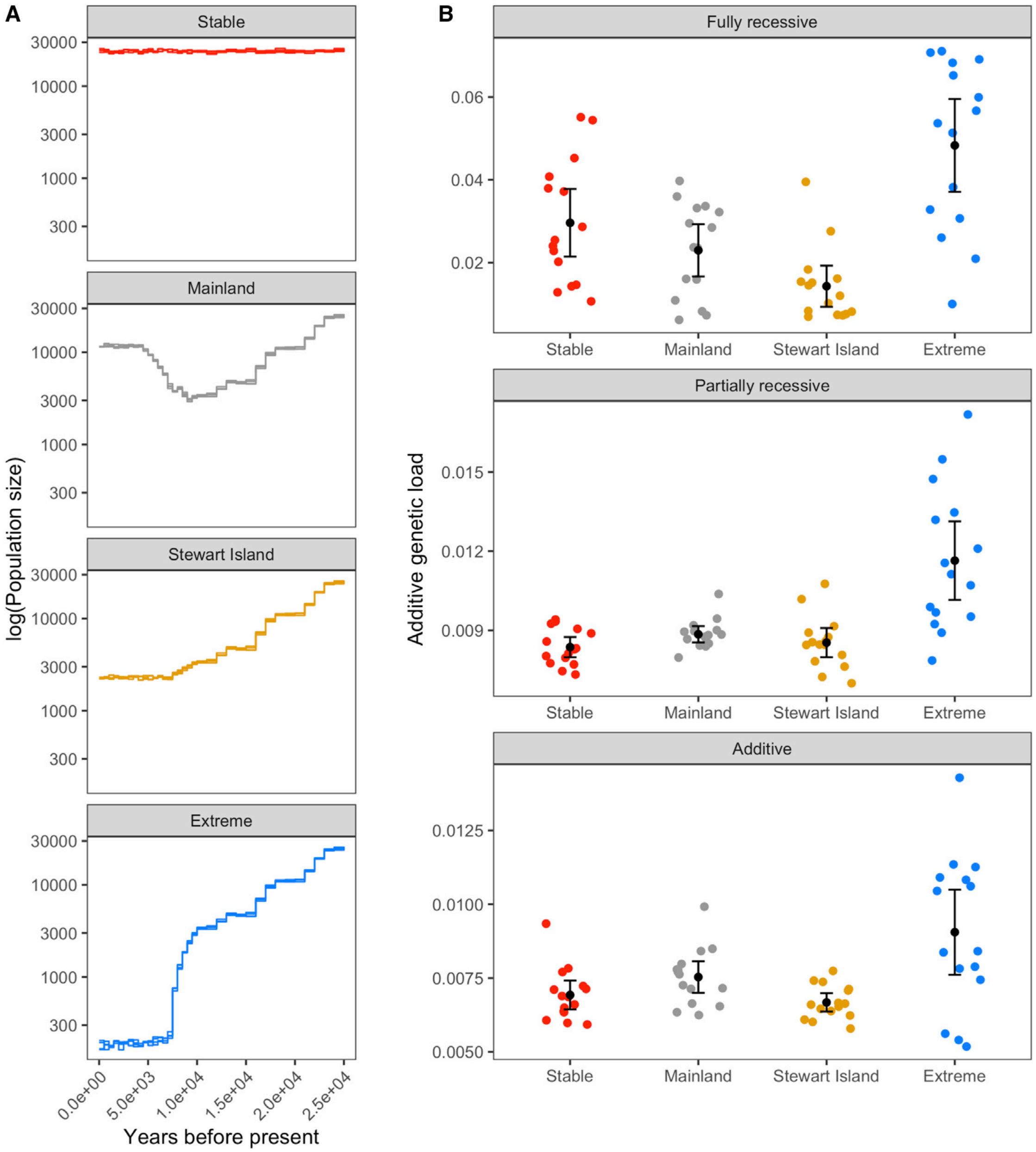

Figure 4. Forward simulations of demographic scenarios and impact on deleterious mutations

(A) Simulated demographic scenarios representing a Stable scenario as control (N_e = 10,000), a Mainland scenario (LGM bottleneck and long-term N_e = 6,000), a Stewart Island scenario (LGM decline and long-term N_e = 1,000), and an Extreme decline scenario (LGM decline and long-term N_e = 100).

(B) Additive genetic load calculated as the sum of selection coefficients for homozygous mutations plus the sum of selection coefficients multiplied by the dominance coefficients for heterozygous mutations. Black dots and whiskers show the means and 95% CIs for each demographic scenario.

See also Figures S23–S27.

population.[15] This interpretation is also supported by the finding of a less pronounced difference in LoF alleles within and outside ROH in the genomes from Stewart Island kākāpō, possibly indicating a reduction in LoF through repeated inbreeding events. Moreover, our forward simulations demonstrated that additive genetic load and the number of deleterious alleles can be reduced in a scenario that recapitulates the demographic history of the Stewart Island population.

Our results suggest that a long-term isolation and slow increase in inbreeding may have offered circumstances for the purging of mutational load in the Stewart Island population.[15] However, it is important to point out that purging in the Stewart Island population is more likely a reflection of its long-term small N_e and that the more recent severe decline may now be exposing the population to the same level of drift load as in our simulated extreme decline scenario.

Previous empirical studies on populations that have experienced population declines have in some cases identified increases in mutational load.[9,10] However, there are also multiple studies that have identified a reduced mutational load following population decline, similar to what we observed in the Stewart Island kākāpō.[10,17,25,28] These contrasting results have important implications since they suggest a complex interaction between population declines and the trajectory of deleterious genetic variation, thus making generalizations across species challenging.

The importance of genetic tools in kākāpō recovery has been increasingly recognized over the past 20 years.[27] Since the translocation of all surviving kākāpō to predator-free islands in the 1980s, efforts have been made to maintain genetic diversity, reduce inbreeding, and avoid the spread of harmful traits.[4,27] Management actions have focused not only on reducing inbreeding by preventing pairings between related individuals[27,29] but also on the maintenance of evolutionary potential by favoring matings with birds of mainland genetic heritage (i.e., Richard Henry and his offspring[27]). While mixing distinct genetic lineages can result in genetic rescue in highly inbred populations,[26] our results show that Richard Henry has a higher mutational load than birds from the Stewart Island population. Even though the fitness and ecological effects of these deleterious mutations are unknown, mixing between the mainland and Stewart Island lineages could lead to the introduction of additional mutational load and thus be detrimental to the viability of the remaining population.[26] Furthermore, the current extremely low population size could be conducive to reduced efficacy of selection and lead to the fixation of deleterious mutations in future generations (i.e., increased drift load). Isle Royal wolves are a natural example of genetic rescue with such unintended negative consequences.[30] Here, the migration of a single male wolf into this small and isolated population resulted in a population decline associated with the introduction of detrimental variation.[16,30] It is thus crucial to balance the positive (i.e., genetic rescue and maintenance of adaptive potential) and negative (i.e., increase in the proportion and expression of mutational load) effects that could result from mixing the two kākāpō genetic lineages and, if necessary, limit breeding between them.[31] Our findings reinforce the need to further examine the genetic basis of inbreeding depression in the extant kākāpō population, in particular with relation to traits associated with fertility and hatching success.

Breeding programs and translocation efforts for other endangered taxa that have experienced severe anthropogenic population declines would benefit greatly from the type of genomic data analyzed here. For instance, evidence of inbreeding depression has been observed also in the New Zealand stitchbird (*Notiomystis cincta*).[32] Similarly, Tasmanian devil (*Sarcophilus harrisii*) populations display very low genetic diversity and high incidence of a tumor disease (Tasmanian devil facial tumor disease [DFTD]).[33] Because captive breeding and/or translocations are part of the management strategy of these species, assessing the mutational load of potential candidates for breeding and translocations will be essential for the success of their conservation.

STAR★METHODS

Detailed methods are provided in the online version of this paper and include the following:

- KEY RESOURCES TABLE
- RESOURCE AVAILABILITY
  - Lead contact
  - Materials availability
  - Data and code availability
- EXPERIMENTAL MODEL AND SUBJECT DETAILS
- METHOD DETAILS
  - DNA extraction
  - Library preparation
- QUANTIFICATION AND STATISTICAL ANALYSIS
  - De-novo assembly and annotation
  - Historical and modern data processing
  - Population structure
  - Demographic reconstruction and population divergence
  - Genomic diversity and inbreeding
  - Mutational load estimation
  - Gene Ontology
  - Forward simulations

SUPPLEMENTAL INFORMATION

Supplemental information can be found online at https://doi.org/10.1016/j.xgen.2021.100002.

ACKNOWLEDGMENTS

We thank the Kākāpō 125+ project for generating the modern genomic data and the New Zealand Department of Conservation (DOC) and Ngāi Tahu for granting access to them. We also thank Anita Gamauf (Vienna Museum, Vienna, Austria), Alan Tennyson (Te Papa, Wellington, New Zealand), Paul Scofield (Canterbury Museum, Christchurch, New Zealand), Brian Gill (Auckland Museum, Auckland, New Zealand), and Walter Boles (Australian Museum, Sydney, Australia) for lending museum skins. We thank Pontus Skoglund for sharing a custom bioinformatics script. We thank Pacific Biosciences, Bionano Genomics, and Arima Genomics for their generous help and for providing genomic sequence data for long reads, optical maps, and HiC linked reads, respectively, of the reference genome Jane. We further thank members of the Rockefeller Vertebrate Genomes (VGP) Lab and the Sanger Institute Genome Reference Informatics Team for help with genome assembly. Resequencing of historical specimens was performed by the Swedish National Genomics Infrastructure (NGI) at the Science for Life Laboratory, which is supported by the Swedish Research Council and the Knut and Alice Wallenberg Foundation. We acknowledge support from the Uppsala Multidisciplinary Centre for Advanced Computational Science for assistance with massively parallel sequencing and access to the UPPMAX computational infrastructure. L.D. and N.D. acknowledge support from FORMAS (grant 2015-676) and the Swiss National Science Foundation (Postdoc Mobility grants P2SKP3_165031 and P300PA_177845), respectively. H.E.M. was funded by an EMBO fellowship (grant 1111-2018) and the European Union's Horizon 2020 research and innovation program under a Marie Skłodowska-Curie grant (840519). K.G. acknowledges support from FORMAS (grant 2016-00835). B.C.R. acknowledges financial support from UO Zoology funding for sample preparation (Kākāpō 125+ project and kākāpō museum samples). E.D.J. acknowledges support from HHMI. A.R. and A.M.P. were supported by the Intramural Research Program of the National Human Genome Research Institute, National Institutes of Health. The work of V.K. and F.T.-N. was supported by the Intramural Research Program of the National Library of Medicine, National

Institutes of Health. L.H. and F.J.M. were supported by the Wellcome Trust (grant 108749/Z/15/Z) and the European Molecular Biology Laboratory. V.E.K. is financially supported by the Knut and Alice Wallenberg Foundation as part of the National Bioinformatics Infrastructure Sweden at SciLifeLab.

AUTHOR CONTRIBUTIONS

Conceptualization, N.D., L.D., B.C.R., and E.D.J.; methodology, N.D., L.D., T.v.d.V., H.E.M., D.D.-d.-M., C.W.W., V.E.K., J.v.S., F.R., B.C.R., E.D.J., J.G., T.W.R.H., and M.F.L.L.; formal analysis, N.D., H.E.M., T.v.d.V., J.H., A.R., A.M.P., J.K., K.H., W.C., S.P., J.D.M.D., A.R.H., G.F., O.F., L.H., F.J.M., V.K., and F.T.-N.; writing, N.D. and L.D., with input from M.K., N.J.G., Y.F., K.G., and all co-authors; resources, L.D., B.C.R., E.D.J., A.M.P., J.K., K.H., H.A.L., A.R.H., O.F., D.I., P.K.D., D.V., A.D., D.E., and R.M.; funding acquisition, L.D.; supervision, L.D.

DECLARATION OF INTERESTS

The authors declare no competing interests.

Received: October 20, 2020
Revised: April 23, 2021
Accepted: June 22, 2021
Published: September 8, 2021

REFERENCES

1. Wilmshurst, J.M., Anderson, A.J., Higham, T.F.G., and Worthy, T.H. (2008). Dating the late prehistoric dispersal of Polynesians to New Zealand using the commensal Pacific rat. Proc. Natl. Acad. Sci. USA *105*, 7676–7680.
2. Duncan, R.P., and Blackburn, T.M. (2004). Extinction and endemism in New Zealand land birds. Glob. Ecol. Biogeogr. *13*, 509–517.
3. Dussex, N., von Seth, J., Robertson, B.C., and Dalén, L. (2018). Full mitogenomes in the critically endangered kākāpō reveal major post-glacial and anthropogenic effects on neutral genetic diversity. Genes *9*, 1–14.
4. Powlesland, R.G., Merton, D.V., and Cockrem, J.F. (2006). A parrot apart: The natural history of the kakapo (*Strigops habroptilus*), and the context of its conservation management. Notornis *53*, 3–26.
5. Bergner, L.M., Dussex, N., Jamieson, I.G., and Robertson, B.C. (2016). European colonization, not polynesian arrival, impacted population size and genetic diversity in the critically endangered New Zealand Kākāpō. J. Hered. *107*, 593–602.
6. White, K.L., Eason, D.K., Jamieson, I.G., and Robertson, B.C. (2015). Evidence of inbreeding depression in the critically endangered parrot, the kakapo. Anim. Conserv. *18*, 341–347.
7. Ouborg, N.J., Pertoldi, C., Loeschcke, V., Bijlsma, R.K., and Hedrick, P.W. (2010). Conservation genetics in transition to conservation genomics. Trends Genet. *26*, 177–187.
8. Lynch, M., Conery, J., and Burger, R. (1995). Mutation Accumulation and the Extinction of Small Populations. Am. Nat. *146*, 489–518.
9. Feng, S., Fang, Q., Barnett, R., Li, C., Han, S., Kuhlwilm, M., Zhou, L., Pan, H., Deng, Y., Chen, G., et al. (2019). The Genomic Footprints of the Fall and Recovery of the Crested Ibis. Curr. Biol. *29*, 340–349.e7.
10. van der Valk, T., Díez-Del-Molino, D., Marques-Bonet, T., Guschanski, K., and Dalén, L. (2019). Historical Genomes Reveal the Genomic Consequences of Recent Population Decline in Eastern Gorillas. Curr. Biol. *29*, 165–170.e6.
11. Rogers, R.L., and Slatkin, M. (2017). Excess of genomic defects in a woolly mammoth on Wrangel island. PLoS Genet. *13*, e1006601.
12. Palkopoulou, E., Mallick, S., Skoglund, P., Enk, J., Rohland, N., Li, H., Omrak, A., Vartanyan, S., Poinar, H., Götherström, A., et al. (2015). Complete genomes reveal signatures of demographic and genetic declines in the woolly mammoth. Curr. Biol. *25*, 1395–1400.
13. Díez-Del-Molino, D., Sánchez-Barreiro, F., Barnes, I., Gilbert, M.T.P., and Dalén, L. (2018). Quantifying Temporal Genomic Erosion in Endangered Species. Trends Ecol. Evol. *33*, 176–185.
14. von Seth, J., Dussex, N., Díez-Del-Molino, D., van der Valk, T., Kutschera, V.E., Kierczak, M., Steiner, C.C., Liu, S., Gilbert, M.T.P., Sinding, M.S., et al. (2021). Genomic insights into the conservation status of the world's last remaining Sumatran rhinoceros populations. Nat. Commun. *12*, 2393.
15. Caballero, A., Bravo, I., and Wang, J. (2017). Inbreeding load and purging: implications for the short-term survival and the conservation management of small populations. Heredity *118*, 177–185.
16. Robinson, J.A., Räikkönen, J., Vucetich, L.M., Vucetich, J.A., Peterson, R.O., Lohmueller, K.E., and Wayne, R.K. (2019). Genomic signatures of extensive inbreeding in Isle Royale wolves, a population on the threshold of extinction. Sci. Adv. *5*, eaau0757.
17. Grossen, C., Guillaume, F., Keller, L.F., and Croll, D. (2020). Purging of highly deleterious mutations through severe bottlenecks in Alpine ibex. Nat. Commun. *11*, 1001.
18. Rhie, A., McCarthy, S.A., Fedrigo, O., Damas, J., Formenti, G., Koren, S., Uliano-Silva, M., Chow, W., Fungtammasan, A., Kim, J., et al. (2021). Towards complete and error-free genome assemblies of all vertebrate species. Nature *592*, 737–746.
19. McKinnon, M. (1997). Plate 12. New Zealand Historical Atlas: Ko Papatuanuku e Takoto Nei (David Bateman).
20. Alloway, B.V., Lowe, D.J., Barrell, D.J.A., Newnham, R.M., Almond, P.C., Augustinus, P.C., Bertler, N.A.N., Carter, L., Litchfield, N.J., McGlone, M.S., et al. (2007). Towards a climate event stratigraphy for New Zealand over the past 30 000 years (NZ-INTIMATE project). J. Quat. Sci. *22*, 9–35.
21. Li, H., and Durbin, R. (2011). Inference of human population history from individual whole-genome sequences. Nature *475*, 493–496.
22. Wood, J.R. (2016). Spatial distribution of late Holocene bird bones in the Mason Bay dune system Stewart Island New Zealand. J. R. Soc. New Zeal. *46*, 103–116.
23. Gibb, J. (1986). A New Zealand regional Holocene eustatic sea-level curve and its application to determination of vertical tectonic movements. R. Soc. N. Z. Bull. *24*, 377–395.
24. Thompson, E.A. (2013). Identity by descent: variation in meiosis, across genomes, and in populations. Genetics *194*, 301–326.
25. Xue, Y., Prado-Martinez, J., Sudmant, P.H., Narasimhan, V., Ayub, Q., Szpak, M., Frandsen, P., Chen, Y., Yngvadottir, B., Cooper, D.N., et al. (2015). Mountain gorilla genomes reveal the impact of long-term population decline and inbreeding. Science *348*, 242–245.
26. Bell, D.A., Robinson, Z.L., Funk, W.C., Fitzpatrick, S.W., Allendorf, F.W., Tallmon, D.A., and Whiteley, A.R. (2019). The Exciting Potential and Remaining Uncertainties of Genetic Rescue. Trends Ecol. Evol. *34*, 1070–1079.
27. Robertson, B. (2006). The role of genetics in kakapo recovery. Notornis *53*, 173.
28. Robinson, J.A., Brown, C., Kim, B.Y., Lohmueller, K.E., and Wayne, R.K. (2018). Purging of Strongly Deleterious Mutations Explains Long-Term Persistence and Absence of Inbreeding Depression in Island Foxes. Curr. Biol. *28*, 3487–3494.e4.
29. Bergner, L.M., Jamieson, I.G., and Robertson, B.C. (2014). Combining genetic data to identify relatedness among founders in a genetically depauperate parrot, the Kakapo (*Strigops habroptilus*). Conserv. Genet. *15*, 1013–1020.
30. Hedrick, P.W., Robinson, J.A., Peterson, R.O., and Vucetich, J.A. (2019). Genetics and extinction and the example of Isle Royale wolves. Anim. Conserv. *22*, 302–309.
31. van Oosterhout, C. (2020). Mutation load is the spectre of species conservation. Nat. Ecol. Evol. *4*, 1004–1006.
32. Brekke, P., Bennett, P.M., Wang, J., Pettorelli, N., and Ewen, J.G. (2010). Sensitive males: inbreeding depression in an endangered bird. Proc. Biol. Sci. *277*, 3677–3684.

33. Hendricks, S., Epstein, B., Schönfeld, B., Wiench, C., Hamede, R., Jones, M., Storfer, A., and Hohenlohe, P. (2017). Conservation implications of limited genetic diversity and population structure in Tasmanian devils (*Sarcophilus harrisii*). Conserv. Genet. *18*, 977–982.
34. Meyer, M., and Kircher, M. (2010). Illumina sequencing library preparation for highly multiplexed target capture and sequencing. Cold Spring Harb. Protoc. *2010*, prot5448.
35. Camacho, C., Coulouris, G., Avagyan, V., Ma, N., Papadopoulos, J., Bealer, K., and Madden, T.L. (2009). BLAST+: architecture and applications. BMC Bioinformatics *10*, 421.
36. Okonechnikov, K., Conesa, A., and García-Alcalde, F. (2016). Qualimap 2: advanced multi-sample quality control for high-throughput sequencing data. Bioinformatics *32*, 292–294.
37. Lord, E., Dussex, N., Kierczak, M., Díez-Del-Molino, D., Ryder, O.A., Stanton, D.W.G., Gilbert, M.T.P., Sánchez-Barreiro, F., Zhang, G., Sinding, M.S., et al. (2020). Pre-extinction Demographic Stability and Genomic Signatures of Adaptation in the Woolly Rhinoceros. Curr. Biol. *30*, 3871–3879.e7.
38. Smit, A.F.A., Hubley, R., and Green, P. (2010). RepeatMasker Open-3.0. 1996-2010 (Institute for Systems Biology).
39. Neethiraj, R., Hornett, E.A., Hill, J.A., and Wheat, C.W. (2017). Investigating the genomic basis of discrete phenotypes using a Pool-Seq-only approach: New insights into the genetics underlying colour variation in diverse taxa. Mol. Ecol. *26*, 4990–5002.
40. Hoff, K.J., Lange, S., Lomsadze, A., Borodovsky, M., and Stanke, M. (2016). BRAKER1: unsupervised RNA-seq-based genome annotation with GeneMark-ET and AUGUSTUS. Bioinformatics *32*, 767–769.
41. Stanke, M., Diekhans, M., Baertsch, R., and Haussler, D. (2008). Using native and syntenically mapped cDNA alignments to improve de novo gene finding. Bioinformatics *24*, 637–644.
42. Stanke, M., Schöffmann, O., Morgenstern, B., and Waack, S. (2006). Gene prediction in eukaryotes with a generalized hidden Markov model that uses hints from external sources. BMC Bioinformatics *7*, 62.
43. Iwata, H., and Gotoh, O. (2012). Benchmarking spliced alignment programs including Spaln2, an extended version of Spaln that incorporates additional species-specific features. Nucleic Acids Res. *40*, e161.
44. Trapnell, C., Roberts, A., Goff, L., Pertea, G., Kim, D., Kelley, D.R., Pimentel, H., Salzberg, S.L., Rinn, J.L., and Pachter, L. (2012). Differential gene and transcript expression analysis of RNA-seq experiments with TopHat and Cufflinks. Nat. Protoc. *7*, 562–578.
45. Roberts, A., Trapnell, C., Donaghey, J., Rinn, J.L., and Pachter, L. (2011). Improving RNA-Seq expression estimates by correcting for fragment bias. Genome Biol. *12*, R22.
46. Huerta-Cepas, J., Szklarczyk, D., Forslund, K., Cook, H., Heller, D., Walter, M.C., Rattei, T., Mende, D.R., Sunagawa, S., Kuhn, M., et al. (2016). eggNOG 4.5: a hierarchical orthology framework with improved functional annotations for eukaryotic, prokaryotic and viral sequences. Nucleic Acids Res. *44* (*D1*), D286–D293.
47. John, J.S. (2011). SeqPrep 1.1. https://github.com/jstjohn/SeqPrep.
48. Li, H., and Durbin, R. (2010). Fast and accurate long-read alignment with Burrows-Wheeler transform. Bioinformatics *26*, 589–595.
49. Li, H., Handsaker, B., Wysoker, A., Fennell, T., Ruan, J., Homer, N., Marth, G., Abecasis, G., and Durbin, R.; 1000 Genome Project Data Processing Subgroup (2009). The Sequence Alignment/Map format and SAMtools. Bioinformatics *25*, 2078–2079.
50. Jónsson, H., Ginolhac, A., Schubert, M., Johnson, P.L.F., and Orlando, L. (2013). mapDamage2.0: fast approximate Bayesian estimates of ancient DNA damage parameters. Bioinformatics *29*, 1682–1684.
51. Bolger, A.M., Lohse, M., and Usadel, B. (2014). Trimmomatic: a flexible trimmer for Illumina sequence data. Bioinformatics *30*, 2114–2120.
52. McKenna, A., Hanna, M., Banks, E., Sivachenko, A., Cibulskis, K., Kernytsky, A., Garimella, K., Altshuler, D., Gabriel, S., Daly, M., and DePristo, M.A. (2010). The Genome Analysis Toolkit: a MapReduce framework for analyzing next-generation DNA sequencing data. Genome Res. *20*, 1297–1303.
53. Li, H. (2011). A statistical framework for SNP calling, mutation discovery, association mapping and population genetical parameter estimation from sequencing data. Bioinformatics *27*, 2987–2993.
54. Quinlan, A.R., and Hall, I.M. (2010). BEDTools: a flexible suite of utilities for comparing genomic features. Bioinformatics *26*, 841–842.
55. Purcell, S., Neale, B., Todd-Brown, K., Thomas, L., Ferreira, M.A.R., Bender, D., Maller, J., Sklar, P., de Bakker, P.I.W., Daly, M.J., and Sham, P.C. (2007). PLINK: a tool set for whole-genome association and population-based linkage analyses. Am. J. Hum. Genet. *81*, 559–575.
56. Zheng, X., Levine, D., Shen, J., Gogarten, S.M., Laurie, C., and Weir, B.S. (2012). A high-performance computing toolset for relatedness and principal component analysis of SNP data. Bioinformatics *28*, 3326–3328.
57. Alexander, D.H., Novembre, J., and Lange, K. (2009). Fast model-based estimation of ancestry in unrelated individuals. Genome Res. *19*, 1655–1664.
58. Simonsen, M., Mailund, T., and Pedersen, C.N.S. (2008). Rapid neighbour-joining. In International Workshop on Algorithms in Bioinformatics, K.A. Crandall and J. Lagergren, eds. (Springer), pp. 113–122.
59. Excoffier, L., and Foll, M. (2011). fastsimcoal: a continuous-time coalescent simulator of genomic diversity under arbitrarily complex evolutionary scenarios. Bioinformatics *27*, 1332–1334.
60. Excoffier, L., Dupanloup, I., Huerta-Sánchez, E., Sousa, V.C., and Foll, M. (2013). Robust demographic inference from genomic and SNP data. PLoS Genet. *9*, e1003905.
61. Lischer, H.E.L., and Excoffier, L. (2012). PGDSpider: an automated data conversion tool for connecting population genetics and genomics programs. Bioinformatics *28*, 298–299.
62. Excoffier, L., and Lischer, H.E.L. (2010). Arlequin suite ver 3.5: a new series of programs to perform population genetics analyses under Linux and Windows. Mol. Ecol. Resour. *10*, 564–567.
63. Schiffels, S., and Wang, K. (2020). MSMC and MSMC2: the multiple sequentially Markovian coalescent. Methods Mol. Biol. *2090*, 147–166.
64. Browning, S.R., and Browning, B.L. (2007). Rapid and accurate haplotype phasing and missing-data inference for whole-genome association studies by use of localized haplotype clustering. Am. J. Hum. Genet. *81*, 1084–1097.
65. Danecek, P., Auton, A., Abecasis, G., Albers, C.A., Banks, E., DePristo, M.A., Handsaker, R.E., Lunter, G., Marth, G.T., Sherry, S.T., et al.; 1000 Genomes Project Analysis Group (2011). The variant call format and VCFtools. Bioinformatics *27*, 2156–2158.
66. Haubold, B., Pfaffelhuber, P., and Lynch, M. (2010). mlRho - a program for estimating the population mutation and recombination rates from shotgun-sequenced diploid genomes. Mol. Ecol. *19* (*Suppl 1*), 277–284.
67. Davydov, E.V., Goode, D.L., Sirota, M., Cooper, G.M., Sidow, A., and Batzoglou, S. (2010). Identifying a high fraction of the human genome to be under selective constraint using GERP++. PLoS Comput. Biol. *6*, e1001025.
68. Cingolani, P., Platts, A., Wang, L., Coon, M., Nguyen, T., Wang, L., Land, S.J., Lu, X., and Ruden, D.M. (2012). A program for annotating and predicting the effects of single nucleotide polymorphisms, SnpEff: SNPs in the genome of Drosophila melanogaster strain w1118; iso-2; iso-3. Fly (Austin) *6*, 80–92.
69. Walker, B.J., Abeel, T., Shea, T., Priest, M., Abouelliel, A., Sakthikumar, S., Cuomo, C.A., Zeng, Q., Wortman, J., Young, S.K., and Earl, A.M. (2014). Pilon: an integrated tool for comprehensive microbial variant detection and genome assembly improvement. PLoS ONE *9*, e112963.
70. Mi, H., Muruganujan, A., Casagrande, J.T., and Thomas, P.D. (2013). Large-scale gene function analysis with the PANTHER classification system. Nat. Protoc. *8*, 1551–1566.
71. Haller, B.C., and Messer, P.W. (2019). SLiM 3: Forward Genetic Simulations Beyond the Wright-Fisher Model. Mol. Biol. Evol. *36*, 632–637.

72. Kim, B.Y., Huber, C.D., and Lohmueller, K.E. (2017). Inference of the distribution of selection coefficients for new nonsynonymous mutations using large samples. Genetics *206*, 345–361.

73. Sambrook, J., Fritsch, E.F., and Maniatis, T. (1989). Molecular Cloning: A Laboratory Manual, Second Edition (Cold Spring Harbor Laboratory Press).

74. Knapp, M., Clarke, A.C., Horsburgh, K.A., and Matisoo-Smith, E.A. (2012). Setting the stage - building and working in an ancient DNA laboratory. Ann. Anat. *194*, 3–6.

75. Briggs, A.W., Stenzel, U., Meyer, M., Krause, J., Kircher, M., and Pääbo, S. (2010). Removal of deaminated cytosines and detection of in vivo methylation in ancient DNA. Nucleic Acids Res. *38*, e87.

76. Kircher, M., Sawyer, S., and Meyer, M. (2012). Double indexing overcomes inaccuracies in multiplex sequencing on the Illumina platform. Nucleic Acids Res. *40*, e3.

77. Aken, B.L., Ayling, S., Barrell, D., Clarke, L., Curwen, V., Fairley, S., Fernandez Banet, J., Billis, K., García Girón, C., Hourlier, T., et al. (2016). The Ensembl gene annotation system. Database (Oxford) *2016*, baw093.

78. Pruitt, K.D., Brown, G.R., Hiatt, S.M., Thibaud-Nissen, F., Astashyn, A., Ermolaeva, O., Farrell, C.M., Hart, J., Landrum, M.J., McGarvey, K.M., et al. (2014). RefSeq: an update on mammalian reference sequences. Nucleic Acids Res. *42*, D756–D763.

79. Kircher, M. (2012). Analysis of high-throughput ancient DNA sequencing data. In Ancient DNA. Methods in Molecular Biology (Methods and Protocols), B. Shapiro, ed. (Humana Press).

80. Saitou, N., and Nei, M. (1987). The neighbor-joining method: a new method for reconstructing phylogenetic trees. Mol. Biol. Evol. *4*, 406–425.

81. Kumar, S., Stecher, G., Suleski, M., and Hedges, S.B. (2017). TimeTree: a resource for timelines, timetrees, and divergence times. Mol. Biol. Evol. *34*, 1812–1819.

82. Jetz, W., Thomas, G.H., Joy, J.B., Hartmann, K., and Mooers, A.O. (2012). The global diversity of birds in space and time. Nature *491*, 444–448.

83. Nielsen, R. (2000). Estimation of population parameters and recombination rates from single nucleotide polymorphisms. Genetics *154*, 931–942.

84. Akaike, H. (1974). A new look at the statistical model identification. IEEE Trans. Automat. Contr. *AC-19*, 716–723.

85. Prado-Martinez, J., Sudmant, P.H., Kidd, J.M., Li, H., Kelley, J.L., Lorente-Galdos, B., Veeramah, K.R., Woerner, A.E., O'Connor, T.D., Santpere, G., et al. (2013). Great ape genetic diversity and population history. Nature *499*, 471–475.

86. Nei, M., and Li, W.H. (1979). Mathematical model for studying genetic variation in terms of restriction endonucleases. Proc. Natl. Acad. Sci. USA *76*, 5269–5273.

87. Lynch, M. (2008). Estimation of nucleotide diversity, disequilibrium coefficients, and mutation rates from high-coverage genome-sequencing projects. Mol. Biol. Evol. *25*, 2409–2419.

88. Pemberton, T.J., Absher, D., Feldman, M.W., Myers, R.M., Rosenberg, N.A., and Li, J.Z. (2012). Genomic patterns of homozygosity in worldwide human populations. Am. J. Hum. Genet. *91*, 275–292.

89. R Development Core Team (2012). R: A Language and Environment for Statistical Computing (R Foundation for Statistical Computing).

90. van der Valk, T., de Manuel, M., Marques-Bonet, T., and Guschanski, K. (2019). Estimates of genetic load in small populations suggest extensive purging of deleterious alleles. bioRxiv. https://doi.org/10.1101/696831.

91. Jarvis, E.D., Mirarab, S., Aberer, A.J., Li, B., Houde, P., Li, C., Ho, S.Y.W., Faircloth, B.C., Nabholz, B., Howard, J.T., et al. (2014). Whole-genome analyses resolve early branches in the tree of life of modern birds. Science *346*, 1320–1331.

92. Zhang, G., Li, C., Li, Q., Li, B., Larkin, D.M., Lee, C., Storz, J.F., Antunes, A., Greenwold, M.J., Meredith, R.W., et al.; Avian Genome Consortium (2014). Comparative genomics reveals insights into avian genome evolution and adaptation. Science *346*, 1311–1320.

93. Haller, B.C., Galloway, J., Kelleher, J., Messer, P.W., and Ralph, P.L. (2019). Tree-sequence recording in SLiM opens new horizons for forward-time simulation of whole genomes. Mol. Ecol. Resour. *19*, 552–566.

94. Haller, B.C., and Messer, P.W. (2019). SLiM 3: Forward genetic simulations beyond the Wright–Fisher model. Mol. Biol. Evol. *36*, 632–637.

95. Pedersen, C.T., Lohmueller, K.E., Grarup, N., Bjerregaard, P., Hansen, T., Siegismund, H.R., Moltke, I., and Albrechtsen, A. (2017). The effect of an extreme and prolonged population bottleneck on patterns of deleterious variation: Insights from the Greenlandic Inuit. Genetics *205*, 787–801.

STAR★METHODS

KEY RESOURCES TABLE

REAGENT or RESOURCE	SOURCE	IDENTIFIER
Chemicals, peptides, and recombinant proteins		
Tango Buffer (10X)	ThermoFisher Scientific	Cat#BY5
ATP (100mM)	ThermoFisher Scientific	Cat#R0441
T4 Polynucleotide Kinase (10U/ul)	ThermoFisher Scientific	Cat#EK0032
T4 DNA Polymerase 5U/ul	ThermoFisher Scientific	Cat#EP0062
USER Enzyme	NEB	Cat#M5505L
T4 DNA Ligase (5U/ul)	ThermoFisher Scientific	Cat#EL0011
Bst Polymerase, Large Fragments	NEB	Cat#M0275S
AccuPrime Pfx	ThermoFisher Scientific	Cat#12344024
T4 DNA ligase (400U/ul)	NEB	Cat#M0202S
T4 DNA polymerase (3U/ul)	NEB	Cat#M0203S
PEG-4000	Sigma-Aldrich	Cat#95904-250G-F
Critical commercial assays		
High Sensitivity DNA kit	Agilent	Cat#5067-4626
DNeasy Blood & Tissue Kit	QIAGEN, Hilden, Germany	Cat#69504
Deposited data		
Raw fastq reads	This study	Historical resequencing data: ENA:PRJEB35522; modern resequencing data: https://repo.data.nesi.org.nz/TAONGA-KAKAPO
de-novo assembly for *Strigops habroptilus*	This study	GenBank: GCF_004027225.2 and GCA_004011185.1; https://ftp.ncbi.nlm.nih.gov/genomes/all/GCF/004/027/225/GCF_004027225.2_bStrHab1.2.pri/GCF_004027225.2_bStrHab1.2.pri_genomic.fna.gz
Oligonucleotides		
IS1 adaptor P5: 5′- A*C*A*C*TCTTTCCCTACACG ACGCTCTTCCG*A*T*C*T-3′	Meyer and Kircher[34]; Sigma-Aldrich	N/A
IS2 adaptor P7: 5′- G*T*G*A*CTGGAGTTCAGAC GTGTGCTCTTCCG*A*T*C*T-3′	Meyer and Kircher[37]; Sigma-Aldrich	N/A
IS3 adaptor P5+P7: 5′- A*G*A*T*CGGAA*G*A*G*C-3′	Meyer and Kircher[37]; Sigma-Aldrich	N/A
Illumina AmplifyingPrimer IS4: 5′- AATGATACGGCG ACCACCGAGATCTACACTCTTTCCCTACACGACG CTCTT-3′	Meyer and Kircher[37]; Sigma-Aldrich	N/A
Illumina Indexing Primer: 5′- CAAGCAGAAGACGGC ATACGAGATNNNNNNNGTGACTGGAGTTCAGAC GTGT-3′	Meyer and Kircher[37]; Sigma-Aldrich	N/A
Ns represent indexes		N/A
Software and algorithms		
VGP pipeline	Rhie et al.[18]	https://vertebrategenomesproject.org/
BLAST+ 2.5.0	Camacho et al.[35]	NCBI
Qualimap v2.2.1	Okonechnikov et al.[36]	http://qualimap.bioinfo.cipf.es/
CpG site masking script	von Seth et al.[14]; Lord et al.[37]	https://github.com/tvdvalk/find_CpG
RepeatMasker v4.0.7	Smit et al.[38]	http://repeatmasker.org
MESPA pipeline	Neethiraj et al.[39]	https://sourceforge.net/projects/mespa/
BRAKER v2.1.1	Hoff et al.[40]; Stanke et al.[41,42]	https://github.com/Gaius-Augustus/BRAKER

(Continued on next page)

Continued

REAGENT or RESOURCE	SOURCE	IDENTIFIER
SPALN2	Iwata and Gotoh[43]	https://github.com/ogotoh/spaln
cufflinks v 2.2.1	Trapnell et al.[44]; Roberts et al.[45]	http://cole-trapnell-lab.github.io/cufflinks/
eggNOG-mapper v4.5.1	Huerta-Cepas et al.[46]	http://eggnog-mapper.embl.de/
bcl2Fastq v1.17.1	Illumina	https://support.illumina.com/sequencing/sequencing_software/bcl2fastq-conversion-software.html
SeqPrep	John[47]	https://github.com/jstjohn/SeqPrep
BWA v0.7.13	Li and Durbin[48]	http://bio-bwa.sourceforge.net/
SAMtools v1.3	Li et al.[49]	https://sourceforge.net/projects/samtools/files/samtools/1.3/
Picard v1.141	Broad Institute	http://broadinstitute.github.io/picard
Mapdamage v2.0	Jónsson et al.[50]	https://ginolhac.github.io/mapDamage/
Trimmomatic v0.32	Bolger et al.[51]	http://www.usadellab.org/cms/?page=trimmomatic
GATK v3.4.0	McKenna et al.[52]	https://gatk.broadinstitute.org/hc/en-us
bcftools v1.3	Li[53]	http://www.htslib.org/
BEDtools v2.29.2	Quinlan and Hall[54]	https://bedtools.readthedocs.io/en/latest/
PLINK v1.9	Purcell et al.[55]	https://www.cog-genomics.org/plink2/
SNPRelate	Zheng et al.[56]	https://www.bioconductor.org/packages/release/bioc/html/SNPRelate.html
ADMIXTURE v1.3.0	Alexander et al.[57]	http://dalexander.github.io/admixture/publications.html
RapidNJ v2.3.2	Simonsen et al.[58]	https://anaconda.org/bioconda/rapidnj
Timetree	Kumar et al.[68]	http://timetree.org/
PSMC v0.6.5	Li and Durbin[21]	https://github.com/lh3/psmc
Fastsimcoal v2.6	Excoffier and Foll [59]; Excoffier et al.[60]	http://cmpg.unibe.ch/software/fastsimcoal2/
PGDspider	Lischer and Excoffier[61]	http://www.cmpg.unibe.ch/software/PGDSpider/
Arlequin v3.5	Excoffier and Lischer[62]	http://cmpg.unibe.ch/software/arlequin35/
MSMC2	Schiffels and Wang [63]	https://github.com/stschiff/msmc2
Beagle v5.1	Browning and Browning[64]	https://faculty.washington.edu/browning/beagle/beagle.html
vcftools	Danecek[65]	http://vcftools.sourceforge.net/
mlRho v2.7	Haubold et al.[66]	http://guanine.evolbio.mpg.de/mlRho/
R	R Development Core Team[84]	https://www.r-project.org/
GERP++	Davydov et al.[67]	http://mendel.stanford.edu/sidowlab/downloads/gerp/index.html
htsbox v1.0	N/A	https://github.com/lh3/htsbox
SNPeff v4.3	Cingolani et al.[68]	http://snpeff.sourceforge.net/index.html
Pilon v1.22	Walker et al.[69]	https://github.com/broadinstitute/pilon/releases/tag/v1.22
Panther v16.0	Mi et al.[70]	http://www.pantherdb.org/
SLiM 3	Haller and Messer[71]; Kim et al.[72]	https://messerlab.org/slim/
**Other**		
Proteinase K	VWR	Cat#1.24568.0100
dNTPs	VWR	Cat#733-1854
Min Elute PCR purification Kit	QIAGEN	Cat#28006
Agencourt AmPure XP 5mL Kit	Beckman Coulter	Cat#63880

RESOURCE AVAILABILITY

Lead contact

Further information and requests for resources and reagents should be directed to and will be fulfilled by the lead contact, Nicolas Dussex (nicolas.dussex@gmail.com).

Materials availability

This study did not generate new unique reagents.

Data and code availability

The genome assembly can be accessed at the NCBI database under BioProject: PRJNA510145. Assembly accesion numbers are Genbank: GCF_004027225.2 and GCA_004011185.1.

Historical resequencing data can be accessed at the European Nucleotide Archive under project ENA: PRJEB35522.

Modern resequencing data https://repo.data.nesi.org.nz from the ongoing Kākāpo 125+ project is maintained at the Genomics Aotearoa data repository (direct link to the Kākāpo 125+ genome sequencing dataset at https://repo.data.nesi.org.nz/TAONGA-KAKAPO). In this study, we used 36 genomes from a dataset currently consisting of 145 whole-genome sequences of kākāpo (raw and untrimmed fastq files). This dataset is made available with controlled access, managed via a data access committee of the New Zealand Department of Conservation (DOC) and Te Rūnanga o Ngāi Tahu. The kākāpo samples were obtained under an agreement that the genomic data is shared in accordance with principles of indigenous data sovereignty and that Te Rūnanga o Ngāi Tahu maintain Kaitiakitanga (i.e., governance and responsibility) over the data. The terms of the controlled access and management follow this agreement. To request access, users need to submit an application from the Genomics Aotearoa repository. This application needs to be made on the form provided at the Genomics Aotearoa repository or directly at the Kākāpo 125+ webpage (https://www.doc.govt.nz/our-work/kakapo-recovery/what-we-do/research-for-the-future/kakapo125-gene-sequencing/request-kakapo125-data/; a copy of this application form is provided in Figure S28). The application will require applicants to provide details of their proposed research project, including names of researchers and collaborators, if phenotypic data about Kākāpo traits is required from DOC, what engagement with Māori has been undertaken (e.g., discussions with Te Rūnanga o Ngāi Tahu about the proposed project, involvement of Māori researchers, benefit sharing with Māori), what considerations have been made for Mātauranga Māori (Māori knowledge; e.g., is Mātauranga Māori part of the project, will results of the project be fed back to Māori, are there intellectual property concerns that could affect Mātauranga Māori?). The application will also require a project summary that details the planned research studies, and which includes a section that details how this research will benefit kākāpo conservation. Direct benefit to kākāpō conservation is preferred, but is not a requirement for acceptance. Applications will be regularly assessed by DOC and Te Rūnanga o Ngāi Tahu. The default approach is to approve applications, as long as the applications are complete. Applications for basic research and to replicate previously published analyses are highly likely to be accepted. It is possible that an application will be rejected if there are significant concerns raised by DOC or Te Rūnanga o Ngāi Tahu. Concerns raised might include commercial use of the data (e.g., data shared and/or used by a for-profit organization such as drug or other companies), the applicant having a track-record of unethical behavior, and loss of the ability to exercise Kaitiakitanga (i.e., governance and responsibility) over the data, among others.

EXPERIMENTAL MODEL AND SUBJECT DETAILS

The sample for the reference kākāpō genome was from a female named Jane (deceased in 2018 of natural causes), collected as part of the G10K-VGP Project, avian B10K Project, and the Kākāpō Recovery Program. We obtained modern genomic data sequenced from blood DNA extracts by the Kākāpō 125+ Project for one mainland male (i.e., Richard Henry) and 35 birds from Stewart Island discovered on Stewart Island in the 1980s (Table S1). Out of these 36 modern birds, 28 birds, including Richard Henry, were founders of the current population and are thus the direct ancestors of all 201 surviving kākāpō. Seven additional founders were not included in our dataset. We also obtained samples from 13 historical birds collected between 1847 and 1924 (Table S1) from the South Island of New Zealand hereafter referred to as the mainland population. Because kākāpō have a long generation time (~15 years; see Methods S1), we can assume that the historical specimens correspond to the same temporal period.

METHOD DETAILS

DNA extraction

For the *de-novo* assembly and modern samples, DNA was extracted using a phenol/chloroform extraction protocol[73] (see Methods S1).

For historical samples, we extracted DNA from samples with high endogenous DNA content (i.e., 75.9%–91.4%)[3] using a DNeasy Blood & Tissue Kit (QIAGEN, Hilden, Germany). Appropriate precautions were taken to minimize the risk of contamination in historical samples.[74]

Library preparation

For the *de-novo* assembly, 15kb and 30kb PacBio libraries were generated, and long-range scaffolding performed with Bionano optical mapping (see Methods S1).

For modern samples, double-stranded libraries were prepared according to New Zealand Genomics Limited (NZGL, Palmerston North, New Zealand) protocols for modern DNA and sequenced on an Illumina HiSeq2500 with a 2 × 150bp setup.

For historical samples, we prepared double-stranded Illumina libraries according to Meyer & Kircher.[34] We used 20 μL of DNA extract in a 40 μL blunt-end repair reaction with the following final concentration: 1 × buffer Tango, 100 μM of each dNTP, 1 mM ATP, 25 U T4 polynucleotide kinase (Thermo Scientific) and 3U USER enzyme (New England Biolabs). To reduce biases caused by erroneous variant calls in historical genomes, we performed USER enzyme treatment to excise uracil residues resulting from post-mortem damage.[75,76] Samples were incubated for 3 h at 37°C, followed by the addition of 1 μL T4 DNA polymerase (Thermo Scientific) and incubation at 25°C for 15 min and 12°C for 5 min. We then cleaned the samples using MinElute spin columns following the manufacturer's protocol and eluted them in 20ul EB Buffer. Next, we performed an adaptor ligation step to ligate DNA fragments within each library to a combination of incomplete, partially double-stranded P5- and P7-adapters (10 μM each). This reaction was done in a 40 μL reaction volume using 20 μL of blunted DNA from the clean-up step and 1 μL P5-P7 adaptor mix per sample with a final concentration of 1 × T4 DNA ligase buffer, 5% PEG-4000, 5U T4 DNA ligase (Thermo Scientific). Samples were incubated for 30 minutes at room temperature and cleaned using MinElute spin columns as described above. Next, we performed an adaptor fill-in reaction in 40 μL final volume using 20 μL adaptor ligated DNA with a final concentration of 1 × Thermopol Reaction Buffer, 250 μM of each dNTP, 8U *Bst* Polymerase, Long Fragments. The libraries were incubated at 37°C for 20 minutes, heat-inactivated at 80°C for 20 minutes. These libraries were then used as stock for indexing PCR amplification.

In order to increase library complexity, we performed six indexing PCR amplifications for each library using different P7 indexing primers[34] in 25 μL volumes with 3 μL of adaptor-ligated library as template, with the following final concentrations: 1x AccuPrime reaction mix, 0.3 μM IS4 amplification primer, 0.3 μM P7 indexing primer, 7 U AccuPrime Pfx (Thermo Scientific) and the following cycling protocol: 95°C for 2 min, 12 cycles at 95°C for 30 s, 55°C for 30 s and 72°C for 1 min and a final extension at 72°C for 5 minutes.

We used Agencourt AMPure XP beads (Beckman Coulter, Brea, CA, USA) for purification and size selection of libraries, first using 0.5X bead:DNA ratio and second 1.8X to remove long and short (i.e., adaptor dimers) fragments, respectively. We then measured library concentration with a high-sensitivity DNAchip on a Bioanalyzer 2100 (Agilent, Santa Clara, CA, USA). Finally, multiplexed libraries (i.e., six indexed libraries) were pooled in equimolar concentrations and sequenced on an Illumina HiSeqX with a 2 × 150bp setup in the High Output mode at the SciLifeLab sequencing facility in Stockholm.

QUANTIFICATION AND STATISTICAL ANALYSIS

De-novo assembly and annotation

The kākāpō assembly was generated with the Vertebrate Genomes Project (VGP) v1.6 assembly pipeline[18] using a combination of PacBio and Hi-C libraries (see Methods S1). The final assembly size was of 1.17 Gb, with a scaffold N50 of 83.2Mb and assigned to 26 chromosomes (24 autosomes and two sex chromosomes; see Methods S1). We identified Z and W chromosomes from the assembled genome by blasting all scaffolds against the Z-chromosome of zebra finch (v3.2.4, *Taeniopygia guttata*; GenBank: GCA_000151805.2) and W-chromosome of chicken (v5.0, *Gallus gallus*, GenBank: GCA_000002315.5) using BLAST+ 2.5.0.[35] The BLAST+ parameters were set as: -evalue = 1e-10; -word_size = 15; -max_target_seqs = 1000. We excluded the identified Z chromosome (CM013763.1; 101.23Mb) and W chromosome (CM013773.1; 35.7Mb), from all downstream analyses in order to avoid bias associated with analyses relying on heterozygosity estimates. We also visually examined genome coverage estimated with Qualimap v2.2.1[36] (see below) for males and females alignments to confirm the identity of the Z and W chromosomes. Males had on average ~15X and ~0X for the Z and W chromosome, respectively, and females had on average ~7X and ~7X for the Z and W chromosome, respectively. We identified CpG sites using a custom script masking CG sites[14,37] and masked repetitive elements in the genome assembly using RepeatMasker v4.0.7[38] applying the repeat element library of the *aves* database.

We annotated the assembly using the MESPA pipeline[39] (see Methods S1). Briefly, we collapsed reference protein sets for zebra finch (*Taeniopygia guttata*; GenBank: GCA_000151805.2) to 90% coverage following Uniprot90 guidelines using a custom script to only retain sequences with at least 90% sequence identity to, and 80% overlap with, the longest sequence. We then generated an annotation in gff format and extracted 85% (13,175 out of 15,342) high quality kākāpō protein models using zebra finch as a reference protein set. We refined this annotation using the BRAKER2 v2.1.1 pipeline[40–42] and used the resulting zebra finch proteome to predict kākāpō proteins with the exon-aware, protein-to-genome aligner SPALN2.[43] We then extracted CDSs and protein sequences from this annotation with cufflinks v2.2.1[44,45] gffread command using the -V option to exclude genes with in-frame STOP codons. We identified 16,171 kākāpō gene models with a mean length of 1,514bp (Median = 672; min = 50; max = 26,940) to be used in downstream analyses. Finally, we performed a functional annotation of these gene models using the eggNOG-mapper v4.5.1[46] and obtained 15,699 annotated gene models (see Methods S1).

Two other annotations not used in downstream analyses were also generated using the Ensembl gene annotation system[77] and NCBI Eukaryotic Genome Annotation Pipeline[78] (see Methods S1).

Historical and modern data processing

All data processing and analyses were performed on resources provided by the Swedish National Infrastructure for Computing (SNIC) at UPPMAX, Uppsala University. Raw historical sequence data were demultiplexed using bcl2Fastq v2.17.1 with default settings (Illumina Inc.). We merged forward and reverse sequencing reads before mapping as recommended for damaged and short reads.[79] We used SeqPrep v1.1[47] to trim adapters and merge paired-end reads using default settings. We made a minor modification to the source code, which enabled to choose the best quality score of the overlapping bases in the merged region instead of aggregating the scores, following Palkopoulou et al.[12] We mapped the merged reads against the reference genome using the BWA v0.7.13 aln algorithm[48] with deactivated seeding (-l 16,500), allowing more substitutions (-n 0.01) and allowing up to two gaps (-o 2). We used the BWA samse command to generate alignments in SAM format and Samtools v1.3[49] to convert these alignments to BAM format, sort and index them. Finally, we removed PCR duplicate reads using a custom python script that takes into account both start and end position of the reads.[12] Even though all historical genomes were USER-treated[75,76] during library preparation to remove post-mortem DNA damage, we used mapDamage v2.0[50] on the 13 historical samples to estimate damage patterns (Figure S4).

For modern samples, we trimmed forward and reverse reads to remove Illumina adapters using Trimmomatic v0.32 with default settings[51] and then mapped them to the reference genome using BWA mem v0.7.13.[48] Samtools was used for sorting, indexing, and removing duplicates from the alignments.

Next, we processed historical and modern bam files using the same approach. We used Picard v1.141 to assign read group information including library, lane and sample identity to each bam file. We then re-aligned reads around indels using GATK IndelRealigner v3.4.0,[52] and only kept reads with mapping quality mapQ $\geq$ 30 for subsequent analysis. For each genome, we estimated the depth of coverage using Qualimap v2.2.1.[36] After this filtering, average genome coverage ranged from 11.8 and 18.2 (average = 15.3) and from 10.3 to 27.7 (average = 14.2) for modern and historical genomes, respectively (Table S1).

We called variants in historical and modern genomes separately for each individual using bcftools mpileup v1.3and bcftools call v1.3[49,53] using a minimum depth of coverage (DP4) of 1/3X of the average coverage (i.e., 5X) and removed SNPs with base quality QV < 30 and those within 5bp of indels. We also filtered out SNPs in heterozygous state with an allelic balance (i.e., number of reads displaying the reference allele/depth) of < 0.2 and > 0.8 in order to avoid biases caused by contamination, mapping or sequencing error.

We removed the Z and W chromosomes, hard masked all identified CpG sites and repeat regions using BEDtools v2.27.1.[54] After merging all 49 individual vcf. files we obtained 2,785,380 high quality SNP calls. We then used PLINK v1.9[55] to filter variants not covered in all of the 49 individuals resulting in a total of 880,370 high quality SNPs that were used in all downstream analyses (i.e., population structure, demography, genome-wide diversity and inbreeding, mutational load estimation).

Population structure

We first used the R package SNPRelate to perform a principal component analysis (PCA) based on the genetic covariance matrix calculated from the genotypes[56] using our filtered SNP dataset.

Second, we used the program ADMIXTURE v1.3.0[57] to identify genetic clusters (K = 1-4) within our dataset. This program estimates ancestry in a model-based manner where individuals are considered unrelated and uses a cross-validation procedure to determine the best number of possible genetic groups present in the dataset.

Third, we constructed a phylogenetic tree using RapidNJ v2.3.2[58] based on the neighbor-joining method.[80] This method calculates the distance matrix of D_{ij} between each pair of individuals (i and j) with the following formula:

$$D_{ij} = \sum_{m=1}^{M} d_{ij}/L$$

Where, M is the number of segregating sites in i and j, L is the length of regions, d_{ij} is the distance between individuals i and j at given site. $d_{ij} = 0$, when individuals i and j are both homozygous for the same allele (AA/AA); $d_{ij} = 0.5$, when at least one of the genotypes of an individual i or j is heterozygous (Aa/AA, AA/Aa or Aa/Aa); and $d_{ij} = 1$, when individuals i and j are both homozygous but for different alleles (AA/aa or aa/AA).

Since all three methods agreed in the main population structure within the specimens in our dataset and showed a clear distinction between the Stewart Island and the mainland population (Figure S5), we used the identified clusters for all downstream analyses. All mislabelled specimens (i.e., VM5, AUC2, LEI2, AUS1) were analyzed as part of the population they were genetically assigned to.

Demographic reconstruction and population divergence

We used the Pairwise Sequentially Markovian Coalescent (PSMC v0.6.5)[21] model to estimate temporal changes in effective population sizes (N_e) of kākāpō. We generated consensus sequences for all autosomes of a subset of historical and modern genomes using the Samtools mpileup v1.3[49] command and the 'vcf2fq' command from vcfutils.pl. We filtered for base and mapping quality below 30, and depth below 1/3X of the average coverage for each specimen. We set N (the number of iterations) = 30, t (Tmax) = 15 and p (atomic time interval) = 64 (4+25*2+4+6, for each of which parameters are estimated with 28 free interval parameters).

To estimate the substitutions rate per site/year, we used TimeTree,[81] which estimates the substitution rate based on automated literature searches. We aligned 135 birds genomes and assumed a divergence time of 25 my BP between the kea and kākāpō lineages.[82] We obtained an estimate of 0.89×10^{-9} substitutions/site/year.

In order to scale population parameters, we assumed a generation time of 15 years making for a rate (μ) of 1.33×10^{-8} substitutions/site/generation which is biologically realistic in a large and natural kākāpō population (data not shown; see Methods S1).

Second, we reconstructed the population history of kākāpō and estimated divergence times between the Stewart Island (N = 35) and the mainland (N = 11; excluding TEP11, AUC2 and VM5 which formed their own cluster; Figures 1and S5) populations and their effective population sizes (N_e) using a composite-likelihood method based on the site frequency spectrum[83] implemented in fastsimcoal2 v2.6.[59,60] We obtained a folded site frequency spectrum by converting the vcf file filtered for missing data (880,370 SNPs) into Arlequin format in PGDspider[61] and then by converting the resulting Arlequin file into a joint Site Frequency Spectrum (joint 2D-SFS) in Arlequin v3.5.[62] We also collapsed all SFS entries less than 5 in a single category (command line option –C5). We designed four competing scenarios including a post-glacial population size change (bottleneck or expansion) and a divergence event of the Stewart Island population from the Mainland: (a) *Post-glacial divergence*, (b) *Post-glacial divergence followed by Stewart Island population expansion*, (c) *Post-glacial divergence followed by Mainland population expansion* and (d) *Post-glacial divergence followed by Stewart Island and Mainland populations expansion* (Figure S6). The latter population size change was not constrained in the model in order to allow for either a bottleneck or population expansion to occur but is referred to as an expansion since it was supported by the simulations (Figure S8; Table S3). For each scenario, we carried out 50 replicate runs with the following settings: -n 100000 -m -q -M 0.001 -l 10 -L 40. Initial prior distributions followed a log-uniform distribution for population sizes ($N_{pre\text{-}glac}$: 10^3 –10^5; N_{Main}: 10^3 –10^5; and $N_{Stewart}$: 10^2 –10^4; $N_{glac\text{-}main}$: 10^3 –10^5; and $N_{glac\text{-}Stewart}$: 10^2 –10^4), timing of glacial bottleneck (T_{BOT}: 10^2 – 10^5), timing of divergence (T_{DIV}: 5×10^2 – 1.5×10^3), timing of terminal expansion for both populations (T_{EXP}: 5×10^2 – 1.5×10^3). The data was modeled as FREQ (1 bp simulated for each locus), with the number of independent chromosomes equal to the total number of loci (including monomorphic loci) characterized. We used the same substitution rate and generation time as mentioned above for the PSMC. We then used the range in parameter estimates across the initial 50 runs as the prior distribution for another 50 replicates within each scenario, until no further increase in likelihood was detected. The parameter values from the final run with the highest likelihood for each scenario were then used for 50 additional runs with –n = 1000000 to obtain a final estimate of the maximum observed likelihood. We assessed the best fitting scenario by Akaike's information criterion (AIC) score[84] and with the AIC's weight (w), as described in Excoffier et al.[60] (Table S2). We then used the parameter values from the best-fitting scenario to simulate 100 parametric bootstraps datasets. In order to obtain confidence intervals for parameter estimates, we used the *.tpl and initial prior distribution *.est files that led to the best replicate and ran 50 replicates per simulated dataset, making for a total of 5000 parameter estimates (Table S3). We changed the data type to DNA (1 bp), with the number of chromosomal segments equalling the total number of loci in the SFS (including monomorphic sites).

Third, we used the multiple sequential Markovian coalescent (MSMC2) model[63] based on phased haplotypes from the two populations to infer changes in kākāpō N_e. We used Beagle v5.1[64] on default settings to phase the SNP-calls. Genome mappability masks and multi-sample input files were obtained using msmc-tools following the pipelines described in Schiffels and Wang.[63] MSMC2 was then run using the five genomes with highest coverage for each population and using default settings. We used the same substitution rate (μ) and generation time as those described for the PSMC for scaling.

Finally, we estimated the split time (T), assuming no coalescent events since divergence between the mainland and Stewart Island using the PSMC approach applied to a pseudo-diploid Z chromosome genome as described in Palkopoulou et al.[12] We extracted the Z-chromosomes from one mainland historical (CAN1) and one modern Stewart Island (Ruth) female. We generated a Z chromosome haploid consensus sequence for each these two females and merged them into a pseudo-diploid sequence using the seqtk mergefa command. We then applied the PSMC method on the pseudo-diploid Z chromosome to estimate changes in N_e over time. Finally, we rescaled the pseudo-diploid Z chromosome curve to 0.25 consistent with the effective population size of chromosome Z relative to that of autosomes (sex-chromosome/autosome ratio: 0.75). We ran the analysis using the same quality filters, parameters (i.e., 64 discrete time intervals) and the same substitution rate as above for the PSMC on autosomes. As a comparison, we also ran the analysis using fewer discrete intervals (i.e., 49 = 6+4+3+13*2+4+6 or 37 = 2+2+1+15*2+2) as recommended by Prado-Martinez et al.[85] in order to avoid underestimation of the split time.

Genomic diversity and inbreeding

We first estimated genome-wide population-level nucleotide diversity (π)[86] in mainland and Stewart Island birds with vcftools[65] using a sliding window of 10kbp.

Second, we used mlRho v2.7[66] to estimate the mutation rate (θ), which approximates the per site heterozygosity under the infinite sites model and uses bam files as input. We first filtered out bases and reads with quality below 30, and positions with root-mean-squared mapping quality below 30 from the historical and modern bam files. Because high or low coverage in some regions resulting from structural variation can create erroneous mapping to the reference genome and false heterozygous sites, for each specimen, we also filtered out sites with depth lower than five times and higher than two times the average coverage across all our specimens. We then estimated the individual θ as the number of heterozygote sites per 1,000bp. The maximum likelihood approach implemented in mlRho has been shown to provide unbiased estimates of average within-individual heterozygosity at high coverage.[66,87]

Third, we estimated individual inbreeding coefficients, by estimating the number and length of Runs of homozygosity (ROH). ROH are long tracts of the genome with very little or no heterozygote sites that can inform about recent and past population events and can be used to estimate individual inbreeding levels.[88] We used PLINK v1.9[55] to identify ROH and per sample inbreeding coefficients (F_{ROH}). We first converted the filtered multi-individual vcf. file comprising 35 Stewart Island and 14 mainland individuals into a ped file and identified ROH in autosomal chromosomes. We used a sliding window size of 100 SNPs (*homozyg-window-snp 100*). We assumed a window to be homozygous if there were not more than 1 heterozygous site per window (*homozyg-window-het 1*). Moreover, if at least 5% of all windows that included a given SNP were defined as homozygous, the SNP was defined as being in a homozygous segment of a chromosome (*homozyg-window-threshold 0.05*). This threshold was chosen to ensure that the edges of a ROH are properly delimited. Furthermore, a homozygous segment was defined as a ROH if all of the following conditions were met: the segment included $\geq$ 25 SNPs (*homozyg-snp 25*) and covered $\geq$ 100kb (*homozyg-kb* 100); the minimum SNP density was one SNP per 50kb (*homozyg-density 50*); the maximum distance between two neighboring SNPs was $\leq$ 1,000kb (*homozyg-gap 1,000*). For the number of heterozygous sites within ROH, we set the value at 750 (*homozyg-het 750*) in order to prevent sequencing errors to cut ROH. Based on these results, we estimated the inbreeding coefficient F_{ROH} estimated as the overall proportion of the genome contained in ROH.

While we were mainly interested in estimating the relative difference between mainland and Stewart Island birds, we also assessed the robustness of our results to the various parameters used and to potential sequencing errors, by running the same analysis using more stringent parameters. Specifically, we varied the number of heterozygous sites per ROH segment (*homozyg-het 1*), at least one SNP in a ROH per 100kb (*homozyg-density* 100) and the maximum distance between two neighboring SNPs (*homozyg-gap* 500).

We statistically compared heterozygosity, F_{ROH} between mainland and Stewart Island kākāpō using a Welch's two-sample t tests in R.[89]

Mutational load estimation

We estimated mutational load in mainland and Stewart Island kākāpō genomes using two approaches. First, we measured the relative mutational load in each individual as the number of derived alleles at sites that are under strict evolutionary constraints (i.e., highly conserved) and thus likely to be deleterious using genomic evolutionary rate profiling scores (GERP) with the GERP++ software[67] and following van der Valk et al.[90] We included both heterozygous (counted as one allele) and homozygous positions (counted as two alleles) even though the mutational effect of heterozygous positions depends on additional assumptions about the dominance coefficient. GERP identifies constrained elements in multiple alignments by quantifying the amount of substitution deficits (e.g., substitutions that would have occurred if the element were neutral DNA, but did not occur because the element has been under functional constraint) by accounting for phylogenetic divergence. High GERP scores (> 1) represent highly conserved regions whereas low scores (< 1) are putatively neutral.

To identify genomic regions under strong evolutionary constraint in the kākāpō we obtained 135 published bird genomes from NCBI (Figure S16). We used TimeTree[81] to estimate the divergence times among these genomes as described above. Each of these genomes were then converted into fastq-format (50 bp reads) and realigned against the kākāpō assembly using BWA mem v0.7.13,[48] slightly lowering mismatch and gaps penalty scores (-B 3, -O 4,4). Additionally, we filtered out all reads from the processed bam files aligning to more than one genomic location using Samtools.[49] Next, we converted each alignment file to fasta-format using htsbox v1.0 -R -q 30 -Q 30 -l 35 -s 1. GERP++ was then used to calculate conservation scores for each site in the genome for which at least three bird species could be accurately aligned to the kākāpō reference (Figure S17). The kea genome (*N. notabilis*) alignment was used for the ancestral allele inference.[91,92]

To estimate the mutational load of each individual we obtained the total number of derived alleles stratified by GERP-score within highly conserved regions of the kākāpō genome (excluding sites with missing genotypes). The individual relative mutational load was then calculated as the sum of the number of all derived alleles above GERP-score of two (as these are considered to be deleterious) multiplied by their GERP-score, divided by the total number of derived alleles by individual (including those below a GERP-score of one). Higher values indicate that a relatively larger proportion of derived alleles is found at conserved genomic sites, thus indicating higher mutational load. We statistically compared GERP-scores between mainland and Stewart Island kākāpō using a Welch's two-sample t tests in R.[89]

Second, we estimated mutational load in coding regions for mainland and Stewart Island kākāpō genomes using SNPeff v4.3.[68] We used our dataset filtered for missing genotypes (880,370 SNPs) to avoid any bias due to sequencing stochasticity when estimating the difference in mutational load between populations and the annotation of 15,699 genes from the MESPA pipeline (see Methods S1) for this analysis. In order to avoid a reference bias when identifying synonymous and non-synonymous variants, we replaced the reference allele with the ancestral allele by using kea (*N. notabilis*) as reference and using a custom script as described above. After replacing the reference allele, we obtained a total of 406,510 SNPs.

We generated a database for kākāpō using the protein sequences extracted from our annotation. We further removed any gene model with in-frame STOP codons using the -V option of gffread from the cufflinks v2.2.1[44,45] package. We first identified putative deleterious variants in four different impact categories as defined in the SNPeff manual: a) *Low*: mostly harmless or unlikely to change protein behavior (i.e., synonymous variants); b) *Moderate*: non-disruptive variants that might change protein effectiveness (i.e., missense variants; Table S4); c) *High*: variant assumed to have high (disruptive) impact in the protein, probably causing protein

truncation, loss of function (LoF) or triggering nonsense mediated decay (i.e., stop codons, splice donor variant and splice acceptor, start codon lost; Table S5); d) *Modifier*: usually non-coding variants or variants affecting non-coding genes, where predictions are difficult or there is no evidence of impact (i.e., downstream or upstream variants).[68] Next, we identified the number of variants in these four categories separated by homozygous and heterozygous state. Because we only used sites covered in all individuals, we counted the number of variants in these four categories separated by homozygous and heterozygous state and did not need to use bootstrapping of allele counts. We then compared the number of each of these variants in mainland and Stewart Island kākāpō using a Welch's two-sample t tests in R.[89]

We then estimated the difference in frequency of variants of all impact categories listed above between mainland and Stewart Island kākāpō using a similar approach to the one described by Xue et al.[25] and van der Valk et al.[10] For each category of variants, we calculated at each site *i* the observed allele frequency in Population x as $f^x_i = d^x_i / n^x_i$, where n^x_i is the total number of alleles called in population x and d^x_i is the total number of called derived alleles. Similarly, we define f^y_i for population y. For each C category of variants we estimated:

$$\mathrm{Freq}_{\mathrm{pop-x}}(C) = \sum_{i \in C} f^x_i\left(1 - f^y_i\right)$$

We then calculated the $R_{xy} = \mathrm{Freq}_{\mathrm{pop\text{-}x}} / \mathrm{Freq}_{\mathrm{pop\text{-}y}}$ ratio, where a value of 1 corresponds to no change in frequency, > 1 a decrease in frequency in population y relative to population x and < 1 to an increase in frequency in population y. relative to population x We estimated the variance in the R_{xy} ratio by running a Jackknife approach in blocks of 1000 from the set of sites in each category of mutation. The R_{xy} ratio only included sites where at least one out of all alleles is derived in both populations.

To check for annotation bias, we performed the same analysis using a consensus mainland historical genome. We modified our modern high quality genome by changing SNPs and indels to the historical state using the genome polishing software Pilon v.1.22[69] with quality filtering (–minmq 20 –minqual 20) and by mapping merged reads from individual LEI2, which had the highest coverage of the historical genomes (Table S1) using BWA mem v0.7.13.[48] A second annotation for the historical genome was generated with the MESPA pipeline (see Methods S1), by using the historical genome as the reference with all other steps identical. The raw data was then mapped to this consensus and the variant calling performed as described above. After filtering for missing genotypes, we obtained 834,420 SNPs. Finally, we also replaced the reference allele with the ancestral allele by using kea (*N. notabilis*) in order to avoid reference bias as described above obtained a total of 371,886 SNPs. Results were consistent with those based on data mapped to the modern assembly (Figure S21).

Purging of recessive deleterious variants (i.e., LoF alleles) is expected to lead to different signatures in homozygous (i.e., runs of homozygosity; ROH) and non-homozygous tracts within individuals. Since the individuals in this study were adults when sampled, recessive LoF variants with a deleterious effect on viability or survival in early infancy should thus be less common in homozygous tracts, where they have been exposed to purifying selection, than elsewhere in the genome. To test this hypothesis, we measured the number of LoF variants sites in homozygous and heterozygous portions of the genome and controlled for differing amounts of homozygosity within individuals by normalizing the rates of LoF variant sites by the rates of synonymous homozygous variant sites in the same regions obtained from the SNPeff output. We then assessed significance of the difference between relative rates of LoF variants in the homozygous and non-homozygous portions of the genomes using a paired t test in R.[89]

Gene Ontology

We performed a functional analysis for genes with LoF variants identified in SNPeff and based on Jane's annotation (Table S6). We obtained the gene IDs associated with each LoF allele identified in the SNPeff analysis from our functional annotation. We then assessed the functional classification of these LoF variants with a Gene Ontology analysis in Panther v16.0[70] using chicken as reference set. Because identifying mutational load in birds that survived the peak of the 1990s bottleneck is highly valuable to guide future conservation actions for kākāpō, we performed this analysis only on survivor birds (i.e., 35 Stewart Island birds and Richard Henry; Tables S6 and S7).

Forward simulations

Since the effect of drift and purifying selection are dependent on N_e[8], we estimated changes in mutational load under contrasting demographic scenarios to assess their respective roles in declining populations. To test whether our results were consistent with purging of deleterious mutations in the Stewart Island population, we performed forward simulations recapitulating the demographic history of mainland and Stewart Island kākāpō. We also simulated scenarios for hypothetical stable and severely bottlenecked populations to model a weak and strong effect of drift, respectively.

We performed individual-based simulations with SLiM 3[71] using the non-Wright-Fisher (non-WF) implementation. As opposed to Wright-Fisher models, which operates under a more restrictive set of assumptions, non-WF models are fully customizable in terms of mate choice, reproduction, survival and population regulation, which allowed us to approximate the kākāpō life-history traits in a more realistic way based on Powlesland et al.[4] We controlled the sex ratio to simulate the observed skewed sex ratio of kākāpō in the wild (~2:1 in favor of males). We controlled time to sexual maturity by only allowing individuals to reproduce after females reached sexual maturity between 7 and 11 years old and males slightly sooner, between 5 and 7 years old. We simulated the known variance in reproductive success by allowing more experienced males (i.e., older males) to form pairs more readily as a function of

their age. Pairs produced clutches in accordance with clutch-sizes observed in the wild, using random draws from a normal distribution (mean = 3, sd = 1.5) each pair produced between two and four individuals, and rarely more than four and less than two (including zero to represent inviable eggs). This mating scheme revealed that in our simulations, approximately a third of the individuals produced all the offspring in a given generation. Therefore, we simulated 2.8 times more individuals than our target effective population size. In non-WF simulations generations are overlapping (as in nature) and the average generation time is an emerging property of the simulation in function of the life-history parameters used. We recorded the full genealogy of 500 simulations steps and estimated that in average the distance between parents and offspring nodes was of ~16 (sd = 2) simulation steps. This is remarkably consistent with the estimated generation time for kākāpō, estimated around 15 years. Thus, each simulation step can be thought as one year (the total simulation time was 25,000 years) and the generation time in our simulation to be in average 16.5 years.

We simulated 3,291 genes across the 23 fully assembled chromosome in relative proportions and positions as observed in the genome assembly, representing 20% of the total kākāpō exome. Each in-silico gene had a length of 1.5kb adding to a total amount of 4,936,500 base pairs simulated for each individual. We used a per-base, per-generation mutation rate of $1.33\text{x}10^{-08}$. A recombination rate of $1\text{x}10^{-9}$ was used between genes, but no recombination was allowed within genes. Neutral and deleterious mutations occurred at a relative proportion of 1:2.31.[72] Selection coefficients of deleterious mutations were drawn from a gamma distribution (mean = −0.024, sd = 0.14), and simulations were performed independently for fully recessive (h = 0), partially recessive (h = 0.25) or additive (h = 0.5) dominance coefficients.

We simulated four distinct scenarios that spanned 25,000 years and that modeled distinct population trajectories since the LGM with N_e estimates from the PSMC used as priors: (i) a *Stewart Island* scenario modeled a decline to a long-term N_e of ~1,000; (ii) a *Mainland* scenario modeled a decline to a long-term N_e of ~6,000; (iii) an *Extreme* scenario modeled a sustained LGM decline to a long-term N_e of ~100 to specifically simulate a strong effect of drift; (iv) a *Stable* scenario modeled a constant N_e of ~10,000 and was used as a control, where the effect of drift should be weak.

We first performed a burn-in simulation step to obtain a fully coalesced population. Since our initial population size of N ~28,000 with overlapping generations could take a very long time to reach coalescence, we sped-up this stage of the simulation by scaling-down population size and scaling-up recombination/mutation rates and selection coefficients by a factor of 10 as recommended in the SLiM 3 manual. We ran the burn-in simulation for 100,000 steps and collected the entire genealogy by the means of tree-sequence recording[93] to confirm the tree had a single root with pyslim (i.e., has reached full-coalescence[94]). We then loaded the tree-sequence to start a new simulation where the scaling factors were removed. We first ran 10,000 generations and kept track of the trend of nucleotide diversity to confirm the scaling change had not disrupted the mutation-selection equilibrium (Figure S27). After 10,000 steps we varied the carrying capacity of the simulation to follow the different trajectories of our demographic scenarios for 25,000 steps (Figure 4) . We randomly sub-sampled 200 individuals from the last simulation step to compare the same sampling effort across all scenarios and models. We counted derived mutations for mutation classes of weakly deleterious ($-0.001 \leq s < 0$), mildly deleterious ($-0.01 \leq s < -0.001$) and strongly deleterious ($s < -0.01$) selection coefficients. We calculated additive genetic load as in Pedersen et al.[95] by adding the sum of selection coefficients for homozygous mutations and the sum of selection coefficients times the dominance coefficients for heterozygous mutations.

Cell Genomics, Volume *1*

Supplemental information

Population genomics of the critically endangered kākāpō

Nicolas Dussex, Tom van der Valk, Hernán E. Morales, Christopher W. Wheat, David Díez-del-Molino, Johanna von Seth, Yasmin Foster, Verena E. Kutschera, Katerina Guschanski, Arang Rhie, Adam M. Phillippy, Jonas Korlach, Kerstin Howe, William Chow, Sarah Pelan, Joanna D. Mendes Damas, Harris A. Lewin, Alex R. Hastie, Giulio Formenti, Olivier Fedrigo, Joseph Guhlin, Thomas W.R. Harrop, Marissa F. Le Lec, Peter K. Dearden, Leanne Haggerty, Fergal J. Martin, Vamsi Kodali, Françoise Thibaud-Nissen, David Iorns, Michael Knapp, Neil J. Gemmell, Fiona Robertson, Ron Moorhouse, Andrew Digby, Daryl Eason, Deidre Vercoe, Jason Howard, Erich D. Jarvis, Bruce C. Robertson, and Love Dalén

Supplemental Information

Population genomics reveals the impact of long-term small population size in the critically endangered kākāpō

*Contact lead: nicolas.dussex@gmail.com; ejarvis@mail.rockefeller.edu; bruce.robertson@otago.ac.nz; love.dalen@nrm.se

Contents

Methods S1

1.1 DNA extraction

1.2 Library preparation for *De-novo* assembly

1.3 *De-novo* assembly generation and curation

1.4 Genome annotations

1.5 Generation time for demographic reconstructions

Figures S1-S27

Methods S1

1.1 DNA extraction

For all modern samples, DNA extractions were performed using phenol-chloroform[1], including for the PacBio and 10X genomics libraries for the *de-novo* assembly. DNA isolated for generating Bionano libraries was mixed and centrifuged at 2,200 rcf for 5 minutes at 4°C. The resulting pellet was re-suspended in 100uL of buffer containing 10 mM Tris (pH 7.2), 50 mM EDTA, and 2 mM NaCl. The cell suspension was embedded into four 0.8% agarose plugs, targeting a 1:1, 1:2, 1:4, and 1:8 titration. Agarose plugs were then treated with Puregene proteinase K and RNAse A, washed, treated with agarase, and drop dialyzed, as described by the Bionano Prep Blood DNA Isolation Protocol (Document number 30033).

For historical samples, we extracted DNA from the toepads of the 13 historical birds (Table S1) using a DNeasy Blood & Tissue Kit (Qiagen, Hilden, Germany). These samples were selected based on high endogenous DNA content (i.e., 75.9-91.4%; Table S1) which was estimated with a

screening sequencing step where sequencing reads were mapped to the kākāpō mitogenome as described in[2]. Appropriate precautions were taken to minimize the risk of contamination in historical samples[3].

1.2 Library preparation for *De-novo* assembly

For the PacBio sequencing, Jane's genomic DNA was sheared using the Megaruptor (Diagenode, Denville, NJ), followed by SMRTbell library preparation according to the manufacturer protocols (Pacific Biosciences, Menlo Park, CA). Two libraries were generated with size selection (Sage Science, Beverly, MA) cut offs of 15kb and 30kb, resulting in libraries with average insert sizes of 40kb and 43kb, respectively. Sequencing was performed in 2016 on the PacBio RSII using P6-C4 sequencing chemistry and 10-hour acquisition times per run. A total of 127 SMRT Cells were run, resulting in ~90 Gb of total sequence data, with an insert size N50 of 13kb.

For Bionano optical mapping using the genomic DNA was fluorescently labeled with the enzymes DLE-1. The labeled DNA was run on a Saphyr instrument according to the Bionano Prep Direct Label and Stain (DLS) (document number 30206) and Bionano Prep Labeling NLRS (document number 30024) protocols.

1.3 *De-novo* assembly generation and curation

The kākāpō assembly was generated with the Vertebrate Genomes Project (VGP) v1.6 assembly pipeline[4]. Contigs were generated from PacBio subreads using FALCON-Unzip with 1 round of Arrow polishing using the DNAnexus FALCON 5.1.1 and FALCON-Unzip 1.0.2 pipelines[5]. This resulted in an initial set of primary contigs, which represent a pseudohaplotype, and a secondary set of haplotigs containing the alternative form of heterozygous alleles. The primary contig set was then iteratively scaffolded with 10X Genomics linked reads using scaff10x 2.0 (git 4.28.2018; https://github.com/wtsi-hpag/Scaff10X). Bionano optical maps were generated into in-silico cmaps using Bionano Solve v3.2.1[6] using non-haplotype aware arguments. One-enzyme scaffolding was performed with default parameters Hi-C libraries were made using the Arima-HiC Kit (P/N: A510008). Existing contigs and scaffolds were arranged into chromosome-level scaffolds using the Illumina paired end reads with Salsa 2.0[7] mapped with Arima mapping pipeline (https://github.com/ArimaGenomics/mapping_pipeline; Fig. S1).

Additional rounds of consensus polishing were conducted by mapping all the PacBio subreads and 10X reads to both the primary and alternative sets. One more round of Arrow polishing was conducted with PacBio reads using smrtanalysis v5.1.0.26412. For final polishing, the 10X linked reads were aligned with Longranger v2.2.2[8], and erroneous bases (variants) were called with Freebayes v1.2.0[9] before correction with bcftools v1.8[10]. During curation, assignment of alternative alleles in the primary assembly was further evaluated by Purge Haplotigs bitbucket 7.10.2018 to reduce artefactual duplication[11]. All scripts used in the assembly pipeline are publicly available from https://github.com/VGP/vgp-assembly.

The resulting kākāpō assembly was subjected to contamination screening and manual curation to detect and correct remaining assembly issues. The contamination screen detected and removed 34 scaffolds that consisted of *E. coli* sequence only (2,243kb total). For manual assembly curation, all available sequencing and mapping and previous assembly data were compared to the assembly using gEVAL[12]. Alignment discordances were manually assessed and resolved by breaking and re-joining scaffolds. A HiC 2D map generated with Juicer and visualized in Juicebox[13] allowed for further scaffold correction and super-scaffolding in order to bring the assembly to chromosome scale (Fig. S1). HiC-led changes were verified against Pacbio, Bionano, 10X link reads and other data in gEVAL.

The manual curation resulted in 19 scaffold rearrangements and the removal of 99 sequence regions that were identified as haplotypic duplications. This reduced the initial assembly length by 14.5Mb (1.2%) and the scaffold number from 229 to 99, whilst decreasing the scaffold NG50 from 88.2Mb to 83.2Mb to correct over-scaffolding. NG50 contig was of 9.1Mb. 99.2% of the final total assembly sequence of 1.17 Gb was assigned to the identified 26 chromosomes (24 autosomes and two sex chromosomes, see below), which were named according to their size (Figs. S2-3). Only 67 scaffolds remain unplaced.

We then identified Z and W chromosomes from the assembled genome by blasting all scaffolds against the Z-chromosome of zebra finch (v3.2.4, *Taeniopygia guttata*; GenBank: GCA_000151805.2) and W-chromosome of chicken (v5.0, *Gallus gallus*, GenBank: GCA_000002315.5) using BLAST+ 2.5.0[14]. The BLAST+ parameters were set as: -evalue = 1e-10; -word_size = 15; -max_target_seqs = 1000. We then excluded the identified Z chromosome (CM013763.1; 101.23Mb) and W chromosome (CM013773.1; 35.7Mb), from all downstream analyses in order to avoid bias associated with analyses relying on heterozygosity estimates. We

also visually examined the coverage across the genome in the bam files generated for males and females to check that the identified chromosomes were in fact the Z and W using Qualimap v2.2.1[15]. Males had on average ~15X and ~0X for the Z and W chromosome, respectively; and females had on average ~7X and ~7X for the Z and W chromosome, respectively. We then identified CpG sites using a custom script masking CG sites and masked repetitive elements in the genome assembly using RepeatMasker v4.0.7[16] (http://repeatmasker.org) applying the repeat element library of the *aves* database.

We also examined synteny between the *de-novo* kākāpō assembly and both zebra finch (v 3.2.4, *Taeniopygia guttata*; GenBank: GCA_000151805.2) and chicken (v5.0, *Gallus gallus*, GenBank: GCA_000002315.5) assemblies. The alignments were done with Mashmap2[17] at 150Kb resolution and 75% identity (Figs. S2-3).

The mitogenome was assembled using a dedicated mitoVGP v1.0 pipeline complementing both PacBio long-reads and 10x linked-reads using the same WGS data employed for the nuclear genome. Briefly, mitochondrial reads were identified in WGS long reads by similarity with a previously reported mitogenome assembly of the kākāpō (NC_005931.1) using BlasR v5.3.2[18]. Reads were assembled with Canu v1.8, the assembly polished with long reads using variantCaller v2.2.2 (Arrow), and further polished with short reads using Bowtie2 v2.1.0[19] and Freebayes v1.2.0[9]. Overlapping ends were trimmed using a custom script (https://github.com/GiulioF1/mitoVGP/tree/master/pipeline_v1.0), a second round of short-read polishing was performed, and the sequence was further trimmed with the same script. The final consensus sequence was manually oriented to start with trnF using annotation from MITOS2 (PMID: 22982435).

The final resulting assembled genome was submitted to the GenBank archive in two parts: the primary pseudohaplotype containing the best representation of the haploid genome (GCA_004027225.1/GCF_004027225.1), and the alternate pseudohaplotype containing all the alternative alleles (GCA_004011185.1).

1.4 Genome annotations

Three types of annotations were built for the kākāpō *de-novo* genome assembly. These annotations generated between 15,699 and 16,060 high-quality protein-coding gene models.

First, because RNA data was not available at the time of the initial analyses, we annotated the kākāpō *de-novo* genome assembly using publicly available protein sequences datasets and used this annotation for the analysis of mutational load in coding and non-coding regions. We first assessed the quality of the annotation using different reference protein sets with the MESPA pipeline[20]. We collapsed reference protein sets for the kea (*Nestor notabilis*; GenBank: SRP029311), which is closely related to kākāpō and zebra finch (*Taeniopygia guttata*; GenBank: GCA_000151805.2) to 90% coverage following Uniprot90 guidelines using a custom script. This results in each protein cluster being composed of sequences with at least 90% sequence identity to, and 80% overlap with, the longest sequence. In that way, we discarded isoforms of the reference datasets. We then used MESPA to extract the gene models in kākāpō with at least 90% length coverage to each set of reference proteins and to generate an annotation in gff format. We extracted 85% (13,175 out of 15,342) high quality kākāpō protein models (i.e., aligning to 90% of their expected length) using zebra finch as a reference protein set, and 86% (10,159 out of 11,806) using kea as a reference protein set.

Next, we refined the annotation for the kākāpō assembly using the BRAKER2 v2.1.1 pipeline[21–23]. BRAKER is an automated method for accurate gene structure annotation, which allows fully automated training of the gene prediction tools GeneMark-EX[24,25] and AUGUSTUS[26–28] for gene model prediction and can incorporate protein homology information from closely related species for training purposes.

In order to generate a high quality protein dataset for training in BRAKER2, we predicted proteins for kākāpō using the zebra finch proteome[29] (ftp://ftp.ensembl.org/pub/release-89/fasta/taeniopygia_guttata/pep/Taeniopygia_guttata.taeGut3.2.4.pep.all.fa.gz), then filtered these for high quality models by comparing them to the flycatcher proteome[30] (ftp://ftp.ensembl.org/pub/release-89/fasta/ficedula_albicollis/pep/Ficedula_albicollis.FicAlb_1.4.pep.all.fa.gz).

The resulting proteins after filtering, were considered high quality kākāpō protein sequences, which were then used as training for gene model predictions by BRAKER2. Both the zebra finch and flycatcher proteomes were first clustered to the longest representative of each set of proteins having at least 90% amino acid identity, using cd-hit v4.6.1 (-c 0.90 -T 16 -aS 0.8 -M 0), reducing the protein dataset from 18,204 to 15,363 and 15,983 to 15,372 proteins, for zebra finch and flycatcher, respectively, in order to remove recent duplicates and isoforms. Using the

zebra finch clustered proteome as input, along with the kākāpō genome (ftp://ftp.ncbi.nlm.nih.gov/genomes/all/GCA/004/027/225/GCA_004027225.1_bStrHab1_v1.p/GCA_004027225.1_bStrHab1_v1.p_genomic.fna.gz), proteins were predicted with the exon aware, protein to genome aligner SPALN2[31].

The resulting kākāpō proteins where then filtered to only retain those with a start codon and no internal stop codons (n=8,784). These were then further filtered to only retain those that when aligned to the flycatcher filtered proteome, covered at least 95% of the flycatcher protein length (n=6,657), and then this filtered kākāpō protein set was then used as a high quality protein set for BRAKER2 training.

Soft masking of the repeat content in the genome was performed using Red[32], which masked 32.33% of the genome (375,531,262 bp). Alignment of the filtered kākāpō protein dataset against the soft masked genome was performed by GENOME THREADER v1.7.0, with the option "-species "chicken" set in order to improve splice-site recognition[33]. The output of the GENOME THREADER alignment was then used by BRAKER2 for gene prediction training (settings: --prg=gth --gth2traingenes --trainFromGth –softmasking).

We then extracted CDSs and protein sequences from this annotation using cufflinks v2.2.1[34,35] gffread command using the -V option to exclude genes with in-frame STOP codons. We identified 16,171 kākāpō gene models with a mean length of 1,514bp (Median=672; min=50; max=26,940) to be used in downstream analyses.

Finally, we performed a functional annotation of these gene models using the eggNOG-mapper v4.5.1[36], which uses fast orthology assignments upon precomputed protein clusters within a phylogenetic context[36]. We used '*Aves*' as taxonomic scope and the 'Restrict to one-to-one' and the 'Use experimental terms only' to prioritize precision, quality of matches and also to remove multiple matches which could represent pseudogenes. Overall, we obtained 15,699 annotated gene models.

Secondly, the Ensembl gene annotation system[37] was used to annotate the kākāpō *de-novo* genome assembly. Multiple lines of evidence were used: MinION kidney and brain long reads (SRX6605068, SRX6605069), Pacbio Iso-Seq liver long reads (SRX5842929), Illumina kidney and brain short reads (SRX6605066, SRX6605067), splice aware protein-to-genome alignments of vertebrate proteins classed as either protein existence level 1 (experimental evidence at the protein level) or level 2 (experimental evidence at the transcript level) from UniProt[38] and

coordinate mapping of human reference annotations to kākāpō via a pairwise whole genome alignment.

Long read data were mapped to the genome using Minimap2[39] (PMID: 29750242) with the recommended settings for Iso-Seq and Nanopore data. Short read data were initially mapped via BWA[40] and then locally re-aligned in a splice-aware manner via Exonerate[41]. Protein-to-genome alignments were carried out via GenBlastG[42]. Annotation mapping from human was carried out via a pairwise alignment using LastZ[43] and subsequent exon coordinate mapping and transcript reconstruction via both in-house software and CESAR v2.0[44].

Due to the high error rate of the Nanopore data, post mapping error correction was employed to maximize the number of usable mappings. Intron/exon boundaries that were non-canonical or deemed low frequency (five or fewer observations across all mappings at a locus) were replaced with high frequency boundary coordinates (greater than five observations) within a 50bp edit distance. High frequency boundary observations were determined both from canonical boundary observations from the Nanopore mapping themselves and also from the alignments of the short-read data. A similar strategy was employed to remove likely artificial gaps of 200bp or less from exons described by the Nanopore data. In these cases, low frequency potential gaps between two adjoining exons were filled in based on high frequency observations of single exons with the same terminal boundary coordinates.

In order to determine the protein coding genes and transcripts, all evidence lines were analysed at each locus. ORF likelihood was determined by aligning the ORF translation against known vertebrate proteins. Preference was given to transcript isoforms generated from the transcriptomic data that had high coverage matches to known vertebrate proteins. For loci where the transcriptomic data was not available or highly fragmented, gap filling was done using the splice-aware protein-to-genome alignments and annotation mappings from human via a pairwise whole genome alignment, with preference given to resulting ORFs that showed a high percent coverage and identity when re-aligned to the original evidence. A total of 16,037 protein-coding genes were identified with 24,520 transcript isoforms.

Pseudogenes called from the protein-coding loci that did not have transcriptional support, were analysed for evidence of structural abnormalities, such as absence of a start coding, non-canonical splicing, unusually small intron structures (< 75bp) or excessive repeat coverage. Loci with two or more such abnormalities were reclassified as pseudogenes. Single exon loci that

showed matched a multi-exon ORF elsewhere in the genome with greater than 80 percent coverage were reclassified as retrotransposed pseudogenes. At total of 103 pseudogenes and 9 retrotransposed pseudogenes were identified.

Long non-coding loci were called using both the short and long read transcriptomic data. Potential lncRNAs were initially called from transcript models where no BLAST[28] hit to a known vertebrate protein was found. The resulting set was then filtered to remove and transcripts with genomic overlap with a protein-coding locus. An addition filter was then applied to remove single exon loci (due to the abundance of transcriptional noise generally found in long read data). We identified 3,578 long non-coding loci with 4,030 transcript isoforms.

Small non-coding loci were predicted using data from miRbase[45] and Rfam[46] and scanning against the genome (described in more detail in the Ensembl annotation system[37]). Initial hits were then filtered based on predicted ability to form stem-loop secondary structures. This resulted in 465 small non-coding gene predictions. The kākāpō gene annotation is due to be released in Ensembl release 99 (expected December 2019). For more detail on the annotation system please refer to the Ensembl annotation system[37].

Finally, the NCBI Eukaryotic Genome Annotation Pipeline[47] was used to annotate genes, transcripts and proteins on bStrHab1_v1.p (GCF_004027225.1), the primary pseudohaplotype of the assembly. Next, the assembly was first masked with WindowMasker[48]. Nearly 350 million RNA-Seq reads from kākāpō brain and kidney tissue, 700 million RNA-Seq from other species in the Psittacidae family (*Amazona ventralis*, *Aratinga solstitialis* and *Platycercus eximius*), 351,938 IsoSeq consensus reads from liver and 8.1 million Oxford Nanopore reads from brain and liver tissue were retrieved from SRA and aligned to the masked genome, along with 9,075 known RefSeq transcripts, 42,131 GenBank transcripts, and 756,464 ESTs from birds. The alignments were performed using BLAST[49] followed by the global aligner Splign[50] for all transcripts except the IsoSeq and Oxford Nanopore data, which were aligned with Minimap2[39]. In the absence of kākāpō proteins in GenBank, the proteins that were chosen as candidates for alignment to the genome by BLAST and ProSplign spanned a wide range of other birds, including, in particular, RefSeq proteins from *Columba livia*, *Parus major*, *Gallus gallus.* RefSeq proteins for *Xenopus laevis* and human were also included.

The resulting transcript and protein alignments were used as evidence to predict the structures and boundaries of gene and transcript models. *Ab initio* extension or joining/filling of

partial open reading frames in compatible frames of these preliminary models was performed by Gnomon (https://www.ncbi.nlm.nih.gov/genome/annotation_euk/gnomon/), using a hidden Markov model trained on kākāpō, in the minority of cases where the overlapping alignments did not define a complete model but the coding propensity of the CDS on the alignments was sufficiently high. RNAs were predicted with tRNAscan-SE:1.2326[51] and small non-coding RNAs were predicted by searching the RFAM 12.0 HMMs for eukaryotes using cmsearch from the Infernal package[52]. The annotation of bStrHab1_v1.p (NCBI *Strigops habroptila* Annotation Release 100) resulted in 16,060 protein-coding genes, 3,131 non-coding genes and 165 pseudogenes (see details in https://www.ncbi.nlm.nih.gov/genome/annotation_euk/Strigops_habroptila/100/).

For the Ensembl and NCBI assembly pipelines, RNA data for kākāpō was extracted from brain and kidney tissue from a female chick that died at three-days old through grinding on liquid nitrogen, tissue disruption in Trizol reagent (Thermofisher), followed by extraction of total RNA using Qiagen RNAeasy technologies. mRNA was purified from 23 µg and 11 µg of kidney and brain total RNA respectively using the Dynabeads mRNA Purification Kit (Invitrogen, U.S.A.). 124 ng and 229 ng of purified mRNA from kidney and brain, respectively, was used for library preparation with the Direct cDNA Sequencing DCS109 kit (Oxford Nanopore Technologies, U.K.). Libraries were sequenced for 48 hours on R9.4.1 MinION flow cells using two Mk1B MinION sequencers (Oxford Nanopore Technologies). Raw data were base-called using guppy_basecaller 3.1.5 in high accuracy mode (dna_r9.4.1_450bps_hac) on a NVIDIA GeForce RTX 2070 GPU.

1.5 Generation time for demographic reconstructions

It was previously thought that generation time in kākāpō was of ~25 years[53]. However, this number may be an overestimate because it is based on the average age to first reproduction in the extant population, with females recorded to have bred between 5 and 18 years while males have bred between 11 to 23 years. However, in this small population, only a few older dominant males have reproduced (Daryl Eason, DOC, pers. comm.) and many young males have thus not had the opportunity to breed yet. We thus used a shorter generation time, assuming that a time of 15 years may be more biologically realistic in a large and natural kākāpō population.

Supplemental figures

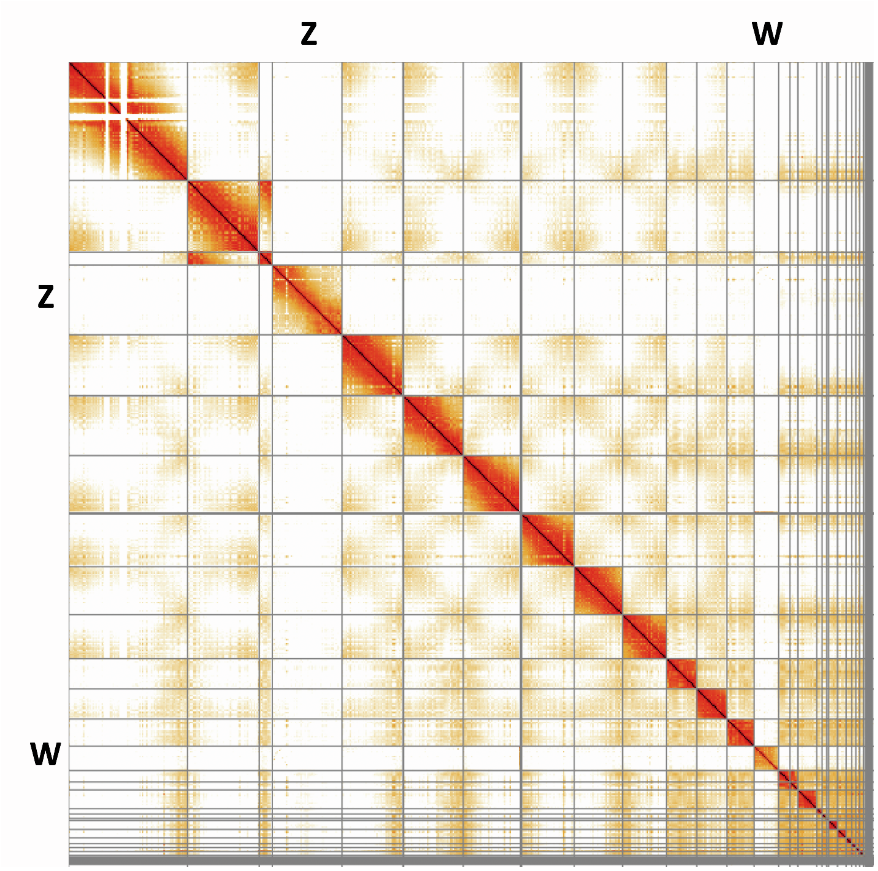

Figure S1. HiC 2D map of the curated bStrHab1 assembly, Related to STAR Methods. The assembly plot was generated using HiGlass[54]. Black lines depict scaffold boundaries. The Z and W sex chromosomes are shown in bold letters. The kākāpō chromosome assignments were based on HiC data and over 99% of the sequence was assignable to arm-to-arm chromosomes via digital karyotyping, where compartimentalization of the HiC signal is interpreted as outline for a self-contained sequence unit, (i.e., chromosome).

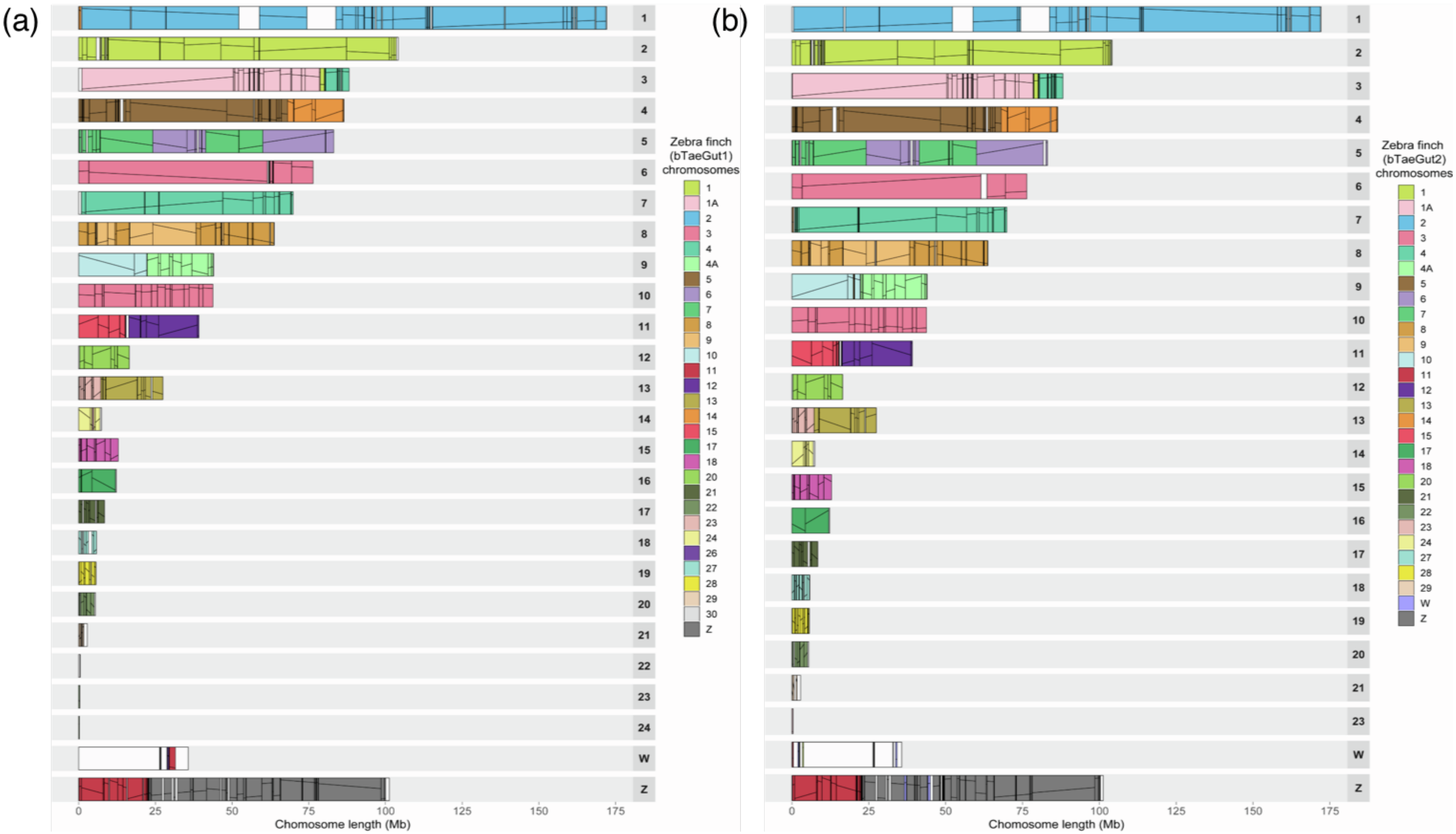

Figure S2. Ideogram of kākāpō chromosomes relative to (a) male and (b) female zebra finch, Related to STAR Methods. Numbered rectangles represent kākāpō chromosomes, and colored blocks inside represent regions of homology with zebra finch chromosomes. Lines within the colored blocks represent block orientation.

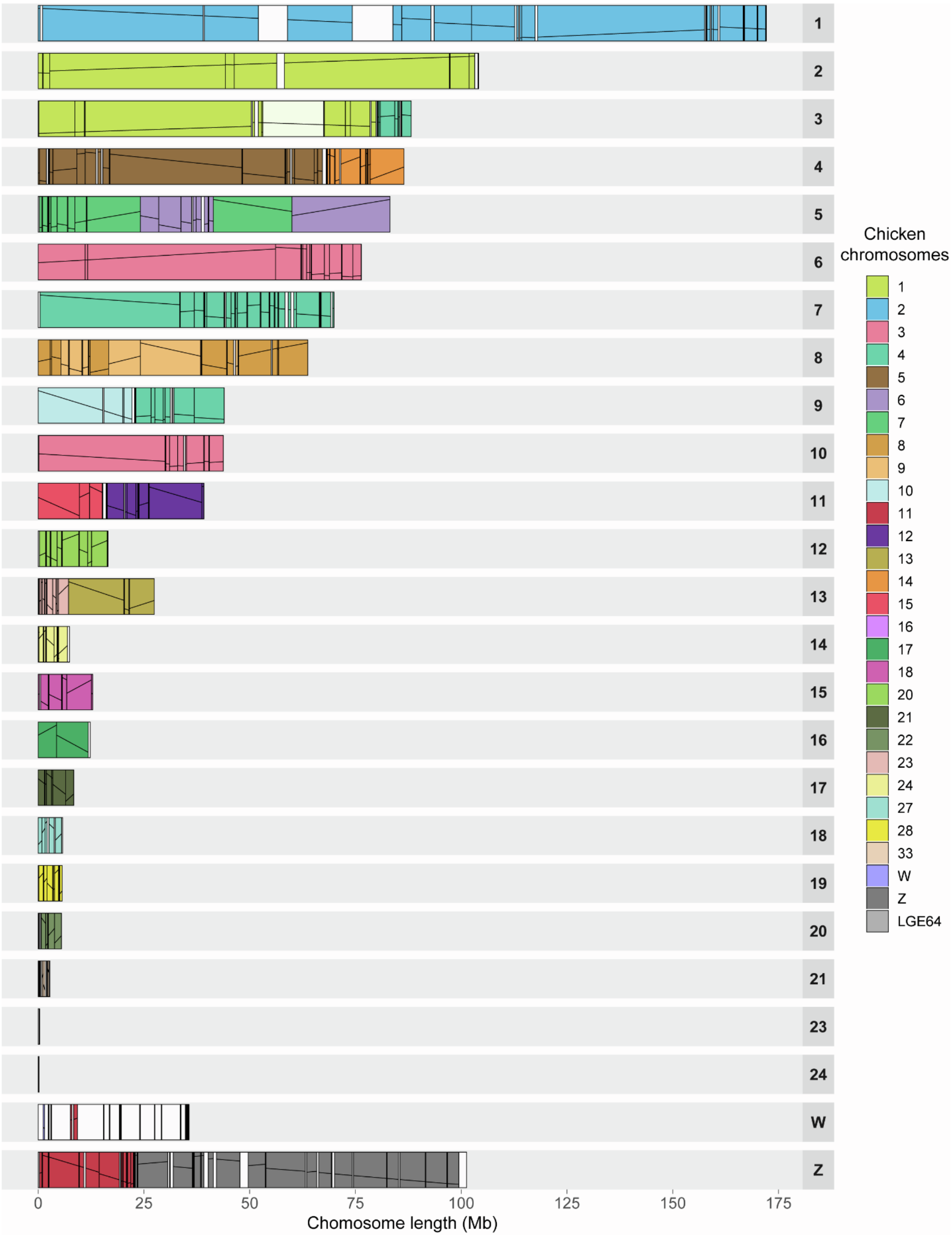

Figure S3. Ideogram of kākāpō chromosomes relative to chicken, Related to STAR Methods. Numbered rectangles represent chromosomes, and colored blocks inside represent regions of homology with chicken chromosomes. Lines within the colored blocks represent block orientation. The Z chromosome

homolog is fused with the chromosome 11 equivalent of chicken based on read and mapping data. Also, two autosomes (Chr 16 and 18) had 0 gaps each and no evidence of collapsed repeats, and all others had only 1 to 24 gaps, which may be explained by the fact that in birds most microchromosomes are acrocentric (i.e., centromere at the extremity of chromosomes).

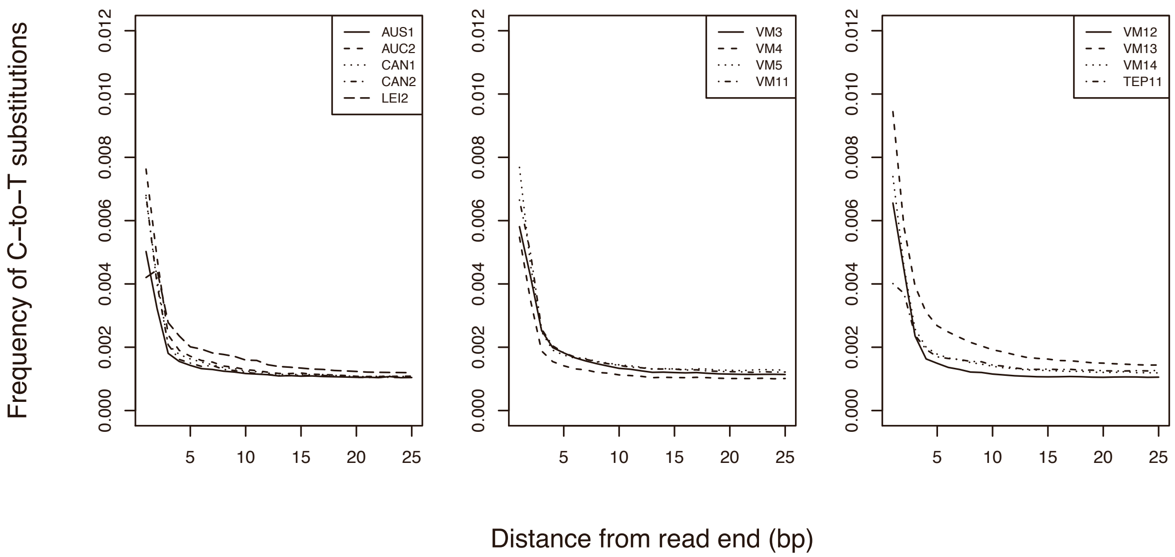

Figure S4. **DNA-damage in 13 historical genomes, Related to STAR Methods.** Fraction of C-to-T substitutions by distance from the read-end averaged over the historical genomes. The USER treatment removed the majority of typical post-mortem damage patterns, with the historical genomes showing slightly elevated levels of these substitutions.

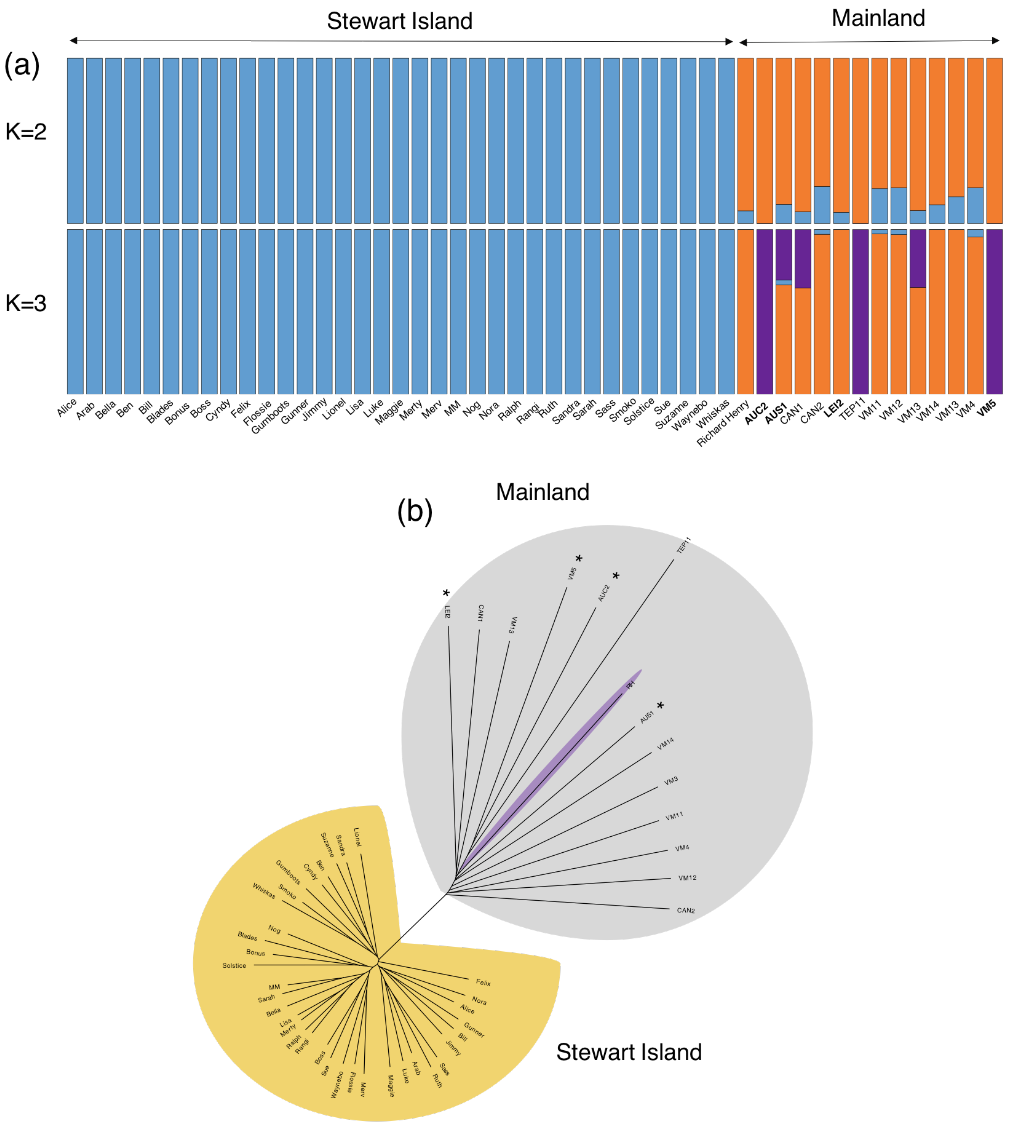

Figure S5. Population structure in 35 Stewart Island and 14 mainland kākāpō, Related to Figure 1 and STAR Methods. (a) Individual clustering assignment in ADMIXTURE. The lowest cross-validation error was obtained for K=2. Individuals in bold are museum specimen showing inconsistencies between genetic clustering and specimen labelling, potentially as a result of museum specimen mislabelling[2]. **(b)** Neighbour-joining tree with asterisks representing museum specimens showing inconsistencies between genetic clustering and specimen labelling. All mislabelled samples were analysed as part of their genetically assigned population in all downstream analyses.

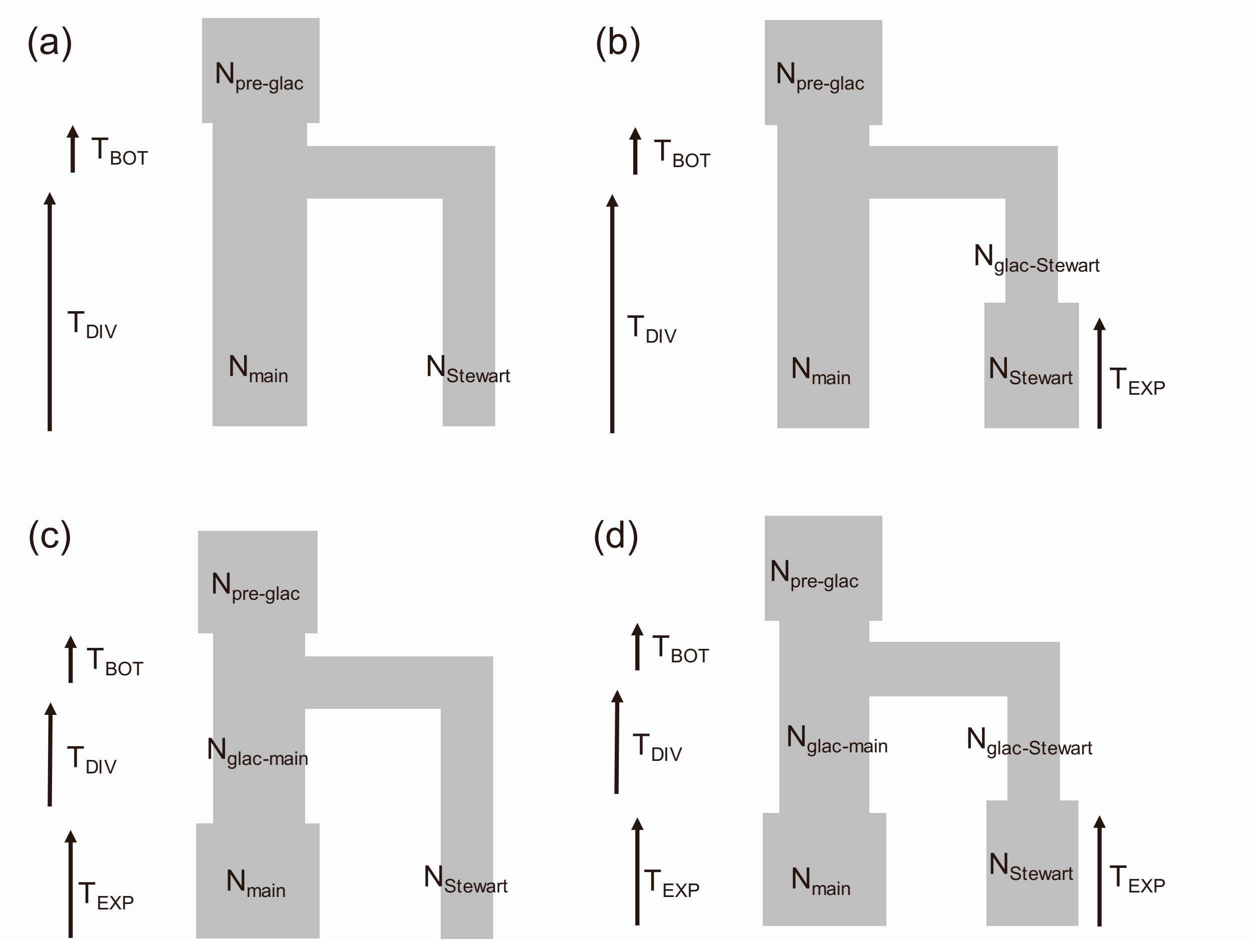

Figure S6. Demographic scenarios for mainland and Stewart Island kākāpō, Related to Figure 1 and STAR Methods. **(a)** *Post-glacial divergence*, **(b)** *Post-glacial divergence followed by Stewart Island population expansion*, **(c)** *Post-glacial divergence followed by Mainland population expansion* and, **(d)** *Post-glacial divergence followed by Stewart Island and Mainland population expansion.* Note that the recent population expansion for the Stewart and Mainland populations (T_{EXP}; STAR Methods) were not constrained in order to allow for either a bottleneck or population expansion to occur. These events are however referred to as expansions as they were supported by the best model. Scenario (d) also depicted in Fig. 1d obtained the highest support as estimated by AIC's weight (w) (Table S2).

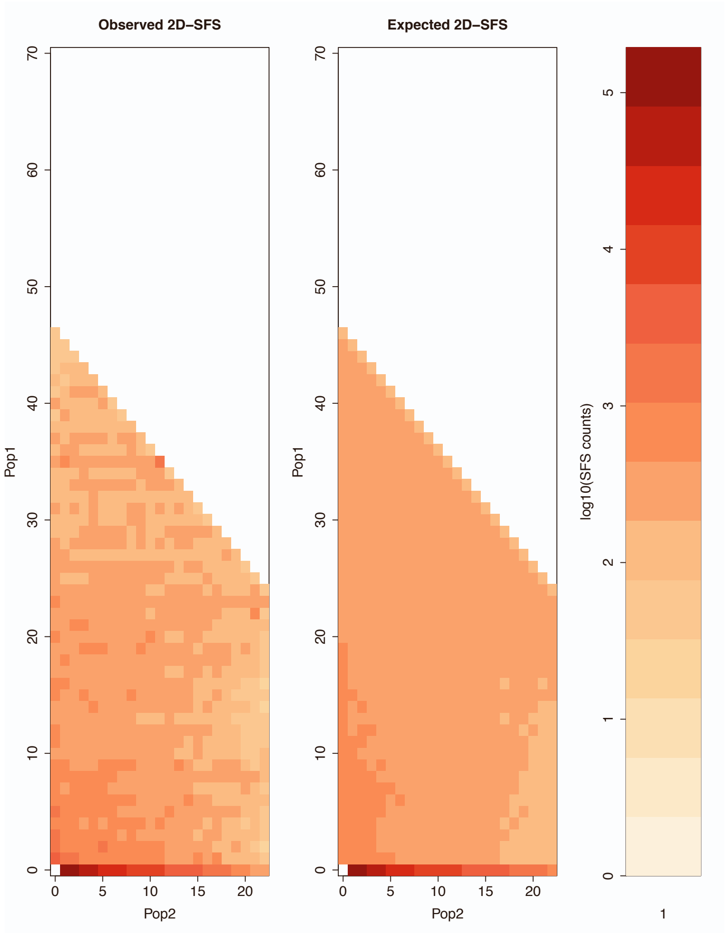

Figure S7. Comparison of observed (left panel) and expected (right panel) 2D-SFS for the preferred scenario of *Post-glacial divergence followed by Stewart Island and mainland population expansion*, Related to Figure 1 and STAR Methods. In the main two panels, each cell represents the number of alleles which occur at frequency *x* in Population 1 (Stewart Island) and frequency *y* in Population 2 (Mainland). The colored bar to the right depicts the count of allele on a logarithmic scale that occur at a x-y frequency combination in the main two panels.

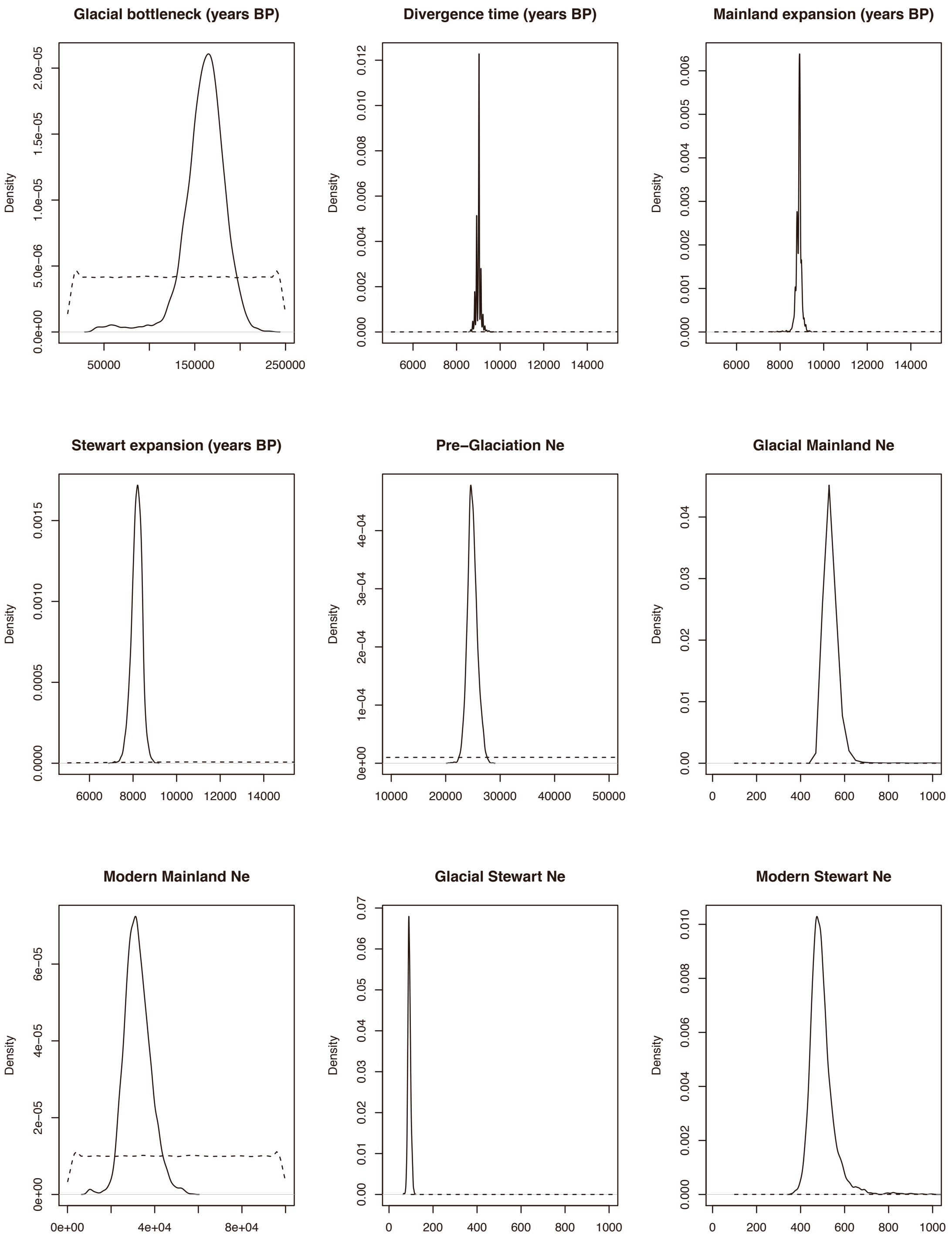

Figure S8. Posterior distributions of effective population sizes, glaciation bottleneck and divergence times and change in effective population size for the preferred scenario of *Post-glacial divergence followed by Stewart Island and mainland population expansion*, Related to Figure 1 and STAR Methods. Dashed and full lines represent prior and posterior distributions, respectively.

Table S2. Relative likelihood of the three different demographic models tested and depicted in Figure S6, Related to Figure 1 and STAR Methods. A scenario of '*Post-glacial divergence followed by Stewart Island and Mainland population expansion*' obtained most support based on AIC's weight.

**Model**	**Log MaxEstLhood**	**N of estimated parameters**	**AIC**	**Δ**	**AIC's weight (w)**
a) Post-glacial divergence	-1522664.81	7	3045343.61	126184.332	0.00E+00
b) Post-glacial divergence and Stewart Island population expansion	-1516988.68	9	3033995.35	114836.0731	0.00E+00
c) Post-glacial divergence and Mainland expansion	-1503772.70	9	3007563.41	88404.128	0.00E+00
d) Post-glacial divergence and Stewart Island and Mainland expansion	-1459568.64	11	2919159.28	0	1.00E+00

[a]Based on the best of 50 likelihood estimates of each scenario

[b]AIC_i=2d 2-ln($Lhood_i$)

[c]Δ_i =AIC_i -min(AIC)

[d] $$w_i = \frac{\exp(-0.5\Delta_i)}{\sum_r^R \exp(-0.5\Delta_r)}$$

Table S3. **Posterior distributions of parameters for the preferred scenario d)** ***Post-glacial divergence followed by Stewart Island and mainland population expansion*****, Related to Figure 1 and STAR Methods.** Times are in years BP. Note that the recent population expansion for the Stewart and Mainland populations ($T_{EXP;}$ STAR Methods) were not constrained in order to allow for either a bottleneck or population expansion to occur. These events are however referred to as expansions as they were supported by the best model.

**Parameter**	**mode**	**95% CI**	
		**Lower CI**	**Higher CI**
T_{BOT}	157,800	130,800	198,165
T_{DIV}	9,030	8,805	9,120
$T_{EXP\text{-}main}$	8,910	8,670	9,015
$T_{EXP\text{-}Stewart}$	8,205	7,740	8,505
$N_{pre\text{-}glac}$	24,714	23,466	26,529
$N_{glac\text{-}main}$	510	505	570
N_{main}	28,135	23,026	41,850
$N_{glac\text{-}Stewart}$	91	81	102
$N_{Stewart}$	485	415	568

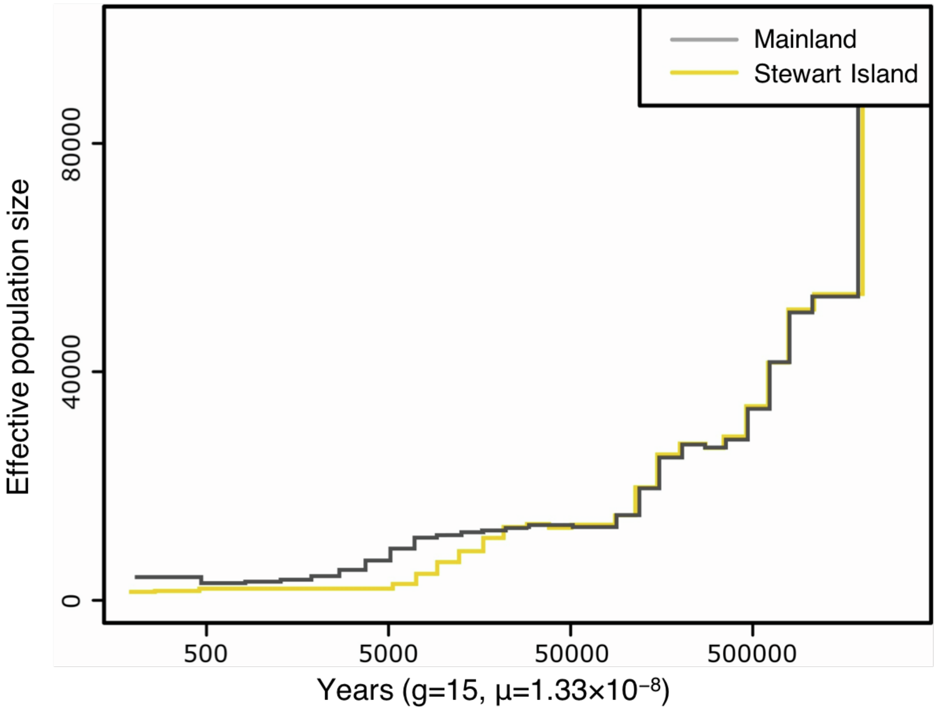

Figure S9. Past demography of kākāpō using the MSMC2 approach, Related to Figure 1 and STAR Methods. The x-axis corresponds to time before present in years on a log scale, assuming a mutation rate of 1.33×10^{-8} substitutions/site/generation and a generation time of 15 years[53]. At the end point of the curve some 300 y BP, N_e estimates for the Stewart Island and the mainland populations were ~300 and ~14,500, respectively. However, it is worth noting that N_e estimates at the end point of the curve should be taken with caution due to the low number of coalescent events[55].

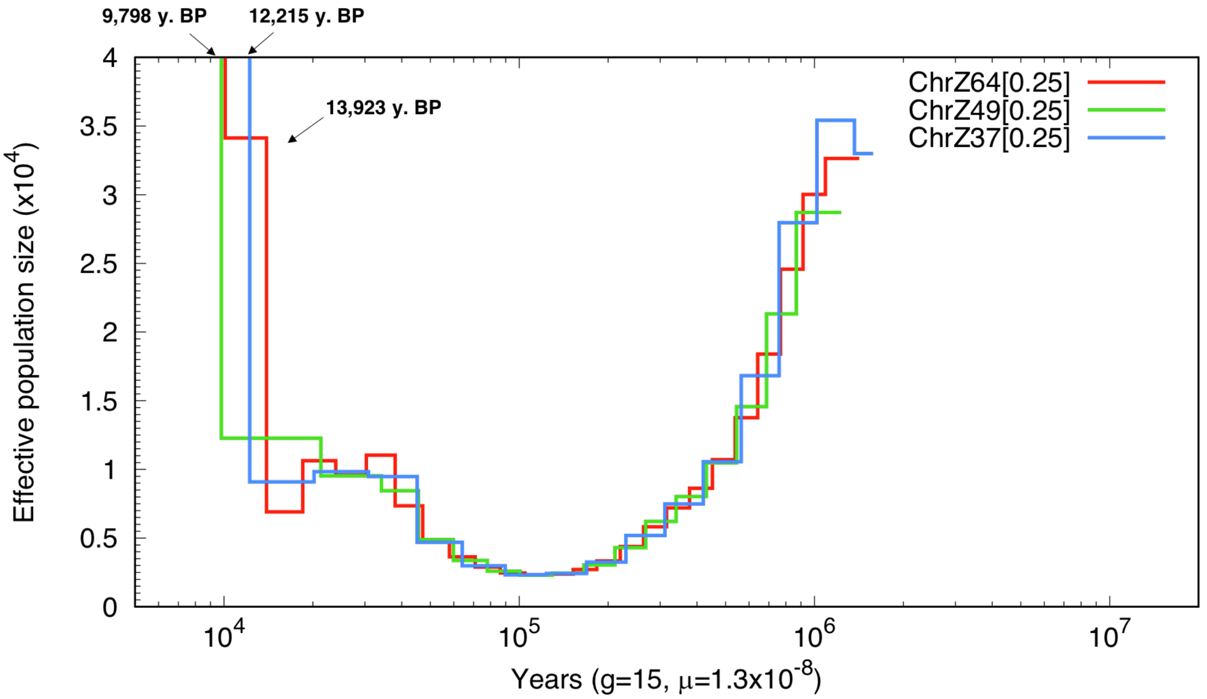

Figure S10. Comparison of divergence estimates between the ancestral population of mainland and Stewart Island using the PSMC approach, Related to Figure 1 and STAR Methods. The x axis corresponds to time before present in years on a log scale, assuming a mutation rate of 1.33×10^{-8} substitutions/site/generation and a generation time of 15 years[53]. The red, green and purple curves represent pseudo-diploid chromosome Z rescaled by factor of 0.25 (sex-chromosome/autosome ratio: 0.75) and using 64, 49 and 37 discrete intervals, respectively.

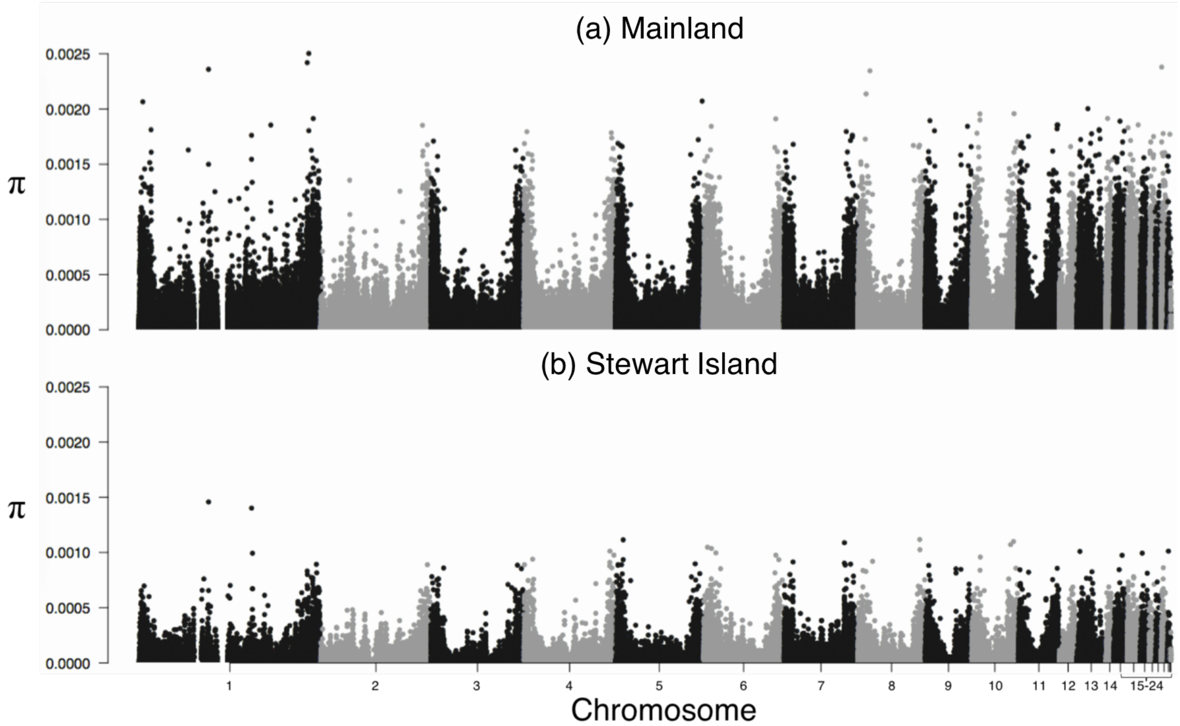

Figure S11. Genome-wide nucleotide diversity (π) estimates, Related to Figure 2 and STAR Methods. Autosomal genome-wide population-level nucleotide diversity for **(a)** Mainland (including Richard Henry) and **(b)** Stewart Island kākāpō. The higher π at the edges of chromosomes compared to the centre of chromosomes reflects the recombination landscape of the bird genome[56,57], with lower recombination rates in the centres of macrochromosomes, relative both to their edges and the micro-chromosomes[56,58].

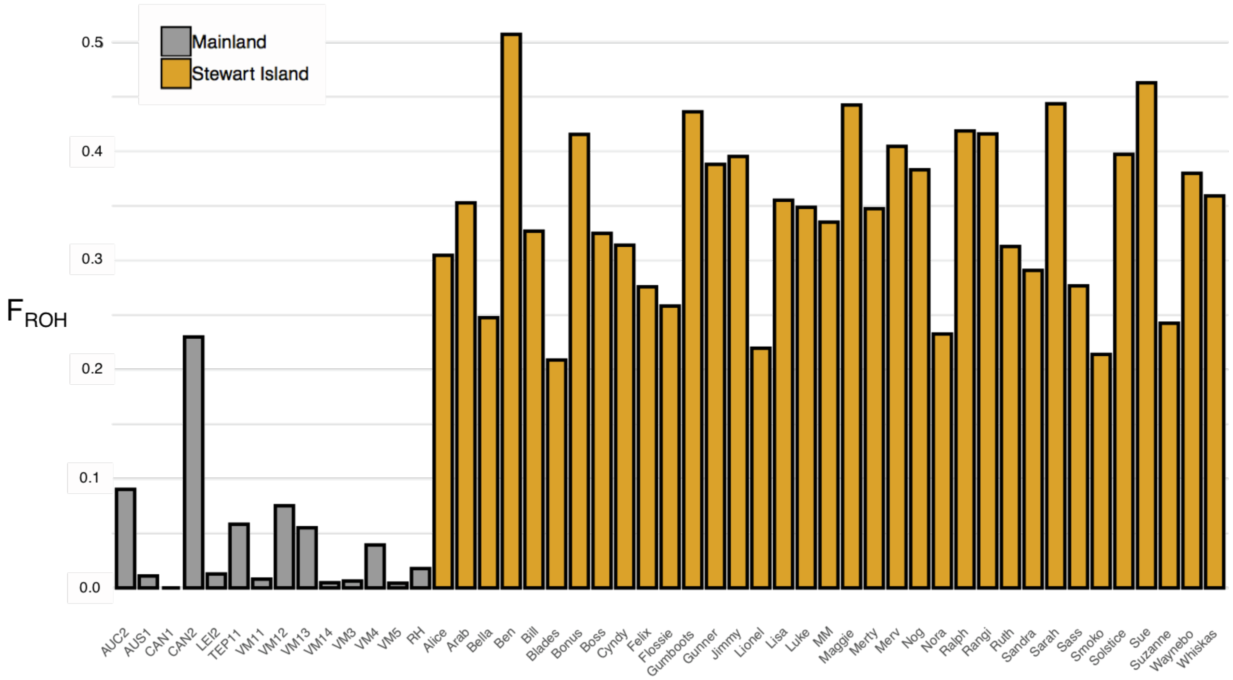

Figure S12. Proportion of the genome in Runs of Homozygosity (F_{ROH}), Related to Figure 2. F_{ROH} was calculated by including only ROH ≥2Mb in 14 mainland and 35 Stewart Island kākāpō using the sliding window approach implemented in PLINK. The average F_{ROH} for ROH ≥100kb was 15.1% ±6.5 SD, and 52.9% ±6.6 SD (P<0.01), for the mainland and Stewart Island, respectively. The average F_{ROH} for ROH ≥2Mb was 4.4% ±6.1 SD and 34.3% ±7.7 SD (P<0.01) for the mainland and Stewart Island, respectively.

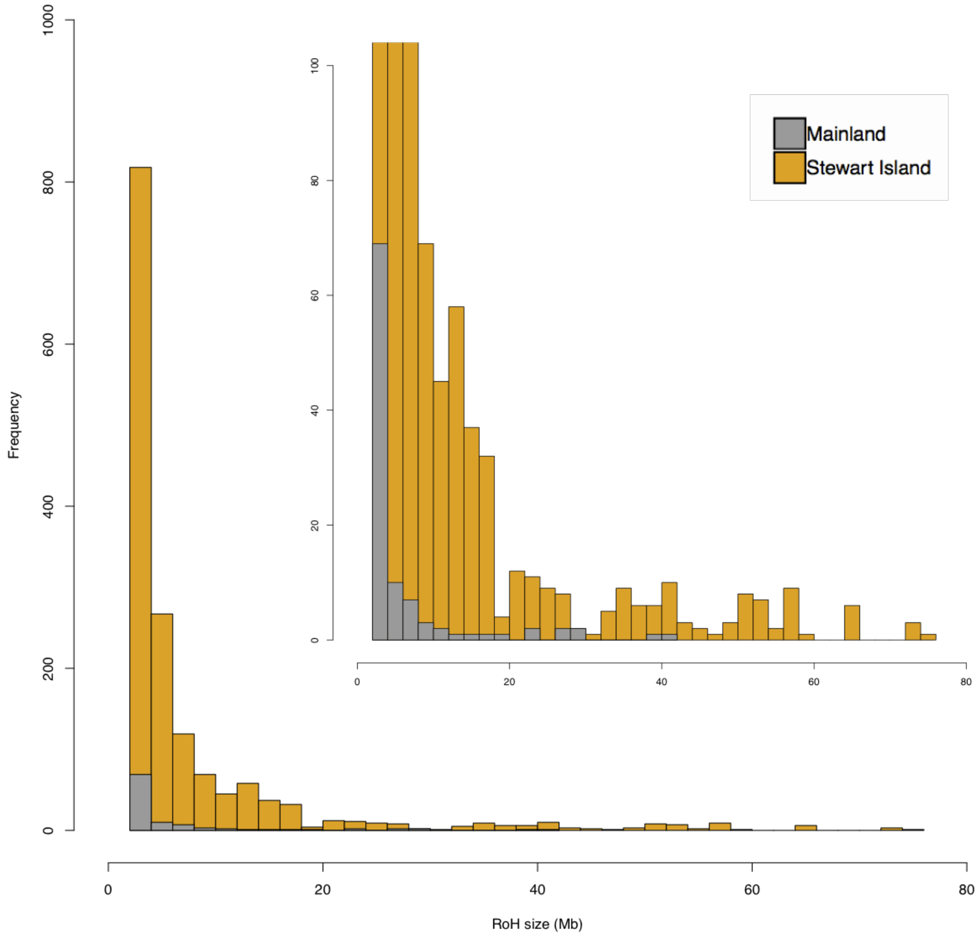

Figure S13. Frequency distribution of runs of homozygosity (ROH) size, Related to Figure 2. Only ROH ≥2Mb are shown. Inset shows the magnification for clarity. We identified a total of 22,458 ROH and the longest ROH sizes for the mainland and Stewart Island were 41.34Mb and of 75.61Mb, respectively.

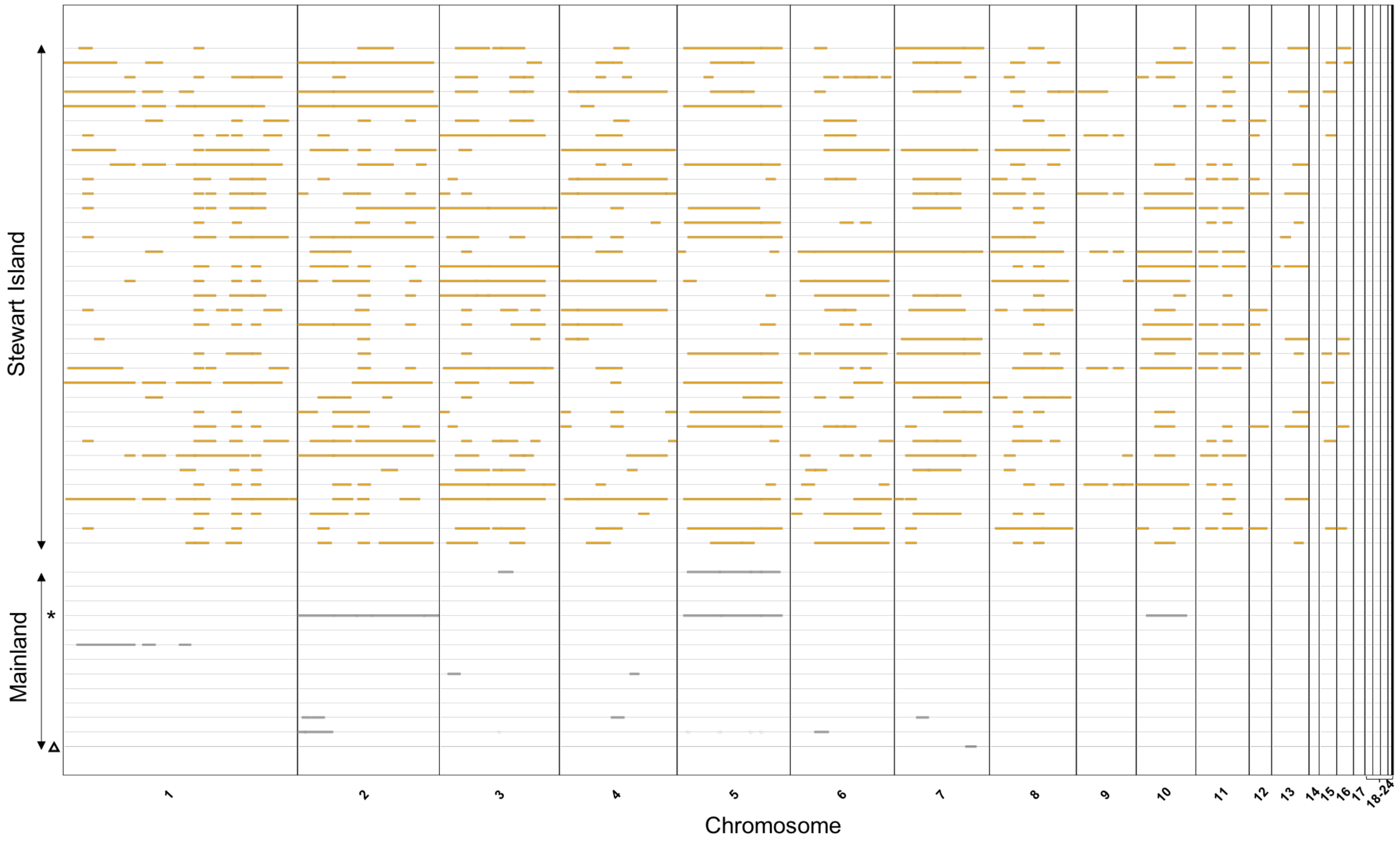

Figure S14. Distribution of ROH ≥5Mb along the 24 autosomes for 35 Stewart Island (top panel, yellow) and 14 mainland (bottom panel, grey) kākāpō, Related to Figure 2. We used the sliding window approach implemented in PLINK. The asterisk and triangle depict the mainland historical sample with the highest F_{ROH} (CAN2) and Richard Henry, respectively. Chromosomes are numbered and separated by vertical lines. In some cases, ROH span nearly entire chromosomes.

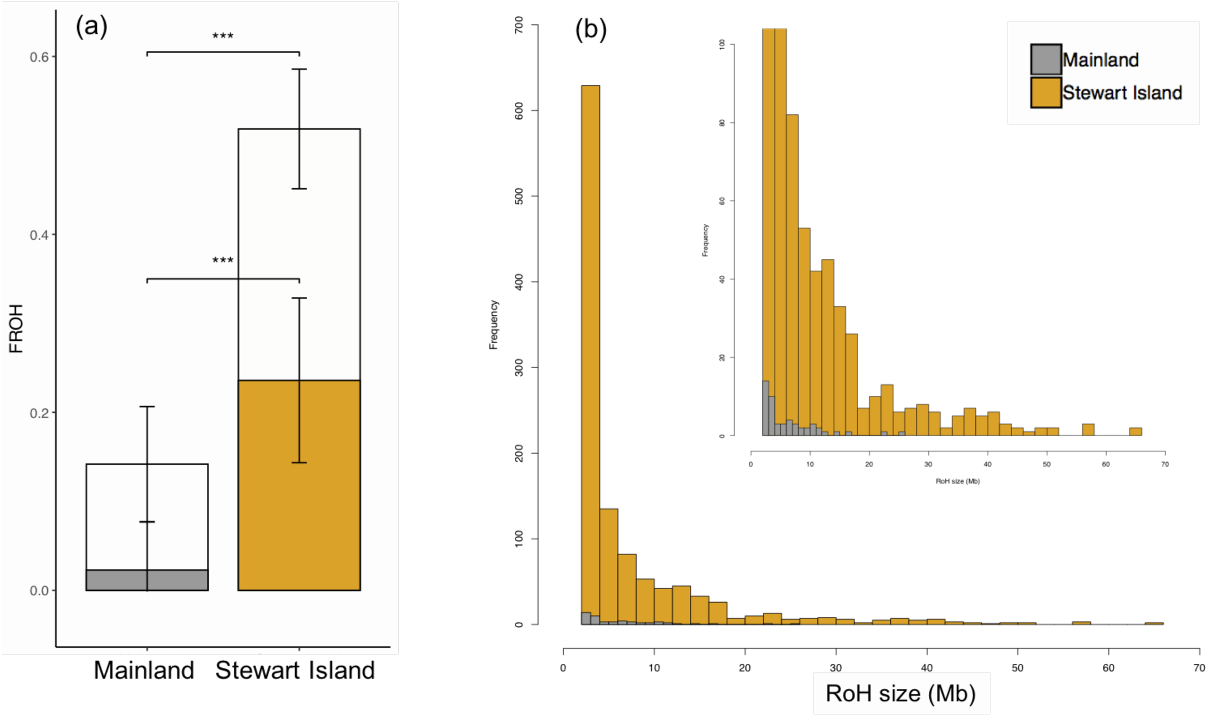

Figure S15. Average proportion of the genome in Runs of Homozygosity (F_{ROH}) and distribution of runs of homozygosity (ROH) in mainland and Stewart Island kākāpō, Related to STAR Methods. Results were obtained using more stringent parameters to assess the robustness of ROH estimates. **(a)** Open bars show total proportion of the genome in ROH ≥100kb and solid bars show proportions in ROH of length ≥2Mb. Bars extending from the mean values represent the standard deviation (Welch's two-sample t-test; ***P<0.001). The average F_{ROH} for ROH ≥100kb was 14.2% ±6.5 SD, and 51.8% ±6.7 SD (P<0.01), for the mainland and Stewart Island, respectively. The average F_{ROH} for ROH ≥2Mb was 2.3% ±5.4 SD and 23.6% ±9.3 SD (P<0.01) for the mainland and Stewart Island, respectively. **(b)** Frequency distribution of ROH size for ROH ≥1Mb. Results are shown here for comparison with main results and were obtained using more stringent parameters such as: *homozyg-het*=1; *homozyg-density* 100; *homozyg-gap* 500. Varying the number of heterozygous sites per ROH and allowing a maximum value of 1 (*homozyg-het 1*) had the most effect on ROH detection and resulted in large ROH being broken into ROH of smaller size, thereby increasing the overall number of ROH to 33,857. The longest ROH sizes for the mainland and Stewart Island were 25.19Mb and of 65.79Mb, respectively.

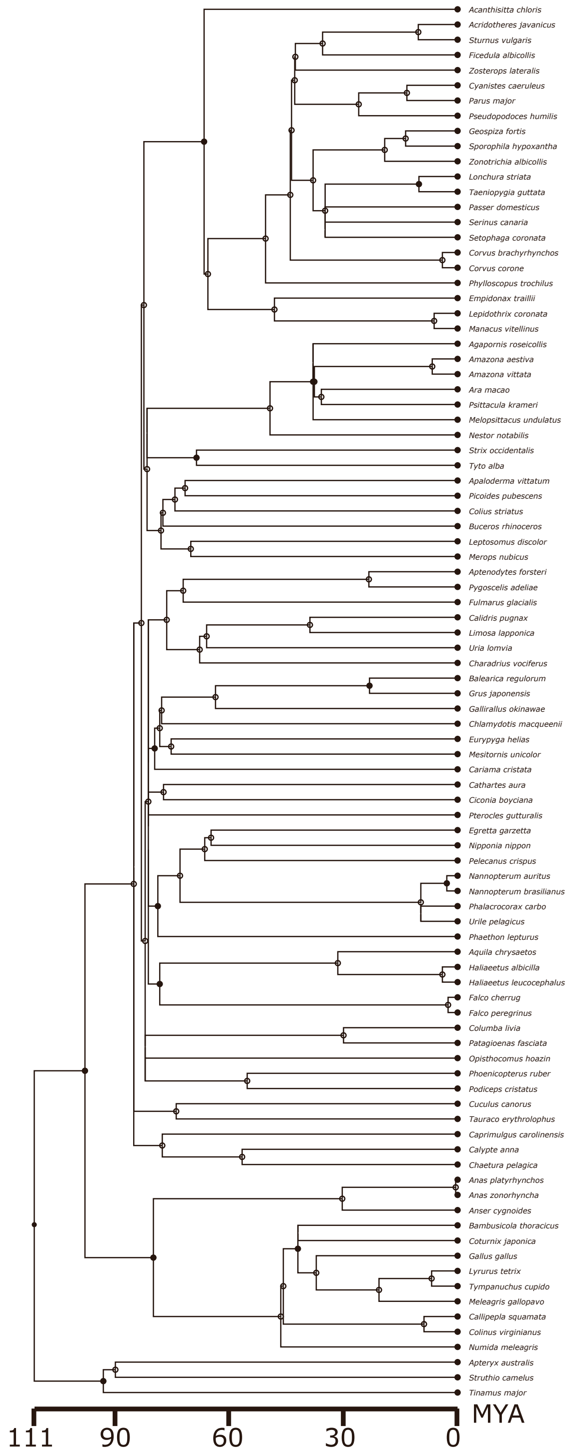

Figure S16. Bird genomes (n=135) used to calculate GERP-scores, Related to STAR Methods. Divergence time estimates were obtained from[59].

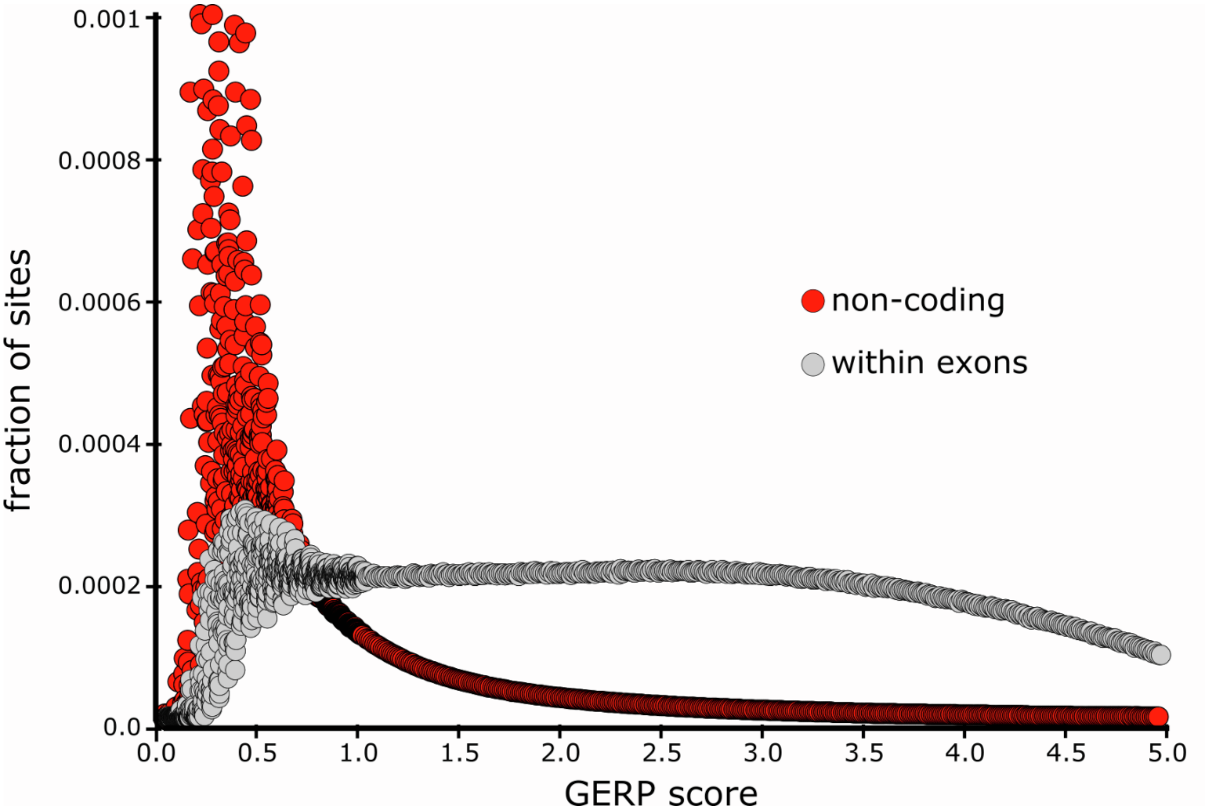

Figure S17. **Distribution of GERP-scores subdivided by non-coding regions (red) and within exons (grey), Related to STAR Methods.** The peak for low GERP scores (<1) is caused by stochastic effects as only few genomes align in these fast-evolving regions. The much higher GERP-scores within exons relative to outside of exons indicates that the method accurately estimates genome conservation[60]. The overlap indicates that not all exonic regions are highly conserved whereas some putatively non-coding regions are.

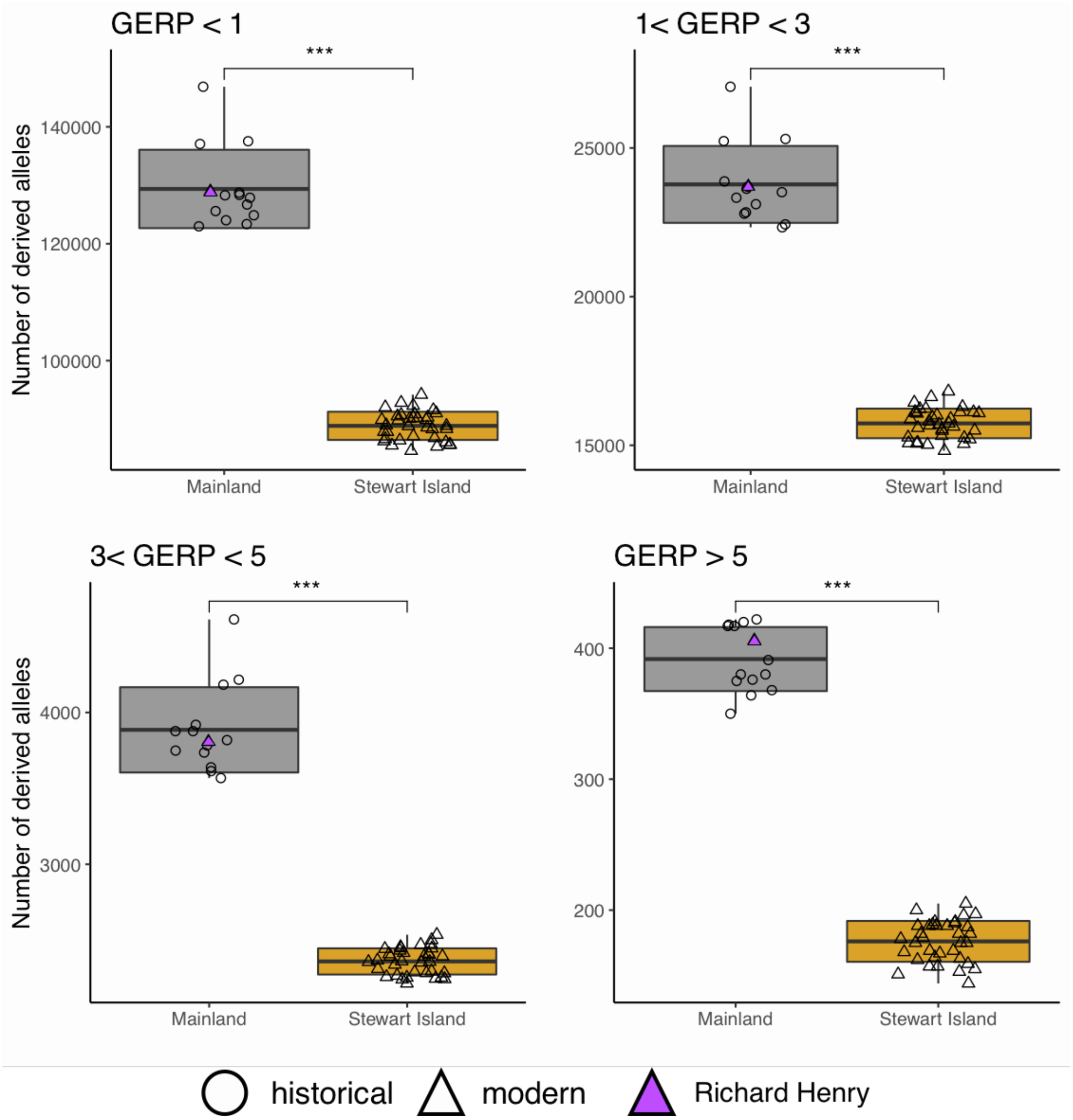

Figure S18. **Number of derived alleles per individual stratified by GERP-score, Related to Figure 3 and STAR Methods.** Middle line within boxplots bounds of boxes represent mean and standard deviation, respectively (Welch's two-sample t-test; ***P<0.001). Stewart Island kākāpō have consistently less derived alleles for all GERP-scores categories. At high GERP-scores, Stewart Island kākāpō have relatively much less derived alleles compared to the mainland population, consistent with a scenario where the most deleterious alleles are purged more efficiently than those at low GERP-scores.

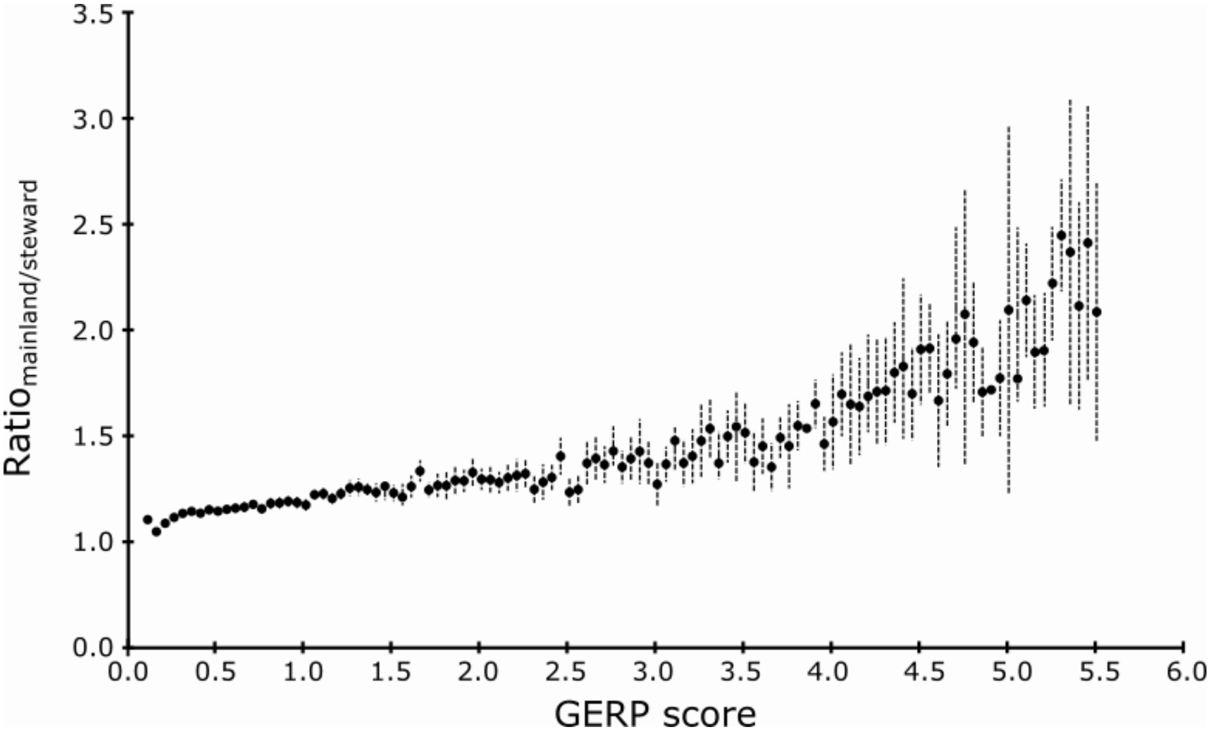

Figure S19. **The ratio of derived alleles between mainland and Stewart Island stratified by GERP-score, Related to Figure 3 and STAR Methods.** Each point represents the ratio of derived alleles between mainland and steward island by GERP (binned in intervals of 0.1) score category and striped lines show the 95% confidence interval. The difference in the number of deleterious alleles is most pronounced at sites under the strongest evolutionary constraint (i.e., GERP>2), with relatively more highly deleterious alleles in the mainland population compared to Stewart Island.

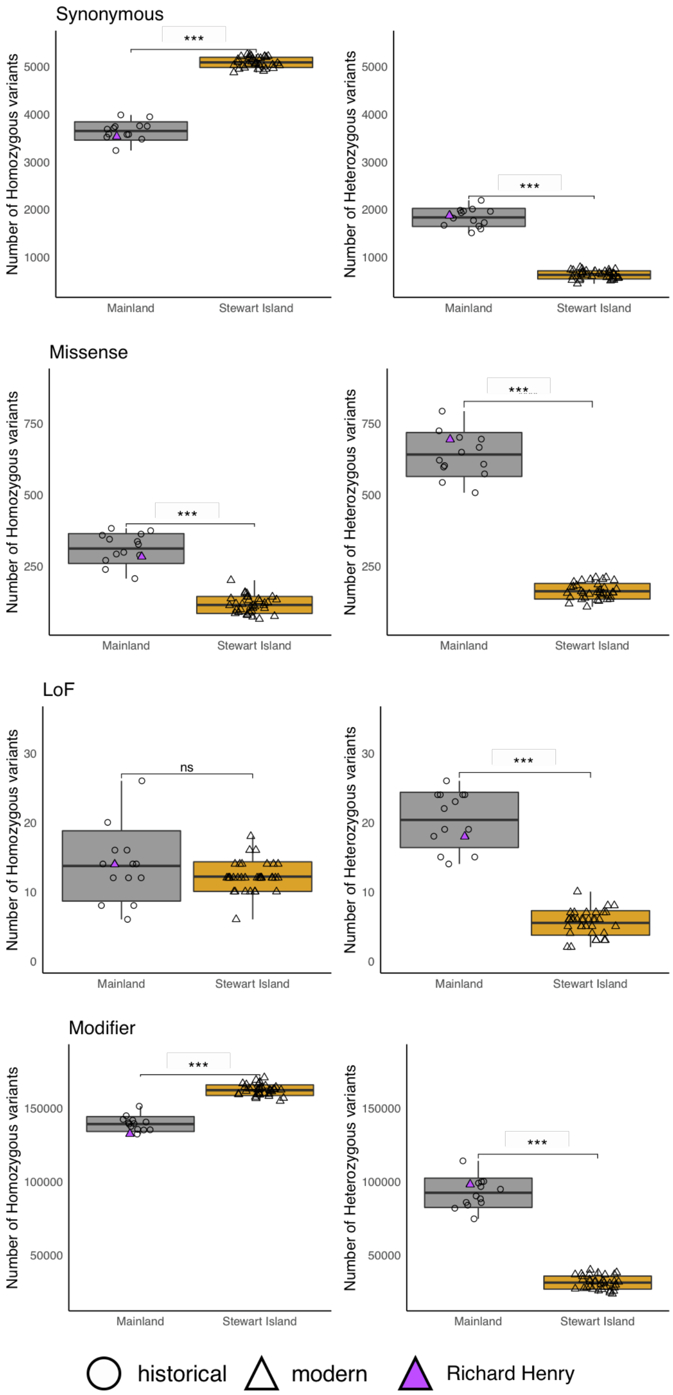

Figure S20. **Number of Synonymous, Missense, LoF and Modifier impact (i.e., downstream or upstream) variants in mainland and Stewart Island kākāpō based on the modern assembly (i.e., Jane), Related to Figure 3 and STAR Methods.** Variants are separated by homozygous or heterozygous state. Low = synonymous; Moderate = missense; High = Loss of function (LoF); Modifier = downstream or upstream. Middle thick lines within boxplots and bounds of boxes represent mean and standard deviation, respectively (Welch's two-sample t-test; ***$P<0.001$). The Stewart Island birds carried on average 17.6

LoF variants per bird while the mainland birds carried on average 34.1 LoF variants, with Richard Henry having 32 LoF variants. In contrast to the Stewart Island population, the mainland population had a higher number of LoF alleles in heterozygous state than the Stewart Island population, which is consistent with deleterious in heterozygous state being less to exposed to selection[61].

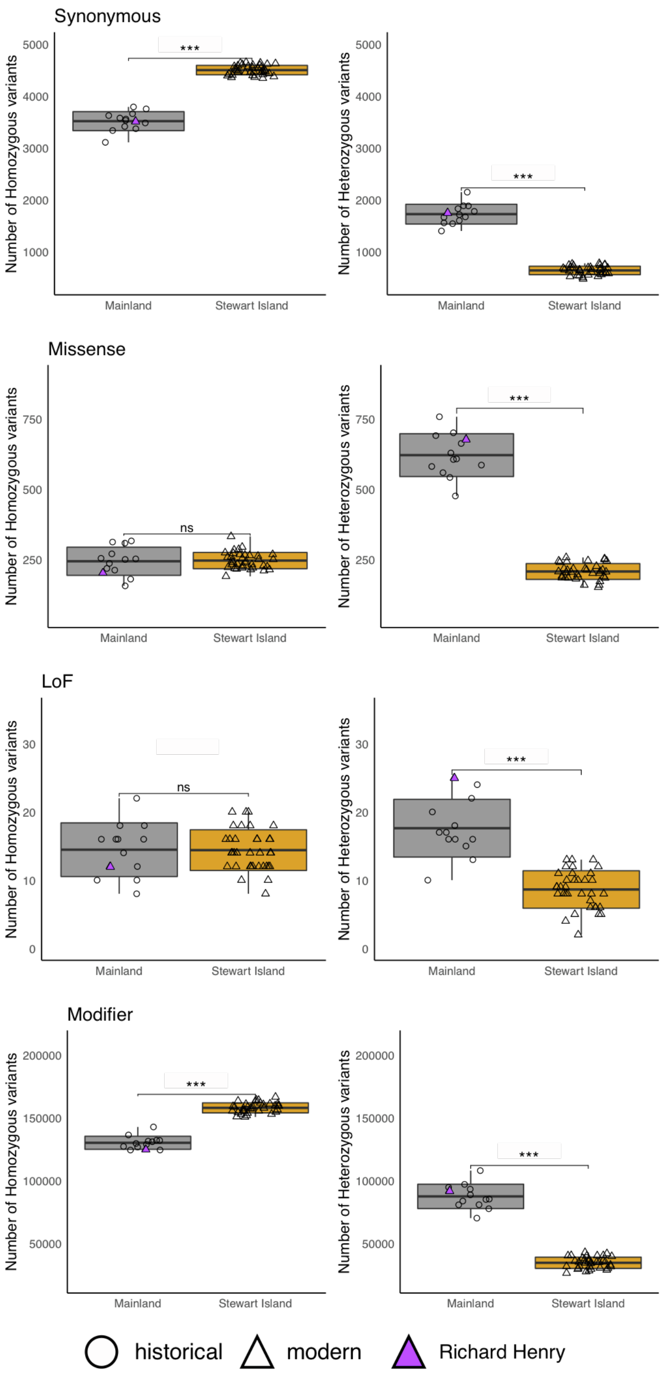

Figure S21. Number of Synonymous, Missense, LoF and Modifier impact (i.e., downstream or upstream) variants in mainland and Stewart Island kākāpō based on an historical consensus assembly (LEI2), Related to Figure 3 and STAR Methods. Variants are separated by homozygous or heterozygous state. Low = synonymous; Moderate = missense; High = Loss of function (LoF); Modifier = downstream or upstream. Middle thick lines within boxplots and bounds of boxes represent mean and standard deviation, respectively (Welch's two-sample t-test; ***P<0.001). The Stewart Island birds carried on average 23 LoF variants per bird while the mainland birds carried on average 31 LoF variants, with Richard Henry having 37 LoF variants.

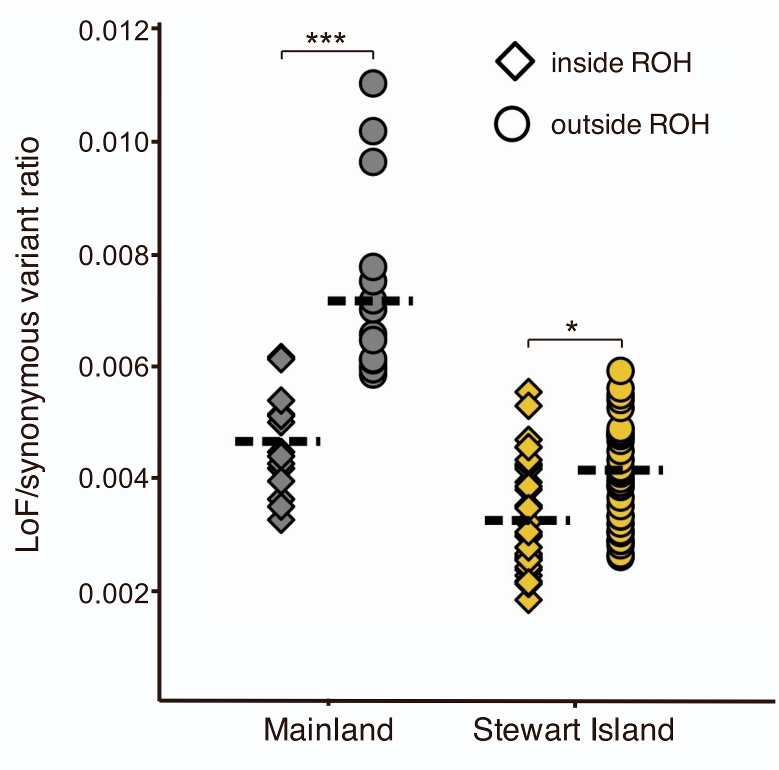

Figure S22. Rates of LoF variants in runs of homozygosity (ROH) for kākāpō, Related to Figure 3 and STAR Methods. Diamonds and circles show the rate of LoF variants relative to synonymous variants inside and outside ROH, respectively, for each individual genome. Middle dashed lines represent means (Welch's two-sample t-test; *P<0.05; ***P<0.001). There was a significantly lower number of LoF alleles inside ROH compared to heterozygous parts of individual genomes in both populations. However, this difference was almost 3-fold smaller in the Stewart Island population compared to the mainland population, suggesting that repeated inbreeding events may have facilitated the removal of a significant proportion of severely deleterious and recessive LoF alleles through exposure in homozygous state from the Stewart island population.

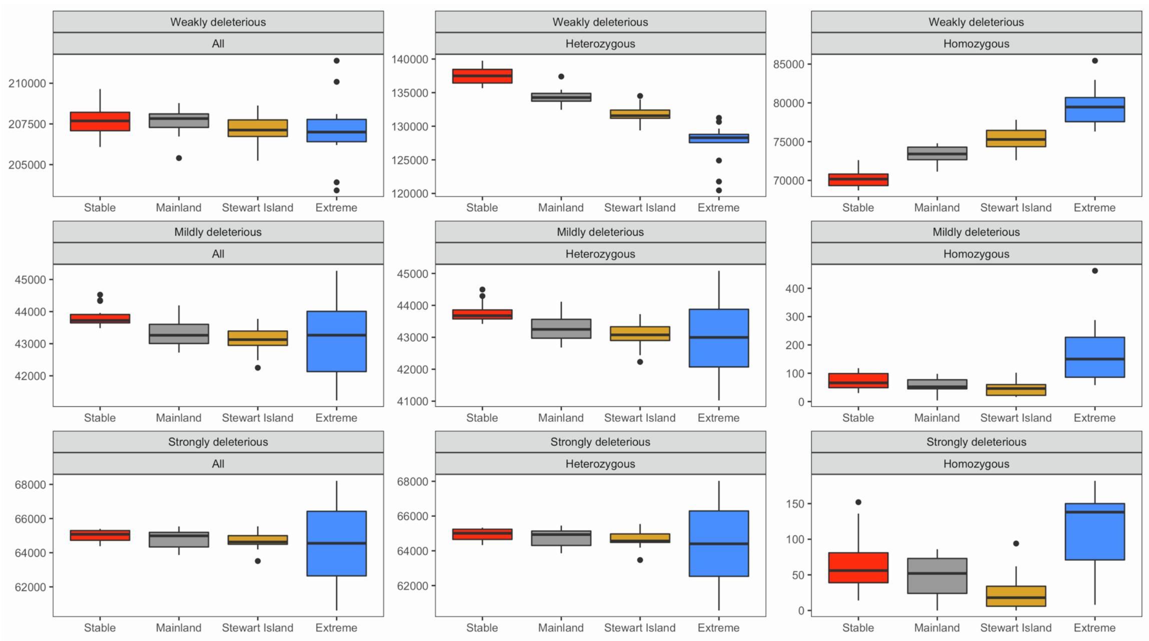

Figure S23. **Counts of derived alleles in the forward simulations with fully recessive dominance coefficients, Related to Figure 4 and STAR Methods.** Allelic counts of all, heterozygous and homozygous for weakly deleterious (-0.001≤ s < 0), mildly deleterious (-0.01 ≤ s < -0.001) and strongly deleterious (s < -0.01) mutations.

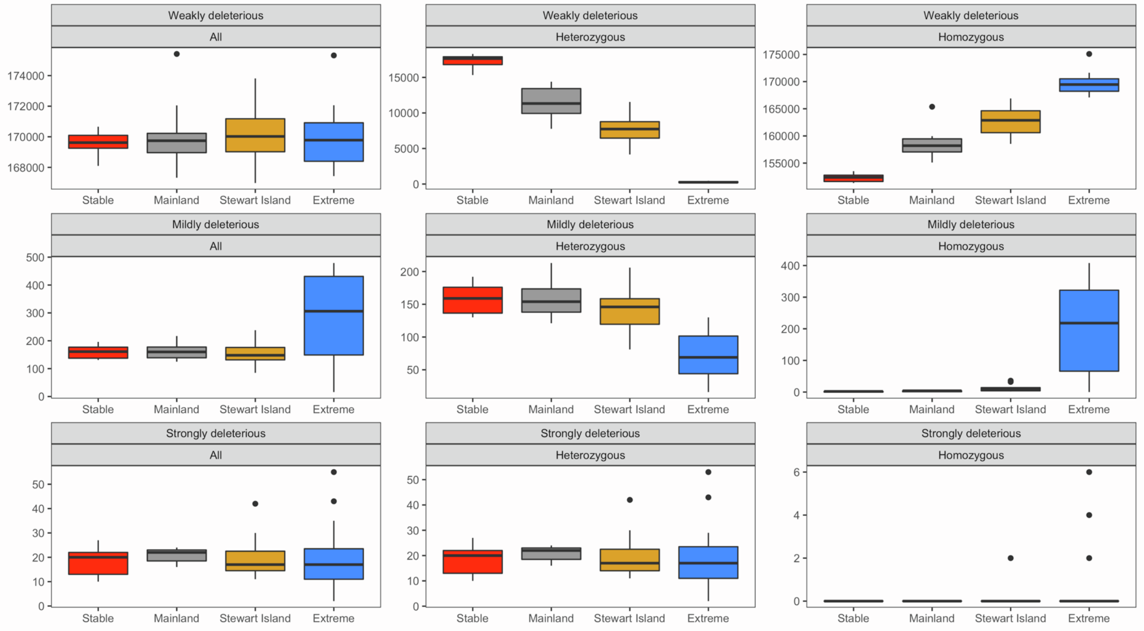

Figure S24. **Counts of derived alleles in the forward simulations with partially recessive dominance coefficients, Related to Figure 4 and STAR Methods.** Allelic counts of all, heterozygous and homozygous for weakly deleterious (-0.001≤ s < 0), mildly deleterious (-0.01 ≤ s < -0.001) and strongly deleterious (s < -0.01) mutations.

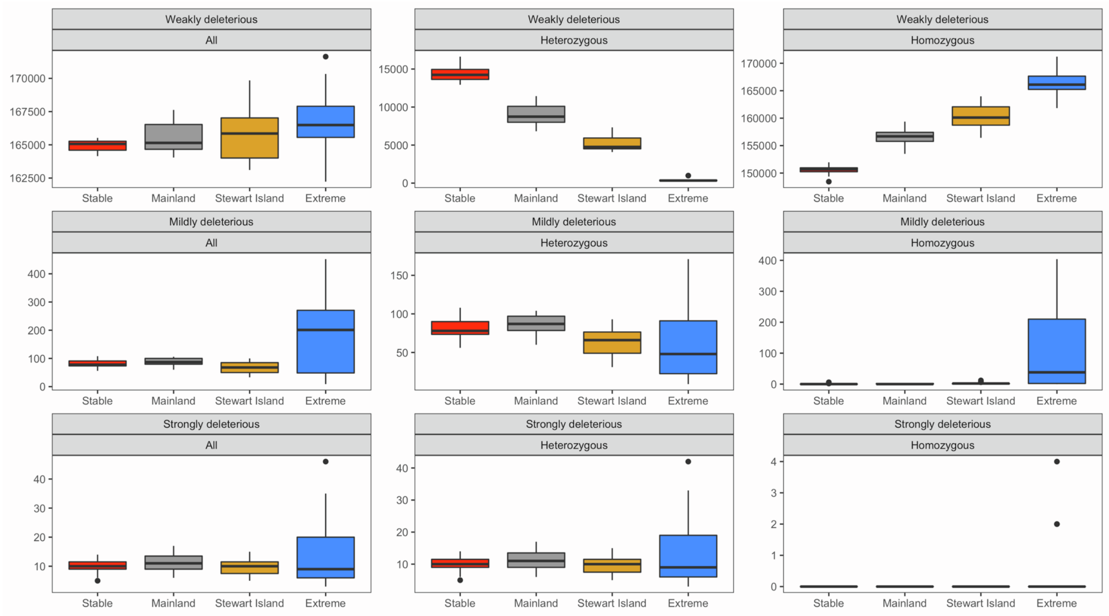

Figure S25. Counts of derived alleles in the forward simulations with additive dominance coefficients, Related to Figure 4 and STAR Methods. Allelic counts of all, heterozygous and homozygous for weakly deleterious (-0.001≤ s < 0), mildly deleterious (-0.01 ≤ s < -0.001) and strongly deleterious (s < -0.01) mutations.

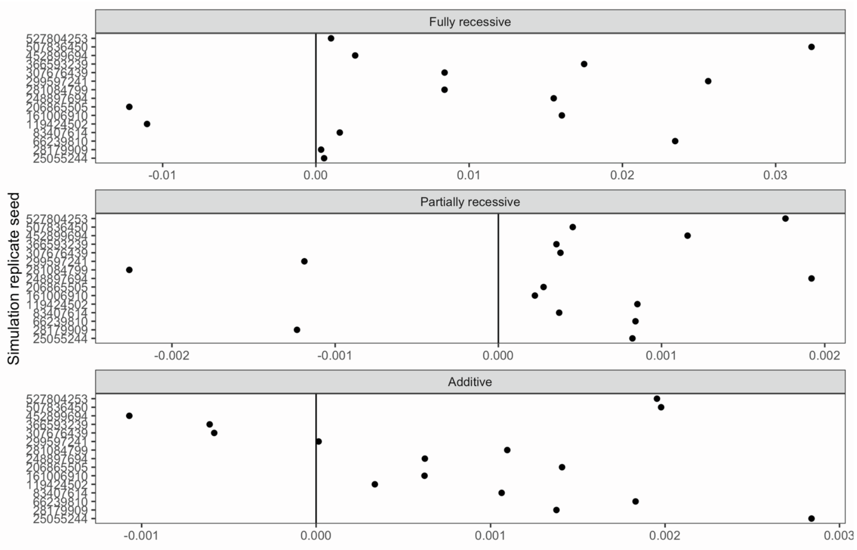

Figure S26. **Additive genetic load difference between simulations under the Stewart Island and the mainland scenarios per replicate, Related to Figure 4 and STAR Methods.** Values above zero indicate replicates where genetic load was lower in the Stewart Island population compared to the mainland population. Not all simulations replicates showed the same pattern, suggesting that this pattern is unlikely to be universal due to the randomness nature of evolutionary dynamics and historical contingencies. Moreover, variable dominance coefficients act simultaneously and partially recessive mutations are expected to disproportionately contribute to inbreeding depression and thus have an impact on purging effects[62]. A wider exploration of different demographic (e.g., size and duration of the founder and bottleneck events), genetic (e.g., range of selection and dominance effects) and life-history (e.g., levels of inbreeding and mating-types) comparisons with simulations and empirical data is needed to investigate the balance between purging and drift-load during evolutionary history (see[63,64]).

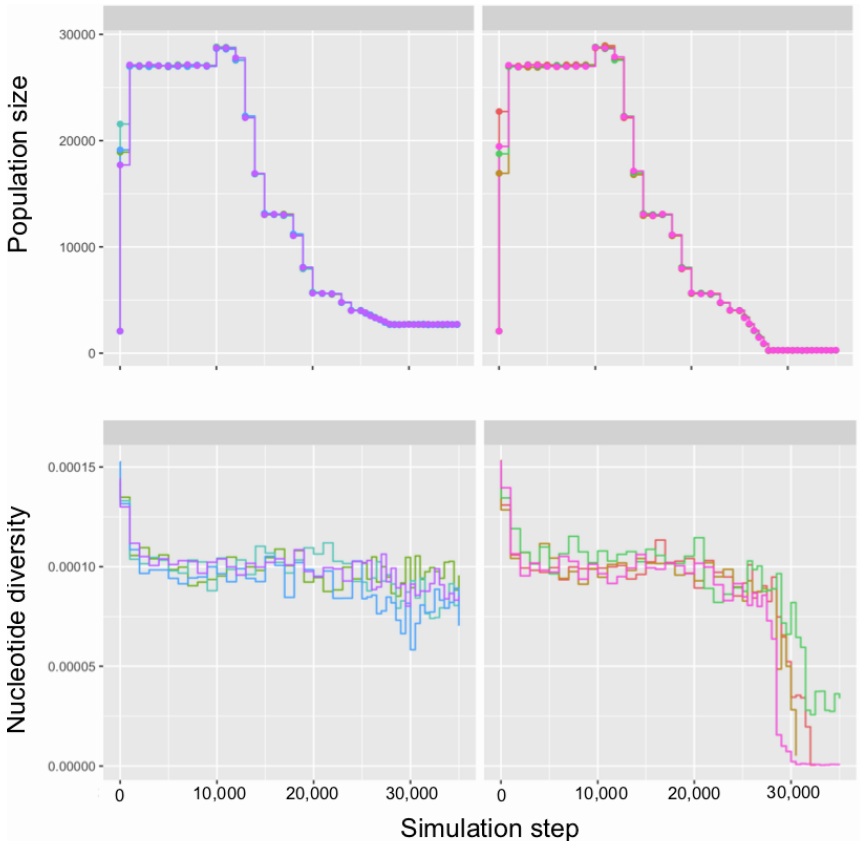

Figure S27. **Effect of scaling during forward simulations, Related to Figure 4 and STAR Methods.** Simulations were scaled to speed-up burn-in period as recommended in the SLiM manual. After burn-in the scaling factor was removed. Here, we show how nucleotide diversity is not impacted by removing the scaling factor (i.e., the mutation-selection equilibrium is maintained) during a period of 10,000 steps (steps 0-10,000) immediately before when we started to vary the population size according to demographic scenarios (steps 10,000-35,000 = 25,000 steps/years).

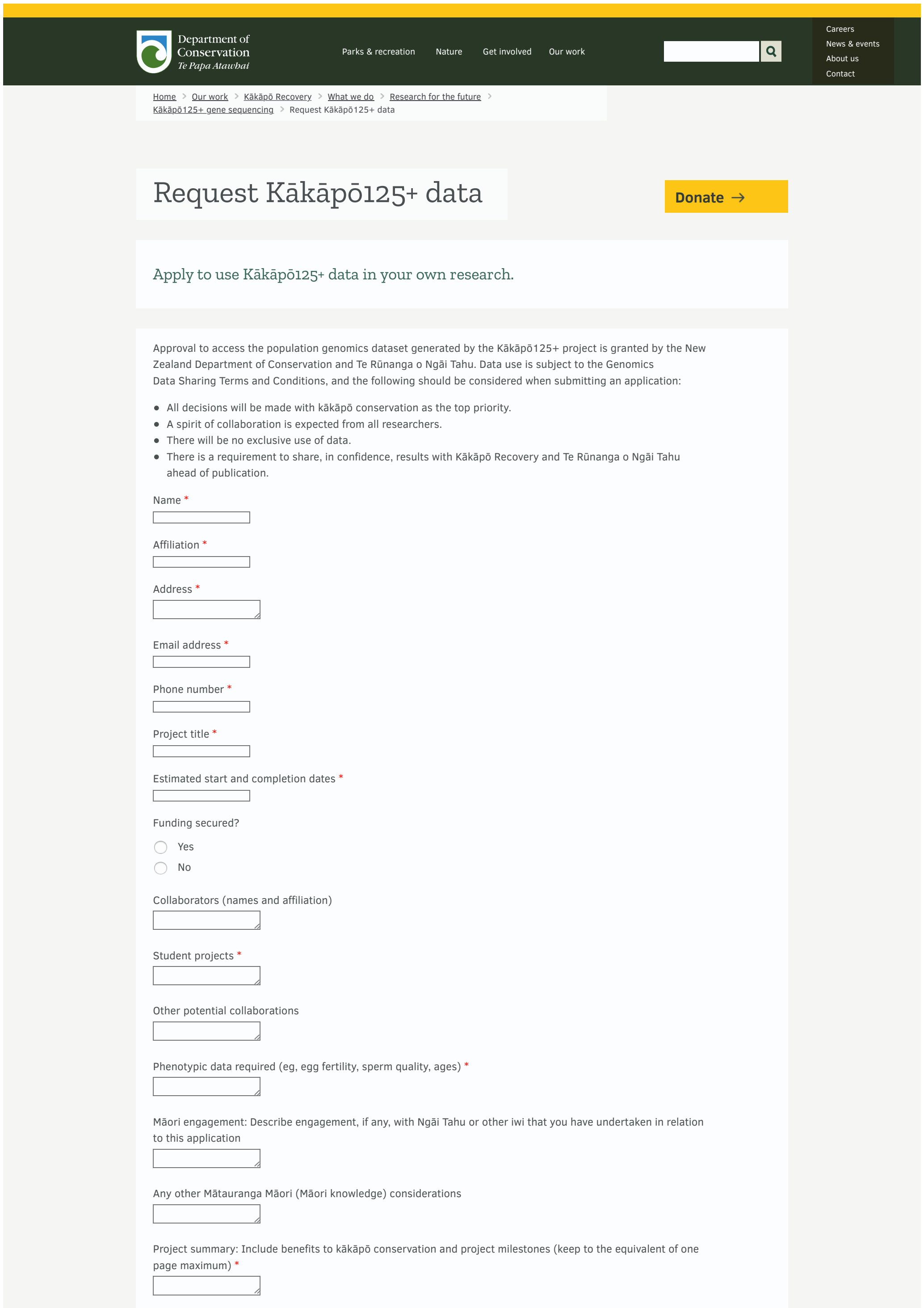

Department of Conservation
Te Papa Atawhai

Parks & recreation | Nature | Get involved | Our work

Careers
News & events
About us
Contact

Home > Our work > Kākāpō Recovery > What we do > Research for the future > Kākāpō125+ gene sequencing > Request Kākāpō125+ data

Request Kākāpō125+ data

Donate →

Apply to use Kākāpō125+ data in your own research.

Approval to access the population genomics dataset generated by the Kākāpō125+ project is granted by the New Zealand Department of Conservation and Te Rūnanga o Ngāi Tahu. Data use is subject to the Genomics Data Sharing Terms and Conditions, and the following should be considered when submitting an application:

- All decisions will be made with kākāpō conservation as the top priority.
- A spirit of collaboration is expected from all researchers.
- There will be no exclusive use of data.
- There is a requirement to share, in confidence, results with Kākāpō Recovery and Te Rūnanga o Ngāi Tahu ahead of publication.

Name *

Affiliation *

Address *

Email address *

Phone number *

Project title *

Estimated start and completion dates *

Funding secured?

Yes

No

Collaborators (names and affiliation)

Student projects *

Other potential collaborations

Phenotypic data required (eg, egg fertility, sperm quality, ages) *

Māori engagement: Describe engagement, if any, with Ngāi Tahu or other iwi that you have undertaken in relation to this application

Any other Mātauranga Māori (Māori knowledge) considerations

Project summary: Include benefits to kākāpō conservation and project milestones (keep to the equivalent of one page maximum) *

Figure S28. Request form for kākāpō125+ modern genomic data, Related to STAR Methods. The online form can be found at: https://www.doc.govt.nz/our-work/kakapo-recovery/what-we-do/research-for-the-future/kakapo125-gene-sequencing/request-kakapo125-data/.

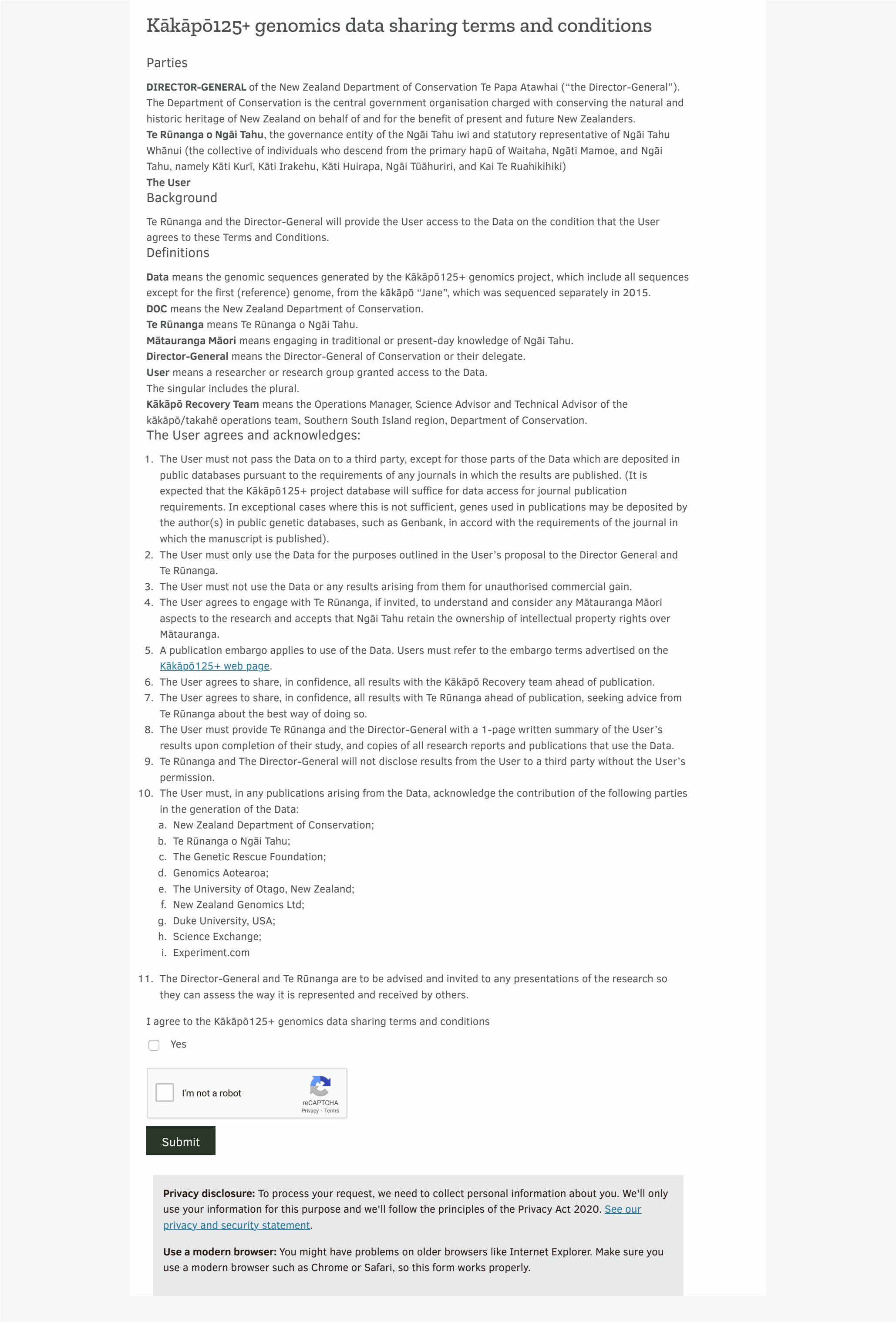

Kākāpō125+ genomics data sharing terms and conditions

Parties

DIRECTOR-GENERAL of the New Zealand Department of Conservation Te Papa Atawhai (“the Director-General”). The Department of Conservation is the central government organisation charged with conserving the natural and historic heritage of New Zealand on behalf of and for the benefit of present and future New Zealanders.
Te Rūnanga o Ngāi Tahu, the governance entity of the Ngāi Tahu iwi and statutory representative of Ngāi Tahu Whānui (the collective of individuals who descend from the primary hapū of Waitaha, Ngāti Mamoe, and Ngāi Tahu, namely Kāti Kurī, Kāti Irakehu, Kāti Huirapa, Ngāi Tūāhuriri, and Kai Te Ruahikihiki)
The User

Background

Te Rūnanga and the Director-General will provide the User access to the Data on the condition that the User agrees to these Terms and Conditions.

Definitions

Data means the genomic sequences generated by the Kākāpō125+ genomics project, which include all sequences except for the first (reference) genome, from the kākāpō “Jane”, which was sequenced separately in 2015.
DOC means the New Zealand Department of Conservation.
Te Rūnanga means Te Rūnanga o Ngāi Tahu.
Mātauranga Māori means engaging in traditional or present-day knowledge of Ngāi Tahu.
Director-General means the Director-General of Conservation or their delegate.
User means a researcher or research group granted access to the Data.
The singular includes the plural.
Kākāpō Recovery Team means the Operations Manager, Science Advisor and Technical Advisor of the kākāpō/takahē operations team, Southern South Island region, Department of Conservation.

The User agrees and acknowledges:

1. The User must not pass the Data on to a third party, except for those parts of the Data which are deposited in public databases pursuant to the requirements of any journals in which the results are published. (It is expected that the Kākāpō125+ project database will suffice for data access for journal publication requirements. In exceptional cases where this is not sufficient, genes used in publications may be deposited by the author(s) in public genetic databases, such as Genbank, in accord with the requirements of the journal in which the manuscript is published).
2. The User must only use the Data for the purposes outlined in the User’s proposal to the Director General and Te Rūnanga.
3. The User must not use the Data or any results arising from them for unauthorised commercial gain.
4. The User agrees to engage with Te Rūnanga, if invited, to understand and consider any Mātauranga Māori aspects to the research and accepts that Ngāi Tahu retain the ownership of intellectual property rights over Mātauranga.
5. A publication embargo applies to use of the Data. Users must refer to the embargo terms advertised on the Kākāpō125+ web page.
6. The User agrees to share, in confidence, all results with the Kākāpō Recovery team ahead of publication.
7. The User agrees to share, in confidence, all results with Te Rūnanga ahead of publication, seeking advice from Te Rūnanga about the best way of doing so.
8. The User must provide Te Rūnanga and the Director-General with a 1-page written summary of the User’s results upon completion of their study, and copies of all research reports and publications that use the Data.
9. Te Rūnanga and The Director-General will not disclose results from the User to a third party without the User’s permission.
10. The User must, in any publications arising from the Data, acknowledge the contribution of the following parties in the generation of the Data:
    a. New Zealand Department of Conservation;
    b. Te Rūnanga o Ngāi Tahu;
    c. The Genetic Rescue Foundation;
    d. Genomics Aotearoa;
    e. The University of Otago, New Zealand;
    f. New Zealand Genomics Ltd;
    g. Duke University, USA;
    h. Science Exchange;
    i. Experiment.com
11. The Director-General and Te Rūnanga are to be advised and invited to any presentations of the research so they can assess the way it is represented and received by others.

I agree to the Kākāpō125+ genomics data sharing terms and conditions

☐ Yes

I'm not a robot reCAPTCHA Privacy - Terms

Submit

Privacy disclosure: To process your request, we need to collect personal information about you. We'll only use your information for this purpose and we'll follow the principles of the Privacy Act 2020. See our privacy and security statement.

Use a modern browser: You might have problems on older browsers like Internet Explorer. Make sure you use a modern browser such as Chrome or Safari, so this form works properly.

Figure S28. **(cont.) Request form for kākāpō125+ modern genomic data, Related to STAR Methods.** The online form can be found at: https://www.doc.govt.nz/our-work/kakapo-recovery/what-we-do/research-for-the-future/kakapo125-gene-sequencing/request-kakapo125-data/.

References

1. Sambrook, J., Fritsch, E.F., and Maniatis, T. (1989). Molecular cloning: A laboratory manual, 2nd edn (Cold Spring Harbor Laboratory Press).
2. Dussex, N., von Seth, J., Robertson, B.C., and Dalén, L. (2018). Full mitogenomes in the critically endangered kākāpō reveal major post-glacial and anthropogenic effects on neutral genetic diversity. Genes. *9*, 1–14.
3. Knapp, M., Clarke, A.C., Horsburgh, K.A., and Matisoo-Smith, E.A. (2012). Setting the stage - Building and working in an ancient DNA laboratory. Ann. Anat. *194*, 3–6.
4. Rhie, A., McCarthy, S.A., Fedrigo, O., Damas, J., Formenti, G., Koren, S., Uliano-Silva, M., Chow, W., Fungtammasan, A., Gedman, G.L., et al. (2021). Towards complete and error-free genome assemblies of all vertebrate species. Nature *592*, 737–746.
5. Chin, C.S., Peluso, P., Sedlazeck, F.J., Nattestad, M., Concepcion, G.T., Clum, A., Dunn, C., O'Malley, R., Figueroa-Balderas, R., Morales-Cruz, A., et al. (2016). Phased diploid genome assembly with single-molecule real-time sequencing. Nat. Methods *13*, 1050–1054.
6. Lam, H.Y.K., Ball, M.P., Nielsen, C.B., Pan, C., Thakuria, J. V, Younesy, H., Clark, M.J., Zaranek, A.W., O'Geen, H., Lacroute, P., et al. (2012). Detecting and annotating genetic variations using the HugeSeq pipeline. Nat. Biotechnol. *30*, 226–229.
7. Ghurye, J., Rhie, A., Walenz, B.P., Schmitt, A., Selvaraj, S., Pop, M., Phillippy, A.M., and Koren, S. (2019). Integrating Hi-C links with assembly graphs for chromosome-scale assembly. PLoS Comput. Biol. *15*, 1–19.
8. Bishara, A., Liu, Y., Weng, Z., Kashef-Haghighi, D., Newburger, D.E., West, R., Sidow, A., and Batzoglou, S. (2015). Read clouds uncover variation in complex regions of the human genome. Genome Res. *25*, 1570–1580.
9. Garrison, E., and Marth, G. (2012). Haplotype-based variant detection from short-read sequencing. arXiv, 1207.3907 [q-bio].
10. Li, H. (2011). A statistical framework for SNP calling, mutation discovery, association mapping and population genetical parameter estimation from sequencing data. Bioinformatics *27*, 2987–2993.
11. Roach, M.J., Schmidt, S.A., and Borneman, A.R. (2018). Purge Haplotigs: Allelic contig reassignment for third-gen diploid genome assemblies. BMC Bioinformatics *19*, 1–10.

12. Chow, W., Brugger, K., Caccamo, M., Sealy, I., Torrance, J., and Howe, K. (2016). GEVAL - A web-based browser for evaluating genome assemblies. Bioinformatics *32*, 2508–2510.
13. Durand, N.C., Robinson, J.T., Shamim, M.S., Machol, I., Mesirov, J.P., Lander, E.S., and Aiden, E.L. (2016). Juicebox Provides a Visualization System for Hi-C Contact Maps with Unlimited Zoom. Cell Syst. *3*, 99–101.
14. Camacho, C., Coulouris, G., Avagyan, V., Ma, N., Papadopoulos, J., Bealer, K., and Madden, T.L. (2009). BLAST + : architecture and applications. BMC Bioinformatics *10*, 421.
15. Okonechnikov, K., Conesa, A., and García-Alcalde, F. (2015). Qualimap 2: Advanced multi-sample quality control for high-throughput sequencing data. Bioinformatics *32*, 292–294.
16. Smit, A.F.A., Hubley, R., and Green, P. (2010). RepeatMasker Open-3.0. 1996-2010. Inst. Syst. Biol.
17. Jain, C., Koren, S., Dilthey, A., Phillippy, A.M., and Aluru, S. (2018). A fast adaptive algorithm for computing whole-genome homology maps. Bioinformatics *34*, i748–i756.
18. Chaisson, M.J., and Tesler, G. (2012). Mapping single molecule sequencing reads using basic local alignment with successive refinement (BLASR): application and theory. BMC Bioinformatics *13*, 238.
19. Salzberg, S.L., and Langmead, B. (2012). Fast gapped-read alignment with Bowtie 2. Nat. Methods *9*, 357–359.
20. Neethiraj, R., Hornett, E.A., Hill, J.A., and Wheat, C.W. (2017). Investigating the genomic basis of discrete phenotypes using a Pool-Seq-only approach: New insights into the genetics underlying colour variation in diverse taxa. Mol. Ecol. *26*, 4990–5002.
21. Hoff, K.J., Lange, S., Lomsadze, A., Borodovsky, M., and Stanke, M. (2016). BRAKER1: Unsupervised RNA-Seq-based genome annotation with GeneMark-ET and AUGUSTUS. Bioinformatics *32*, 767–769.
22. Stanke, M., Diekhans, M., and Robert Baertsch, D.H. (2008). Using native and syntenically mapped cDNA alignments to improve de novo gene finding. Bioinformatics *24*, 637–644.
23. Stanke, M., Schöffmann, O., Morgenstern, B., and Waack, S. (2006). Gene prediction in

eukaryotes with a generalized hidden Markov model that uses hints from external sources. BMC Bioinformatics *7*.

24. Lomsadze, A., Ter-Hovhannisyan, V., Chernoff, Y.O., and Borodovsky, M. (2005). Gene identification in novel eukaryotic genomes by self-training algorithm. Nucleic Acids Res. *33*, 6494–6506.
25. Ter-Hovhannisyan, V., Lomsadze, A., Chernoff, Y.O., and Borodovsky, M. (2008). Gene prediction in novel fungal genomes using an ab initio algorithm with unsupervised training. Genome Res. *18*, 1979–1990.
26. Stanke, M., Keller, O., Gunduz, I., Hayes, A., Waack, S., and Morgenstern, B. (2006). AUGUSTUS: Ab initio prediction of alternative transcripts. Nucleic Acids Res. *34*, 435–439.
27. Camacho, C., Coulouris, G., Avagyan, V., Ma, N., Papadopoulos, J., Bealer, K., and Madden, T.L. (2009). BLAST+: Architecture and applications. BMC Bioinformatics *10*, 421.
28. Altschul, A.F., Gish, W., Miller, W., Myers, E.W. and Lipman, D.J. (1990). Basic Local Alignment Search Tool. Mol. Biol. *215*, 403–410.
29. Warren, W.C., Clayton, D.F., Ellegren, H., Arnold, A.P., Hillier, L.W., Künstner, A., Searle, S., White, S., Vilella, A.J., Fairley, S., et al. (2010). The genome of a songbird. Nature *464*, 757–762.
30. Ellegren, H., Smeds, L., Burri, R., Olason, P.I., Backström, N., Kawakami, T., Künstner, A., Mäkinen, H., Nadachowska-Brzyska, K., Qvarnström, A., et al. (2012). The genomic landscape of species divergence in Ficedula flycatchers. Nature *491*, 756–760.
31. Iwata, H., and Gotoh, O. (2012). Benchmarking spliced alignment programs including Spaln2, an extended version of Spaln that incorporates additional species-specific features. Nucleic Acids Res. *40*, 1–9.
32. Girgis, H.Z. (2015). Red: An intelligent, rapid, accurate tool for detecting repeats de-novo on the genomic scale. BMC Bioinformatics *16*.
33. Gremme, G., Brendel, V., Sparks, M.E., and Kurtz, S. (2005). Engineering a software tool for gene structure prediction in higher organisms. Inf. Softw. Technol. *47*, 965–978.
34. Trapnell, C., Roberts, A., Goff, L., Pertea, G., Kim, D., Kelley, D.R., Pimentel, H., Salzberg, S.L., Rinn, J.L., and Pachter, L. (2012). Differential gene and transcript

expression analysis of RNA-seq experiments with TopHat and Cufflinks. Nat. Protoc. *7*, 562–78.

35. Roberts, A., Trapnell, C., Donaghey, J., Rinn, J.L., and Pachter, L. (2011). Improving RNA-Seq expression estimates by correcting for fragment bias. Genome Biol. *12*.

36. Huerta-Cepas, J., Szklarczyk, D., Forslund, K., Cook, H., Heller, D., Walter, M.C., Rattei, T., Mende, D.R., Sunagawa, S., Kuhn, M., et al. (2016). EGGNOG 4.5: A hierarchical orthology framework with improved functional annotations for eukaryotic, prokaryotic and viral sequences. Nucleic Acids Res. *44*, D286–D293.

37. Aken, B.L., Ayling, S., Barrell, D., Clarke, L., Curwen, V., Fairley, S., Fernandez Banet, J., Billis, K., García Girón, C., Hourlier, T., et al. (2016). The Ensembl gene annotation system. Database (Oxford). *2016*.

38. The UniProt Consortium (2019). UniProt: A worldwide hub of protein knowledge. Nucleic Acids Res. *47*, D506–D515.

39. Li, H. (2018). Minimap2: Pairwise alignment for nucleotide sequences. Bioinformatics *34*, 3094–3100.

40. Li, H., and Durbin, R. (2010). Fast and accurate long-read alignment with Burrows-Wheeler transform. Bioinformatics *26*, 589–595.

41. Slater, G.S.C., and Birney, E. (2005). Automated generation of heuristics for biological sequence comparison. BMC Bioinformatics *6*.

42. She, R., Chu, J.S.C., Uyar, B., Wang, J., Wang, K., and Chen, N. (2011). genBlastG: Using BLAST searches to build homologous gene models. Bioinformatics *27*, 2141–2143.

43. Harris, R.S. (2007). Improved pairwise alignment of genomic DNA.

44. Sharma, V., Schwede, P., and Hiller, M. (2017). CESAR 2.0 substantially improves speed and accuracy of comparative gene annotation. Bioinformatics *33*, 3985–3987.

45. Kozomara, A., Birgaoanu, M., and Griffiths-Jones, S. (2019). MiRBase: From microRNA sequences to function. Nucleic Acids Res. *47*, D155–D162.

46. Kalvari, I., Argasinska, J., Quinones-Olvera, N., Nawrocki, E.P., Rivas, E., Eddy, S.R., Bateman, A., Finn, R.D., and Petrov, A.I. (2018). Rfam 13.0: Shifting to a genome-centric resource for non-coding RNA families. Nucleic Acids Res. *46*, D335–D342.

47. Pruitt, K.D., Brown, G.R., Hiatt, S.M., Thibaud-Nissen, F., Astashyn, A., Ermolaeva, O., Farrell, C.M., Hart, J., Landrum, M.J., McGarvey, K.M., et al. (2014). RefSeq: An update

on mammalian reference sequences. Nucleic Acids Res. *42*.

48. Morgulis, A., Gertz, E.M., Schäffer, A.A., and Agarwala, R. (2006). WindowMasker: Window-based masker for sequenced genomes. Bioinformatics *22*, 134–141.
49. Altschul, S.F., Gish, W.R., Miller, W., Myers, E.W., and Lipman, D.J. (1990). Basic local alignment search tool. J. Mol. Biol. *215*, 403–410.
50. Kapustin, Y., Souvorov, A., Tatusova, T., and Lipman, D. (2008). Splign: Algorithms for computing spliced alignments with identification of paralogs. Biol. Direct *3*.
51. Lowe, T.M., and Eddy, S.R. (1996). TRNAscan-SE: A program for improved detection of transfer RNA genes in genomic sequence. Nucleic Acids Res. *25*, 955–964.
52. Nawrocki, E.P., Burge, S.W., Bateman, A., Daub, J., Eberhardt, R.Y., Eddy, S.R., Floden, E.W., Gardner, P.P., Jones, T.A., Tate, J., et al. (2015). Rfam 12.0: Updates to the RNA families database. Nucleic Acids Res. *43*, D130–D137.
53. Elliott, G P (Department of Conservation, Nelson, N.Z. (2016). Personal communication.
54. Kerpedjiev, P., Abdennur, N., Lekschas, F., McCallum, C., Dinkla, K., Strobelt, H., Luber, J.M., Ouellette, S.B., Azhir, A., Kumar, N., et al. (2018). HiGlass: Web-based visual exploration and analysis of genome interaction maps. Genome Biol. *19*.
55. Schiffels, S., and Wang, K. (2020). MSMC and MSMC2: The multiple sequentially Markovian coalescent. Methods Mol. Biol. *2090*, 147–166.
56. Murray, G.G.R., Soares, A.E.R., Novak, B.J., Schaefer, N.K., Cahill, J.A., Baker, A.J., Demboski, J.R., Doll, A., Da Fonseca, R.R., Fulton, T.L., et al. (2017). Natural selection shaped the rise and fall of passenger pigeon genomic diversity. Science. *358*, 951–954.
57. Jarvis, E.D., Mirarab, S., Aberer, A.J., Li, B., Houde, P., Li, C., Ho, S.Y.W., Faircloth, B.C., Nabholz, B., Howard, J.T., et al. (2014). Whole-genome analyses resolve early branches in the tree of life of modern birds. Science. *346*, 1320–1331.
58. Weber, C.C., Boussau, B., Romiguier, J., Jarvis, E.D., and Ellegren, H. (2014). Evidence for GC-biased gene conversion as a driver of between-lineage differences in avian base composition. Genome Biol. *15*, 1–16.
59. Hedges, S.B., Marin, J., Suleski, M., Paymer, M., and Kumar, S. (2015). Tree of life reveals clock-like speciation and diversification. Mol. Biol. Evol. *32*, 835–845.
60. Valk, T. van der, Manuel, M. de, Marques-Bonet, T., and Guschanski, K. (2019). Estimates of genetic load in small populations suggest extensive purging of deleterious

alleles. bioRxiv, 696831.

61. Hedrick, P.W., and Garcia-Dorado, A. (2016). Understanding Inbreeding Depression, Purging, and Genetic Rescue. Trends Ecol. Evol. *31*, 940–952.

62. Wang, J., Hill, W.G., Charlesworth, D., and Charlesworth, B. (1999). Dynamics of inbreeding depression due to deleterious mutations in small populations: Mutation parameters and inbreeding rate. Genet. Res. *74*, 165–178.

63. Glémin, S. (2003). How are deleterious mutations purged? Drift versus nonrandom mating. Evolution (N. Y). *57*, 2678–87.

64. García-Dorado, A. (2012). Understanding and predicting the fitness decline of shrunk populations: Inbreeding, purging, mutation, and standard selection. Genetics *190*, 1461–1476.
